# Supplementary figures and images for: Possible favorable lifestyle changes owing to the coronavirus disease 2019 (COVID-19) pandemic among middle-aged Japanese women: An ancillary survey of the TRF-Japan study using the original “Taberhythm” smartphone app
Source: PLoS One. 2021 Mar 25;16(3):e0248935. doi: 10.1371/journal.pone.0248935 (PMC7993768; doi:10.1371/journal.pone.0248935)

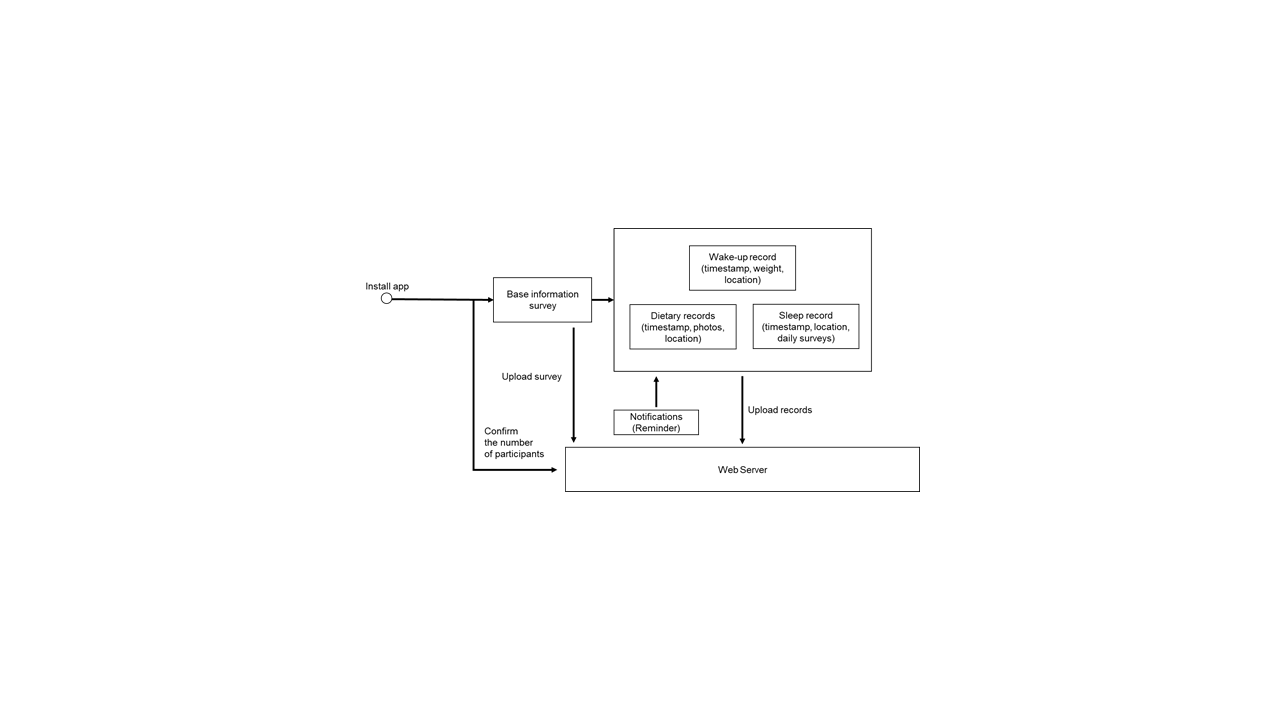

Supplement: S1 Fig — (TIF) [file pone.0248935.s001.tif]

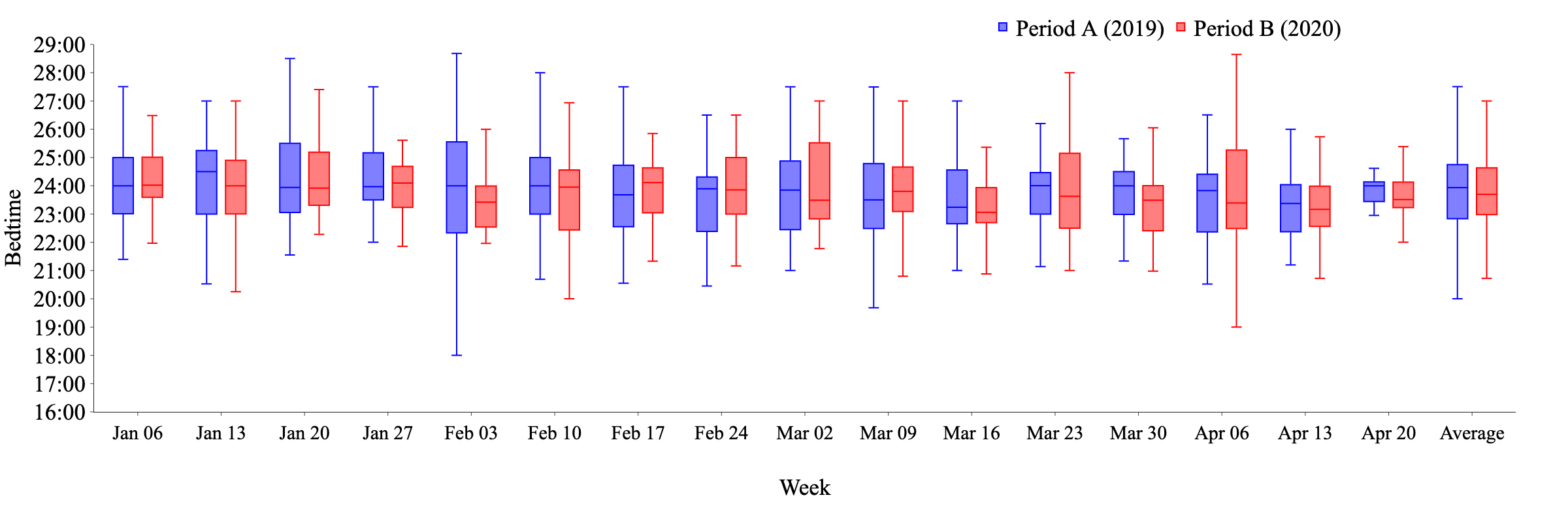

Supplement: S2 Fig — Distributions of all logged data for bedtimes are shown weekly from January 7 to April 28, 2019 (Period A; blue bars) and from January 6 to April 26, 2020 (Period B; red bars). There was no significant change in bedtimes between Period A and Period B. (TIF) [file pone.0248935.s002.tif]

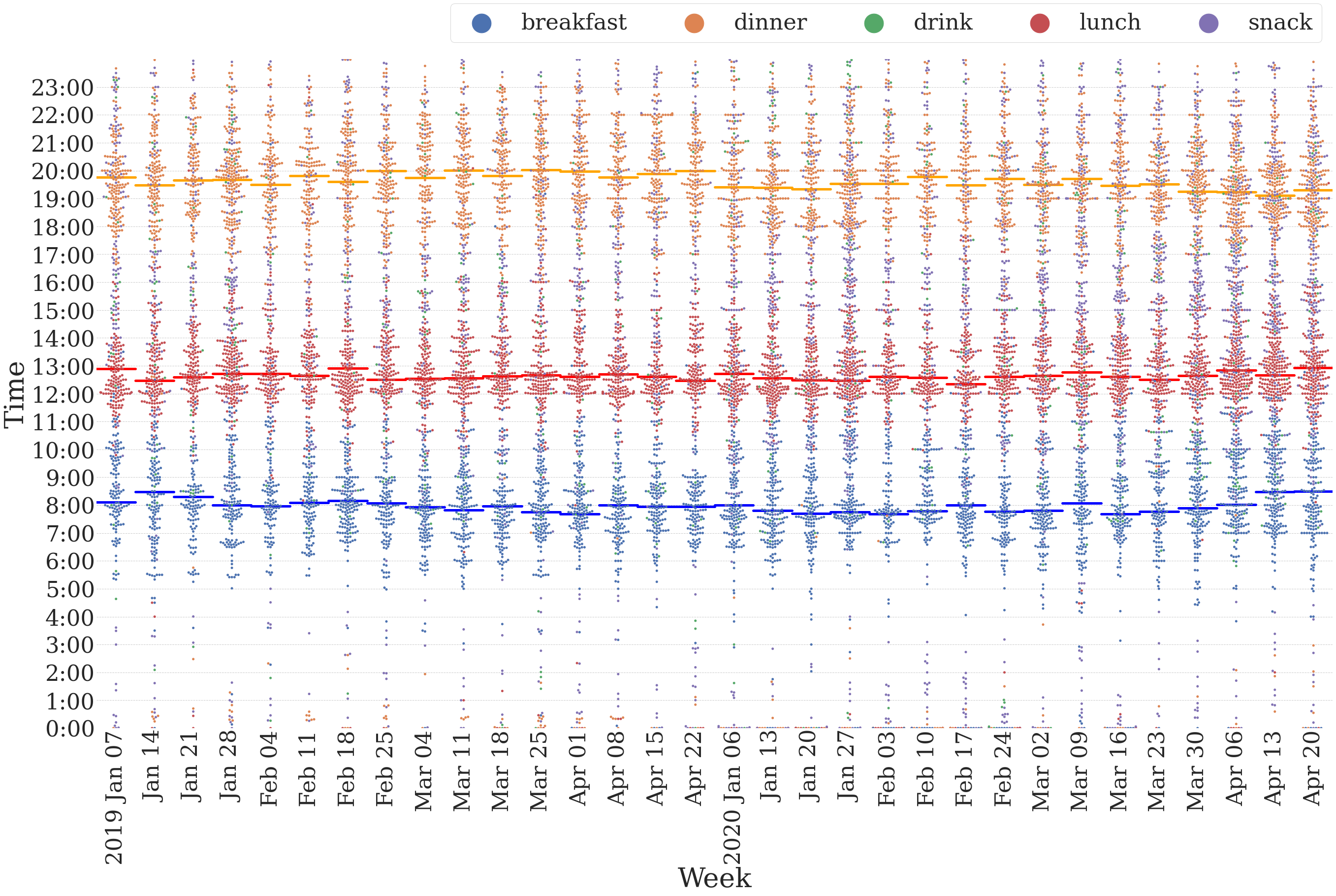

Supplement: S3 Fig — All the raw data for mealtimes are shown weekly as scatterplots from January 7 to April 28, 2019 (Period A) and from January 6 to April 26, 2020 (Period B). Five meal categories are shown as different color plots; blue: breakfast; red: lunch; orange: dinner; purple: snacks; green: drinks (calorie-containing). (TIF) [file pone.0248935.s003.tif]
