# Supplementary material for: Possible favorable lifestyle changes owing to the coronavirus disease 2019 (COVID-19) pandemic among middle-aged Japanese women: An ancillary survey of the TRF-Japan study using the original “Taberhythm” smartphone app
Source: PLoS One. 2021 Mar 25;16(3):e0248935. doi: 10.1371/journal.pone.0248935 (PMC7993768; doi:10.1371/journal.pone.0248935)
Supplement: S2 File — (PDF) [file pone.0248935.s005.pdf]

user_id	year	week	eating_duration_min	eating_duration_hour	gender	age	height
15	2019	7	658	11.0	male	69	164
15	2019	9	690	11.5	male	69	164
15	2019	10	720	12.0	male	69	164
100	2019	7	780	13.0	male	23	172
100	2019	2	825	13.8	male	23	172
100	2019	3	872	14.5	male	23	172
100	2019	4	924	15.4	male	23	172
100	2019	6	937	15.6	male	23	172
100	2019	5	964	16.1	male	23	172
250	2019	3	768	12.8	male	22	172
250	2019	2	919	15.3	male	22	172
499	2019	2	796	13.3	male	50	180
499	2019	13	811	13.5	male	50	180
499	2019	3	840	14.0	male	50	180
499	2019	12	840	14.0	male	50	180
643	2019	8	561	9.4	female	63	161
643	2019	14	569	9.5	female	63	161
643	2019	2	600	10.0	female	63	161
643	2019	3	611	10.2	female	63	161
643	2019	9	652	10.9	female	63	161
643	2019	16	670	11.2	female	63	161
643	2019	10	691	11.5	female	63	161
650	2019	3	470	7.8	male	59	162
650	2020	5	625	10.4	male	59	162
650	2019	4	680	11.3	male	59	162
650	2019	9	685	11.4	male	59	162
650	2019	5	690	11.5	male	59	162
650	2019	15	697	11.6	male	59	162
650	2019	6	701	11.7	male	59	162
650	2019	14	702	11.7	male	59	162
650	2019	8	707	11.8	male	59	162
650	2019	2	725	12.1	male	59	162
650	2019	17	746	12.4	male	59	162
650	2019	7	755	12.6	male	59	162
650	2019	12	793	13.2	male	59	162
650	2019	11	796	13.3	male	59	162
650	2019	13	818	13.6	male	59	162
650	2019	16	842	14.0	male	59	162

650	2019	10	869	14.5	male	59	162
724	2019	12	664	11.1	male	37	161
724	2019	15	777	13.0	male	37	161
724	2019	14	786	13.1	male	37	161
724	2019	17	799	13.3	male	37	161
724	2019	16	809	13.5	male	37	161
770	2020	11	758	12.6	male	39	173
833	2019	10	573	9.5	female	60	159
980	2019	3	521	8.7	female	44	159
980	2019	2	591	9.8	female	44	159
1042	2020	10	645	10.8	male	20	170
1042	2020	9	753	12.6	male	20	170
1190	2019	3	455	7.6	female	24	164
1190	2019	4	509	8.5	female	24	164
1190	2019	2	671	11.2	female	24	164
1229	2019	3	708	11.8	female	61	158
1229	2019	2	726	12.1	female	61	158
1268	2019	6	554	9.2	male	56	174
1268	2019	7	682	11.4	male	56	174
1268	2019	12	702	11.7	male	56	174
1268	2019	2	704	11.7	male	56	174
1268	2019	4	722	12.0	male	56	174
1268	2019	3	743	12.4	male	56	174
1268	2019	10	752	12.5	male	56	174
1268	2019	5	755	12.6	male	56	174
1268	2019	11	762	12.7	male	56	174
1268	2019	8	795	13.3	male	56	174
1268	2019	15	808	13.5	male	56	174
1268	2019	14	822	13.7	male	56	174
1268	2019	13	826	13.8	male	56	174
1611	2019	2	452	7.5	female	34	153
1611	2019	6	527	8.8	female	34	153
1611	2019	5	534	8.9	female	34	153
1611	2019	4	596	9.9	female	34	153
1611	2019	3	598	10.0	female	34	153
1802	2020	13	585	9.8	male	48	168
1802	2020	8	626	10.4	male	48	168
1802	2019	7	640	10.7	male	48	168
1802	2020	12	645	10.8	male	48	168

1802	2020	15	651	10.9	male	48	168
1802	2020	11	654	10.9	male	48	168
1802	2020	9	677	11.3	male	48	168
1802	2020	4	682	11.4	male	48	168
1802	2019	3	692	11.5	male	48	168
1802	2019	4	693	11.5	male	48	168
1802	2020	16	698	11.6	male	48	168
1802	2020	6	699	11.6	male	48	168
1802	2019	13	700	11.7	male	48	168
1802	2019	2	702	11.7	male	48	168
1802	2020	10	703	11.7	male	48	168
1802	2020	5	708	11.8	male	48	168
1802	2019	10	708	11.8	male	48	168
1802	2020	7	720	12.0	male	48	168
1802	2019	15	722	12.0	male	48	168
1802	2019	8	723	12.1	male	48	168
1802	2019	6	732	12.2	male	48	168
1802	2019	12	740	12.3	male	48	168
1802	2020	3	751	12.5	male	48	168
1802	2020	2	751	12.5	male	48	168
1802	2020	17	756	12.6	male	48	168
1802	2019	11	757	12.6	male	48	168
1802	2019	9	762	12.7	male	48	168
1802	2019	17	781	13.0	male	48	168
1802	2019	5	786	13.1	male	48	168
1802	2020	14	798	13.3	male	48	168
1802	2019	14	814	13.6	male	48	168
1802	2019	16	840	14.0	male	48	168
1965	2019	2	555	9.2	female	20	161
1965	2019	11	577	9.6	female	20	161
1965	2019	3	600	10.0	female	20	161
1965	2019	8	611	10.2	female	20	161
1965	2019	10	611	10.2	female	20	161
1965	2019	6	623	10.4	female	20	161
1965	2019	12	657	11.0	female	20	161
1965	2019	4	695	11.6	female	20	161
1965	2019	7	695	11.6	female	20	161
1965	2019	5	702	11.7	female	20	161
1965	2019	9	715	11.9	female	20	161

2031	2019	9	643	10.7	female	20	156
2524	2019	3	703	11.7	female	64	161
2524	2019	2	704	11.7	female	64	161
2816	2019	11	907	15.1	male	23	162
2816	2019	8	1070	17.8	male	23	162
2816	2019	14	1072	17.9	male	23	162
3006	2019	2	540	9.0	female	21	163
3006	2019	3	578	9.6	female	21	163
3068	2019	5	621	10.4	female	27	163
3068	2019	4	772	12.9	female	27	163
3122	2019	3	795	13.3	female	50	153
3124	2019	11	593	9.9	female	54	161
3124	2019	3	606	10.1	female	54	161
3124	2019	2	609	10.2	female	54	161
3124	2019	7	659	11.0	female	54	161
3124	2019	6	660	11.0	female	54	161
3124	2019	16	666	11.1	female	54	161
3124	2019	8	683	11.4	female	54	161
3124	2019	4	695	11.6	female	54	161
3124	2019	14	704	11.7	female	54	161
3124	2019	9	730	12.2	female	54	161
3124	2019	5	741	12.4	female	54	161
3124	2019	10	762	12.7	female	54	161
3124	2019	15	772	12.9	female	54	161
3333	2020	10	620	10.3	female	31	159
3333	2019	7	626	10.4	female	31	159
3333	2020	14	647	10.8	female	31	159
3333	2020	5	662	11.0	female	31	159
3333	2020	8	663	11.0	female	31	159
3333	2019	12	677	11.3	female	31	159
3333	2019	17	691	11.5	female	31	159
3333	2020	12	703	11.7	female	31	159
3333	2020	16	705	11.8	female	31	159
3333	2020	2	711	11.9	female	31	159
3333	2020	17	715	11.9	female	31	159
3333	2020	6	746	12.4	female	31	159
3333	2020	15	756	12.6	female	31	159
3333	2020	7	758	12.6	female	31	159
3333	2019	11	761	12.7	female	31	159

3333	2019	16	768	12.8	female	31	159
3333	2020	4	775	12.9	female	31	159
3333	2019	15	785	13.1	female	31	159
3333	2019	6	796	13.3	female	31	159
3333	2020	13	799	13.3	female	31	159
3333	2019	5	801	13.4	female	31	159
3333	2020	11	803	13.4	female	31	159
3333	2019	9	804	13.4	female	31	159
3333	2020	3	808	13.5	female	31	159
3333	2020	9	810	13.5	female	31	159
3333	2019	8	824	13.7	female	31	159
3333	2019	3	845	14.1	female	31	159
3333	2019	4	852	14.2	female	31	159
3333	2019	2	854	14.2	female	31	159
3333	2019	10	858	14.3	female	31	159
3333	2019	14	911	15.2	female	31	159
3333	2019	13	930	15.5	female	31	159
3335	2019	9	746	12.4	female	22	158
3344	2019	7	522	8.7	female	26	149
3344	2019	9	551	9.2	female	26	149
3344	2019	10	578	9.6	female	26	149
3344	2019	3	620	10.3	female	26	149
3344	2019	5	637	10.6	female	26	149
3344	2019	4	655	10.9	female	26	149
3344	2019	2	805	13.4	female	26	149
3344	2019	6	861	14.4	female	26	149
3367	2019	5	519	8.7	female	34	158
3367	2019	6	530	8.8	female	34	158
3367	2019	4	571	9.5	female	34	158
3367	2019	12	613	10.2	female	34	158
3367	2019	15	708	11.8	female	34	158
3370	2019	2	795	13.3	female	37	160
3386	2019	2	813	13.6	female	30	150
3387	2020	3	448	7.5	female	59	160
3387	2020	5	453	7.6	female	59	160
3387	2020	9	561	9.4	female	59	160
3387	2020	8	598	10.0	female	59	160
3387	2020	4	635	10.6	female	59	160
3387	2020	14	641	10.7	female	59	160

3387	2020	6	654	10.9	female	59	160
3387	2020	2	664	11.1	female	59	160
3387	2020	16	678	11.3	female	59	160
3387	2019	2	683	11.4	female	59	160
3387	2019	3	684	11.4	female	59	160
3387	2019	16	684	11.4	female	59	160
3387	2020	10	686	11.4	female	59	160
3387	2019	9	688	11.5	female	59	160
3387	2020	11	691	11.5	female	59	160
3387	2019	7	692	11.5	female	59	160
3387	2020	7	693	11.5	female	59	160
3387	2020	15	694	11.6	female	59	160
3387	2020	13	697	11.6	female	59	160
3387	2019	17	708	11.8	female	59	160
3387	2019	11	709	11.8	female	59	160
3387	2020	12	712	11.9	female	59	160
3387	2019	8	716	11.9	female	59	160
3387	2020	17	720	12.0	female	59	160
3387	2019	15	722	12.0	female	59	160
3387	2019	5	722	12.0	female	59	160
3387	2019	13	728	12.1	female	59	160
3387	2019	12	738	12.3	female	59	160
3387	2019	4	741	12.4	female	59	160
3387	2019	14	742	12.4	female	59	160
3387	2019	10	757	12.6	female	59	160
3389	2019	8	660	11.0	female	20	158
3389	2019	10	820	13.7	female	20	158
3389	2019	11	888	14.8	female	20	158
3389	2019	9	888	14.8	female	20	158
3393	2019	7	399	6.7	female	34	153
3393	2019	9	451	7.5	female	34	153
3393	2019	2	574	9.6	female	34	153
3393	2019	8	674	11.2	female	34	153
3393	2019	4	933	15.5	female	34	153
3393	2019	3	1061	17.7	female	34	153
3406	2019	4	677	11.3	female	47	167
3406	2019	3	685	11.4	female	47	167
3406	2019	2	685	11.4	female	47	167
3423	2019	2	728	12.1	female	20	160

3434	2019	3	641	10.7	female	32	166
3434	2019	2	776	12.9	female	32	166
3435	2019	2	817	13.6	male	29	181
3440	2019	2	804	13.4	female	46	157
3441	2019	2	625	10.4	female	27	158
3442	2019	2	606	10.1	female	20	158
3444	2019	2	926	15.4	female	20	161
3447	2019	2	672	11.2	female	51	171
3448	2019	2	535	8.9	female	41	156
3448	2019	3	557	9.3	female	41	156
3448	2019	12	583	9.7	female	41	156
3448	2019	8	586	9.8	female	41	156
3448	2019	7	600	10.0	female	41	156
3448	2019	4	621	10.3	female	41	156
3448	2019	5	660	11.0	female	41	156
3448	2019	17	664	11.1	female	41	156
3448	2019	11	709	11.8	female	41	156
3448	2019	6	713	11.9	female	41	156
3448	2019	16	719	12.0	female	41	156
3448	2019	10	721	12.0	female	41	156
3448	2019	13	731	12.2	female	41	156
3448	2019	9	743	12.4	female	41	156
3448	2019	15	777	13.0	female	41	156
3448	2019	14	968	16.1	female	41	156
3452	2019	3	733	12.2	female	33	155
3452	2019	2	747	12.4	female	33	155
3454	2019	2	848	14.1	female	43	154
3459	2019	4	779	13.0	female	44	164
3461	2019	3	723	12.0	female	27	151
3462	2019	3	1080	18.0	female	20	159
3463	2019	8	518	8.6	female	31	166
3463	2019	12	589	9.8	female	31	166
3463	2019	10	616	10.3	female	31	166
3463	2019	7	631	10.5	female	31	166
3463	2019	9	636	10.6	female	31	166
3463	2019	11	674	11.2	female	31	166
3463	2019	3	702	11.7	female	31	166
3463	2019	6	731	12.2	female	31	166
3463	2019	4	732	12.2	female	31	166

3463	2019	5	737	12.3	female	31	166
3470	2019	4	561	9.4	female	21	149
3470	2019	9	752	12.5	female	21	149
3470	2019	7	765	12.8	female	21	149
3470	2019	6	778	13.0	female	21	149
3470	2019	11	780	13.0	female	21	149
3470	2019	14	785	13.1	female	21	149
3470	2019	10	789	13.1	female	21	149
3470	2019	8	789	13.2	female	21	149
3470	2019	16	802	13.4	female	21	149
3470	2019	5	805	13.4	female	21	149
3470	2019	12	810	13.5	female	21	149
3470	2019	13	818	13.6	female	21	149
3470	2019	15	831	13.9	female	21	149
3470	2019	17	912	15.2	female	21	149
3476	2019	4	895	14.9	female	25	166
3479	2019	4	762	12.7	female	50	162
3480	2019	4	755	12.6	female	74	155
3482	2019	4	660	11.0	female	52	168
3487	2019	5	676	11.3	male	23	170
3487	2019	4	715	11.9	male	23	170
3490	2019	4	716	11.9	female	62	154
3494	2019	4	457	7.6	female	51	164
3504	2019	5	901	15.0	female	27	156
3506	2019	6	813	13.6	female	35	152
3506	2019	5	841	14.0	female	35	152
3508	2019	5	477	8.0	female	25	157
3509	2019	6	811	13.5	female	24	155
3509	2019	7	1042	17.4	female	24	155
3509	2019	5	1051	17.5	female	24	155
3509	2019	9	1258	21.0	female	24	155
3513	2019	5	675	11.3	female	23	166
3515	2019	5	764	12.7	female	33	170
3515	2019	7	770	12.8	female	33	170
3515	2019	6	816	13.6	female	33	170
3518	2019	6	741	12.3	male	25	161
3518	2019	8	786	13.1	male	25	161
3518	2019	7	790	13.2	male	25	161
3518	2019	5	799	13.3	male	25	161

3518	2019	9	821	13.7	male	25	161
3520	2019	5	842	14.0	female	22	156
3522	2019	6	633	10.6	female	45	160
3523	2019	14	707	11.8	male	53	172
3523	2019	6	713	11.9	male	53	172
3523	2019	12	767	12.8	male	53	172
3523	2019	8	770	12.8	male	53	172
3523	2019	17	780	13.0	male	53	172
3523	2019	15	802	13.4	male	53	172
3523	2019	10	803	13.4	male	53	172
3523	2019	9	804	13.4	male	53	172
3523	2019	11	808	13.5	male	53	172
3523	2019	13	835	13.9	male	53	172
3523	2019	16	870	14.5	male	53	172
3527	2019	6	669	11.2	female	42	158
3530	2019	6	699	11.7	female	48	154
3532	2019	6	728	12.1	female	25	157
3536	2019	7	851	14.2	male	25	156
3540	2019	7	439	7.3	female	32	158
3540	2019	12	503	8.4	female	32	158
3540	2019	8	579	9.7	female	32	158
3540	2019	11	591	9.9	female	32	158
3546	2019	12	536	8.9	female	48	151
3546	2019	7	604	10.1	female	48	151
3546	2019	8	644	10.7	female	48	151
3546	2019	9	699	11.7	female	48	151
3547	2019	7	763	12.7	female	22	152
3553	2019	8	727	12.1	female	47	158
3560	2019	8	640	10.7	female	25	159
3562	2019	14	488	8.1	female	36	160
3562	2019	11	513	8.6	female	36	160
3562	2019	10	561	9.3	female	36	160
3562	2019	13	588	9.8	female	36	160
3562	2019	8	630	10.5	female	36	160
3562	2019	12	656	10.9	female	36	160
3567	2019	8	630	10.5	female	49	160
3568	2019	8	541	9.0	female	47	158
3571	2019	8	554	9.2	female	20	170
3575	2019	8	644	10.7	female	40	161

3581	2019	9	726	12.1	male	43	170
3583	2019	16	657	11.0	male	23	175
3583	2019	14	716	11.9	male	23	175
3583	2019	10	781	13.0	male	23	175
3583	2019	12	782	13.0	male	23	175
3583	2019	13	792	13.2	male	23	175
3583	2019	11	855	14.2	male	23	175
3583	2019	9	855	14.3	male	23	175
3583	2019	17	907	15.1	male	23	175
3585	2019	9	773	12.9	female	45	157
3587	2019	9	526	8.8	female	29	152
3590	2019	15	745	12.4	female	56	161
3590	2019	16	750	12.5	female	56	161
3590	2019	17	758	12.6	female	56	161
3590	2019	14	769	12.8	female	56	161
3590	2019	9	781	13.0	female	56	161
3590	2019	12	791	13.2	female	56	161
3590	2019	10	799	13.3	female	56	161
3590	2019	13	836	13.9	female	56	161
3590	2019	11	844	14.1	female	56	161
3591	2019	9	665	11.1	female	27	156
3595	2019	10	628	10.5	female	27	160
3595	2019	9	742	12.4	female	27	160
3600	2019	9	508	8.5	male	51	177
3600	2019	13	768	12.8	male	51	177
3600	2019	11	802	13.4	male	51	177
3600	2019	14	814	13.6	male	51	177
3600	2019	10	831	13.8	male	51	177
3600	2019	12	943	15.7	male	51	177
3602	2019	9	759	12.6	female	36	163
3603	2019	10	663	11.0	male	45	165
3603	2019	13	720	12.0	male	45	165
3603	2019	14	810	13.5	male	45	165
3603	2019	12	903	15.1	male	45	165
3604	2019	9	849	14.2	male	25	175
3611	2019	13	661	11.0	male	28	168
3612	2019	10	909	15.2	female	24	156
3616	2019	10	760	12.7	female	30	153
3617	2019	10	1033	17.2	male	22	173

3620	2019	11	570	9.5	female	23	158
3623	2019	10	700	11.7	female	29	155
3625	2019	10	678	11.3	female	41	162
3634	2019	15	670	11.2	female	36	168
3634	2019	16	695	11.6	female	36	168
3634	2019	17	703	11.7	female	36	168
3634	2019	11	752	12.5	female	36	168
3634	2019	12	758	12.6	female	36	168
3634	2019	14	764	12.7	female	36	168
3634	2019	13	773	12.9	female	36	168
3635	2019	11	840	14.0	female	36	157
3637	2019	12	692	11.5	female	42	152
3637	2019	13	739	12.3	female	42	152
3637	2019	11	755	12.6	female	42	152
3646	2019	12	690	11.5	male	53	158
3646	2019	11	720	12.0	male	53	158
3649	2020	6	563	9.4	female	26	150
3649	2020	9	603	10.1	female	26	150
3649	2020	7	608	10.1	female	26	150
3649	2020	8	615	10.3	female	26	150
3649	2019	14	620	10.3	female	26	150
3649	2020	17	624	10.4	female	26	150
3649	2020	11	626	10.4	female	26	150
3649	2020	16	626	10.4	female	26	150
3649	2020	14	628	10.5	female	26	150
3649	2020	3	631	10.5	female	26	150
3649	2020	12	635	10.6	female	26	150
3649	2020	4	651	10.9	female	26	150
3649	2020	15	656	10.9	female	26	150
3649	2020	10	682	11.4	female	26	150
3649	2020	5	684	11.4	female	26	150
3649	2020	13	695	11.6	female	26	150
3649	2019	12	714	11.9	female	26	150
3649	2019	15	715	11.9	female	26	150
3649	2020	2	717	12.0	female	26	150
3649	2019	17	728	12.1	female	26	150
3649	2019	16	753	12.6	female	26	150
3650	2019	12	574	9.6	female	24	151
3652	2019	12	816	13.6	female	47	153

3661	2019	12	600	10.0	female	37	161
3662	2019	12	642	10.7	male	20	171
3663	2019	13	911	15.2	female	24	157
3663	2019	12	921	15.3	female	24	157
3666	2019	17	560	9.3	female	25	162
3666	2019	14	565	9.4	female	25	162
3666	2019	13	630	10.5	female	25	162
3666	2019	16	750	12.5	female	25	162
3667	2019	12	890	14.8	female	26	158
3683	2019	16	599	10.0	female	20	160
3683	2019	15	652	10.9	female	20	160
3683	2019	14	700	11.7	female	20	160
3683	2019	17	710	11.8	female	20	160
3683	2019	13	715	11.9	female	20	160
3688	2019	13	685	11.4	female	33	161
3691	2019	14	713	11.9	female	22	157
3693	2019	14	662	11.0	female	54	163
3693	2019	16	697	11.6	female	54	163
3693	2019	17	725	12.1	female	54	163
3693	2019	15	734	12.2	female	54	163
3694	2019	13	687	11.5	female	46	159
3694	2019	14	779	13.0	female	46	159
3696	2019	13	473	7.9	female	56	164
3696	2019	14	780	13.0	female	56	164
3699	2019	13	661	11.0	female	37	157
3701	2019	14	703	11.7	female	57	156
3702	2019	13	510	8.5	female	41	162
3702	2019	14	720	12.0	female	41	162
3705	2019	14	868	14.5	female	24	159
3709	2019	14	766	12.8	female	20	165
3710	2019	14	844	14.1	female	28	162
3726	2019	16	584	9.7	male	36	172
3726	2019	15	604	10.1	male	36	172
3726	2019	14	611	10.2	male	36	172
3726	2019	17	642	10.7	male	36	172
3731	2019	15	725	12.1	female	25	160
3733	2019	15	591	9.8	female	25	158
3733	2019	16	668	11.1	female	25	158
3741	2019	15	579	9.6	female	25	160

3741	2019	16	625	10.4	female	25	160
3741	2019	17	788	13.1	female	25	160
3744	2019	15	795	13.3	female	26	163
3751	2019	16	840	14.0	female	47	156
3751	2019	17	856	14.3	female	47	156
3753	2019	16	775	12.9	male	21	178
3754	2019	16	688	11.5	female	32	162
3759	2019	16	716	11.9	female	35	162
3767	2019	16	816	13.6	female	34	158
3768	2019	16	740	12.3	female	30	164
3774	2019	16	799	13.3	female	30	154
3778	2019	17	737	12.3	female	26	157
3779	2019	17	567	9.5	female	30	163
3781	2019	17	540	9.0	female	20	155
4078	2020	9	765	12.8	male	54	160
4078	2020	3	785	13.1	male	54	160
4078	2020	6	814	13.6	male	54	160
4078	2020	2	825	13.8	male	54	160
4078	2020	4	826	13.8	male	54	160
4078	2020	17	836	13.9	male	54	160
4078	2020	5	861	14.4	male	54	160
4078	2020	16	866	14.4	male	54	160
4078	2020	7	888	14.8	male	54	160
4078	2020	10	909	15.2	male	54	160
4078	2020	8	914	15.2	male	54	160
4078	2020	11	924	15.4	male	54	160
4078	2020	15	927	15.4	male	54	160
4078	2020	12	954	15.9	male	54	160
4078	2020	14	963	16.1	male	54	160
4078	2020	13	984	16.4	male	54	160
4159	2020	8	581	9.7	female	53	165
4159	2020	6	584	9.7	female	53	165
4159	2020	4	610	10.2	female	53	165
4159	2020	2	654	10.9	female	53	165
4159	2020	7	688	11.5	female	53	165
4159	2020	3	737	12.3	female	53	165
4343	2020	16	518	8.6	male	29	172
4343	2020	3	542	9.0	male	29	172
4343	2020	11	606	10.1	male	29	172

4343	2020	8	623	10.4	male	29	172
4343	2020	9	629	10.5	male	29	172
4343	2020	7	677	11.3	male	29	172
4343	2020	17	685	11.4	male	29	172
4343	2020	15	688	11.5	male	29	172
4343	2020	14	701	11.7	male	29	172
4343	2020	10	759	12.7	male	29	172
4343	2020	4	791	13.2	male	29	172
4343	2020	5	795	13.2	male	29	172
4343	2020	13	817	13.6	male	29	172
4343	2020	2	832	13.9	male	29	172
4645	2020	16	766	12.8	male	55	160
4673	2020	11	749	12.5	male	57	170
4673	2020	5	776	12.9	male	57	170
4673	2020	4	780	13.0	male	57	170
4673	2020	7	787	13.1	male	57	170
4673	2020	10	790	13.2	male	57	170
4673	2020	8	799	13.3	male	57	170
4673	2020	3	800	13.3	male	57	170
4673	2020	12	815	13.6	male	57	170
4673	2020	6	819	13.7	male	57	170
4673	2020	9	822	13.7	male	57	170
4673	2020	2	835	13.9	male	57	170
4701	2020	4	666	11.1	female	20	151
4701	2020	2	667	11.1	female	20	151
4701	2020	3	676	11.3	female	20	151
4701	2020	5	690	11.5	female	20	151
4701	2020	7	717	12.0	female	20	151
4701	2020	6	746	12.4	female	20	151
4737	2020	3	830	13.8	female	52	160
4737	2020	2	850	14.2	female	52	160
4737	2020	4	859	14.3	female	52	160
4744	2020	2	420	7.0	male	28	175
4760	2020	3	649	10.8	female	21	154
4760	2020	4	691	11.5	female	21	154
4760	2020	2	794	13.2	female	21	154
4768	2020	3	723	12.1	female	30	164
4777	2020	7	628	10.5	female	37	150
4777	2020	8	643	10.7	female	37	150

4777	2020	2	758	12.6	female	37	150
4777	2020	6	760	12.7	female	37	150
4777	2020	3	778	13.0	female	37	150
4777	2020	5	779	13.0	female	37	150
4777	2020	4	784	13.1	female	37	150
4779	2020	7	277	4.6	female	36	158
4779	2020	4	654	10.9	female	36	158
4779	2020	12	660	11.0	female	36	158
4779	2020	3	674	11.2	female	36	158
4779	2020	2	700	11.7	female	36	158
4779	2020	6	709	11.8	female	36	158
4802	2020	2	718	12.0	male	38	164
4814	2020	2	634	10.6	female	45	163
4814	2020	3	783	13.0	female	45	163
4817	2020	2	630	10.5	female	49	160
4822	2020	3	678	11.3	female	25	161
4826	2020	7	470	7.8	female	50	150
4826	2020	6	499	8.3	female	50	150
4826	2020	3	525	8.8	female	50	150
4826	2020	2	593	9.9	female	50	150
4826	2020	4	594	9.9	female	50	150
4834	2020	2	686	11.4	female	24	159
4835	2020	2	783	13.1	female	49	157
4840	2020	3	835	13.9	female	42	156
4840	2020	2	886	14.8	female	42	156
4842	2020	3	811	13.5	female	20	146
4842	2020	2	922	15.4	female	20	146
4845	2020	2	625	10.4	male	20	171
4851	2020	2	751	12.5	female	49	158
4857	2020	5	462	7.7	female	20	154
4857	2020	4	566	9.4	female	20	154
4857	2020	6	574	9.6	female	20	154
4857	2020	2	773	12.9	female	20	154
4870	2020	2	540	9.0	male	29	167
4872	2020	3	590	9.8	female	30	162
4875	2020	3	553	9.2	female	28	157
4881	2020	3	459	7.7	male	49	170
4884	2020	3	714	11.9	female	24	161
4884	2020	4	779	13.0	female	24	161

4890	2020	5	663	11.1	female	28	161
4894	2020	3	600	10.0	female	30	157
4910	2020	4	757	12.6	female	24	163
4912	2020	5	406	6.8	male	43	165
4912	2020	3	600	10.0	male	43	165
4912	2020	4	1019	17.0	male	43	165
4913	2020	5	669	11.1	female	32	162
4913	2020	3	675	11.3	female	32	162
4913	2020	4	686	11.4	female	32	162
4914	2020	4	723	12.1	female	33	166
4914	2020	5	727	12.1	female	33	166
4914	2020	6	790	13.2	female	33	166
4918	2020	5	675	11.3	female	33	145
4918	2020	4	703	11.7	female	33	145
4922	2020	4	719	12.0	female	34	153
4927	2020	4	749	12.5	female	20	159
4927	2020	5	776	12.9	female	20	159
4928	2020	4	780	13.0	female	28	163
4933	2020	4	809	13.5	female	30	153
4934	2020	5	551	9.2	female	34	164
4934	2020	4	668	11.1	female	34	164
4938	2020	4	418	7.0	female	30	164
4940	2020	6	792	13.2	female	22	166
4940	2020	5	902	15.0	female	22	166
4947	2020	5	639	10.7	female	26	161
4950	2020	7	623	10.4	female	32	157
4950	2020	6	842	14.0	female	32	157
4950	2020	5	850	14.2	female	32	157
4950	2020	4	860	14.3	female	32	157
4956	2020	5	657	11.0	female	20	153
4959	2020	8	580	9.7	male	45	163
4959	2020	6	600	10.0	male	45	163
4959	2020	5	632	10.5	male	45	163
4960	2020	5	711	11.9	male	39	175
4964	2020	5	585	9.8	female	37	153
4968	2020	9	892	14.9	female	100	163
4968	2020	7	893	14.9	female	100	163
4968	2020	6	1322	22.0	female	100	163
4972	2020	5	809	13.5	male	37	164

4975	2020	5	554	9.2	female	37	153
4978	2020	13	522	8.7	female	42	163
4978	2020	11	695	11.6	female	42	163
4978	2020	9	696	11.6	female	42	163
4978	2020	12	700	11.7	female	42	163
4978	2020	7	716	11.9	female	42	163
4978	2020	6	718	12.0	female	42	163
4978	2020	14	742	12.4	female	42	163
4978	2020	8	747	12.4	female	42	163
4978	2020	15	775	12.9	female	42	163
4978	2020	10	798	13.3	female	42	163
4978	2020	5	810	13.5	female	42	163
4979	2020	5	517	8.6	female	42	159
4982	2020	5	875	14.6	female	25	165
4984	2020	5	583	9.7	female	68	155
4999	2020	5	540	9.0	female	35	156
5000	2020	6	501	8.4	female	42	150
5005	2020	6	970	16.2	female	21	168
5011	2020	6	625	10.4	female	29	163
5012	2020	6	611	10.2	female	32	157
5021	2020	6	722	12.0	female	24	151
5024	2020	6	766	12.8	female	42	164
5025	2020	6	868	14.5	female	45	165
5026	2020	7	574	9.6	female	29	163
5026	2020	8	578	9.6	female	29	163
5029	2020	6	1229	20.5	male	21	168
5030	2020	6	645	10.8	female	23	166
5031	2020	6	540	9.0	female	35	160
5039	2020	6	585	9.8	female	40	163
5042	2020	7	660	11.0	female	23	155
5045	2020	7	762	12.7	female	23	153
5047	2020	8	675	11.3	female	42	158
5050	2020	13	659	11.0	female	20	156
5050	2020	7	682	11.4	female	20	156
5068	2020	11	425	7.1	female	23	165
5068	2020	9	514	8.6	female	23	165
5068	2020	7	593	9.9	female	23	165
5068	2020	8	607	10.1	female	23	165
5068	2020	14	675	11.3	female	23	165

5068	2020	15	844	14.1	female	23	165
5072	2020	8	695	11.6	female	33	158
5075	2020	8	546	9.1	female	20	149
5078	2020	8	568	9.5	female	28	165
5079	2020	8	402	6.7	female	36	157
5083	2020	8	929	15.5	female	20	159
5086	2020	9	623	10.4	female	47	152
5086	2020	8	765	12.8	female	47	152
5089	2020	10	699	11.7	female	50	157
5089	2020	8	704	11.7	female	50	157
5089	2020	9	744	12.4	female	50	157
5091	2020	8	733	12.2	female	35	159
5091	2020	9	752	12.5	female	35	159
5094	2020	8	890	14.8	female	24	167
5095	2020	8	810	13.5	female	25	168
5099	2020	16	616	10.3	female	30	161
5099	2020	17	651	10.9	female	30	161
5099	2020	15	659	11.0	female	30	161
5099	2020	10	728	12.1	female	30	161
5099	2020	14	745	12.4	female	30	161
5099	2020	12	759	12.7	female	30	161
5099	2020	9	783	13.0	female	30	161
5099	2020	13	790	13.2	female	30	161
5099	2020	11	859	14.3	female	30	161
5101	2020	11	509	8.5	female	20	163
5101	2020	10	548	9.1	female	20	163
5101	2020	8	687	11.5	female	20	163
5101	2020	9	720	12.0	female	20	163
5101	2020	12	800	13.3	female	20	163
5104	2020	9	739	12.3	female	24	162
5106	2020	9	510	8.5	female	42	155
5108	2020	9	654	10.9	female	22	166
5113	2020	10	780	13.0	female	21	151
5117	2020	11	473	7.9	female	20	166
5117	2020	10	623	10.4	female	20	166
5117	2020	9	923	15.4	female	20	166
5118	2020	10	571	9.5	female	32	157
5118	2020	9	693	11.6	female	32	157
5122	2020	9	658	11.0	female	37	168

5122	2020	15	670	11.2	female	37	168
5122	2020	13	711	11.8	female	37	168
5122	2020	14	737	12.3	female	37	168
5122	2020	17	751	12.5	female	37	168
5122	2020	11	753	12.5	female	37	168
5122	2020	16	758	12.6	female	37	168
5122	2020	12	798	13.3	female	37	168
5122	2020	10	800	13.3	female	37	168
5131	2020	9	760	12.7	female	20	154
5131	2020	10	1190	19.8	female	20	154
5133	2020	11	673	11.2	female	50	161
5133	2020	10	708	11.8	female	50	161
5135	2020	16	320	5.3	female	20	161
5135	2020	10	463	7.7	female	20	161
5135	2020	17	581	9.7	female	20	161
5135	2020	11	688	11.5	female	20	161
5135	2020	14	700	11.7	female	20	161
5137	2020	10	705	11.8	female	39	163
5141	2020	12	510	8.5	female	50	163
5141	2020	13	533	8.9	female	50	163
5141	2020	14	596	9.9	female	50	163
5141	2020	11	605	10.1	female	50	163
5141	2020	16	605	10.1	female	50	163
5141	2020	10	619	10.3	female	50	163
5141	2020	15	651	10.9	female	50	163
5146	2020	10	947	15.8	female	20	164
5150	2020	10	544	9.1	female	23	149
5152	2020	10	561	9.4	female	22	156
5152	2020	11	684	11.4	female	22	156
5157	2020	10	626	10.4	female	30	169
5157	2020	11	713	11.9	female	30	169
5159	2020	11	669	11.2	female	20	168
5169	2020	12	668	11.1	female	45	164
5169	2020	13	694	11.6	female	45	164
5169	2020	11	753	12.6	female	45	164
5170	2020	11	623	10.4	female	35	164
5172	2020	11	600	10.0	female	58	160
5172	2020	12	637	10.6	female	58	160
5172	2020	13	653	10.9	female	58	160

5174	2020	11	687	11.5	male	71	174
5174	2020	12	690	11.5	male	71	174
5183	2020	11	635	10.6	female	53	157
5183	2020	14	657	11.0	female	53	157
5183	2020	13	692	11.5	female	53	157
5183	2020	12	832	13.9	female	53	157
5185	2020	13	720	12.0	female	46	157
5185	2020	16	753	12.6	female	46	157
5185	2020	14	799	13.3	female	46	157
5185	2020	12	869	14.5	female	46	157
5185	2020	11	901	15.0	female	46	157
5186	2020	11	819	13.7	female	25	157
5202	2020	12	481	8.0	female	29	162
5210	2020	13	803	13.4	male	47	170
5215	2020	12	751	12.5	female	25	148
5216	2020	12	545	9.1	male	62	168
5216	2020	13	606	10.1	male	62	168
5217	2020	12	707	11.8	female	31	156
5220	2020	12	794	13.2	female	54	158
5222	2020	12	1030	17.2	female	25	159
5222	2020	13	1099	18.3	female	25	159
5240	2020	14	893	14.9	female	22	164
5240	2020	16	960	16.0	female	22	164
5240	2020	17	1037	17.3	female	22	164
5240	2020	13	1137	19.0	female	22	164
5240	2020	15	1153	19.2	female	22	164
5246	2020	13	600	10.0	female	43	158
5249	2020	13	886	14.8	female	35	159
5250	2020	14	540	9.0	female	22	161
5250	2020	13	720	12.0	female	22	161
5253	2020	13	660	11.0	female	36	157
5254	2020	13	775	12.9	female	52	161
5254	2020	14	940	15.7	female	52	161
5257	2020	14	735	12.3	female	48	158
5259	2020	14	673	11.2	female	24	156
5264	2020	14	795	13.3	male	45	173
5267	2020	17	471	7.9	female	30	159
5267	2020	15	538	9.0	female	30	159
5267	2020	16	719	12.0	female	30	159

5267	2020	14	737	12.3	female	30	159
5268	2020	14	860	14.3	male	33	172
5279	2020	14	689	11.5	female	20	160
5280	2020	14	634	10.6	female	23	156
5282	2020	16	595	9.9	female	43	157
5282	2020	14	640	10.7	female	43	157
5282	2020	15	681	11.3	female	43	157
5284	2020	17	537	8.9	female	20	151
5284	2020	16	590	9.8	female	20	151
5284	2020	15	697	11.6	female	20	151
5284	2020	14	889	14.8	female	20	151
5287	2020	17	489	8.2	female	31	166
5288	2020	15	718	12.0	female	24	160
5288	2020	14	720	12.0	female	24	160
5303	2020	15	629	10.5	female	20	160
5305	2020	15	644	10.7	female	25	157
5306	2020	17	489	8.2	female	26	160
5306	2020	16	527	8.8	female	26	160
5306	2020	15	594	9.9	female	26	160
5307	2020	15	522	8.7	male	24	170
5314	2020	15	780	13.0	female	20	162
5320	2020	15	633	10.5	female	26	165
5321	2020	15	789	13.2	female	26	163
5337	2020	15	664	11.1	female	20	157
5340	2020	16	765	12.7	female	26	163
5340	2020	17	795	13.2	female	26	163
5344	2020	15	750	12.5	female	32	165
5348	2020	16	571	9.5	female	28	169
5349	2020	15	573	9.6	female	34	155
5349	2020	17	623	10.4	female	34	155
5349	2020	16	741	12.3	female	34	155
5351	2020	16	673	11.2	female	53	163
5355	2020	16	582	9.7	female	39	159
5356	2020	16	527	8.8	female	56	150
5356	2020	15	690	11.5	female	56	150
5359	2020	16	615	10.3	female	26	155
5364	2020	16	547	9.1	female	20	158
5366	2020	15	540	9.0	female	55	150
5366	2020	16	660	11.0	female	55	150

5367	2020	16	590	9.8	female	55	150
5371	2020	16	949	15.8	female	29	148
5372	2020	17	637	10.6	female	23	158
5373	2020	17	706	11.8	female	32	152
5373	2020	16	758	12.6	female	32	152
5378	2020	16	680	11.3	male	52	170
5383	2020	17	482	8.0	female	20	155
5383	2020	16	567	9.5	female	20	155
5386	2020	16	668	11.1	female	46	155
5386	2020	17	693	11.6	female	46	155
5390	2020	16	650	10.8	female	22	165
5391	2020	16	651	10.9	female	29	162
5398	2020	16	513	8.5	female	29	157
5398	2020	17	515	8.6	female	29	157
5399	2020	16	720	12.0	female	31	165
5402	2020	16	570	9.5	female	21	161
5404	2020	17	594	9.9	female	27	157
5407	2020	17	567	9.5	female	55	150
5407	2020	16	627	10.5	female	55	150
5416	2020	17	751	12.5	female	26	168
5420	2020	17	659	11.0	female	23	160
5421	2020	17	683	11.4	female	42	162
5423	2020	17	553	9.2	male	38	170
5428	2020	17	780	13.0	female	23	168
5429	2020	17	698	11.6	female	20	160
5431	2020	17	728	12.1	female	53	157
5432	2020	17	598	10.0	male	23	181
5433	2020	17	677	11.3	female	23	167
5437	2020	17	725	12.1	female	25	149
5442	2020	17	768	12.8	female	25	160
5447	2020	17	646	10.8	female	29	162
5448	2020	17	686	11.4	female	39	162
5460	2020	17	763	12.7	female	29	151
5461	2020	17	730	12.2	female	20	163
5462	2020	17	838	14.0	female	22	160
5462	2020	16	842	14.0	female	22	160
5470	2020	17	570	9.5	female	39	160
5471	2020	17	885	14.8	female	25	153
5476	2020	17	633	10.6	male	26	168

weight	bmi
70.5	26.2
70.5	26.2
70.5	26.2
65.0	22.0
65.0	22.0
65.0	22.0
65.0	22.0
65.0	22.0
65.0	22.0
56.0	18.9
56.0	18.9
72.0	22.2
72.0	22.2
72.0	22.2
72.0	22.2
49.0	18.9
49.0	18.9
49.0	18.9
49.0	18.9
49.0	18.9
49.0	18.9
49.0	18.9
54.5	20.8
54.5	20.8
54.5	20.8
54.5	20.8
54.5	20.8
54.5	20.8
54.5	20.8
54.5	20.8
54.5	20.8
54.5	20.8
54.5	20.8
54.5	20.8
54.5	20.8
54.5	20.8
54.5	20.8
54.5	20.8

54.5	20.8
65.0	25.1
65.0	25.1
65.0	25.1
65.0	25.1
65.0	25.1
69.0	23.1
75.0	29.7
55.3	21.9
55.3	21.9
60.0	20.8
60.0	20.8
47.0	17.5
47.0	17.5
47.0	17.5
74.2	29.7
74.2	29.7
98.8	32.6
98.8	32.6
98.8	32.6
98.8	32.6
98.8	32.6
98.8	32.6
98.8	32.6
98.8	32.6
98.8	32.6
98.8	32.6
98.8	32.6
98.8	32.6
98.8	32.6
53.0	22.6
53.0	22.6
53.0	22.6
53.0	22.6
53.0	22.6
82.0	29.1
82.0	29.1
82.0	29.1
82.0	29.1

82.0	29.1
82.0	29.1
82.0	29.1
82.0	29.1
82.0	29.1
82.0	29.1
82.0	29.1
82.0	29.1
82.0	29.1
82.0	29.1
82.0	29.1
82.0	29.1
82.0	29.1
82.0	29.1
82.0	29.1
82.0	29.1
82.0	29.1
82.0	29.1
82.0	29.1
82.0	29.1
82.0	29.1
82.0	29.1
82.0	29.1
82.0	29.1
82.0	29.1
82.0	29.1
82.0	29.1
82.0	29.1
53.7	20.7
53.7	20.7
53.7	20.7
53.7	20.7
53.7	20.7
53.7	20.7
53.7	20.7
53.7	20.7
53.7	20.7
53.7	20.7
53.7	20.7

61.0	25.1
53.0	20.4
53.0	20.4
80.0	30.5
80.0	30.5
80.0	30.5
50.0	18.8
50.0	18.8
65.0	24.5
65.0	24.5
55.0	23.5
52.6	20.3
52.6	20.3
52.6	20.3
52.6	20.3
52.6	20.3
52.6	20.3
52.6	20.3
52.6	20.3
52.6	20.3
52.6	20.3
52.6	20.3
52.6	20.3
52.6	20.3
59.9	23.7
59.9	23.7
59.9	23.7
59.9	23.7
59.9	23.7
59.9	23.7
59.9	23.7
59.9	23.7
59.9	23.7
59.9	23.7
59.9	23.7
59.9	23.7
59.9	23.7
59.9	23.7
59.9	23.7

59.9	23.7
59.9	23.7
59.9	23.7
59.9	23.7
59.9	23.7
59.9	23.7
59.9	23.7
59.9	23.7
59.9	23.7
59.9	23.7
59.9	23.7
59.9	23.7
59.9	23.7
59.9	23.7
59.9	23.7
59.9	23.7
59.9	23.7
56.0	22.4
43.0	19.4
43.0	19.4
43.0	19.4
43.0	19.4
43.0	19.4
43.0	19.4
43.0	19.4
43.0	19.4
49.0	19.6
49.0	19.6
49.0	19.6
49.0	19.6
49.0	19.6
95.0	37.1
50.0	22.2
73.5	28.7
73.5	28.7
73.5	28.7
73.5	28.7
73.5	28.7
73.5	28.7

73.5	28.7
73.5	28.7
73.5	28.7
73.5	28.7
73.5	28.7
73.5	28.7
73.5	28.7
73.5	28.7
73.5	28.7
73.5	28.7
73.5	28.7
73.5	28.7
73.5	28.7
73.5	28.7
73.5	28.7
73.5	28.7
73.5	28.7
73.5	28.7
73.5	28.7
73.5	28.7
73.5	28.7
73.5	28.7
73.5	28.7
73.5	28.7
73.5	28.7
49.0	19.6
49.0	19.6
49.0	19.6
49.0	19.6
57.0	24.3
57.0	24.3
57.0	24.3
57.0	24.3
57.0	24.3
57.0	24.3
77.0	27.6
77.0	27.6
77.0	27.6
44.0	17.2

73.0	26.5
73.0	26.5
76.5	23.4
69.0	28.0
51.0	20.4
95.0	38.1
58.0	22.4
60.0	20.5
58.0	23.8
58.0	23.8
58.0	23.8
58.0	23.8
58.0	23.8
58.0	23.8
58.0	23.8
58.0	23.8
58.0	23.8
58.0	23.8
58.0	23.8
58.0	23.8
58.0	23.8
58.0	23.8
58.0	23.8
58.0	23.8
89.0	37.0
89.0	37.0
63.5	26.8
71.0	26.4
49.0	21.5
60.0	23.7
120.0	43.5
120.0	43.5
120.0	43.5
120.0	43.5
120.0	43.5
120.0	43.5
120.0	43.5
120.0	43.5
120.0	43.5

120.0	43.5
43.0	19.4
43.0	19.4
43.0	19.4
43.0	19.4
43.0	19.4
43.0	19.4
43.0	19.4
43.0	19.4
43.0	19.4
43.0	19.4
43.0	19.4
43.0	19.4
43.0	19.4
43.0	19.4
69.0	25.0
65.8	25.1
53.0	22.1
71.6	25.4
53.0	18.3
53.0	18.3
51.5	21.7
61.0	22.7
56.0	23.0
47.0	20.3
47.0	20.3
75.8	30.8
50.0	20.8
50.0	20.8
50.0	20.8
50.0	20.8
55.0	20.0
60.0	20.8
60.0	20.8
60.0	20.8
56.0	21.6
56.0	21.6
56.0	21.6
56.0	21.6

56.0	21.6
50.0	20.5
55.0	21.5
92.0	31.1
92.0	31.1
92.0	31.1
92.0	31.1
92.0	31.1
92.0	31.1
92.0	31.1
92.0	31.1
92.0	31.1
92.0	31.1
92.0	31.1
51.0	20.4
74.0	31.2
49.0	19.9
47.0	19.3
53.0	21.2
53.0	21.2
53.0	21.2
53.0	21.2
55.0	24.1
55.0	24.1
55.0	24.1
55.0	24.1
52.0	22.5
61.4	24.6
56.0	22.2
51.0	19.9
51.0	19.9
51.0	19.9
51.0	19.9
51.0	19.9
51.0	19.9
84.4	33.0
61.5	24.6
55.0	19.0
60.0	23.1

71.0	24.6
70.5	23.0
70.5	23.0
70.5	23.0
70.5	23.0
70.5	23.0
70.5	23.0
70.5	23.0
70.5	23.0
74.0	30.0
45.0	19.5
61.0	23.5
61.0	23.5
61.0	23.5
61.0	23.5
61.0	23.5
61.0	23.5
61.0	23.5
61.0	23.5
61.0	23.5
48.5	19.9
50.0	19.5
50.0	19.5
93.0	29.7
93.0	29.7
93.0	29.7
93.0	29.7
93.0	29.7
93.0	29.7
77.0	29.0
63.0	23.1
63.0	23.1
63.0	23.1
63.0	23.1
63.0	20.6
55.0	19.5
50.0	20.5
47.0	20.1
57.5	19.2

76.0	30.4
57.0	23.7
115.0	43.8
56.1	19.9
56.1	19.9
56.1	19.9
56.1	19.9
56.1	19.9
56.1	19.9
56.1	19.9
46.0	18.7
54.6	23.6
54.6	23.6
54.6	23.6
58.0	23.2
58.0	23.2
46.6	20.7
46.6	20.7
46.6	20.7
46.6	20.7
46.6	20.7
46.6	20.7
46.6	20.7
46.6	20.7
46.6	20.7
46.6	20.7
46.6	20.7
46.6	20.7
46.6	20.7
46.6	20.7
46.6	20.7
46.6	20.7
46.6	20.7
46.6	20.7
46.6	20.7
46.6	20.7
46.6	20.7
48.9	21.4
46.5	19.9

57.0	22.0
49.0	16.8
59.0	23.9
59.0	23.9
40.0	15.2
40.0	15.2
40.0	15.2
40.0	15.2
57.0	22.8
53.2	20.8
53.2	20.8
53.2	20.8
53.2	20.8
53.2	20.8
55.0	21.2
53.0	21.5
48.0	18.1
48.0	18.1
48.0	18.1
48.0	18.1
55.0	21.8
55.0	21.8
78.0	29.0
78.0	29.0
52.0	21.1
44.0	18.1
115.0	43.8
115.0	43.8
52.0	20.6
59.0	21.7
45.0	17.1
60.0	20.3
60.0	20.3
60.0	20.3
60.0	20.3
55.0	21.5
55.0	22.0
55.0	22.0
47.0	18.4

47.0	18.4
47.0	18.4
61.0	23.0
49.0	20.1
49.0	20.1
68.0	21.5
56.0	21.3
51.0	19.4
53.0	21.2
86.0	32.0
54.0	22.8
52.0	21.1
52.5	19.8
50.0	20.8
59.0	23.0
59.0	23.0
59.0	23.0
59.0	23.0
59.0	23.0
59.0	23.0
59.0	23.0
59.0	23.0
59.0	23.0
59.0	23.0
59.0	23.0
59.0	23.0
59.0	23.0
59.0	23.0
59.0	23.0
59.0	23.0
49.0	18.0
49.0	18.0
49.0	18.0
49.0	18.0
49.0	18.0
49.0	18.0
60.0	20.3
60.0	20.3
60.0	20.3

60.0	20.3
60.0	20.3
60.0	20.3
60.0	20.3
60.0	20.3
60.0	20.3
60.0	20.3
60.0	20.3
60.0	20.3
60.0	20.3
60.0	20.3
56.0	21.9
52.0	18.0
52.0	18.0
52.0	18.0
52.0	18.0
52.0	18.0
52.0	18.0
52.0	18.0
52.0	18.0
52.0	18.0
52.0	18.0
52.0	18.0
46.7	20.5
46.7	20.5
46.7	20.5
46.7	20.5
46.7	20.5
46.7	20.5
60.0	23.4
60.0	23.4
60.0	23.4
81.0	26.4
47.2	19.9
47.2	19.9
47.2	19.9
54.0	20.1
53.0	23.6
53.0	23.6

53.0	23.6
53.0	23.6
53.0	23.6
53.0	23.6
53.0	23.6
59.0	23.6
59.0	23.6
59.0	23.6
59.0	23.6
59.0	23.6
59.0	23.6
65.0	24.2
49.0	18.4
49.0	18.4
57.0	22.3
62.0	23.9
66.0	29.3
66.0	29.3
66.0	29.3
66.0	29.3
66.0	29.3
91.0	36.0
59.0	23.9
67.8	27.9
67.8	27.9
50.0	23.5
50.0	23.5
73.0	25.0
70.0	28.0
58.0	24.5
58.0	24.5
58.0	24.5
58.0	24.5
76.0	27.3
96.0	36.6
51.0	20.7
64.0	22.1
59.6	23.0
59.6	23.0

56.9	22.0
48.0	19.5
52.0	19.6
74.5	27.4
74.5	27.4
74.5	27.4
52.0	19.8
52.0	19.8
52.0	19.8
84.0	30.5
84.0	30.5
84.0	30.5
48.0	22.8
48.0	22.8
48.0	20.5
45.0	17.8
45.0	17.8
63.0	23.7
60.0	25.6
62.0	23.1
62.0	23.1
60.5	22.5
59.0	21.4
59.0	21.4
53.5	20.6
77.0	31.2
77.0	31.2
77.0	31.2
77.0	31.2
73.0	31.2
50.0	18.8
50.0	18.8
50.0	18.8
85.0	27.8
52.2	22.3
100.0	37.6
100.0	37.6
100.0	37.6
54.0	20.1

51.0	21.8
53.0	19.9
53.0	19.9
53.0	19.9
53.0	19.9
53.0	19.9
53.0	19.9
53.0	19.9
53.0	19.9
53.0	19.9
53.0	19.9
53.0	19.9
60.4	23.9
53.0	19.5
58.0	24.1
74.0	30.4
60.0	26.7
54.0	19.1
54.0	20.3
62.0	25.2
46.7	20.5
68.0	25.3
61.0	22.4
41.0	15.4
41.0	15.4
80.0	28.3
57.0	20.7
54.0	21.1
64.0	24.1
48.0	20.0
58.0	24.8
71.0	28.4
55.8	22.9
55.8	22.9
75.3	27.7
75.3	27.7
75.3	27.7
75.3	27.7
75.3	27.7

75.3	27.7
48.0	19.2
51.0	23.0
55.2	20.3
66.1	26.8
49.0	19.4
45.0	19.5
45.0	19.5
63.0	25.6
63.0	25.6
63.0	25.6
65.0	25.7
65.0	25.7
105.0	37.6
58.0	20.5
65.0	25.1
65.0	25.1
65.0	25.1
65.0	25.1
65.0	25.1
65.0	25.1
65.0	25.1
65.0	25.1
65.0	25.1
52.0	19.6
52.0	19.6
52.0	19.6
52.0	19.6
52.0	19.6
67.3	25.6
53.0	22.1
53.0	19.2
55.0	24.1
65.0	23.6
65.0	23.6
65.0	23.6
61.0	24.7
61.0	24.7
60.2	21.3

60.2	21.3
60.2	21.3
60.2	21.3
60.2	21.3
60.2	21.3
60.2	21.3
60.2	21.3
60.2	21.3
43.5	18.3
43.5	18.3
53.0	20.4
53.0	20.4
48.5	18.7
48.5	18.7
48.5	18.7
48.5	18.7
48.5	18.7
50.0	18.8
63.0	23.7
63.0	23.7
63.0	23.7
63.0	23.7
63.0	23.7
63.0	23.7
63.0	23.7
57.0	21.2
44.0	19.8
68.5	28.1
68.5	28.1
56.0	19.6
56.0	19.6
58.0	20.5
69.0	25.7
69.0	25.7
69.0	25.7
73.0	27.1
59.5	23.2
59.5	23.2
59.5	23.2

87.0	28.7
87.0	28.7
62.0	25.2
62.0	25.2
62.0	25.2
62.0	25.2
57.0	23.1
57.0	23.1
57.0	23.1
57.0	23.1
57.0	23.1
51.0	20.7
55.0	21.0
68.0	23.5
44.0	20.1
64.0	22.7
64.0	22.7
64.4	26.5
55.6	22.3
57.8	22.9
57.8	22.9
55.0	20.4
55.0	20.4
55.0	20.4
55.0	20.4
55.0	20.4
75.0	30.0
64.4	25.5
57.0	22.0
57.0	22.0
57.0	23.1
69.7	26.9
69.7	26.9
62.5	25.0
52.3	21.5
92.0	30.7
46.5	18.4
46.5	18.4
46.5	18.4

46.5	18.4
63.0	21.3
53.8	21.0
55.0	22.6
53.0	21.5
53.0	21.5
53.0	21.5
59.2	26.0
59.2	26.0
59.2	26.0
59.2	26.0
62.0	22.5
58.9	23.0
58.9	23.0
53.0	20.7
51.0	20.7
50.0	19.5
50.0	19.5
50.0	19.5
65.0	22.5
62.8	23.9
60.0	22.0
49.0	18.4
48.0	19.5
50.0	18.8
50.0	18.8
62.0	22.8
56.3	19.7
66.0	27.5
66.0	27.5
66.0	27.5
54.0	20.3
49.0	19.4
55.0	24.4
55.0	24.4
59.0	24.6
59.0	23.6
55.0	24.4
55.0	24.4

55.0	24.4
45.8	20.9
50.8	20.3
55.0	23.8
55.0	23.8
82.0	28.4
53.3	22.2
53.3	22.2
52.0	21.6
52.0	21.6
57.0	20.9
54.7	20.8
64.0	26.0
64.0	26.0
65.0	23.9
61.0	23.5
62.5	25.4
55.0	24.4
55.0	24.4
58.0	20.5
55.0	21.5
48.0	18.3
78.0	27.0
66.0	23.4
50.5	19.7
59.0	23.9
75.0	22.9
60.0	21.5
62.0	27.9
63.0	24.6
54.0	20.6
63.0	24.0
43.3	19.0
60.0	22.6
41.0	16.0
41.0	16.0
57.0	22.3
48.5	20.7
78.0	27.6
