# Supplementary material for: Possible favorable lifestyle changes owing to the coronavirus disease 2019 (COVID-19) pandemic among middle-aged Japanese women: An ancillary survey of the TRF-Japan study using the original “Taberhythm” smartphone app
Source: PLoS One. 2021 Mar 25;16(3):e0248935. doi: 10.1371/journal.pone.0248935 (PMC7993768; doi:10.1371/journal.pone.0248935)
Supplement: S1 File — (PDF) [file pone.0248935.s004.pdf]

	user_id	Female0	age	height	weight	bmi	wake_up_2019_2	wake_up_2019_3
0	6	1	20	178	64.0	20.2	.	.
1	15	1	69	164	70.5	26.2	.	.
2	16	1	43	171	73.0	25.0	.	.
3	26	1	42	168	78.5	27.8	.	.
4	30	0	28	161	49.0	18.9	.	.
5	38	0	36	154	45.0	19.0	.	.
6	43	0	36	157	42.0	17.0	.	.
7	64	1	33	179	68.0	21.2	.	.
8	79	1	32	172	75.0	25.4	.	.
9	87	1	44	166	66.0	24.0	.	.
10	100	1	23	172	65.0	22.0	322	396
11	103	1	45	176	75.0	24.2	.	.
12	106	0	23	156	44.9	18.5	.	.
13	112	1	23	167	64.0	22.9	.	500
14	122	1	26	173	66.0	22.1	.	.
15	132	0	42	165	49.0	18.0	.	.
16	134	0	34	163	52.0	19.6	.	.
17	155	0	41	153	59.0	25.2	.	345
18	157	0	42	160	45.0	17.6	.	.
19	168	0	37	151	59.0	25.9	.	.
20	182	1	29	170	62.0	21.5	.	.
21	186	1	28	173	63.0	21.0	.	.
22	187	1	35	172	74.0	25.0	.	.
23	202	0	65	155	57.7	24.0	.	.
24	224	1	53	162	64.0	24.4	.	.
25	227	1	60	167	71.0	25.5	304	.
26	250	1	22	172	56.0	18.9	.	.
27	261	1	35	170	66.0	22.8	.	.
28	266	0	41	159	49.0	19.4	.	.
29	269	0	50	155	50.0	20.8	.	.
30	319	1	27	163	79.0	29.7	.	.
31	324	1	42	177	79.0	25.2	.	.
32	358	0	27	172	78.0	26.4	.	.
33	381	0	51	156	73.0	30.0	.	.
34	387	1	36	159	68.5	27.1	.	.
35	410	1	32	178	66.0	20.8	.	.
36	425	0	24	167	63.0	22.6	.	.
37	496	1	35	180	86.0	26.5	.	.
38	499	1	50	180	72.0	22.2	.	.
39	643	0	63	161	49.0	18.9	.	.
40	650	1	59	162	54.5	20.8	350	440
41	658	0	40	155	75.0	31.2	.	.
42	670	1	50	168	53.0	18.8	.	.
43	722	1	53	171	84.0	28.7	.	.
44	724	1	37	161	65.0	25.1	240	390
45	732	1	43	179	87.0	27.2	.	.
46	770	1	39	173	69.0	23.1	.	.
47	776	1	55	171	68.1	23.3	.	.
48	791	0	37	160	44.2	17.3	367	.
49	833	0	60	159	75.0	29.7	.	375
50	836	0	59	159	55.0	21.8	.	.
51	882	0	43	172	57.0	19.3	.	.
52	893	1	44	180	96.0	29.6	.	.
53	942	1	46	165	64.0	23.5	.	.
54	948	0	29	154	47.0	19.8	.	.
55	980	0	44	159	55.3	21.9	.	.
56	1008	1	48	173	108.0	36.1	.	.
57	1021	0	20	156	54.8	22.5	.	.

58	1029	1	27	171	70.3	24.0	.	.
59	1042	1	20	170	60.0	20.8	.	.
60	1043	0	39	159	108.8	43.0	.	.
61	1051	0	25	155	56.0	23.3	.	.
62	1132	0	27	156	48.0	19.7	.	.
63	1190	0	24	164	47.0	17.5	.	.
64	1212	0	28	158	45.0	18.0	.	.
65	1229	0	61	158	74.2	29.7	425	448
66	1251	1	57	161	54.0	20.8	.	.
67	1268	1	56	174	98.8	32.6	456	434
68	1292	0	28	158	48.0	19.2	.	.
69	1365	0	40	149	48.0	21.6	.	.
70	1379	1	27	172	74.0	25.0	.	.
71	1406	1	36	177	72.0	23.0	.	.
72	1429	1	44	172	88.0	29.7	.	.
73	1438	0	26	156	52.0	21.4	.	.
74	1513	1	38	176	110.0	35.5	.	.
75	1527	1	20	170	60.0	20.8	.	.
76	1569	0	26	160	73.2	28.6	.	.
77	1582	0	37	161	47.0	18.1	.	.
78	1611	0	34	153	53.0	22.6	.	.
79	1614	0	24	149	58.2	26.2	.	.
80	1633	1	27	168	57.0	20.2	.	.
81	1653	0	25	156	43.0	17.7	.	.
82	1677	0	21	163	56.0	21.1	.	.
83	1702	0	59	162	46.0	17.5	.	.
84	1707	0	24	158	84.0	33.6	.	.
85	1765	0	30	157	50.0	20.3	.	.
86	1802	1	48	168	82.0	29.1	337	308
87	1812	1	20	170	91.0	31.5	.	.
88	1875	1	43	169	75.0	26.3	.	.
89	1900	0	29	168	72.0	25.5	.	.
90	1965	0	20	161	53.7	20.7	440	402
91	1969	0	47	164	83.0	30.9	.	.
92	1978	0	68	153	60.0	25.6	.	.
93	2018	1	25	174	62.0	20.5	.	.
94	2031	0	20	156	61.0	25.1	.	.
95	2137	1	51	169	75.0	26.3	.	.
96	2140	0	36	160	55.0	21.5	.	.
97	2228	0	40	149	42.0	18.9	.	.
98	2250	0	27	161	48.0	18.5	.	.
99	2258	0	23	154	67.0	28.3	.	.
100	2263	0	23	165	53.5	19.7	.	.
101	2363	0	47	160	74.0	28.9	.	.
102	2369	0	23	149	71.0	32.0	.	.
103	2377	0	25	159	55.0	21.8	.	.
104	2383	1	60	170	78.0	27.0	.	.
105	2469	1	27	159	93.0	36.8	.	.
106	2524	0	64	161	53.0	20.4	396	407
107	2561	0	20	150	60.0	26.7	.	.
108	2569	1	28	174	69.0	22.8	.	.
109	2571	0	43	161	95.5	36.8	440	398
110	2599	0	35	167	64.0	22.9	.	.
111	2696	1	54	172	54.0	18.3	.	.
112	2702	0	58	156	47.0	19.3	.	.
113	2705	0	22	162	53.7	20.5	.	.
114	2710	0	52	160	62.0	24.2	.	.
115	2715	0	36	160	54.0	21.1	449	.
116	2722	1	56	164	89.2	33.2	.	.

117	2736	1	29	179	106.5	33.2	.	.
118	2737	1	23	166	61.0	22.1	.	.
119	2749	0	24	151	58.0	25.4	.	.
120	2751	1	61	168	64.0	22.7	.	.
121	2754	1	40	181	103.0	31.4	.	.
122	2774	1	21	174	84.0	27.7	.	.
123	2816	1	23	162	80.0	30.5	.	.
124	2817	1	21	172	75.0	25.4	.	.
125	2904	0	51	171	77.0	26.3	.	.
126	2943	0	25	158	56.0	22.4	.	.
127	2973	0	21	167	56.0	20.1	.	.
128	3006	0	21	163	50.0	18.8	.	.
129	3058	1	49	170	63.0	21.8	.	.
130	3060	1	43	174	75.0	24.8	.	.
131	3067	0	42	158	60.2	24.1	.	.
132	3068	0	27	163	65.0	24.5	.	.
133	3092	0	20	160	46.4	18.1	.	.
134	3120	0	32	162	121.2	46.2	.	.
135	3122	0	50	153	55.0	23.5	383	355
136	3124	0	54	161	52.6	20.3	502	554
137	3138	0	34	159	61.5	24.3	.	.
138	3177	0	23	151	42.0	18.4	.	.
139	3194	1	27	173	74.0	24.7	.	.
140	3217	1	54	165	77.0	28.3	.	.
141	3288	1	22	168	68.0	24.1	.	.
142	3296	1	48	181	100.0	30.5	.	.
143	3301	0	30	151	58.0	25.4	.	.
144	3308	0	42	158	48.0	19.2	.	.
145	3333	0	31	159	59.9	23.7	486	471
146	3335	0	22	158	56.0	22.4	.	.
147	3344	0	26	149	43.0	19.4	.	.
148	3345	1	63	167	65.0	23.3	.	.
149	3367	0	34	158	49.0	19.6	.	.
150	3369	0	58	161	53.0	20.4	.	.
151	3370	0	37	160	95.0	37.1	309	.
152	3372	1	63	158	58.9	23.6	.	.
153	3376	0	46	156	53.0	21.8	.	.
154	3379	0	37	150	51.0	22.7	.	.
155	3380	0	30	171	46.0	15.7	.	.
156	3386	0	30	150	50.0	22.2	.	.
157	3387	0	59	160	73.5	28.7	467	446
158	3389	0	20	158	49.0	19.6	.	.
159	3392	0	41	164	53.6	19.9	380	.
160	3393	0	34	153	57.0	24.3	.	.
161	3399	0	20	151	51.0	22.4	452	.
162	3401	0	36	154	61.0	25.7	595	.
163	3406	0	47	167	77.0	27.6	411	444
164	3416	1	37	164	53.0	19.7	.	.
165	3418	1	44	180	60.0	18.5	.	.
166	3423	0	20	160	44.0	17.2	.	.
167	3434	0	32	166	73.0	26.5	.	.
168	3435	1	29	181	76.5	23.4	.	.
169	3440	0	46	157	69.0	28.0	393	.
170	3441	0	27	158	51.0	20.4	502	.
171	3442	0	20	158	95.0	38.1	537	.
172	3444	0	20	161	58.0	22.4	382	.
173	3445	0	38	145	32.5	15.5	.	.
174	3447	0	51	171	60.0	20.5	330	.
175	3448	0	41	156	58.0	23.8	377	.

176	3449	0	25	146	50.0	23.5		460	.	
177	3452	0	33	155	89.0	37.0		380		454
178	3453	0	20	172	62.0	21.0		435	.	
179	3454	0	43	154	63.5	26.8		399		502
180	3455	0	31	158	80.0	32.0		390	.	
181	3456	1	42	168	70.4	24.9	.		.	
182	3457	1	20	180	60.0	18.5	.		.	
183	3459	0	44	164	71.0	26.4		310		430
184	3460	0	39	153	45.0	19.2	.			360
185	3461	0	27	151	49.0	21.5	.			484
186	3462	0	20	159	60.0	23.7	.			400
187	3463	0	31	166	120.0	43.5	.			432
188	3464	0	26	142	49.0	24.3	.		.	
189	3465	0	35	160	82.9	32.4	.			464
190	3466	0	21	168	87.0	30.8	.		.	
191	3468	0	24	153	45.0	19.2	.		.	
192	3469	1	70	169	75.1	26.3	.			405
193	3470	0	21	149	43.0	19.4	.			420
194	3472	0	27	150	47.0	20.9	.		.	
195	3473	0	20	150	55.0	24.4	.			360
196	3474	0	49	164	55.0	20.4	.			500
197	3475	0	23	148	52.0	23.7	.		.	
198	3476	0	25	166	69.0	25.0	.			390
199	3477	0	46	167	65.0	23.3	.		.	
200	3478	0	39	165	61.8	22.7	.		.	
201	3479	0	50	162	65.8	25.1	.		.	
202	3480	0	74	155	53.0	22.1	.		.	
203	3481	0	38	159	54.0	21.4	.		.	
204	3482	0	52	168	71.6	25.4	.		.	
205	3483	0	25	152	52.0	22.5	.		.	
206	3484	1	40	168	60.0	21.3	.		.	
207	3485	0	24	159	67.0	26.5	.		.	
208	3486	0	23	157	52.0	21.1	.		.	
209	3487	1	23	170	53.0	18.3	.		.	
210	3488	0	26	162	68.0	25.9	.		.	
211	3489	0	20	161	65.0	25.1	.		.	
212	3490	0	62	154	51.5	21.7	.		.	
213	3491	0	47	150	58.0	25.8	.		.	
214	3492	0	26	163	55.0	20.7	.		.	
215	3493	0	28	167	61.0	21.9	.		.	
216	3494	0	51	164	61.0	22.7	.		.	
217	3495	0	32	162	55.0	21.0	.		.	
218	3497	0	21	153	52.0	22.2	.		.	
219	3499	0	53	157	66.6	27.0	.		.	
220	3500	0	34	160	76.0	29.7	.		.	
221	3501	1	22	174	65.0	21.5	.		.	
222	3502	0	22	163	63.0	23.7	.		.	
223	3503	0	25	165	69.0	25.3	.		.	
224	3504	0	27	156	56.0	23.0	.		.	
225	3505	0	26	156	60.0	24.7	.		.	
226	3506	0	35	152	47.0	20.3	.		.	
227	3508	0	25	157	75.8	30.8	.		.	
228	3509	0	24	155	50.0	20.8	.		.	
229	3512	0	51	159	82.0	32.4	.		.	
230	3513	0	23	166	55.0	20.0	.		.	
231	3515	0	33	170	60.0	20.8	.		.	
232	3517	0	28	160	53.0	20.7	.		.	
233	3518	1	25	161	56.0	21.6	.		.	
234	3520	0	22	156	50.0	20.5	.		.	

235	3521	0	32	150	49.5	22.0	.	.
236	3522	0	45	160	55.0	21.5	.	.
237	3523	1	53	172	92.0	31.1	.	.
238	3524	0	32	155	51.5	21.4	.	.
239	3525	0	32	161	51.5	19.9	.	.
240	3526	0	27	170	70.0	24.2	.	.
241	3527	0	42	158	51.0	20.4	.	.
242	3528	0	22	163	48.0	18.1	.	.
243	3529	0	22	163	48.0	18.1	.	.
244	3530	0	48	154	74.0	31.2	.	.
245	3531	1	22	172	67.0	22.6	.	.
246	3532	0	25	157	49.0	19.9	.	.
247	3536	1	25	156	47.0	19.3	.	.
248	3537	0	40	152	47.0	20.3	.	.
249	3538	0	25	156	42.0	17.3	.	.
250	3540	0	32	158	53.0	21.2	.	.
251	3543	0	20	159	65.0	25.7	.	.
252	3544	0	25	157	65.0	26.4	.	.
253	3545	0	20	159	50.8	20.1	.	.
254	3546	0	48	151	55.0	24.1	.	.
255	3547	0	22	152	52.0	22.5	.	.
256	3548	0	24	165	46.0	16.9	.	.
257	3549	0	29	160	46.8	18.3	.	.
258	3550	1	49	180	68.0	21.0	.	.
259	3552	0	47	158	61.0	24.4	.	.
260	3553	0	47	158	61.4	24.6	.	.
261	3554	0	49	164	45.0	16.7	.	.
262	3555	0	44	157	57.0	23.1	.	.
263	3556	0	40	157	47.0	19.1	.	.
264	3558	0	20	153	63.2	27.0	.	.
265	3560	0	25	159	56.0	22.2	.	.
266	3561	0	55	163	70.0	26.3	.	.
267	3562	0	36	160	51.0	19.9	.	.
268	3563	0	27	160	36.0	14.1	.	.
269	3564	0	22	165	63.0	23.1	.	.
270	3565	0	38	150	64.0	28.4	.	.
271	3567	0	49	160	84.4	33.0	.	.
272	3568	0	47	158	61.5	24.6	.	.
273	3569	0	34	152	55.0	23.8	.	.
274	3570	0	65	155	58.0	24.1	.	.
275	3571	0	20	170	55.0	19.0	.	.
276	3572	0	45	170	50.0	17.3	.	.
277	3573	0	24	165	59.5	21.9	.	.
278	3574	0	24	171	57.0	19.5	.	.
279	3575	0	40	161	60.0	23.1	.	.
280	3576	0	25	153	59.0	25.2	.	.
281	3577	0	22	151	58.0	25.4	.	.
282	3578	0	27	158	56.0	22.4	.	.
283	3580	0	44	165	52.0	19.1	.	.
284	3581	1	43	170	71.0	24.6	.	.
285	3582	1	58	165	51.0	18.7	.	.
286	3583	1	23	175	70.5	23.0	.	.
287	3584	1	55	171	83.0	28.4	.	.
288	3585	0	45	157	74.0	30.0	.	.
289	3587	0	29	152	45.0	19.5	.	.
290	3588	0	36	152	52.0	22.5	.	.
291	3589	0	27	160	53.0	20.7	.	.
292	3590	0	56	161	61.0	23.5	.	.
293	3591	0	27	156	48.5	19.9	.	.

294	3593	0	24	161	50.1	19.3	.	.
295	3594	1	36	172	69.0	23.3	.	.
296	3595	0	27	160	50.0	19.5	.	.
297	3596	0	24	163	57.0	21.5	.	.
298	3597	0	29	157	46.0	18.7	.	.
299	3598	0	24	152	49.0	21.2	.	.
300	3599	1	26	169	90.0	31.5	.	.
301	3600	1	51	177	93.0	29.7	.	.
302	3601	0	21	163	70.0	26.3	.	.
303	3602	0	36	163	77.0	29.0	.	.
304	3603	1	45	165	63.0	23.1	.	.
305	3604	1	25	175	63.0	20.6	.	.
306	3605	0	52	160	87.0	34.0	.	.
307	3606	0	21	164	65.0	24.2	.	.
308	3608	0	38	163	60.2	22.7	.	.
309	3609	0	37	156	60.0	24.7	.	.
310	3610	0	21	169	64.0	22.4	.	.
311	3611	1	28	168	55.0	19.5	.	.
312	3612	0	24	156	50.0	20.5	.	.
313	3615	0	50	167	58.0	20.8	.	.
314	3616	0	30	153	47.0	20.1	.	.
315	3617	1	22	173	57.5	19.2	.	.
316	3619	1	34	181	84.0	25.6	.	.
317	3620	0	23	158	76.0	30.4	.	.
318	3621	0	28	162	51.3	19.5	.	.
319	3622	0	36	156	59.3	24.4	.	.
320	3623	0	29	155	57.0	23.7	.	.
321	3624	0	24	155	31.0	12.9	.	.
322	3625	0	41	162	115.0	43.8	.	.
323	3626	0	20	168	52.0	18.4	.	.
324	3627	1	46	170	68.7	23.8	.	.
325	3628	1	47	174	90.0	29.7	.	.
326	3630	0	21	158	48.0	19.2	.	.
327	3631	0	22	159	52.8	20.9	.	.
328	3632	0	21	158	49.0	19.6	.	.
329	3633	1	37	175	104.0	34.0	.	.
330	3634	0	36	168	56.1	19.9	.	.
331	3635	0	36	157	46.0	18.7	.	.
332	3636	0	27	160	51.0	19.9	.	.
333	3637	0	42	152	54.6	23.6	.	.
334	3639	0	22	159	80.0	31.6	.	.
335	3640	0	26	148	63.0	28.8	.	.
336	3641	0	36	160	74.6	29.1	.	.
337	3642	0	26	164	59.5	22.1	.	.
338	3643	1	35	173	56.0	18.7	.	.
339	3644	1	41	163	65.0	24.5	.	.
340	3645	1	34	179	81.0	25.3	.	.
341	3646	1	53	158	58.0	23.2	.	.
342	3647	0	46	166	65.0	23.6	.	.
343	3648	0	23	157	63.0	25.6	.	.
344	3649	0	26	150	46.6	20.7	.	.
345	3650	0	24	151	48.9	21.4	.	.
346	3651	0	36	150	55.0	24.4	.	.
347	3652	0	47	153	46.5	19.9	.	.
348	3653	0	33	157	75.0	30.4	.	.
349	3654	0	33	162	83.0	31.6	.	.
350	3655	0	20	154	53.0	22.3	.	.
351	3656	0	26	150	49.0	21.8	.	.
352	3657	0	24	158	53.8	21.6	.	.

353	3658	1	55	169	87.0	30.5	.	.
354	3659	0	25	158	63.8	25.6	.	.
355	3660	0	24	167	60.0	21.5	.	.
356	3661	0	37	161	57.0	22.0	.	.
357	3662	1	20	171	49.0	16.8	.	.
358	3663	0	24	157	59.0	23.9	.	.
359	3664	0	26	158	53.5	21.4	.	.
360	3666	0	25	162	40.0	15.2	.	.
361	3667	0	26	158	57.0	22.8	.	.
362	3668	1	23	165	64.0	23.5	.	.
363	3669	0	30	160	53.0	20.7	.	.
364	3671	0	30	156	47.0	19.3	.	.
365	3672	1	33	170	64.0	22.1	.	.
366	3673	0	30	161	55.0	21.2	.	.
367	3674	0	30	162	68.0	25.9	.	.
368	3675	0	20	162	57.0	21.7	.	.
369	3676	0	24	152	47.0	20.3	.	.
370	3677	0	34	163	51.5	19.4	.	.
371	3680	0	20	157	60.0	24.3	.	.
372	3682	0	33	157	63.0	25.6	.	.
373	3683	0	20	160	53.2	20.8	.	.
374	3684	0	44	153	53.0	22.6	.	.
375	3686	0	32	164	53.0	19.7	.	.
376	3687	1	43	174	62.0	20.5	.	.
377	3688	0	33	161	55.0	21.2	.	.
378	3689	0	27	161	59.5	23.0	.	.
379	3690	0	32	165	58.0	21.3	.	.
380	3691	0	22	157	53.0	21.5	.	.
381	3692	0	34	165	60.0	22.0	.	.
382	3693	0	54	163	48.0	18.1	.	.
383	3694	0	46	159	55.0	21.8	.	.
384	3696	0	56	164	78.0	29.0	.	.
385	3697	1	57	176	70.0	22.6	.	.
386	3698	0	20	158	50.0	20.0	.	.
387	3699	0	37	157	52.0	21.1	.	.
388	3700	0	23	155	48.0	20.0	.	.
389	3701	0	57	156	44.0	18.1	.	.
390	3702	0	41	162	115.0	43.8	.	.
391	3703	0	20	158	56.6	22.7	.	.
392	3704	0	26	163	67.5	25.4	.	.
393	3705	0	24	159	52.0	20.6	.	.
394	3706	0	24	153	42.7	18.2	.	.
395	3707	1	36	169	76.0	26.6	.	.
396	3709	0	20	165	59.0	21.7	.	.
397	3710	0	28	162	45.0	17.1	.	.
398	3711	0	28	152	42.0	18.2	.	.
399	3713	0	27	153	53.0	22.6	.	.
400	3715	1	53	164	77.0	28.6	.	.
401	3716	0	26	159	55.0	21.8	.	.
402	3717	0	59	160	56.0	21.9	.	.
403	3718	0	34	159	56.0	22.2	.	.
404	3719	0	24	153	44.8	19.1	.	.
405	3720	0	20	157	58.0	23.5	.	.
406	3722	0	33	160	49.0	19.1	.	.
407	3723	0	26	163	55.0	20.7	.	.
408	3724	0	48	160	58.0	22.7	.	.
409	3725	0	28	155	44.0	18.3	.	.
410	3726	1	36	172	60.0	20.3	.	.
411	3728	0	31	159	47.0	18.6	.	.

412	3729	0	23	153	46.0	19.7	.	.
413	3731	0	25	160	55.0	21.5	.	.
414	3732	0	38	163	63.0	23.7	.	.
415	3733	0	25	158	55.0	22.0	.	.
416	3734	0	20	150	47.0	20.9	.	.
417	3735	0	21	157	52.0	21.1	.	.
418	3736	0	46	155	48.0	20.0	.	.
419	3737	0	50	150	51.0	22.7	.	.
420	3738	0	49	164	52.0	19.3	.	.
421	3739	0	20	172	70.0	23.7	.	.
422	3741	0	25	160	47.0	18.4	.	.
423	3742	0	29	159	73.0	28.9	.	.
424	3743	0	27	162	60.0	22.9	.	.
425	3744	0	26	163	61.0	23.0	.	.
426	3745	0	31	159	48.0	19.0	.	.
427	3746	0	31	152	54.0	23.4	.	.
428	3747	1	53	171	79.5	27.2	.	.
429	3748	0	29	157	50.0	20.3	.	.
430	3750	0	33	167	56.5	20.3	.	.
431	3751	0	47	156	49.0	20.1	.	.
432	3752	0	33	167	58.8	21.1	.	.
433	3753	1	21	178	68.0	21.5	.	.
434	3754	0	32	162	56.0	21.3	.	.
435	3755	0	21	159	63.0	24.9	.	.
436	3756	1	60	175	88.0	28.7	.	.
437	3758	0	35	162	51.0	19.4	.	.
438	3759	0	35	162	51.0	19.4	.	.
439	3760	0	36	167	53.0	19.0	.	.
440	3761	0	25	168	65.0	23.0	.	.
441	3763	1	20	160	50.0	19.5	.	.
442	3764	0	26	157	49.0	19.9	.	.
443	3765	1	23	170	60.0	20.8	.	.
444	3766	1	49	165	68.0	25.0	.	.
445	3767	0	34	158	53.0	21.2	.	.
446	3768	0	30	164	86.0	32.0	.	.
447	3770	0	47	167	62.6	22.4	.	.
448	3772	1	61	181	75.0	22.9	.	.
449	3773	0	23	155	51.0	21.2	.	.
450	3774	0	30	154	54.0	22.8	.	.
451	3775	0	30	168	64.0	22.7	.	.
452	3776	0	46	162	51.0	19.4	.	.
453	3777	0	22	157	60.0	24.3	.	.
454	3778	0	26	157	52.0	21.1	.	.
455	3779	0	30	163	52.5	19.8	.	.
456	3780	0	27	153	50.6	21.6	.	.
457	3781	0	20	155	50.0	20.8	.	.
458	3783	0	40	161	46.0	17.7	.	.
459	3785	0	35	155	56.0	23.3	.	.
460	3786	0	56	158	43.0	17.2	.	.
461	3787	0	41	164	80.0	29.7	.	.
462	3788	0	35	160	85.0	33.2	.	.
463	3789	0	20	163	49.0	18.4	.	.
464	3830	0	41	150	48.0	21.3	.	.
465	3869	0	43	158	45.0	18.0	.	.
466	3952	0	47	161	85.6	33.0	.	.
467	3972	0	45	162	63.0	24.0	.	.
468	3986	1	20	160	60.0	23.4	.	.
469	4030	0	33	150	54.5	24.2	.	.
470	4078	1	54	160	59.0	23.0	.	.

471	4103	0	27	155	63.0	26.2	.	.
472	4136	0	22	152	44.0	19.0	.	.
473	4159	0	53	165	49.0	18.0	.	.
474	4218	0	38	161	75.0	28.9	.	.
475	4243	0	28	153	80.8	34.5	.	.
476	4264	0	47	154	73.0	30.8	.	.
477	4337	0	22	157	54.0	21.9	.	.
478	4343	1	29	172	60.0	20.3	.	.
479	4349	1	20	171	56.0	19.2	.	.
480	4392	0	36	155	55.0	22.9	.	.
481	4415	1	23	170	75.0	26.0	.	.
482	4431	0	30	162	48.0	18.3	.	.
483	4435	0	31	155	74.8	31.1	.	.
484	4454	0	20	130	24.0	14.2	.	.
485	4495	1	29	181	85.0	25.9	.	.
486	4503	1	26	177	65.0	20.7	.	.
487	4529	0	34	153	58.0	24.8	.	.
488	4532	0	33	157	50.0	20.3	.	.
489	4536	1	45	167	59.2	21.2	.	.
490	4537	1	30	177	75.0	23.9	.	.
491	4543	1	20	177	82.0	26.2	.	.
492	4577	1	37	158	52.0	20.8	.	.
493	4586	0	23	154	53.1	22.4	.	.
494	4615	0	22	162	66.0	25.1	.	.
495	4623	0	48	156	63.0	25.9	.	.
496	4630	0	44	151	65.0	28.5	.	.
497	4645	1	55	160	56.0	21.9	.	.
498	4646	1	43	172	85.0	28.7	.	.
499	4672	0	28	150	39.0	17.3	.	.
500	4673	1	57	170	52.0	18.0	.	.
501	4676	1	38	166	70.0	25.4	.	.
502	4683	0	27	165	60.0	22.0	.	.
503	4688	0	43	157	48.0	19.5	.	.
504	4701	0	20	151	46.7	20.5	.	.
505	4708	0	20	170	55.0	19.0	.	.
506	4729	0	42	155	73.0	30.4	.	.
507	4737	0	52	160	60.0	23.4	.	.
508	4744	1	28	175	81.0	26.4	.	.
509	4754	1	47	185	81.0	23.7	.	.
510	4755	0	32	163	47.0	17.7	.	.
511	4760	0	21	154	47.2	19.9	.	.
512	4768	0	30	164	54.0	20.1	.	.
513	4777	0	37	150	53.0	23.6	.	.
514	4779	0	36	158	59.0	23.6	.	.
515	4783	0	28	157	57.0	23.1	.	.
516	4789	0	21	160	51.0	19.9	.	.
517	4800	0	45	159	55.0	21.8	.	.
518	4801	0	24	165	58.0	21.3	.	.
519	4802	1	38	164	65.0	24.2	.	.
520	4805	1	22	170	63.0	21.8	.	.
521	4806	0	49	160	48.0	18.7	.	.
522	4807	0	21	148	48.0	21.9	.	.
523	4810	0	33	162	41.0	15.6	.	.
524	4811	0	23	158	58.5	23.4	.	.
525	4814	0	45	163	49.0	18.4	.	.
526	4815	0	39	152	75.0	32.5	.	.
527	4816	0	22	150	46.2	20.5	.	.
528	4817	0	49	160	57.0	22.3	.	.
529	4818	0	43	162	75.2	28.7	.	.

530	4819	0	23	153	53.2	22.7	.	.
531	4820	0	55	160	47.0	18.4	.	.
532	4821	0	32	164	48.0	17.8	.	.
533	4822	0	25	161	62.0	23.9	.	.
534	4823	0	27	164	57.0	21.2	.	.
535	4826	0	50	150	66.0	29.3	.	.
536	4827	0	31	162	59.0	22.5	.	.
537	4828	0	32	167	55.0	19.7	.	.
538	4830	1	46	174	65.7	21.7	.	.
539	4832	0	32	164	48.0	17.8	.	.
540	4833	0	24	156	58.0	23.8	.	.
541	4834	0	24	159	91.0	36.0	.	.
542	4835	0	49	157	59.0	23.9	.	.
543	4836	0	32	156	48.0	19.7	.	.
544	4837	0	34	146	27.0	12.7	.	.
545	4838	1	38	173	72.0	24.1	.	.
546	4839	0	25	156	47.5	19.5	.	.
547	4840	0	42	156	67.8	27.9	.	.
548	4841	1	36	168	100.0	35.4	.	.
549	4842	0	20	146	50.0	23.5	.	.
550	4843	1	42	178	80.0	25.2	.	.
551	4844	0	58	159	70.0	27.7	.	.
552	4845	1	20	171	73.0	25.0	.	.
553	4846	0	55	160	46.0	18.0	.	.
554	4847	0	30	154	47.0	19.8	.	.
555	4848	0	20	153	54.0	23.1	.	.
556	4849	0	20	175	62.0	20.2	.	.
557	4850	0	33	155	50.0	20.8	.	.
558	4851	0	49	158	70.0	28.0	.	.
559	4852	0	48	161	59.0	22.8	.	.
560	4855	0	42	150	58.0	25.8	.	.
561	4856	0	35	160	54.5	21.3	.	.
562	4857	0	20	154	58.0	24.5	.	.
563	4861	1	42	185	92.0	26.9	.	.
564	4862	0	42	150	58.0	25.8	.	.
565	4863	0	24	172	60.0	20.3	.	.
566	4864	0	20	142	58.3	28.9	.	.
567	4865	0	31	160	56.0	21.9	.	.
568	4866	0	20	160	63.0	24.6	.	.
569	4867	0	32	162	67.7	25.8	.	.
570	4868	0	23	171	72.0	24.6	.	.
571	4869	0	38	158	66.0	26.4	.	.
572	4870	1	29	167	76.0	27.3	.	.
573	4872	0	30	162	96.0	36.6	.	.
574	4873	0	32	170	54.0	18.7	.	.
575	4874	0	46	164	65.5	24.4	.	.
576	4875	0	28	157	51.0	20.7	.	.
577	4876	0	37	164	96.0	35.7	.	.
578	4877	1	28	175	82.0	26.8	.	.
579	4878	0	21	161	57.0	22.0	.	.
580	4879	1	20	160	80.0	31.2	.	.
581	4880	1	22	175	80.0	26.1	.	.
582	4881	1	49	170	64.0	22.1	.	.
583	4882	0	20	165	54.0	19.8	.	.
584	4883	0	20	156	51.0	21.0	.	.
585	4884	0	24	161	59.6	23.0	.	.
586	4885	0	53	162	99.0	37.7	.	.
587	4886	1	20	160	80.0	31.2	.	.
588	4887	0	20	160	70.0	27.3	.	.

589	4889	0	48	155	63.0	26.2	.	.
590	4890	0	28	161	56.9	22.0	.	.
591	4891	1	20	170	80.0	27.7	.	.
592	4892	0	26	156	59.1	24.3	.	.
593	4893	0	28	154	50.0	21.1	.	.
594	4894	0	30	157	48.0	19.5	.	.
595	4895	0	30	167	54.8	19.6	.	.
596	4897	0	25	157	49.9	20.2	.	.
597	4898	0	25	167	57.0	20.4	.	.
598	4899	0	32	162	70.0	26.7	.	.
599	4900	1	24	170	48.0	16.6	.	.
600	4901	0	28	165	55.0	20.2	.	.
601	4902	0	21	159	48.0	19.0	.	.
602	4903	0	39	152	60.0	26.0	.	.
603	4904	1	30	169	62.0	21.7	.	.
604	4906	0	27	160	53.0	20.7	.	.
605	4909	0	43	163	60.0	22.6	.	.
606	4910	0	24	163	52.0	19.6	.	.
607	4911	0	23	156	46.0	18.9	.	.
608	4912	1	43	165	74.5	27.4	.	.
609	4913	0	32	162	52.0	19.8	.	.
610	4914	0	33	166	84.0	30.5	.	.
611	4915	0	40	169	70.0	24.5	.	.
612	4916	0	39	161	65.1	25.1	.	.
613	4917	0	25	162	60.0	22.9	.	.
614	4918	0	33	145	48.0	22.8	.	.
615	4919	0	37	156	54.7	22.5	.	.
616	4920	0	37	156	54.7	22.5	.	.
617	4921	0	26	155	60.0	25.0	.	.
618	4922	0	34	153	48.0	20.5	.	.
619	4923	0	40	157	61.3	24.9	.	.
620	4926	0	52	150	71.0	31.6	.	.
621	4927	0	20	159	45.0	17.8	.	.
622	4928	0	28	163	63.0	23.7	.	.
623	4929	0	42	164	83.0	30.9	.	.
624	4930	0	29	159	56.0	22.2	.	.
625	4931	0	20	164	52.0	19.3	.	.
626	4933	0	30	153	60.0	25.6	.	.
627	4934	0	34	164	62.0	23.1	.	.
628	4935	0	29	154	47.0	19.8	.	.
629	4936	0	32	160	55.0	21.5	.	.
630	4937	0	21	163	55.0	20.7	.	.
631	4938	0	30	164	60.5	22.5	.	.
632	4939	0	20	159	52.6	20.8	.	.
633	4940	0	22	166	59.0	21.4	.	.
634	4941	0	22	156	51.0	21.0	.	.
635	4942	0	36	164	69.5	25.8	.	.
636	4944	0	26	154	48.0	20.2	.	.
637	4945	0	28	157	49.1	19.9	.	.
638	4946	0	32	156	46.8	19.2	.	.
639	4947	0	26	161	53.5	20.6	.	.
640	4948	0	30	154	60.0	25.3	.	.
641	4949	0	23	160	56.0	21.9	.	.
642	4950	0	32	157	77.0	31.2	.	.
643	4951	0	20	157	55.0	22.3	.	.
644	4952	0	20	163	51.0	19.2	.	.
645	4954	0	28	168	60.0	21.3	.	.
646	4955	1	52	169	74.0	25.9	.	.
647	4956	0	20	153	73.0	31.2	.	.

648	4957	0	22	152	46.0	19.9	.	.
649	4958	0	30	158	45.7	18.3	.	.
650	4959	1	45	163	50.0	18.8	.	.
651	4960	1	39	175	85.0	27.8	.	.
652	4961	0	35	154	69.0	29.1	.	.
653	4962	0	20	150	47.0	20.9	.	.
654	4963	0	37	153	52.2	22.3	.	.
655	4964	0	37	153	52.2	22.3	.	.
656	4965	0	20	160	42.0	16.4	.	.
657	4967	0	25	166	35.0	12.7	.	.
659	4969	1	52	168	73.5	26.0	.	.
660	4970	0	25	154	46.0	19.4	.	.
661	4971	0	41	164	66.0	24.5	.	.
662	4972	1	37	164	54.0	20.1	.	.
663	4973	0	47	160	100.0	39.1	.	.
664	4974	0	54	166	78.3	28.4	.	.
665	4975	0	37	153	51.0	21.8	.	.
666	4976	0	44	159	77.6	30.7	.	.
667	4977	0	51	157	52.0	21.1	.	.
668	4978	0	42	163	53.0	19.9	.	.
669	4979	0	42	159	60.4	23.9	.	.
670	4980	1	20	175	80.0	26.1	.	.
671	4981	1	36	183	83.0	24.8	.	.
672	4982	0	25	165	53.0	19.5	.	.
673	4983	0	31	156	50.0	20.5	.	.
674	4984	0	68	155	58.0	24.1	.	.
675	4986	0	46	157	78.0	31.6	.	.
676	4987	0	24	165	50.0	18.4	.	.
677	4988	0	23	162	55.0	21.0	.	.
678	4989	0	34	150	67.0	29.8	.	.
679	4990	0	34	150	67.0	29.8	.	.
680	4991	0	59	156	60.0	24.7	.	.
681	4992	0	20	154	43.0	18.1	.	.
682	4993	0	36	153	44.5	19.0	.	.
683	4994	0	29	162	56.0	21.3	.	.
684	4995	0	28	155	50.9	21.2	.	.
685	4996	0	54	165	50.0	18.4	.	.
686	4997	0	20	170	50.0	17.3	.	.
687	4998	0	24	153	52.0	22.2	.	.
688	4999	0	35	156	74.0	30.4	.	.
689	5000	0	42	150	60.0	26.7	.	.
690	5001	0	48	160	64.0	25.0	.	.
691	5002	1	44	165	83.0	30.5	.	.
692	5005	0	21	168	54.0	19.1	.	.
693	5006	0	46	165	63.0	23.1	.	.
694	5009	1	55	176	70.5	22.8	.	.
695	5010	0	20	157	48.0	19.5	.	.
696	5011	0	29	163	54.0	20.3	.	.
697	5012	0	32	157	62.0	25.2	.	.
698	5013	0	20	154	51.0	21.5	.	.
699	5014	0	38	165	37.5	13.8	.	.
700	5015	0	24	153	54.0	23.1	.	.
701	5016	0	37	163	60.0	22.6	.	.
702	5017	0	25	158	54.0	21.6	.	.
703	5018	0	28	157	44.0	17.9	.	.
704	5019	0	22	171	67.0	22.9	.	.
705	5021	0	24	151	46.7	20.5	.	.
706	5022	0	28	161	70.0	27.0	.	.
707	5023	1	37	174	64.0	21.1	.	.

708	5024	0	42	164	68.0	25.3	.	.
709	5025	0	45	165	61.0	22.4	.	.
710	5026	0	29	163	41.0	15.4	.	.
711	5027	0	25	160	57.0	22.3	.	.
712	5028	1	20	177	65.0	20.7	.	.
713	5029	1	21	168	80.0	28.3	.	.
714	5030	0	23	166	57.0	20.7	.	.
715	5031	0	35	160	54.0	21.1	.	.
716	5033	0	24	157	52.0	21.1	.	.
717	5034	0	25	156	55.0	22.6	.	.
718	5036	0	27	166	54.0	19.6	.	.
719	5037	0	22	154	60.0	25.3	.	.
720	5038	0	25	151	45.0	19.7	.	.
721	5039	0	40	163	64.0	24.1	.	.
722	5040	0	45	151	51.0	22.4	.	.
723	5042	0	23	155	48.0	20.0	.	.
724	5043	0	43	153	53.0	22.6	.	.
725	5045	0	23	153	58.0	24.8	.	.
726	5046	1	23	165	53.0	19.5	.	.
727	5047	0	42	158	71.0	28.4	.	.
728	5048	0	30	170	60.0	20.8	.	.
729	5050	0	20	156	55.8	22.9	.	.
730	5053	1	29	173	64.0	21.4	.	.
731	5054	0	27	165	59.0	21.7	.	.
732	5055	0	28	164	63.6	23.6	.	.
733	5056	1	41	178	65.7	20.7	.	.
734	5057	0	37	157	51.5	20.9	.	.
735	5058	0	53	160	68.0	26.6	.	.
736	5059	1	20	170	72.0	24.9	.	.
737	5060	0	26	165	50.0	18.4	.	.
738	5062	0	20	162	54.0	20.6	.	.
739	5063	0	20	162	58.9	22.4	.	.
740	5064	0	45	164	55.0	20.4	.	.
741	5065	1	20	150	30.0	13.3	.	.
742	5066	0	37	157	60.0	24.3	.	.
743	5067	0	51	160	65.0	25.4	.	.
744	5068	0	23	165	75.3	27.7	.	.
745	5070	0	49	160	48.0	18.7	.	.
746	5071	0	25	161	51.5	19.9	.	.
747	5072	0	33	158	48.0	19.2	.	.
748	5073	0	20	162	59.0	22.5	.	.
749	5075	0	20	149	51.0	23.0	.	.
750	5077	0	37	155	59.0	24.6	.	.
751	5078	0	28	165	55.2	20.3	.	.
752	5079	0	36	157	66.1	26.8	.	.
753	5083	0	20	159	49.0	19.4	.	.
754	5084	0	30	157	70.0	28.4	.	.
755	5085	0	55	151	60.0	26.3	.	.
756	5086	0	47	152	45.0	19.5	.	.
757	5088	0	21	164	69.0	25.7	.	.
758	5089	0	50	157	63.0	25.6	.	.
759	5090	0	46	162	58.0	22.1	.	.
760	5091	0	35	159	65.0	25.7	.	.
761	5092	0	20	171	68.0	23.3	.	.
762	5093	0	22	156	49.0	20.1	.	.
763	5094	0	24	167	105.0	37.6	.	.
764	5095	0	25	168	58.0	20.5	.	.
765	5096	0	29	165	93.0	34.2	.	.
766	5097	0	27	158	47.0	18.8	.	.

767	5098	0	21	160	53.0	20.7	.	.
768	5099	0	30	161	65.0	25.1	.	.
769	5100	0	20	163	52.0	19.6	.	.
770	5101	0	20	163	52.0	19.6	.	.
771	5102	0	20	160	53.0	20.7	.	.
772	5103	0	21	163	75.0	28.2	.	.
773	5104	0	24	162	67.3	25.6	.	.
774	5105	0	54	146	68.0	31.9	.	.
775	5106	0	42	155	53.0	22.1	.	.
776	5108	0	22	166	53.0	19.2	.	.
777	5109	1	35	173	69.0	23.1	.	.
778	5110	1	37	184	89.0	26.3	.	.
779	5111	0	27	163	52.0	19.6	.	.
780	5112	0	27	147	61.3	28.4	.	.
781	5113	0	21	151	55.0	24.1	.	.
782	5114	0	25	152	53.0	22.9	.	.
783	5117	0	20	166	65.0	23.6	.	.
784	5118	0	32	157	61.0	24.7	.	.
785	5119	0	29	161	57.0	22.0	.	.
786	5120	0	28	150	48.0	21.3	.	.
787	5121	0	27	164	60.0	22.3	.	.
788	5122	0	37	168	60.2	21.3	.	.
789	5123	1	37	164	66.5	24.7	.	.
790	5124	0	20	164	52.0	19.3	.	.
791	5126	1	58	165	87.0	32.0	.	.
792	5127	1	60	169	69.8	24.4	.	.
793	5128	0	35	162	87.0	33.2	.	.
794	5130	0	45	156	58.4	24.0	.	.
795	5131	0	20	154	43.5	18.3	.	.
796	5132	0	23	156	56.9	23.4	.	.
797	5133	0	50	161	53.0	20.4	.	.
798	5134	0	43	159	60.0	23.7	.	.
799	5135	0	20	161	48.5	18.7	.	.
800	5136	0	43	159	61.0	24.1	.	.
801	5137	0	39	163	50.0	18.8	.	.
802	5138	0	22	158	49.0	19.6	.	.
803	5139	0	33	158	46.5	18.6	.	.
804	5140	0	28	161	50.0	19.3	.	.
805	5141	0	50	163	63.0	23.7	.	.
806	5142	0	37	155	54.0	22.5	.	.
807	5143	0	55	151	45.0	19.7	.	.
808	5144	0	38	167	63.0	22.6	.	.
809	5145	0	21	164	53.0	19.7	.	.
810	5146	0	20	164	57.0	21.2	.	.
811	5147	0	31	160	56.0	21.9	.	.
812	5148	0	24	150	63.5	28.2	.	.
813	5149	1	20	171	50.0	17.1	.	.
814	5150	0	23	149	44.0	19.8	.	.
815	5151	0	23	162	65.0	24.8	.	.
816	5152	0	22	156	68.5	28.1	.	.
817	5154	0	47	162	65.6	25.0	.	.
818	5156	0	23	153	120.0	51.3	.	.
819	5157	0	30	169	56.0	19.6	.	.
820	5158	0	29	153	56.0	23.9	.	.
821	5159	0	20	168	58.0	20.5	.	.
822	5160	0	36	156	56.0	23.0	.	.
823	5162	0	32	163	62.0	23.3	.	.
824	5163	0	26	154	94.0	39.6	.	.
825	5164	0	27	160	50.0	19.5	.	.

826	5165	1	44	174	67.0	22.1	.	.
827	5166	1	21	169	66.0	23.1	.	.
828	5167	0	58	160	59.5	23.2	.	.
829	5168	0	47	163	61.0	23.0	.	.
830	5169	0	45	164	69.0	25.7	.	.
831	5170	0	35	164	73.0	27.1	.	.
832	5171	0	33	161	53.0	20.4	.	.
833	5172	0	58	160	59.5	23.2	.	.
834	5174	1	71	174	87.0	28.7	.	.
835	5175	0	29	157	43.0	17.4	.	.
836	5176	0	29	160	65.0	25.4	.	.
837	5177	1	28	175	80.0	26.1	.	.
838	5178	1	20	170	80.0	27.7	.	.
839	5179	0	25	161	51.1	19.7	.	.
840	5180	0	26	154	48.0	20.2	.	.
841	5182	0	26	162	50.0	19.1	.	.
842	5183	0	53	157	62.0	25.2	.	.
843	5184	0	36	168	64.0	22.7	.	.
844	5185	0	46	157	57.0	23.1	.	.
845	5186	0	25	157	51.0	20.7	.	.
846	5187	0	20	159	58.6	23.2	.	.
847	5188	0	28	151	54.0	23.7	.	.
848	5189	0	27	153	70.0	29.9	.	.
849	5190	0	21	162	48.5	18.5	.	.
850	5191	0	25	158	58.0	23.2	.	.
851	5192	0	29	161	57.0	22.0	.	.
852	5194	0	34	162	54.0	20.6	.	.
853	5195	0	34	162	54.0	20.6	.	.
854	5196	0	22	143	59.0	28.9	.	.
855	5197	1	47	185	85.0	24.8	.	.
856	5199	0	24	162	54.0	20.6	.	.
857	5201	0	38	158	92.4	37.0	.	.
858	5202	0	29	162	55.0	21.0	.	.
859	5203	0	47	150	55.0	24.4	.	.
860	5204	0	44	165	54.0	19.8	.	.
861	5206	0	28	158	86.0	34.4	.	.
862	5207	0	30	153	74.4	31.8	.	.
863	5208	0	47	156	46.0	18.9	.	.
864	5209	0	28	151	52.0	22.8	.	.
865	5210	1	47	170	68.0	23.5	.	.
866	5211	0	24	159	70.0	27.7	.	.
867	5212	0	20	153	49.0	20.9	.	.
868	5213	0	27	155	65.0	27.1	.	.
869	5214	1	20	156	55.0	22.6	.	.
870	5215	0	25	148	44.0	20.1	.	.
871	5216	1	62	168	64.0	22.7	.	.
872	5217	0	31	156	64.4	26.5	.	.
873	5220	0	54	158	55.6	22.3	.	.
874	5222	0	25	159	57.8	22.9	.	.
875	5223	0	20	157	50.5	20.5	.	.
876	5224	0	20	158	55.0	22.0	.	.
877	5226	0	33	162	52.0	19.8	.	.
878	5227	0	45	156	48.3	19.8	.	.
879	5228	0	20	155	37.0	15.4	.	.
880	5230	0	38	165	60.0	22.0	.	.
881	5232	0	53	163	74.0	27.9	.	.
882	5234	0	35	163	67.0	25.2	.	.
883	5236	0	20	153	50.5	21.6	.	.
884	5237	0	45	158	57.0	22.8	.	.

885	5238	0	30	165	79.0	29.0	.	.
886	5240	0	22	164	55.0	20.4	.	.
887	5241	0	45	155	61.0	25.4	.	.
888	5242	0	20	172	56.0	18.9	.	.
889	5243	0	28	163	67.7	25.5	.	.
890	5244	0	55	155	59.0	24.6	.	.
891	5245	0	39	160	99.0	38.7	.	.
892	5246	0	43	158	75.0	30.0	.	.
893	5247	0	23	158	53.0	21.2	.	.
894	5248	0	46	169	52.0	18.2	.	.
895	5249	0	35	159	64.4	25.5	.	.
896	5250	0	22	161	57.0	22.0	.	.
897	5252	0	27	167	63.0	22.6	.	.
898	5253	0	36	157	57.0	23.1	.	.
899	5254	0	52	161	69.7	26.9	.	.
900	5255	1	40	178	74.0	23.4	.	.
901	5256	0	21	160	46.0	18.0	.	.
902	5257	0	48	158	62.5	25.0	.	.
903	5259	0	24	156	52.3	21.5	.	.
904	5260	0	38	163	54.0	20.3	.	.
905	5263	0	21	160	53.0	20.7	.	.
906	5264	1	45	173	92.0	30.7	.	.
907	5265	0	20	157	47.0	19.1	.	.
908	5266	1	37	172	62.0	21.0	.	.
909	5267	0	30	159	46.5	18.4	.	.
910	5268	1	33	172	63.0	21.3	.	.
911	5269	0	29	157	50.7	20.6	.	.
912	5271	0	39	153	73.0	31.2	.	.
913	5272	0	20	155	54.0	22.5	.	.
914	5273	0	21	157	52.0	21.1	.	.
915	5274	0	29	152	46.0	19.9	.	.
916	5275	0	21	154	60.0	25.3	.	.
917	5276	0	20	161	47.0	18.1	.	.
918	5277	0	47	148	58.1	26.5	.	.
919	5278	1	27	170	60.0	20.8	.	.
920	5279	0	20	160	53.8	21.0	.	.
921	5280	0	23	156	55.0	22.6	.	.
922	5281	0	45	153	63.0	26.9	.	.
923	5282	0	43	157	53.0	21.5	.	.
924	5284	0	20	151	59.2	26.0	.	.
925	5285	0	30	161	48.0	18.5	.	.
926	5287	0	31	166	62.0	22.5	.	.
927	5288	0	24	160	58.9	23.0	.	.
928	5289	0	23	162	60.0	22.9	.	.
929	5290	0	22	166	56.0	20.3	.	.
930	5291	0	22	166	56.0	20.3	.	.
931	5292	0	22	163	55.4	20.9	.	.
932	5293	0	36	166	96.0	34.8	.	.
933	5294	0	22	154	45.0	19.0	.	.
934	5295	0	39	147	45.0	20.8	.	.
935	5296	0	33	158	49.7	19.9	.	.
936	5297	0	29	157	57.0	23.1	.	.
937	5298	0	24	166	63.0	22.9	.	.
938	5300	0	20	161	57.0	22.0	.	.
939	5301	1	27	170	60.0	20.8	.	.
940	5302	0	20	159	52.0	20.6	.	.
941	5303	0	20	160	53.0	20.7	.	.
942	5304	0	34	171	53.0	18.1	.	.
943	5305	0	25	157	51.0	20.7	.	.

944	5306	0	26	160	50.0	19.5	.	.
945	5307	1	24	170	65.0	22.5	.	.
946	5308	0	56	154	67.0	28.3	.	.
947	5309	0	20	158	60.0	24.0	.	.
948	5310	1	20	175	80.0	26.1	.	.
949	5311	0	26	155	43.0	17.9	.	.
950	5312	0	51	157	50.0	20.3	.	.
951	5313	0	47	150	50.0	22.2	.	.
952	5314	0	20	162	62.8	23.9	.	.
953	5316	1	39	167	90.0	32.3	.	.
954	5317	0	25	152	57.0	24.7	.	.
955	5320	0	26	165	60.0	22.0	.	.
956	5321	0	26	163	49.0	18.4	.	.
957	5322	0	26	160	47.5	18.6	.	.
958	5323	0	23	169	53.0	18.6	.	.
959	5324	0	42	160	56.0	21.9	.	.
960	5325	0	24	156	85.0	34.9	.	.
961	5326	0	23	161	57.0	22.0	.	.
962	5327	0	37	152	46.0	19.9	.	.
963	5329	0	43	158	55.0	22.0	.	.
964	5330	0	22	163	75.0	28.2	.	.
965	5331	0	38	156	59.5	24.4	.	.
966	5332	0	37	168	56.0	19.8	.	.
967	5333	0	38	157	47.0	19.1	.	.
968	5334	0	23	162	55.0	21.0	.	.
969	5335	0	41	160	60.0	23.4	.	.
970	5336	0	20	157	53.0	21.5	.	.
971	5337	0	20	157	48.0	19.5	.	.
972	5339	0	21	156	48.0	19.7	.	.
973	5340	0	26	163	50.0	18.8	.	.
974	5341	1	44	172	70.0	23.7	.	.
975	5342	0	20	170	55.4	19.2	.	.
976	5343	0	20	165	68.0	25.0	.	.
977	5344	0	32	165	62.0	22.8	.	.
978	5345	0	22	163	52.0	19.6	.	.
979	5346	0	22	163	70.0	26.3	.	.
980	5347	0	32	157	60.7	24.6	.	.
981	5348	0	28	169	56.3	19.7	.	.
982	5349	0	34	155	66.0	27.5	.	.
983	5351	0	53	163	54.0	20.3	.	.
984	5352	0	35	161	64.0	24.7	.	.
985	5354	1	27	167	61.0	21.9	.	.
986	5355	0	39	159	49.0	19.4	.	.
987	5356	0	56	150	55.0	24.4	.	.
988	5357	0	41	171	62.9	21.5	.	.
989	5358	0	23	156	51.0	21.0	.	.
990	5359	0	26	155	59.0	24.6	.	.
991	5360	0	25	160	56.9	22.2	.	.
992	5361	1	20	170	80.0	27.7	.	.
993	5362	0	31	160	58.0	22.7	.	.
994	5363	0	27	162	61.0	23.2	.	.
995	5364	0	20	158	59.0	23.6	.	.
996	5366	0	55	150	55.0	24.4	.	.
997	5367	0	55	150	55.0	24.4	.	.
998	5368	0	22	160	41.0	16.0	.	.
999	5369	0	40	166	68.0	24.7	.	.
1000	5370	0	20	159	54.0	21.4	.	.
1001	5371	0	29	148	45.8	20.9	.	.
1002	5372	0	23	158	50.8	20.3	.	.

1003	5373	0	32	152	55.0	23.8	.	.
1004	5375	0	25	169	75.0	26.3	.	.
1005	5377	0	21	160	58.3	22.8	.	.
1006	5378	1	52	170	82.0	28.4	.	.
1007	5379	0	20	162	60.7	23.1	.	.
1008	5380	0	25	150	42.0	18.7	.	.
1009	5382	1	33	175	76.0	24.8	.	.
1010	5383	0	20	155	53.3	22.2	.	.
1011	5384	0	27	160	56.9	22.2	.	.
1012	5385	0	30	156	62.0	25.5	.	.
1013	5386	0	46	155	52.0	21.6	.	.
1014	5389	1	51	175	78.0	25.5	.	.
1015	5390	0	22	165	57.0	20.9	.	.
1016	5391	0	29	162	54.7	20.8	.	.
1017	5392	0	35	160	64.0	25.0	.	.
1018	5395	0	48	163	50.0	18.8	.	.
1019	5396	0	35	166	59.0	21.4	.	.
1020	5398	0	29	157	64.0	26.0	.	.
1021	5399	0	31	165	65.0	23.9	.	.
1022	5400	0	26	158	67.6	27.1	.	.
1023	5401	0	38	168	55.0	19.5	.	.
1024	5402	0	21	161	61.0	23.5	.	.
1025	5403	1	45	171	81.0	27.7	.	.
1026	5404	0	27	157	62.5	25.4	.	.
1027	5405	0	46	165	62.0	22.8	.	.
1028	5407	0	55	150	55.0	24.4	.	.
1029	5408	0	20	168	53.0	18.8	.	.
1030	5409	0	23	151	46.0	20.2	.	.
1031	5410	0	28	153	47.0	20.1	.	.
1032	5411	0	23	148	46.0	21.0	.	.
1033	5413	0	25	153	49.0	20.9	.	.
1034	5414	0	26	160	66.6	26.0	.	.
1035	5415	1	47	179	98.0	30.6	.	.
1036	5416	0	26	168	58.0	20.5	.	.
1037	5418	0	23	158	48.0	19.2	.	.
1038	5419	0	21	163	63.0	23.7	.	.
1039	5420	0	23	160	55.0	21.5	.	.
1040	5421	0	42	162	48.0	18.3	.	.
1041	5422	0	20	163	50.9	19.2	.	.
1042	5423	1	38	170	78.0	27.0	.	.
1043	5424	0	20	135	28.0	15.4	.	.
1044	5426	1	38	176	76.0	24.5	.	.
1045	5427	1	48	161	73.0	28.2	.	.
1046	5428	0	23	168	66.0	23.4	.	.
1047	5429	0	20	160	50.5	19.7	.	.
1048	5430	0	20	155	52.0	21.6	.	.
1049	5431	0	53	157	59.0	23.9	.	.
1050	5432	1	23	181	75.0	22.9	.	.
1051	5433	0	23	167	60.0	21.5	.	.
1052	5434	0	27	155	43.0	17.9	.	.
1053	5435	0	20	156	53.0	21.8	.	.
1054	5436	0	30	160	75.0	29.3	.	.
1055	5437	0	25	149	62.0	27.9	.	.
1056	5438	0	24	154	52.0	21.9	.	.
1057	5439	0	30	160	63.0	24.6	.	.
1058	5440	0	20	152	52.0	22.5	.	.
1059	5441	0	22	160	58.4	22.8	.	.
1060	5442	0	25	160	63.0	24.6	.	.
1061	5444	0	20	156	51.0	21.0	.	.

1062	5446	0	20	161	49.0	18.9	.	.
1063	5447	0	29	162	54.0	20.6	.	.
1064	5448	0	39	162	63.0	24.0	.	.
1065	5449	0	24	150	46.0	20.4	.	.
1066	5450	0	27	155	57.0	23.7	.	.
1067	5451	0	37	167	46.0	16.5	.	.
1068	5452	0	26	165	52.0	19.1	.	.
1069	5453	1	43	165	76.0	27.9	.	.
1070	5454	0	33	160	63.0	24.6	.	.
1071	5455	0	20	161	56.0	21.6	.	.
1072	5456	0	30	157	73.0	29.6	.	.
1073	5457	0	42	156	76.4	31.4	.	.
1074	5458	0	20	160	51.0	19.9	.	.
1075	5460	0	29	151	43.3	19.0	.	.
1076	5461	0	20	163	60.0	22.6	.	.
1077	5462	0	22	160	41.0	16.0	.	.
1078	5463	0	20	154	60.0	25.3	.	.
1079	5466	0	42	156	57.0	23.4	.	.
1080	5467	0	42	150	60.6	26.9	.	.
1081	5469	0	56	163	65.0	24.5	.	.
1082	5470	0	39	160	57.0	22.3	.	.
1083	5471	0	25	153	48.5	20.7	.	.
1084	5473	0	24	161	49.7	19.2	.	.
1085	5475	0	28	158	50.0	20.0	.	.
1086	5477	0	20	168	64.0	22.7	.	.

wake_up_2019_4	wake_up_2019_5	wake_up_2019_6	wake_up_2019_7	wake_up_2019_8	wake_up_2019_9
.	.	.	.	.	.
.	.	.	373	358	360
.	.	.	.	.	.
.	.	.	.	.	.
.	.	.	.	.	.
.	.	.	.	.	.
.	.	.	.	.	.
.	.	.	.	.	.
.	.	.	.	.	.
.	.	.	.	.	.
370	330	325	326	.	.
.	.	.	.	.	.
.	.	.	.	.	.
.	.	.	.	.	.
.	.	.	.	.	.
.	.	.	.	.	.
.	.	.	.	.	.
.	.	.	.	.	.
.	.	.	.	.	.
.	.	.	.	.	.
.	.	.	.	.	.
.	.	.	.	.	.
.	.	.	.	.	.
.	.	.	.	.	.
.	.	.	.	.	.
.	.	.	.	.	.
.	.	.	.	.	.
.	.	.	.	.	.
.	.	.	.	.	.
.	.	.	.	.	.
.	.	.	.	.	.
.	.	.	.	.	.
.	.	.	.	.	.
.	.	.	.	.	.
.	.	.	.	.	.
.	.	.	.	.	.
.	.	.	.	.	.
.	.	.	.	.	.
.	.	.	.	.	.
.	.	.	.	.	.
422	433	446	378	442	445
.	.	.	.	.	.
.	.	.	.	.	.
.	.	.	.	.	.
540	.	.	.	.	.
.	.	.	.	.	.
.	.	.	.	.	.
.	.	.	.	.	.
.	.	.	.	.	.
450	.	.	.	.	430
.	.	.	.	.	.
.	.	.	.	.	.
.	.	.	.	.	.
.	.	.	.	.	.
.	.	.	.	.	.
.	.	.	.	.	.
516	.	.	.	.	.
.	.	.	.	.	.

455
474
422
438 442 439 453 448 417
390
526
313 322 341 317 341 352
426 436 395 400 407 465
333 529 450

260					
445		401	456	382	
507	516	481	509	505	536
305					
487	563	393	383		
				540	
412	403	436	403	408	402
421				330	
431					
	391	386	355	449	395

.	.	.	.	.	.
370	.	.	.	.	.
.	.	.	.	.	.
.	.	.	.	.	.
.	.	.	.	.	.
.	.	.	.	.	.
.	.	.	.	.	.
403	.	.	383	.	.
.	.	.	.	.	.
.	.	.	.	.	.
.	.	.	.	.	.
437	458	432	.	.	.
.	.	.	.	.	.
.	.	.	.	.	.
.	.	.	.	.	.
.	.	.	.	.	.
.	.	.	.	.	.
406	457	472	455	448	422
.	.	.	.	.	.
.	.	.	.	.	.
.	.	.	.	.	.
.	.	.	.	.	.
370	.	.	.	.	.
380	.	.	.	.	.
.	.	.	.	.	.
315	.	.	.	.	.
525	.	.	.	.	.
710	.	.	.	.	.
525	.	.	.	.	.
335	.	.	.	.	.
405	.	.	.	.	.
150	.	.	.	.	.
410	.	.	.	.	.
434	494	.	.	.	.
420	.	.	.	.	.
450	.	.	.	.	.
330	.	.	.	.	.
.	.	.	.	.	.
633	.	.	.	.	.
331	.	.	.	.	.
.	.	.	.	.	.
.	.	.	.	.	.
278	.	.	.	.	.
.	.	.	.	.	.
.	.	.	.	.	.
677	.	.	.	.	.
.	.	.	.	.	.
.	.	.	.	.	.
.	329	.	.	.	.
.	.	.	.	.	.
.	412	387	440	.	.
.	390	.	.	.	.
.	300	409	486	.	.
.	.	.	600	.	.
.	430	.	.	.	.
.	568	455	447	.	477
.	.	.	.	.	.
.	465	426	473	414	438
.	420	.	.	.	.

.	.	.	.	.	.
.	.	420	.	.	.
.	.	412	403	371	390
.	.	.	.	.	.
.	.	.	.	.	.
.	.	.	.	.	.
.	.	373	407	.	.
.	.	.	.	.	.
.	.	420	.	.	.
.	.	420	.	.	.
.	.	445	.	.	.
.	.	448	.	.	.
.	.	.	469	.	.
.	.	510	500	.	.
.	.	.	.	.	.
.	.	.	268	320	299
.	.	.	.	.	.
.	.	.	528	.	.
.	.	.	453	.	.
.	.	.	563	517	480
.	.	.	360	.	.
.	.	.	.	.	.
.	.	.	660	.	.
.	.	.	.	.	.
.	.	.	520	.	.
.	.	.	506	351	.
.	.	.	390	.	.
.	.	.	420	.	.
.	.	.	330	.	.
.	.	.	.	406	410
.	.	.	.	413	.
.	.	.	.	470	.
.	.	.	.	.	.
.	.	.	.	399	380
.	.	.	.	.	.
.	.	.	.	.	.
.	.	.	.	480	.
.	.	.	.	465	.
.	.	.	.	.	.
.	.	.	.	.	.
.	.	.	.	.	.
.	.	.	.	.	.
.	.	.	.	.	.
.	.	.	.	.	.
.	.	.	.	499	.
.	.	.	.	480	.
.	.	.	.	362	.
.	.	.	.	404	.
.	.	.	.	565	.
.	.	.	.	.	487
.	.	.	.	.	270
.	.	.	.	.	406
.	.	.	.	.	.
.	.	.	.	.	440
.	.	.	.	.	.
.	.	.	.	.	.
.	.	.	.	.	470
.	.	.	.	.	392
.	.	.	.	.	531

.	.	.	.	.	.	
.	.	.	.	.		395
.	.	.	.	.		338
.	.	.	.	.		415
.	.	.	.	.		600
.	.	.	.	.		530
.	.	.	.	.		360
.	.	.	.	.		270
.	.	.	.	.	.	
.	.	.	.	.		464
.	.	.	.	.	.	
.	.	.	.	.		465
.	.	.	.	.		390
.	.	.	.	.		465
.	.	.	.	.		420
.	.	.	.	.		450
.	.	.	.	.	.	
.	.	.	.	.	.	
.	.	.	.	.	.	
.	.	.	.	.	.	
.	.	.	.	.	.	
.	.	.	.	.	.	
.	.	.	.	.	.	
.	.	.	.	.	.	
.	.	.	.	.	.	
.	.	.	.	.	.	
.	.	.	.	.	.	
.	.	.	.	.	.	
.	.	.	.	.	.	
.	.	.	.	.	.	
.	.	.	.	.	.	
.	.	.	.	.	.	
.	.	.	.	.	.	
.	.	.	.	.	.	
.	.	.	.	.	.	
.	.	.	.	.	.	
.	.	.	.	.	.	
.	.	.	.	.	.	
.	.	.	.	.	.	
.	.	.	.	.	.	
.	.	.	.	.	.	
.	.	.	.	.	.	
.	.	.	.	.	.	
.	.	.	.	.	.	
.	.	.	.	.	.	
.	.	.	.	.	.	
.	.	.	.	.	.	
.	.	.	.	.	.	
.	.	.	.	.	.	
.	.	.	.	.	.	
.	.	.	.	.	.	
.	.	.	.	.	.	
.	.	.	.	.	.	
.	.	.	.	.	.	
.	.	.	.	.	.	
.	.	.	.	.	.	
.	.	.	.	.	.	
.	.	.	.	.	.	
.	.	.	.	.	.	

wake_up_2019_10	wake_up_2019_11	wake_up_2019_12	wake_up_2019_13	wake_up_2019_14
.	.	.	.	.
349	366	.	.	270
.	.	.	.	.
.	.	.	.	.
.	.	.	.	.
.	.	.	.	.
.	.	.	.	.
.	.	.	.	.
.	.	.	.	.
.	.	.	.	.
.	.	.	.	.
.	.	.	.	.
.	.	.	.	.
.	.	.	.	.
.	.	.	.	.
.	.	.	.	.
.	.	.	.	.
.	.	.	.	.
.	.	.	.	.
.	.	.	.	.
.	.	.	.	.
.	.	.	.	.
.	.	.	.	.
.	.	.	.	.
.	.	.	.	.
.	.	.	.	.
.	.	.	.	.
.	.	.	.	.
.	.	.	.	.
.	.	.	.	.
.	.	.	.	.
.	.	.	.	.
.	.	.	.	.
.	.	.	.	.
.	.	.	.	.
.	.	.	.	.
.	.	.	.	.
.	.	.	.	.
.	.	.	.	.
.	.	.	.	.
370	400	443	376	443
.	.	.	.	.
.	.	.	.	.
.	.	.	.	.
.	.	420	423	418
.	.	.	.	.
.	.	.	.	.
.	.	.	.	.
.	.	.	.	.
435	360	.	.	.
.	.	.	.	.
.	.	.	.	.
.	.	.	.	.
.	.	.	.	.
.	.	.	.	.
.	.	.	.	.
.	.	.	.	.
.	.	.	.	.

391	409	399	396	408
410				
347	345	342	327	374
			510	
401	429	399		

430

420 450 373 336

501 470 463 485 529

518

480

393 401 398 415 403

386 433 390 422

458

241

394	410	408	388	412
281	272	230		
510		503	450	
390	418	479	391	
440				
510	485	486		
481	459	460	435	471
374	384	370	367	400

.	.	.	.	.
.	.	.	.	.
401	305	.	.	.
.	420	.	.	.
.	.	.	.	.
.	.	.	.	.
.	.	.	.	.
300	273	273	293	293
.	.	.	.	.
.	.	.	.	.
320	279	315	330	291
490	.	.	.	.
.	.	.	.	.
.	.	.	.	.
.	.	.	.	.
550	.	.	.	.
420	.	.	.	.
555	430	399	427	.
400	.	.	.	.
.	.	.	.	.
500	.	.	.	.
600	.	.	.	.
380	.	.	.	.
.	530	.	.	.
.	.	.	.	.
350	.	.	.	.
395	.	.	.	.
.	.	.	.	.
300	324	.	.	.
390	.	.	.	.
.	.	.	.	.
.	.	.	.	.
.	440	.	.	.
.	.	.	.	.
.	440	.	.	.
.	.	.	.	.
.	421	.	.	.
.	450	.	.	.
.	.	.	.	.
.	317	285	293	304
.	.	.	.	.
.	420	.	.	.
.	430	.	.	.
.	.	.	.	.
.	.	.	.	.
.	.	.	.	.
.	512	.	.	.
.	420	360	.	.
.	390	.	.	.
.	.	.	.	.
.	.	370	.	.
.	.	.	.	.
.	.	600	.	.
.	.	333	.	.
.	.	302	.	368
.	.	390	.	.
.	.	.	.	.
.	.	.	.	.
.	.	331	.	.

.	.	.		.		.	
.	.	.		.		.	
.	.	.		.		.	
.	.		570	.		.	
.	.		407		274	.	
.	.		420	.		.	
.	.	.		.		.	
.	.		420	.			540
.	.		390	.		.	
.	.	.		.		.	
.	.		390	.		.	
.	.	.		.		.	
.	.	.		.		.	
.	.	.		.		.	
.	.	.		.		.	
.	.	.		.		.	
.	.	.		.		.	
.	.		535	.		.	
.	.	.		.		.	
.	.	.		.		.	
.	.	.			394		415
.	.	.		.		.	
.	.	.			440	.	
.	.	.			450		419
.	.	.			404	.	
.	.	.			506	.	
.	.	.			481	.	
.	.	.		.			390
.	.	.		.		.	
.	.	.			420		375
.	.	.		.			434
.	.	.			365		380
.	.	.		.			424
.	.	.		.		.	
.	.	.			415	.	
.	.	.		.		.	
.	.	.		.		.	
.	.	.			420		422
.	.	.		.		.	
.	.	.		.			316
.	.	.		.		.	
.	.	.		.		.	
.	.	.		.		.	
.	.	.		.			409
.	.	.		.			325
.	.	.		.		.	
.	.	.		.			472
.	.	.		.			260
.	.	.		.		.	
.	.	.		.		.	
.	.	.		.			493
.	.	.		.			600
.	.	.		.		.	
.	.	.		.			498
.	.	.		.		.	
.	.	.		.			454
.	.	.		.		.	
.	.	.		.			511
.	.	.		.		.	

wake_up_2019_15	wake_up_2019_16	wake_up_2019_17	wake_up_2020_2	wake_up_2020_3
.	.	.	.	.
390	270	350	.	.
.	.	.	.	.
.	.	.	.	.
.	.	.	.	.
.	.	.	.	.
.	.	.	.	.
.	.	.	.	.
.	.	.	.	.
.	.	.	.	.
.	.	.	.	.
390	.	.	.	.
.	.	.	.	.
.	.	.	.	.
.	.	.	.	.
.	.	.	.	.
.	.	.	.	.
.	.	.	.	.
.	.	.	.	.
.	.	.	.	.
.	.	.	.	.
.	.	.	.	.
.	.	.	.	.
.	.	.	.	.
.	.	.	.	.
.	.	.	.	.
.	.	.	.	.
.	.	.	.	.
.	.	.	.	.
.	.	.	.	.
.	.	.	.	.
.	.	.	.	.
.	.	.	.	.
.	.	.	.	.
.	.	.	.	.
.	.	.	.	.
.	.	.	.	.
.	.	.	.	.
.	.	.	.	.
.	.	.	.	.
401	440	410	.	.
.	354	.	.	.
.	.	.	.	.
.	.	.	.	.
429	385	365	.	.
.	.	.	.	.
.	.	.	.	.
.	.	.	.	.
.	.	.	.	.
.	.	.	.	.
.	.	.	.	.
.	.	.	.	.
.	.	.	.	.
.	.	.	.	.
.	.	.	.	.
.	.	.	.	.
.	.	.	.	.
.	.	.	.	.

399	387	384		
			260	421
362	377	368		

399	491	394<br>447		
392				
401	393	401	404	405
447	430	497		

381	414	389	
	390		
445	463	424	
416	404	381	

360

564

572

	540	550
372	419	364
331	317	307
376		

.		.		.		.	.
	370	.		.		.	.
	406	.		.		.	.
	502		483		480	.	.
.		.		.		.	.
.		.		.		.	.
.		.		.		.	.
	350	.		.		.	.
	450	.		.		.	.
.		.		.		.	.
	510		504		510	.	.
	345	.		.		.	.
.		.		.		.	.
	420	.		.		.	.
	360	.		.		.	.
	492	.		.		.	.
.		.		.		.	.
.		.		.		.	.
.		.		.		.	.
.			387		382	.	.
.			300	.		.	.
.			474	.		.	.
.			451	.		.	.
.			360	.		.	.
.		.		.		.	.
.		.		.		.	.
.			313	.		.	.
.		.		.		.	.
.			420	.		.	.
.		.		.		.	.
.			470	.		.	.
.		.		.		.	.
.			420	.		.	.
.			388	.		.	.
.			420	.		.	.
.			440	.		.	.
.		.		.		.	.
.		.			420	.	.
.			400	.		.	.
.		.		.		.	.
.		.			367	.	.
.		.		.		.	.
.		.		.		.	.
.		.		.		.	.
.		.			511	.	.
.		.			460	.	.
.		.		.		.	.
.		.		.		.	.
.		.		.		.	.
.		.		.		.	.
.		.		.		.	.
.		.		.		.	.
.		.		.		.	.
.		.		.		.	.
.		.		.		.	.
.		.		.		.	.
.		.		.		.	.
.		.		.		.	.
.		.		.		.	.

.	.	.	.	.
.	.	.	.	.
.	.	.	429	434
.	.	.	.	.
.	.	.	.	.
.	.	.	.	478
.	.	.	430	.
.	.	.	.	.
.	.	.	.	.
.	.	.	.	.
.	.	.	.	.
.	.	.	.	.
.	.	.	405	.
.	.	.	.	.
.	.	.	.	.
.	.	.	.	.
.	.	.	.	.
.	.	.	.	.
.	.	.	.	.
.	.	.	.	.
.	.	.	.	.
.	.	.	.	.
.	.	.	.	.
.	.	.	.	.
.	.	.	.	.
.	.	.	.	.
.	.	.	.	.
.	.	.	.	.
.	.	.	417	415
.	.	.	.	.
.	.	.	.	.
.	.	.	.	.
.	.	.	431	421
.	.	.	.	.
.	.	.	.	.
.	.	.	515	502
.	.	.	472	.
.	.	.	.	.
.	.	.	.	.
.	.	.	447	463
.	.	.	559	532
.	.	.	415	429
.	.	.	446	448
.	.	.	442	445
.	.	.	.	.
.	.	.	.	.
.	.	.	.	.
.	.	.	373	.
.	.	.	.	.
.	.	.	388	.
.	.	.	.	.
.	.	.	438	.
.	.	.	.	.
.	.	.	.	.
.	.	.	.	.
.	.	.	356	.
.	.	.	388	.
.	.	.	435	.

.	.	.		400	.	
.	.	.		510	.	
.	.	.	.		.	
.	.	.		391	.	
.	.	.	.		.	
.	.	.		578	.	
.	.	.		425	.	
.	.	.		570	.	
.	.	.		439	.	
.	.	.	.		.	
.	.	.	.		.	
.	.	.		450	.	
.	.	.		536	.	
.	.	.		448	.	
.	.	.	.		.	
.	.	.	.		.	
.	.	.	.		.	
.	.	.		461		442
.	.	.		405		373
.	.	.		438		418
.	.	.	.		.	
.	.	.		345	.	
.	.	.		477	.	
.	.	.	.		.	
.	.	.	.		.	
.	.	.	.		.	
.	.	.	.		.	
.	.	.		495	.	
.	.	.		338	.	
.	.	.	.		.	
.	.	.		365	.	
.	.	.		351		337
.	.	.		570	.	
.	.	.	.		.	
.	.	.	.		.	
.	.	.		565	.	
.	.	.	.		.	
.	.	.	.		.	
.	.	.		530		608
.	.	.	.		.	
.	.	.	.			646
.	.	.	.			420
.	.	.	.		.	
.	.	.	.			540
.	.	.	.			360
.	.	.	.		.	
.	.	.	.			360
.	.	.	.		.	
.	.	.	.		.	
.	.	.	.			367
.	.	.	.		.	
.	.	.		432	.	
.	.	.	.		.	
.	.	.	.		.	
.	.	.	.			330
.	.	.	.			427
.	.	.	.			428
.	.	.	.		.	
.	.	.	.		.	

.	.	.	.		452
.	.	.	.	.	
.	.	.	.	.	
.	.	.	.		510
.	.	.	.		525
.	.	.	.		398
.	.	.	.		515
.	.	.	.		450
.	.	.	.		480
.	.	.	.		480
.	.	.	.		480
.	.	.	.	.	
.	.	.	.		300
.	.	.	.	.	
.	.	.	.	.	
.	.	.	.		510
.	.	.	.	.	
.	.	.	.	.	
.	.	.	.	.	
.	.	.	.		370
.	.	.	.		350
.	.	.	.		469
.	.	.	.	.	
.	.	.	.	.	
.	.	.	.	.	
.	.	.	.	.	
.	.	.	.	.	
.	.	.	.	.	
.	.	.	.	.	
.	.	.	.	.	
.	.	.	.	.	
.	.	.	.	.	
.	.	.	.	.	
.	.	.	.	.	
.	.	.	.	.	
.	.	.	.	.	
.	.	.	.	.	
.	.	.	.	.	
.	.	.	.	.	
.	.	.	.	.	
.	.	.	.	.	
.	.	.	.	.	
.	.	.	.	.	
.	.	.	.	.	
.	.	.	.	.	
.	.	.	.	.	
.	.	.	.	.	
.	.	.	.	.	
.	.	.	.	.	
.	.	.	.	.	
.	.	.	.	.	
.	.	.	.	.	
.	.	.	.	.	
.	.	.	.	.	
.	.	.	.	.	
.	.	.	.	.	
.	.	.	.	.	
.	.	.	.	.	
.	.	.	.	.	

wake_up_2020_4	wake_up_2020_5	wake_up_2020_6	wake_up_2020_7	wake_up_2020_8	wake_up_2020_9
.	.	.	.	.	.
.	.	.	.	.	.
.	.	.	.	.	.
.	.	.	.	.	.
.	.	.	.	.	.
.	.	.	.	.	.
.	.	.	.	.	.
.	.	.	.	.	.
.	.	.	.	.	.
.	.	.	.	.	.
.	.	.	.	.	.
.	.	.	.	.	.
.	.	.	.	.	.
.	.	.	.	.	.
.	.	.	.	.	.
.	.	.	.	.	.
.	.	.	.	.	.
.	.	.	.	.	.
.	.	.	.	.	.
.	.	.	.	.	.
.	.	.	.	.	.
.	.	.	.	.	.
.	.	.	.	.	.
.	.	.	.	.	.
.	.	.	.	.	.
.	.	.	.	.	.
.	.	.	.	.	.
.	.	.	.	.	.
.	.	.	.	.	.
.	.	.	.	.	.
.	.	.	.	.	.
.	.	.	.	.	.
.	.	.	.	.	.
.	.	.	.	.	.
.	.	.	.	.	.
.	.	.	.	.	.
.	.	.	.	.	.
.	.	.	.	.	.
.	.	.	.	.	.
.	.	.	.	.	.
.	.	.	.	.	.
.	.	.	.	.	.
.	.	.	.	.	.
.	.	.	.	.	.
.	.	.	.	.	.
.	.	.	.	.	.
.	.	.	.	.	.
.	.	.	.	.	.
.	.	.	.	.	.
.	.	.	.	.	.
.	.	.	.	.	.
.	.	.	.	.	.
.	.	.	.	.	.
.	.	.	.	.	.
.	.	.	.	.	.
.	.	.	.	.	.
.	.	.	.	.	.
.	.	.	.	.	.

550

401	423	404	387	414	405

449		379	410	345	
					440
					413
398	411	418	406	407	410
420	403				
517	385				
360			450		
383	391	472	494	515	477
540					
403	410	399	374	384	370
		471			
343					
	550	281			

540

380

400

.	.	.	.	.	.
.	.	.	.	.	.
.	.	.	.	.	.
.	.	.	.	.	.
.	.	.	.	.	.
.	.	.	.	.	.
.	.	.	.	.	.
.	.	.	.	.	.
.	.	.	.	.	.
.	.	.	.	.	.
.	.	.	.	.	.
.	.	.	.	.	.
.	.	.	.	.	.
.	.	.	.	.	.
.	.	.	.	.	.
.	.	.	.	.	.
.	.	.	.	.	.
582	.	.	.	.	.
.	.	.	.	.	.
.	.	.	.	.	.
367	392	.	.	.	.
416	404	344	.	.	.
.	.	.	.	.	.
393	.	.	.	.	.
.	.	.	.	.	.
484	498	.	.	.	.
350	.	.	.	.	.
323	.	.	.	.	.
.	.	.	.	.	.
412	.	.	.	.	.
410	.	.	.	.	.
382	.	.	.	.	.
405	.	.	.	.	.
406	.	.	.	.	.
.	.	.	.	.	.
426	.	.	.	.	.
430	391	.	.	.	.
405	.	.	.	.	.
419	425	.	.	.	.
590	.	.	.	.	.
.	.	.	.	.	.
.	.	.	.	.	.
575	.	.	.	.	.
500	.	.	.	.	.
385	296	351	.	.	.
.	.	.	.	.	.
.	.	.	.	.	.
360	.	.	.	.	.
460	381	.	.	.	.
443	435	.	.	.	.
470	475	.	.	.	.
.	.	.	.	.	.
.	.	.	.	.	.
375	386	410	422	368	.
.	.	.	.	.	.
.	.	.	.	.	.
.	570	.	.	.	.
.	.	.	.	.	.
.	.	.	.	.	.

.	.	.	.	.	.
.	.	.	.	.	.
.	447	398	.	420	421
.	424	440	.	.	.
.	270	.	.	.	.
.	.	.	.	.	.
.	.	.	.	.	.
.	420	.	.	.	.
.	334	.	.	.	.
.	391	.	.	.	.
.	363	339	.	.	.
.	.	.	.	.	.
.	360	.	.	.	.
.	420	.	.	.	.
.	.	.	.	.	.
.	326	.	.	.	.
.	325	.	.	.	.
.	437	420	.	.	.
.	380	.	.	.	.
.	436	449	437	419	427
.	.	420	.	.	.
.	.	.	.	.	.
.	.	.	.	.	.
.	484	.	.	.	.
.	465	.	.	.	.
.	375	420	.	.	.
.	.	.	.	.	.
.	.	.	.	.	.
.	.	.	.	.	.
.	.	.	.	.	.
.	.	.	.	.	.
.	535	.	.	.	.
.	.	.	.	.	.
.	.	.	.	.	.
.	570	.	.	.	.
.	.	.	.	.	.
.	.	.	.	.	.
.	.	640	.	.	.
.	.	.	.	.	.
.	.	.	.	.	.
.	.	383	.	.	.
.	.	300	.	.	.
.	.	.	.	.	.
.	.	.	.	.	.
.	.	.	.	.	.
.	.	.	.	.	.
.	.	600	.	.	.
.	.	443	.	.	.
.	.	352	315	315	.
.	.	480	.	.	.
.	.	.	.	.	.
.	.	.	.	.	.
.	.	.	.	.	.
.	.	.	.	.	.
.	.	.	.	.	.
.	.	645	.	.	.
.	.	474	.	.	.
.	.	.	.	.	.
.	.	.	.	.	.

.	.	.	.	.	.	
.	.		462 .	.	.	
.	.		568	604	626 .	
.	.	.	.	.	.	
.	.	.	.	.	.	
.	.		660 .	.	.	
.	.		463	390 .	.	
.	.		447 .	.	.	
.	.	.	.	.	.	
.	.	.	.	.	.	
.	.	.	.	.	.	
.	.		480 .	.	.	
.	.	.	.	.	.	
.	.		450	450 .	.	
.	.		420 .	.	.	
.	.	.	.	.	.	
.	.	.	.	.	.	
.	.	.		364 .	.	
.	.	.	.	.	.	
.	.	.		341	387	372
.	.	.		390 .	.	
.	.	.		495	510 .	
.	.	.	.	.	.	
.	.	.	.	.	.	
.	.	.	.	.	.	
.	.	.		375 .	.	
.	.	.		440 .	.	
.	.	.		380 .	.	
.	.	.	.	.	.	
.	.	.		302 .	.	
.	.	.	.	.	.	
.	.	.		570 .	.	
.	.	.		390 .	.	
.	.	.	.	.	.	
.	.	.	.	.	.	
.	.	.		420 .	.	
.	.	.		630	564	558
.	.	.	.	.	.	
.	.	.	.	.	.	
.	.	.	.		409 .	
.	.	.	.		321 .	
.	.	.	.		540 .	
.	.	.	.		377 .	
.	.	.	.		420 .	
.	.	.	.		535 .	
.	.	.	.		432 .	
.	.	.	.		270	282
.	.	.	.		471 .	
.	.	.	.		443	393
.	.	.	.	.	.	
.	.	.	.		388	383
.	.	.	.		420 .	
.	.	.	.		490	497
.	.	.	.		390 .	
.	.	.	.		435 .	
.	.	.	.	.		579
.	.	.	.		480 .	
.	.	.	.	.	.	
.	.	.	.	.	.	

.	.	.	.		625	.	
.	.	.	.	.			369
.	.	.	.	.		.	
.	.	.	.		530		481
.	.	.	.		480	.	
.	.	.	.	.			388
.	.	.	.	.			473
.	.	.	.	.		.	
.	.	.	.	.		.	
.	.	.	.	.			474
.	.	.	.	.			495
.	.	.	.	.			450
.	.	.	.	.			480
.	.	.	.	.		.	
.	.	.	.	.			432
.	.	.	.	.			240
.	.	.	.	.		.	
.	.	.	.	.			333
.	.	.	.	.		.	
.	.	.	.	.		.	
.	.	.	.	.		.	
.	.	.	.	.		.	
.	.	.	.	.			390
.	.	.	.	.		.	
.	.	.	.	.		.	
.	.	.	.	.			422
.	.	.	.	.			390
.	.	.	.	.		.	
.	.	.	.	.		.	
.	.	.	.	.		.	
.	.	.	.	.		.	
.	.	.	.	.		.	
.	.	.	.	.		.	
.	.	.	.	.		.	
.	.	.	.	.		.	
.	.	.	.	.		.	
.	.	.	.	.		.	
.	.	.	.	.		.	
.	.	.	.	.		.	
.	.	.	.	.		.	
.	.	.	.	.		.	
.	.	.	.	.		.	
.	.	.	.	.		.	
.	.	.	.	.		.	
.	.	.	.	.		.	
.	.	.	.	.		.	
.	.	.	.	.		.	
.	.	.	.	.		.	
.	.	.	.	.		.	
.	.	.	.	.		.	
.	.	.	.	.		.	
.	.	.	.	.		.	
.	.	.	.	.		.	
.	.	.	.	.		.	
.	.	.	.	.		.	
.	.	.	.	.		.	
.	.	.	.	.		.	
.	.	.	.	.		.	

wake_up_2020_10	wake_up_2020_11	wake_up_2020_12	wake_up_2020_13	wake_up_2020_14
.	.	.	.	.
.	.	.	.	.
.	.	.	.	.
.	.	.	.	.
.	.	.	.	.
.	.	.	.	.
.	.	.	.	.
.	.	.	.	.
.	.	.	.	.
.	.	.	.	.
.	.	.	.	.
.	.	.	.	.
.	.	.	.	.
.	.	.	.	.
.	.	.	.	.
.	.	.	.	.
.	.	.	.	.
.	.	.	.	.
.	.	.	.	.
.	.	.	.	.
.	.	.	.	.
.	.	.	.	.
.	.	.	.	.
.	.	.	.	.
.	.	.	.	.
.	.	.	.	.
.	.	.	.	.
.	.	.	.	.
.	.	.	.	.
.	.	.	.	.
.	.	.	.	.
.	.	.	.	.
.	.	.	.	.
.	.	.	.	.
.	.	.	.	.
.	.	.	.	.
.	.	.	.	.
.	.	.	.	.
.	.	.	.	.
.	.	.	.	.
.	.	.	.	.
.	.	.	.	.
.	.	.	.	.
.	.	.	.	.
.	.	.	.	.
.	.	.	.	.
720	442	.	.	.
.	.	.	.	.
.	.	.	.	.
.	.	.	.	.
.	.	.	.	.
.	.	.	.	.
.	.	.	.	.
.	.	.	.	.
.	.	.	.	.
.	.	.	.	.
.	.	.	.	.
.	.	.	.	.

451

420

420

401 404 396 418 412

405 413 411

440 460 434 516

471

422

431 453 415 387 410

395

540 604

555

366

747

.		.		.		.		.	
	466		305		367		303		286
.		.		.		.		.	
	622		574		390		525	.	
.		.		.		.		.	
.		.		.		.		.	
.			460	.		.		.	
.		.		.		.		.	
.		.		.		.		.	
.		.		.		.		.	
.		.		.		.		.	
.		.		.		.		.	
	510	.		.		.		.	
.		.		.		.		.	
	392	.		.		.		.	
.		.		.		.		.	
.			567	.		.		.	
	350	.		.		.		.	
.		.		.		.		.	
	330	.		.		.		.	
.		.		.		.		.	
.		.		.		.		.	
.		.		.		.		.	
.		.		.		.		.	
.		.		.		.		.	
.		.		.		.		.	
.		.		.		.		.	
.		.		.		.		.	
	618	.		.		.		.	
.		.		.		.		.	
	360	.		.		.		.	
	246	.		.		.		.	
	557	.		.		.		.	
	245	.		.		.		.	
	490	.		.		.		.	
	532	.		.		.		.	
	375	.		.		.		.	
	410	.		.		.		.	
	459		472		504		503		524
.		.		.		.		.	
.		.		.		.		.	
	434	.		.		.		.	
	570	.		.		.		.	
	575		574	.		.		.	
	293	.		.		.		.	
	405	.		.		.		.	
.		.		.		.		.	
	530		585	.		.		.	
	540	.		.		.		.	
	540		541	.		.		.	
	420	.		.		.		.	
.		.		.		.		.	
	410		430	.		.		.	
	570		510	.		.		.	
.			468	.		.		.	
	390	.		.		.		.	
	310	.		.		.		.	
.			490	.		.		.	
.			413	.		.		.	

.	385	.	.	.
.	.	.	.	.
.	.	.	.	.
.	480	.	.	.
.	427	405	421	400
.	540	.	.	.
.	375	.	.	.
.	373	.	.	.
.	428	411	.	.
.	585	390	330	.
.	583	.	.	.
.	491	.	.	.
.	.	.	.	.
.	.	.	.	.
.	360	.	.	.
.	.	.	.	.
.	403	377	402	398
.	420	.	.	.
.	304	325	292	371
.	477	366	660	.
.	570	.	.	.
.	.	.	.	.
.	435	.	.	.
.	445	435	.	.
.	.	.	.	.
.	.	.	.	.
.	.	.	.	.
.	540	.	.	.
.	.	.	.	.
.	.	.	.	.
.	.	430	.	.
.	.	.	.	.
.	.	.	.	.
.	.	.	.	.
.	.	.	.	.
.	.	.	.	.
.	.	.	.	.
.	.	451	.	.
.	.	.	.	.
.	.	320	335	.
.	.	.	.	.
.	.	.	.	.
.	.	.	.	.
.	.	.	.	.
.	.	393	400	.
.	.	.	.	.
.	.	435	.	.
.	.	428	.	.
.	.	.	445	.
.	.	.	.	.
.	.	.	525	.
.	.	.	.	.
.	.	.	465	.
.	.	.	390	.
.	.	.	240	.
.	.	.	335	.
.	.	.	.	.
.	.	.	.	.
.	.	.	.	.

.	.	.	720	.
.	.	.	679	569
.	.	.	450	.
.	.	.	.	480
.	.	.	300	.
.	.	.	540	.
.	.	.	450	.
.	.	.	450	.
.	.	.	.	.
.	.	.	480	.
.	.	.	276	.
.	.	.	420	472
.	.	.	.	.
.	.	.	301	.
.	.	.	.	338
.	.	.	.	420
.	.	.	.	780
.	.	.	.	350
.	.	.	.	493
.	.	.	.	287
.	.	.	.	570
.	.	.	.	475
.	.	.	.	438
.	.	.	.	.
.	.	.	.	554
.	.	.	.	363
.	.	.	.	345
.	.	.	.	.
.	.	.	.	533
.	.	.	.	.
.	.	.	.	490
.	.	.	.	580
.	.	.	.	525
.	.	.	.	.
.	.	.	.	.
.	.	.	.	300
.	.	.	.	385
.	.	.	.	.
.	.	.	.	431
.	.	.	.	195
.	.	.	.	600
.	.	.	.	.
.	.	.	.	435
.	.	.	.	350
.	.	.	.	.
.	.	.	.	780
.	.	.	.	.
.	.	.	.	.
.	.	.	.	.
.	.	.	.	.
.	.	.	.	.
.	.	.	.	.
.	.	.	.	.
.	.	.	.	.
.	.	.	.	.
.	.	.	.	.
.	.	.	.	.
.	.	.	.	.
.	.	.	.	.

wake_up_2020_15	wake_up_2020_16	wake_up_2020_17	bedtime_2019_2	bedtime_2019_3
.	.	.	.	.
.	.	.	.	.
.	.	.	.	.
.	.	.	.	.
.	.	.	.	.
.	.	.	.	.
.	.	.	.	.
.	.	.	.	.
.	.	.	.	.
.	.	.	.	.
.	.	.	1419	.
.	.	.	.	.
.	.	.	.	.
.	.	.	.	.
.	.	.	.	.
.	.	.	.	.
.	.	.	.	.
.	.	.	.	1470
.	.	.	.	.
.	.	.	.	.
.	.	.	.	.
.	.	.	.	.
.	.	.	.	.
.	.	.	.	.
.	.	.	.	.
.	.	.	1380	.
.	.	.	.	.
.	.	.	.	.
.	.	.	.	.
.	.	.	.	.
.	.	.	.	.
.	.	.	.	.
.	.	.	.	.
.	.	.	.	.
.	.	.	.	.
.	.	.	.	.
.	.	.	.	.
.	.	.	.	.
.	.	.	.	.
.	.	.	.	.
.	.	.	1491	.
.	.	.	.	.
.	.	.	.	.
.	.	.	.	.
.	.	.	.	.
.	.	.	.	.
.	.	.	.	.
.	.	.	.	.
.	.	.	1470	.
.	.	.	.	1501
.	.	.	.	.
.	.	.	.	.
.	.	.	.	.
.	.	.	.	.
.	.	.	.	.
.	.	.	.	.
.	.	.	.	.
.	.	.	.	.

1449	1473
1547	1491
1360	1335
1334	1364
1375	1334

.	.	.	.	.
.	.	.	.	.
.	.	.	.	.
.	.	.	.	.
.	.	.	.	.
.	.	.	.	.
.	.	.	.	.
.	.	.	.	.
.	.	.	.	.
.	.	.	.	.
.	.	.	.	.
.	.	.	.	.
.	.	.	.	.
.	.	.	.	.
.	.	.	.	.
.	.	.	.	.
.	.	.	.	.
.	.	.	.	.
.	.	.	1488	1232
.	.	.	1407	1468
.	.	.	.	.
.	.	.	.	.
.	.	.	.	.
.	.	.	.	1435
510	.	.	.	.
.	.	.	.	.
.	.	.	.	.
.	.	.	.	.
.	.	.	1488	1507
.	.	.	.	.
.	.	.	.	.
.	.	.	.	.
.	.	.	.	.
.	.	.	.	.
.	.	.	1429	.
.	.	.	.	.
.	.	.	.	.
.	.	.	.	.
.	.	.	.	.
.	.	.	.	.
403	398	391	.	.
.	.	.	.	.
.	.	.	.	.
.	.	.	.	.
.	.	.	.	.
.	.	.	1558	.
.	.	.	1493	1502
.	.	.	.	.
.	.	.	.	.
.	.	.	.	.
.	.	.	.	.
.	.	.	.	.
.	.	.	.	.
.	.	.	.	.
.	.	.	1544	.
.	.	.	1407	.
.	.	.	.	.
.	.	.	.	.
.	.	.	.	.

1479 1367
1335
1447
1498
1509
1415
1501
1505

452

340

460

409

329	348	348	
	360	760	
458	543	504	

430			
248	285	234	

.	.	.	.	.
	705	556	520 .	.
.	.	.	.	.
.	.	.	.	.
.	.	.	.	.
.	.	.	.	.
.	.	.	.	.
.	.	.	.	.
.	.	.	.	.
.	.	.	.	.
.	.	.	.	.
	486 .	.	.	.
.	.	.	.	.
.	.	.	.	.
.	.	.	.	.
.	.	.	.	.
.	.	.	.	.
.	.	.	.	.
.	.	.	.	.
.	.	.	.	.
.	.	.	.	.
.	.	.	.	.
.	.	.	.	.
.	.	.	.	.
	624	660 .	.	.
.	.	.	.	.
.	.	.	.	.
.	.	.	.	.
	511 .	.	.	.
.	.	.	.	.
.	.	.	.	.
.	.	.	.	.
.	.	.	.	.
.	.	.	.	.
.	.	.	.	.
.	.	.	.	.
.	.	.	.	.
.	.	.	.	.
	488	540	472 .	.
	534 .	.	.	.
.	.	.	.	.
	528 .		620 .	.
	418 .	.	.	.
.	.	.	.	.
.	.	.	.	.
.	.	.	.	.
	439 .	.	.	.
	375 .	.	.	.
.	.	.	.	.
	521 .	.	.	.
	520 .	.	.	.
	370 .	.	.	.
	420 .	.	.	.
.	.	.	.	.
.	.	.	.	.
.	.	.	.	.
	524 .	.	.	.
	400 .	.	.	.
	556 .	.	.	.

	459		460		491	.	.
.		.		.		.	.
.		.		.		.	.
	420	.		.		.	.
.		.		.		.	.
.		.		.		.	.
	540	.		.		.	.
	330	.		.		.	.
	510	.		.		.	.
	481	.		.		.	.
.		.		.		.	.
	453	.		.		.	.
	432	.		.		.	.
.		.		.		.	.
.		.		.		.	.
.		.		.		.	.
.		.		.		.	.
	430	.		.		.	.
	552	.		.		.	.
.		.		.		.	.
	398		390	.		.	.
	720	.		.		.	.
.		.		.		.	.
.		.		.		.	.
.		.		.		.	.
.		.		.		.	.
	677		562		574	.	.
	445		510	.		.	.
	430	.		.		.	.
.		.		.		.	.
	451	.		.		.	.
	653	.		.		.	.
	700	.		.		.	.
	310	.		.		.	.
.		.		.		.	.
.		.		.		.	.
	330	.		.		.	.
.			415	.		.	.
.			540	.		.	.
.			360	.		.	.
.			388	.		.	.
.			480	.		.	.
.			370	.		.	.
.			407	.		.	.
.			450	.		.	.
.			505	.		.	.
.			610	.		.	.
.			480	.		.	.
.		.		.		.	.
.			540	.		.	.
.		.		.		.	.
.			735		519	.	.
.			278	.		.	.
.			301		291	.	.
.			350	.		.	.
.		.		.		.	.
.			455	.		.	.
.			303	.		.	.
.			528		475	.	.

.		488		443	.	.
.		510	.		.	.
.		746		664	.	.
.	.		.		.	.
.	.		.		.	.
.	.		.		.	.
.		510	.		.	.
.		522		510	.	.
.		540	.		.	.
.		586	.		.	.
.		545		513	.	.
.	.		.		.	.
.		660	.		.	.
.	.		.		.	.
.	.		.		.	.
.	.		.		.	.
.		380	.		.	.
.		570		599	.	.
.		560	.		.	.
.	.		.		.	.
.	.		.		.	.
.		390		425	.	.
.		469	.		.	.
.	.			565	.	.
.	.		.		.	.
.	.		.		.	.
.	.			423	.	.
.	.		.		.	.
.	.		.		.	.
.	.			510	.	.
.	.		.		.	.
.	.		.		.	.
.	.			554	.	.
.	.			400	.	.
.	.			360	.	.
.	.			390	.	.
.	.			480	.	.
.	.			418	.	.
.	.		.		.	.
.	.			490	.	.
.	.			390	.	.
.	.		.		.	.
.	.		.		.	.
.	.		.		.	.
.	.		.		.	.
.	.		.		.	.
.	.			330	.	.
.	.			433	.	.
.	.			380	.	.
.	.			617	.	.
.	.		.		.	.
.	.			320	.	.
.	.			423	.	.
.	.			360	.	.
.	.		.		.	.
.	.			451	.	.
.	.			471	.	.
.	.		.		.	.
.	.			477	.	.

.	.		642	.	.
.	.	.		.	.
.	.		390	.	.
.	.	.		.	.
.	.		366	.	.
.	.	.		.	.
.	.		650	.	.
.	.		378	.	.
.	.		560	.	.
.	.		460	.	.
.	.		450	.	.
.	.		240	.	.
.	.	.		.	.
.	.		435	.	.
.	.		415	.	.
.	.		400	.	.
.	.	.		.	.
.	.		320	.	.
.	.		490	.	.
.	.		420	.	.
.	.	.		.	.
.	.	.		.	.
.	.	.		.	.
.	.	.		.	.
.	.	.		.	.

bedtime_2019_4	bedtime_2019_5	bedtime_2019_6	bedtime_2019_7	bedtime_2019_8	bedtime_2019_9
.	.	.	.	.	.
.	.	.	.	.	.
.	.	.	.	.	.
.	.	.	.	.	.
.	.	.	.	.	.
.	.	.	.	.	.
.	.	.	.	.	.
.	.	.	.	.	.
.	.	.	.	.	.
.	.	.	.	.	.
1427	1439	1410	1429	.	.
.	.	.	.	.	.
.	.	.	.	.	.
.	.	.	.	.	.
.	.	.	.	.	.
.	.	.	.	.	.
.	.	.	.	.	.
.	.	.	.	.	.
.	.	.	.	.	.
.	.	.	.	.	.
.	.	.	.	.	.
.	.	.	.	.	.
.	.	.	.	.	.
.	.	.	.	.	.
.	.	.	.	.	.
.	.	.	.	.	.
.	.	.	.	.	.
.	.	.	.	.	.
.	.	.	.	.	.
.	.	.	.	.	.
.	.	.	.	.	.
.	.	.	.	.	.
.	.	.	.	.	.
.	.	.	.	.	.
.	.	.	.	.	.
.	.	.	.	.	.
.	.	.	.	.	.
.	.	.	.	.	.
.	.	.	.	.	.
.	.	.	.	.	.
1584	1533	1450	1478	1470	1452
.	.	.	.	.	.
.	.	.	.	.	.
.	.	.	.	.	.
.	.	.	.	.	.
.	.	.	.	.	.
.	.	.	.	.	.
.	.	.	.	.	.
.	.	.	.	.	.
.	.	.	.	.	.
.	.	.	.	.	.
.	.	.	.	.	.
.	.	.	.	.	.
.	.	.	.	.	.
.	.	.	.	.	.
.	.	.	.	.	.
1449	.	.	.	.	.
.	.	.	.	.	.

			1461		
1550	1460	1586	1511	1490	1485
	1437				
1375	1396	1351	1337	1420	1361
1347	1372	1330	1314	1325	1309

		1490			
1410	1456	1326		1443	1456
1571	1620	1483	1495		
				1443	
1433					

1461					
1436	1528	1540	1505	1543	1536
1710					
1680					
1383					
1381	1394				
1440					
	1425				
	1320	1386	1556	1380	1440
	1545	1559			
	1440	1421	1425	1415	1439
	1413				

			1360			
			1560	1435	1469	1460
			1672			
			1313			
			1650			
			1417	1441		
				1404		
			1271			
				1675		
				1351		
				1440		
				1394		
				1425		
					1320	
					1327	1334
					1620	
					1356	
					1335	
						1488
					1419	1447
					1410	1378

.	.	.	.	.	.	
.	.	.	.	.		1460
.	.	.	.	.	.	
.	.	.	.	.		1462
.	.	.	.	.		1530
.	.	.	.	.	.	
.	.	.	.	.	.	
.	.	.	.	.		1290
.	.	.	.	.	.	
.	.	.	.	.		1397
.	.	.	.	.	.	
.	.	.	.	.	.	
.	.	.	.	.	.	
.	.	.	.	.	.	
.	.	.	.	.	.	
.	.	.	.	.	.	
.	.	.	.	.	.	
.	.	.	.	.	.	
.	.	.	.	.	.	
.	.	.	.	.	.	
.	.	.	.	.	.	
.	.	.	.	.	.	
.	.	.	.	.	.	
.	.	.	.	.	.	
.	.	.	.	.	.	
.	.	.	.	.	.	
.	.	.	.	.	.	
.	.	.	.	.	.	
.	.	.	.	.	.	
.	.	.	.	.	.	
.	.	.	.	.	.	
.	.	.	.	.	.	
.	.	.	.	.	.	
.	.	.	.	.	.	
.	.	.	.	.	.	
.	.	.	.	.	.	
.	.	.	.	.	.	
.	.	.	.	.	.	
.	.	.	.	.	.	
.	.	.	.	.	.	
.	.	.	.	.	.	
.	.	.	.	.	.	
.	.	.	.	.	.	
.	.	.	.	.	.	
.	.	.	.	.	.	
.	.	.	.	.	.	
.	.	.	.	.	.	
.	.	.	.	.	.	
.	.	.	.	.	.	
.	.	.	.	.	.	
.	.	.	.	.	.	
.	.	.	.	.	.	
.	.	.	.	.	.	
.	.	.	.	.	.	
.	.	.	.	.	.	
.	.	.	.	.	.	
.	.	.	.	.	.	
.	.	.	.	.	.	
.	.	.	.	.	.	

bedtime_2019_10	bedtime_2019_11	bedtime_2019_12	bedtime_2019_13	bedtime_2019_14
.	.	.	.	.
.	.	.	.	.
.	.	.	.	.
.	.	.	.	.
.	.	.	.	.
.	.	.	.	.
.	.	.	.	.
.	.	.	.	.
.	.	.	.	.
.	.	.	.	.
.	.	.	.	.
.	.	.	.	.
.	.	.	.	.
.	.	.	.	.
.	.	.	.	.
.	.	.	.	.
.	.	.	.	.
.	.	.	.	.
.	.	.	.	.
.	.	.	.	.
.	.	.	.	.
.	.	.	.	.
.	.	.	.	.
.	.	.	.	.
.	.	.	.	.
.	.	.	.	.
.	.	.	.	.
.	.	.	.	.
.	.	.	.	.
.	.	.	.	.
.	.	.	.	.
.	.	.	.	.
.	.	.	.	.
.	.	.	.	.
.	.	.	.	.
.	.	.	.	.
.	.	.	.	.
.	.	.	.	.
.	.	.	.	.
.	.	.	.	.
1506	1486	1441	1440	1475
.	.	.	.	.
.	.	.	.	.
.	.	.	.	.
.	.	.	.	1336
.	.	.	.	.
.	.	.	.	.
.	.	.	.	.
.	.	.	.	.
.	.	.	.	.
.	.	.	.	.
.	.	.	.	.
.	.	.	.	.
.	.	.	.	.
.	.	.	.	.
.	.	.	.	.
.	.	.	.	.
.	.	.	.	.

1504	1496	1520	1455	1433
1364	1354	1360		1324
			1601	
1333	1300	1359		

1602 1478

1445 1442 1430 1458

1522

1460	1463	1509	1465	1485
1357	1374	1433	1381	
		1462		
1442		1440	1451	
1366	1415	1382	1404	1440

.	.	.	.	.
.	.	.	.	.
.	.	.	.	.
.	.	.	.	.
.	.	.	.	.
.	.	.	.	.
.	.	.	.	.
1324	1320	1350	.	.
.	.	.	.	.
.	.	.	.	.
.	.	.	.	.
.	.	.	.	.
.	.	.	.	.
.	.	.	.	.
.	.	.	.	.
.	.	.	.	.
.	.	.	.	.
1468	1488	1486	1507	.
1560	.	.	.	.
.	.	.	.	.
.	.	.	.	.
1543	1513	.	.	.
1353	.	.	.	.
.	.	.	.	.
.	.	.	.	.
.	.	.	.	.
1488	.	.	.	.
.	.	.	.	.
.	.	.	.	.
.	.	.	.	.
.	.	.	.	.
.	.	.	.	.
.	.	.	.	.
.	.	.	.	.
.	.	.	.	.
.	.	.	.	.
.	.	.	.	.
.	1500	.	.	.
.	.	.	.	.
.	1268	1293	1296	.
.	.	.	.	.
.	.	.	.	.
.	.	.	.	.
.	.	.	.	.
.	.	.	.	.
.	.	.	.	.
.	1483	.	.	.
.	.	.	.	.
.	.	.	.	.
.	.	.	.	.
.	.	.	.	.
.	.	.	.	.
.	.	.	.	.
.	.	1385	.	.
.	.	1325	.	.
.	.	.	.	.
.	.	.	.	.
.	.	.	.	.
.	.	1403	.	.

.	.	1380	.	.
.	.	.	.	.
.	.	.	.	.
.	.	.	.	.
.	.	1260	.	.
.	.	1522	.	.
.	.	.	.	.
.	.	.	1500	.
.	.	.	.	.
.	.	.	.	.
.	.	.	.	.
.	.	.	.	.
.	.	.	.	.
.	.	.	.	.
.	.	.	.	.
.	.	.	.	.
.	.	.	.	.
.	.	.	.	.
.	.	1360	.	.
.	.	.	.	.
.	.	.	1322	1356
.	.	.	.	.
.	.	.	.	.
.	.	.	1475	1445
.	.	.	1320	.
.	.	.	1454	.
.	.	.	.	.
.	.	.	.	.
.	.	.	.	.
.	.	.	1388	1361
.	.	.	1511	1479
.	.	.	1320	1380
.	.	.	.	1536
.	.	.	.	.
.	.	.	.	.
.	.	.	.	.
.	.	.	.	.
.	.	.	.	.
.	.	.	.	.
.	.	.	.	.
.	.	.	.	.
.	.	.	.	.
.	.	.	.	.
.	.	.	.	1320
.	.	.	.	1433
.	.	.	.	.
.	.	.	.	.
.	.	.	.	.
.	.	.	.	.
.	.	.	.	.
.	.	.	.	1498
.	.	.	.	.
.	.	.	.	.
.	.	.	.	1454
.	.	.	.	.
.	.	.	.	.
.	.	.	.	.
.	.	.	.	.
.	.	.	.	.

bedtime_2019_15	bedtime_2019_16	bedtime_2019_17	bedtime_2020_2	bedtime_2020_3	bedtime_2020_4
.	.	.	.	.	.
.	.	.	.	.	.
.	.	.	.	.	.
.	.	.	.	.	.
.	.	.	.	.	.
.	.	.	.	.	.
.	.	.	.	.	.
.	.	.	.	.	.
.	.	.	.	.	.
.	.	.	.	.	.
.	.	.	.	.	.
.	.	.	.	.	.
.	.	.	.	.	.
.	.	.	.	.	.
.	.	.	.	.	.
.	.	.	.	.	.
.	.	.	.	.	.
.	.	.	.	.	.
.	.	.	.	.	.
.	.	.	.	.	.
.	.	.	.	.	.
.	.	.	.	.	.
.	.	.	.	.	.
.	.	.	.	.	.
.	.	.	.	.	.
.	.	.	.	.	.
.	.	.	.	.	.
.	.	.	.	.	.
.	.	.	.	.	.
.	.	.	.	.	.
.	.	.	.	.	.
.	.	.	.	.	.
.	.	.	.	.	.
.	.	.	.	.	.
.	.	.	.	.	.
.	.	.	.	.	.
.	.	.	.	.	.
.	.	.	.	.	.
.	.	.	.	.	.
.	.	.	.	.	.
1480	.	.	.	.	.
.	.	.	.	.	.
.	.	.	.	.	.
.	.	.	.	.	.
1413	1373	1431	.	.	.
.	.	.	.	.	.
.	.	.	.	.	.
.	.	.	.	.	.
.	.	.	.	.	.
.	.	.	.	.	.
.	.	.	.	.	.
.	.	.	.	.	.
.	.	.	.	.	.
.	.	.	.	.	.
.	.	.	.	.	.
.	.	.	.	.	.
.	.	.	.	.	.
.	.	.	.	.	.

1480	1422	1430		
			1421	
1324	1367	1356		

1427

1412

1451

1471	1485	1459
	1456	
1426	1440	1443

1327	1362	1377
1355	1350	1451

.	.	.	.	.	.
.	.	.	.	.	.
1479	.	.	.	.	.
1451	.	.	.	.	.
1399	.	.	.	.	.
.	.	.	.	.	.
.	.	.	.	.	.
1232	.	.	.	.	.
.	.	.	.	.	.
.	.	.	.	.	.
1591	.	.	.	.	.
.	.	.	.	.	.
.	.	.	.	.	.
.	.	.	.	.	.
.	.	.	.	.	.
1440	.	.	.	.	.
.	.	.	.	.	.
.	.	.	.	.	.
.	.	.	.	.	.
.	1401	1380	.	.	.
.	.	.	.	.	.
1545	1528	.	.	.	.
.	.	.	.	.	.
.	.	.	.	.	.
.	.	.	.	.	.
.	1330	.	.	.	.
.	1301	.	.	.	.
.	.	.	.	.	.
.	.	.	.	.	.
.	.	.	.	.	.
.	.	.	.	.	.
.	.	.	.	.	.
.	.	.	.	.	.
.	.	.	.	.	.
.	1452	.	.	.	.
.	.	.	.	.	.
.	.	.	.	.	.
.	.	.	.	.	.
.	.	.	.	.	.
.	.	.	.	.	.
.	.	.	.	.	.
.	.	.	.	.	.
.	.	.	.	.	.
.	.	.	.	.	.
.	.	.	.	.	.
.	.	.	.	.	.
.	.	.	.	.	.
.	.	.	.	.	.
.	.	.	.	.	.
.	.	.	.	.	.
.	.	.	.	.	.
.	.	.	.	.	.
.	.	.	.	.	.
.	.	.	.	.	.
.	.	.	.	.	.
.	.	.	.	.	.
.	.	.	.	.	.
.	.	.	.	.	.
.	.	.	.	.	.

1470	1449	1425
1571	1582	1568
1440		
1436	1389	1431
		1573
1440		
1384		
1380		
1436		

.	.	.	.	.	.	
.	.	.	.	.	.	
.	.	.	.	.	.	
.	.	.	.	.	.	
.	.	.	.	.	.	
.	.	.	.	.	.	
.	.	.	.	.	.	
.	.	.	.	.	.	
.	.	.		1503 .	.	
.	.	.	.	.	.	
.	.	.	.	.	.	
.	.	.	.	.	.	
.	.	.		1428 .	.	
.	.	.	.	.	.	
.	.	.	.	.	.	
.	.	.	.	.	.	
.	.	.		1539 .	.	
.	.	.		1422 .	.	
.	.	.		1556	1485 .	
.	.	.	.		1554 .	
.	.	.	.	.	.	
.	.	.	.	.	.	
.	.	.		1412 .	.	
.	.	.	.	.	.	
.	.	.	.	.	.	
.	.	.	.	.	.	
.	.	.	.	.	.	
.	.	.	.	.	.	
.	.	.		1383 .	.	
.	.	.	.	.	.	
.	.	.		1465 .	.	
.	.	.	.	.	.	
.	.	.	.	.	.	
.	.	.	.	.	.	
.	.	.	.	.	.	
.	.	.		1462 .	.	
.	.	.	.	.	.	
.	.	.	.	.	.	
.	.	.	.	.	.	
.	.	.	.	.	.	
.	.	.	.	.	.	
.	.	.	.	.	.	
.	.	.	.	.	.	
.	.	.	.		1339 .	
.	.	.	.		1470 .	
.	.	.	.	.	.	
.	.	.	.		1290 .	
.	.	.	.	.	.	
.	.	.	.	.	.	
.	.	.	.	.	.	
.	.	.	.	.	.	
.	.	.		1379	1406 .	
.	.	.	.		1620 .	
.	.	.	.	.	.	
.	.	.	.	.	.	
.	.	.	.		1365	1370
.	.	.	.	.	.	
.	.	.	.	.	.	
.	.	.	.	.	.	

.	.	.	.	.	.
.	.	.	.	.	.
.	.	.	.	.	.
.	.	.	.	.	.
.	.	.	.	.	.
.	.	.	.	1380	.
.	.	.	.	.	.
.	.	.	.	.	.
.	.	.	.	.	.
.	.	.	.	.	.
.	.	.	.	.	.
.	.	.	.	.	.
.	.	.	.	1256	.
.	.	.	.	.	.
.	.	.	.	.	.
.	.	.	.	.	.
.	.	.	.	.	.
.	.	.	.	.	1571
.	.	.	.	.	.
.	.	.	.	.	.
.	.	.	.	.	.
.	.	.	.	.	1386
.	.	.	.	.	.
.	.	.	.	.	.
.	.	.	.	.	.
.	.	.	.	.	1419
.	.	.	.	.	.
.	.	.	.	.	.
.	.	.	.	.	.
.	.	.	.	.	.
.	.	.	.	.	.
.	.	.	.	.	.
.	.	.	.	.	1466
.	.	.	.	.	.
.	.	.	.	.	.
.	.	.	.	.	.
.	.	.	.	.	1405
.	.	.	.	.	1572
.	.	.	.	.	1373
.	.	.	.	.	.
.	.	.	.	.	.
.	.	.	.	.	.
.	.	.	.	.	1462
.	.	.	.	.	.
.	.	.	.	.	.
.	.	.	.	.	.
.	.	.	.	.	.
.	.	.	.	.	.
.	.	.	.	.	1522
.	.	.	.	.	.
.	.	.	.	.	1427
.	.	.	.	.	.
.	.	.	.	.	.
.	.	.	.	.	1373
.	.	.	.	.	.
.	.	.	.	.	.
.	.	.	.	.	.
.	.	.	.	.	.
.	.	.	.	.	.

bedtime_2020_5	bedtime_2020_6	bedtime_2020_7	bedtime_2020_8	bedtime_2020_9	bedtime_2020_10
.	.	.	.	.	.
.	.	.	.	.	.
.	.	.	.	.	.
.	.	.	.	.	.
.	.	.	.	.	.
.	.	.	.	.	.
.	.	.	.	.	.
.	.	.	.	.	.
.	.	.	.	.	.
.	.	.	.	.	.
.	.	.	.	.	.
.	.	.	.	.	.
.	.	.	.	.	.
.	.	.	.	.	.
.	.	.	.	.	.
.	.	.	.	.	.
.	.	.	.	.	.
.	.	.	.	.	.
.	.	.	.	.	.
.	.	.	.	.	.
.	.	.	.	.	.
.	.	.	.	.	.
.	.	.	.	.	.
.	.	.	.	.	.
.	.	.	.	.	.
.	.	.	.	.	.
.	.	.	.	.	.
.	.	.	.	.	.
.	.	.	.	.	.
.	.	.	.	.	.
.	.	.	.	.	.
.	.	.	.	.	.
.	.	.	.	.	.
.	.	.	.	.	.
.	.	.	.	.	.
.	.	.	.	.	.
.	.	.	.	.	.
.	.	.	.	.	.
.	.	.	.	.	.
.	.	.	.	.	.
.	.	.	.	.	.
.	.	.	.	.	.
.	.	.	.	.	.
.	.	.	.	.	.
.	.	.	.	.	.
.	.	.	.	.	.
.	.	.	.	.	.
.	.	.	.	.	.
.	.	.	.	.	.
.	.	.	.	.	.
.	.	.	.	.	.
.	.	.	.	.	.
.	.	.	.	.	.
.	.	.	.	.	.
.	.	.	.	.	.
.	.	.	.	.	.
.	.	.	.	.	.
.	.	.	.	.	.

1407					
1450	1437	1428	1420	1406	1414
1482	1381				

1311		
1430		
1467	1418	1347

.	.	.	.	.	.	
.	.	.	.	.	.	
.	.	.	.	.	.	
	1410 .	.	.	.	.	
.	.	.	.	.	.	
.	.	.	.	.	.	
.	.	.	.	.	.	
.	.	.	.	.	.	
.	.	.	.	.	.	
.	.	.	.	.	.	
	1430 .	.	.	.	.	
.	.	.	.	.	.	
	1358 .	.	.	.	.	
.	.	.	.	.	.	
.	.	.	.	.	.	
.	.	.	.	.	.	
	1500 .	.	.	.	.	
	1481 .	.	.	.	.	
.	.	.	.	.	.	
	1359	1361	1424	1380	1362	1358
.	.	.	.	.	.	
.	.	.	.	.	.	
.	.	.	.	.	.	
	1535 .	.	.	.	.	
.	.	.	.	.	.	
	1488 .	.	.	.	.	
.	.	.	.	.	.	
.	.	.	.	.	.	
.	.	.	.	.	.	
.	.	.	.	.	.	
.	.	.	.	.	.	
	1423 .	.	.	.	.	
.	.	.	.	.	.	
.	.	.	.	.	.	
.	.	.	.	.	.	
.	.	.	.	.	.	
.	.	.	.	.	.	
.	.	.	.	.	.	
.	.	.	.	.	.	
.	.	.	.	.	.	
.	.	.	.	.	.	
.	.	.	.	.	.	
.	.	.	.	.	.	
.	.	.	.	.	.	
.	.	.	.	.	.	
.	.	.	.	.	.	
.	.	.	.	.	.	
.		1420 .	.	.	.	
.		1387 .	.	.	.	
.		1457 .	.	.	.	
.	.	.	.	.	.	
.	.	.	.	.	.	
.	.	.	.	.	.	
.	.	.	.	.	.	
.	.	.	.	.	.	
.	.	.	.	.	.	
.		1351 .	.	.	.	
.	.	.	.	.	.	
.	.	.	.	.	.	

.	.		.		.		.		.	
.		1455	.		.		.		.	
.		1476		1497		1459	.		.	
.	.		.		.		.		.	
.	.		.		.		.		.	
.	.		.		.		.		.	
.		1432	.		.		.		.	
.	.		.		.		.		.	
.	.		.		.		.		.	
.	.		.		.		.		.	
.	.		.		.		.		.	
.	.		.		.		.		.	
.	.		.		.		.		.	
.	.		.		.		.		.	
.	.		.		.		.		.	
.	.			1560	.		.		.	
.	.		.		.		.		.	
.	.		.		.		.		.	
.	.		.		.		.		.	
.	.			1300		1280		1280	.	
.	.		.		.		.		.	
.	.		.		.		.		.	
.	.		.		.		.		.	
.	.		.		.		.		.	
.	.		.		.		.		.	
.	.			1290	.		.		.	
.	.		.		.		.		.	
.	.			1440	.		.		.	
.	.		.		.		.		.	
.	.			1440	.		.		.	
.	.		.		.		.		.	
.	.		.		.		.		.	
.	.		.		.		.		.	
.	.		.		.		.		.	
.	.		.		.		.		.	
.	.		.		.		.		.	
.	.		.		.		.		.	
.	.		.		.		.		.	
.	.		.			1441	.		.	
.	.			1200		1445	.		.	
.	.		.		.		.		.	
.	.		.		.		.		.	
.	.		.		.		.		.	
.	.		.		.		.		.	
.	.		.			1527	.		.	
.	.		.			1443	.		.	
.	.		.		.		.		.	
.	.		.		.		.		.	
.	.		.			1509		1501	.	
.	.		.		.		.		.	
.	.		.			1466		1491		1448
.	.		.		.		.		.	
.	.		.			1380	.		.	
.	.		.		.		.		.	
.	.		.		.		.		.	
.	.		.		.			1590	.	
.	.		.		.		.		.	
.	.		.		.		.		.	
.	.		.		.		.		.	

.	.	.	.	.		.	
.	.	.	.		1382		1566
.	.	.	.	.		.	
.	.	.	.		1477		1488
.	.	.	.	.		.	
.	.	.	.	.		.	
.	.	.	.		1531	.	
.	.	.	.	.		.	
.	.	.	.	.		.	
.	.	.	.	.		.	
.	.	.	.	.		.	
.	.	.	.		1500	.	
.	.	.	.	.		.	
.	.	.	.	.		.	
.	.	.	.	.		.	
.	.	.	.	.		.	
.	.	.	.	.		.	
.	.	.	.		1384	.	
.	.	.	.	.		.	
.	.	.	.	.		.	
.	.	.	.	.		.	
.	.	.	.	.		.	
.	.	.	.	.		.	
.	.	.	.	.		.	
.	.	.	.	.		.	
.	.	.	.	.		.	
.	.	.	.	.		.	
.	.	.	.	.		.	
.	.	.	.	.			1599
.	.	.	.	.		.	
.	.	.	.	.			1410
.	.	.	.		1560	.	
.	.	.	.	.		.	
.	.	.	.	.			1335
.	.	.	.	.			1500
.	.	.	.	.			1346
.	.	.	.	.		.	
.	.	.	.	.		.	
.	.	.	.	.			1373
.	.	.	.	.		.	
.	.	.	.	.		.	
.	.	.	.	.		.	
.	.	.	.	.		.	
.	.	.	.	.		.	
.	.	.	.	.		.	
.	.	.	.	.		.	
.	.	.	.	.		.	
.	.	.	.	.			1590
.	.	.	.	.		.	
.	.	.	.	.		.	
.	.	.	.	.			1380
.	.	.	.	.		.	
.	.	.	.	.		.	
.	.	.	.	.		.	
.	.	.	.	.		.	
.	.	.	.	.		.	
.	.	.	.	.			1361
.	.	.	.	.		.	
.	.	.	.	.		.	

bedtime_2020_11	bedtime_2020_12	bedtime_2020_13	bedtime_2020_14	bedtime_2020_15
.	.	.	.	.
.	.	.	.	.
.	.	.	.	.
.	.	.	.	.
.	.	.	.	.
.	.	.	.	.
.	.	.	.	.
.	.	.	.	.
.	.	.	.	.
.	.	.	.	.
.	.	.	.	.
.	.	.	.	.
.	.	.	.	.
.	.	.	.	.
.	.	.	.	.
.	.	.	.	.
.	.	.	.	.
.	.	.	.	.
.	.	.	.	.
.	.	.	.	.
.	.	.	.	.
.	.	.	.	.
.	.	.	.	.
.	.	.	.	.
.	.	.	.	.
.	.	.	.	.
.	.	.	.	.
.	.	.	.	.
.	.	.	.	.
.	.	.	.	.
.	.	.	.	.
.	.	.	.	.
.	.	.	.	.
.	.	.	.	.
.	.	.	.	.
.	.	.	.	.
.	.	.	.	.
.	.	.	.	.
.	.	.	.	.
.	.	.	.	.
.	.	.	.	.
.	.	.	.	.
.	.	.	.	.
.	.	.	.	.
.	.	.	.	.
.	.	.	.	.
1539	.	.	.	.
.	.	.	.	.
.	.	.	.	.
.	.	.	.	.
.	.	.	.	.
.	.	.	.	.
.	.	.	.	.
.	.	.	.	.
.	.	.	.	.
.	.	.	.	.
.	.	.	.	.
.	.	.	.	.

1423 1404

1376	1374	1375	1411	1402

1349	1288	1301	1283	1321
1481				
1440				
1460				
1396	1381	1416	1419	1369
1552				
1454				

.	.	.	.	.
.	.	.	.	.
.	.	.	.	.
.	.	.	.	.
	1425	1440	1440 .	.
	1555 .	.	.	.
.	.	.	.	.
	1430 .	.	.	.
	1440 .	.	.	.
.	.		1471 .	.
.	.	.	.	.
.	.	.	.	.
.	.	.	.	.
.	.	.	.	.
.	.	.	.	.
.	.	.	.	.
	1534	1454	1528	1540 .
.	.	.	.	.
	1298	1412 .		1433 .
.	.		1680 .	.
.	.	.	.	.
.	.	.	.	.
.	.	.	.	.
.	.	.	.	.
.	.	.	.	.
.	.	.	.	.
.	.	.	.	.
.	.	.	.	.
.	.	.	.	.
.	.	.	.	.
.	.	.	.	.
.	.	.	.	.
.	.	.	.	.
.	.	.	.	.
.	.	.	.	.
.	.	.	.	.
.	.	.	.	.
.		1437 .	.	.
.	.	.	.	.
.	.	.	.	.
.		1539 .	.	.
.	.	.	.	.
.	.	.	.	.
.	.	.	.	.
.	.	.	.	.
.	.	.	.	.
.		1319 .	.	.
.	.	.	.	.
.	.	.	.	.
.		1382 .	.	.
.		1411 .	.	.
.	.	.	.	.
.	.		1444 .	.
.	.	.	.	.
.	.	.	.	.
.	.	.	.	.
.	.	.	.	.
.	.	.	.	.
.	.	.	.	.

.	.	.		.		.	
.	.		1611		1568		1627
.	.	.		.		.	
.	.	.		.		.	
.	.	.		.		.	
.	.		1515	.		.	
.	.	.		.		.	
.	.	.		.		.	
.	.	.		.		.	
.	.	.		.		.	
.	.	.		.		.	
.	.	.		.		.	
.	.	.		.		.	
.	.	.		.		.	
.	.	.			1419	.	
.	.	.		.		.	
.	.	.		.		.	
.	.	.			1430	.	
.	.	.		.		.	
.	.	.		.		.	
.	.	.		.		.	
.	.	.			1440	.	
.	.	.			1392		1488
.	.	.		.		.	
.	.	.		.		.	
.	.	.			1403	.	
.	.	.		.		.	
.	.	.		.		.	
.	.	.			1437	.	
.	.	.		.		.	
.	.	.		.		.	
.	.	.		.		.	
.	.	.		.		.	
.	.	.		.		.	
.	.	.		.		.	
.	.	.		.		.	
.	.	.		.		.	
.	.	.		.		.	
.	.	.			1409		1425
.	.	.			1375		1557
.	.	.		.		.	
.	.	.		.		.	
.	.	.			1365		1320
.	.	.			1345	.	
.	.	.		.		.	
.	.	.		.		.	
.	.	.		.			1392
.	.	.		.		.	
.	.	.		.		.	
.	.	.		.		.	
.	.	.		.		.	
.	.	.		.		.	
.	.	.		.		.	
.	.	.		.		.	
.	.	.		.		.	
.	.	.		.		.	
.	.	.		.		.	
.	.	.		.		.	
.	.	.		.		.	

1376

1440

1335

1462

1222

1380
1400

bedtime_2020_16	bedtime_2020_17	happiness_2019_2	happiness_2019_3	happiness_2019_4
.	.	.	.	.
.	.	.	.	.
.	.	.	.	.
.	.	.	.	.
.	.	.	.	.
.	.	.	.	.
.	.	.	.	.
.	.	.	.	.
.	.	.	.	.
.	.	.	.	.
.	.	10	.	33
.	.	.	.	.
.	.	.	.	.
.	.	.	.	.
.	.	.	.	.
.	.	.	.	.
.	.	.	.	.
.	.	.	20	.
.	.	.	.	.
.	.	.	.	.
.	.	.	.	.
.	.	.	.	.
.	.	.	.	.
.	.	.	.	.
.	.	.	.	.
.	.	90	.	.
.	.	.	.	.
.	.	.	.	.
.	.	.	.	.
.	.	.	.	.
.	.	.	.	.
.	.	.	.	.
.	.	.	.	.
.	.	.	.	.
.	.	.	.	.
.	.	.	.	.
.	.	.	.	.
.	.	.	.	.
.	.	.	.	.
.	.	.	.	.
.	.	56	.	43
.	.	.	.	.
.	.	.	.	.
.	.	.	.	.
.	.	.	.	.
.	.	.	.	.
.	.	.	.	.
.	.	.	.	.
.	.	65	.	.
.	.	.	20	.
.	.	.	.	.
.	.	.	.	.
.	.	.	.	.
.	.	.	.	.
.	.	.	.	.
.	.	.	.	.
.	.	.	.	58
.	.	.	.	.

		14	0	
		33	24	25
		78	100	69
		40	59	45
		0	0	

100	81	
59	52	58
	100	
53	52	68
17		
21		
45	35	50
66		
44		

.	.	.		.		.	
.	.		71		27	.	
.	.	.		.		.	
.	.		0	.		.	
.	.	.		.		.	
.	.	.		.		.	
.	.		0	.		.	
.	.	.			68		69
.	.	.		.		.	
.	.	.			46	.	
.	.	.		.		.	
.	.	.			22	.	
.	.	.		.		.	
.	.	.		.		.	
.	.	.		.		.	
.	.	.			10	.	
.	.	.		.		.	
.	.	.			75		60
.	.	.		.		.	
.	.	.		.		.	
.	.	.			75		100
.	.	.		.		.	
.	.	.		.		.	
.	.	.		.		.	
.	.	.		.		.	
.	.	.		.		.	
.	.	.		.		.	
.	.	.		.			0
.	.	.		.		.	
.	.	.		.		.	
.	.	.		.		.	
.	.	.		.		.	
.	.	.		.			70
.	.	.		.			46
.	.	.		.			100
.	.	.		.		.	
.	.	.		.		.	
.	.	.		.		.	
.	.	.		.			66
.	.	.		.		.	
.	.	.		.		.	
.	.	.		.		.	
.	.	.		.		.	
.	.	.		.		.	
.	.	.		.		.	
.	.	.		.		.	
.	.	.		.		.	
.	.	.		.		.	
.	.	.		.		.	
.	.	.		.		.	
.	.	.		.		.	
.	.	.		.		.	
.	.	.		.		.	
.	.	.		.		.	
.	.	.		.		.	
.	.	.		.		.	
.	.	.		.		.	
.	.	.		.		.	

1382	1375

1380

1464	1592	
1411	1380	
	1470	

	1393	1420 .	.	.
.	.	.	.	.
.	.	.	.	.
.	.	.	.	.
.	.	.	.	.
.	.	.	.	.
.	.	.	.	.
.	.	.	.	.
.	.	.	.	.
.	.	.	.	.
.	.	.	.	.
.	.	.	.	.
.	.	.	.	.
.	.	.	.	.
.	.	.	.	.
.	.	.	.	.
.	.	.	.	.
.	.	.	.	.
.	.	.	.	.
.	.	.	.	.
.	.	.	.	.
.	.	.	.	.
.	.	.	.	.
.	.	.	.	.
.	.	.	.	.
.	.	.	.	.
.	.	.	.	.
.	.	.	.	.
.	.	.	.	.
.	.	.	.	.
.	.	.	.	.
.	.	.	.	.
.	.	.	.	.
.	.	.	.	.
.	.	.	.	.
.	.	.	.	.
.	.	.	.	.
	1298 .	.	.	.
.	.	.	.	.
	1366 .	.	.	.
	1415 .	.	.	.
.	.	.	.	.
.	.	.	.	.
.	.	.	.	.
.	.	.	.	.
.	.	.	.	.
.	.	.	.	.
.	.	.	.	.
.	.	.	.	.
.	.	.	.	.
.	.	.	.	.
	1260 .	.	.	.
	1380 .	.	.	.
	1365 .	.	.	.
.	.	.	.	.
.	.	.	.	.
	1446 .	.	.	.
	1316 .	.	.	.
	1466 .	.	.	.

.		.		.	.	.
.		.		.	.	.
.			1443	.	.	.
.		.		.	.	.
.		.		.	.	.
.		.		.	.	.
.		.		.	.	.
	1398		1400	.	.	.
.		.		.	.	.
.		.		.	.	.
	1500		1504	.	.	.
.		.		.	.	.
.		.		.	.	.
.		.		.	.	.
.		.		.	.	.
.		.		.	.	.
.		.		.	.	.
.			1404	.	.	.
	1284	.		.	.	.
.		.		.	.	.
.		.		.	.	.
	1352	.		.	.	.
.		.		.	.	.
.		.		.	.	.
.		.		.	.	.
.		.		.	.	.
.		.		.	.	.
.		.		.	.	.
.		.		.	.	.
.		.		.	.	.
.		.		.	.	.
.		.		.	.	.
.		.		.	.	.
.		.		.	.	.
.		.		.	.	.
.		.		.	.	.
.		.		.	.	.
.			1395	.	.	.
.		.		.	.	.
.		.		.	.	.
.		.		.	.	.
.		.		.	.	.
.		.		.	.	.
.		.		.	.	.
.		.		.	.	.
.		.		.	.	.
.			1611	.	.	.
.			1478	.	.	.
.			1430	.	.	.
.		.		.	.	.
.		.		.	.	.
.		.		.	.	.
.			1300	.	.	.
.		.		.	.	.
.		.		.	.	.
.		.		.	.	.
.		.		.	.	.
.		.		.	.	.
.		.		.	.	.

1440

1404

happiness_2019_5	happiness_2019_6	happiness_2019_7	happiness_2019_8	happiness_2019_9
.	.	.	.	.
.	.	.	.	.
.	.	.	.	.
.	.	.	41	.
.	.	.	.	.
.	.	.	.	.
.	.	.	.	.
.	.	.	.	.
.	.	.	.	.
.	.	.	.	.
25	35	25	.	.
.	.	.	.	.
.	.	.	.	.
.	.	.	.	.
.	.	.	.	.
.	.	.	.	.
.	.	.	.	.
.	.	.	.	.
.	.	.	.	.
.	.	.	.	.
.	.	.	.	.
.	.	.	.	.
.	.	.	.	.
.	.	.	.	.
.	.	.	.	.
.	.	.	.	.
.	.	.	.	.
.	.	.	.	.
.	.	.	.	.
.	.	.	.	.
35	.	.	.	.
.	.	.	.	.
.	.	.	.	.
.	.	.	.	.
.	.	.	.	.
.	.	.	.	.
.	.	.	.	.
.	.	.	.	.
.	.	.	.	.
.	.	.	.	.
24	34	52	64	44
.	.	.	.	.
.	.	.	.	.
.	.	.	.	.
.	.	.	.	.
.	.	.	.	.
.	.	.	.	.
.	.	.	.	.
.	.	.	.	.
.	.	.	.	.
.	.	.	.	.
.	.	.	.	.
.	.	.	.	.
.	.	.	.	.
.	.	.	.	.
.	.	.	.	.
.	.	.	.	.
.	.	.	.	.

		97		
36	41	74	31	43
0				
86	86	96	78	100
48	54	38	53	40

	88			
55	52		54	55
63	23	64		
			50	

79	75	69	68	66
31				
21				
68				
0	38	0	0	0
27	18			
63	55	55	64	63
51				

.	.	.	.	.
.	78	.	.	.
.	0	0	0	0
.	.	.	.	.
.	.	.	.	.
.	52	.	.	.
.	53	.	.	.
.	.	.	.	.
.	100	.	.	.
.	.	.	.	.
.	.	.	.	.
.	71	64	.	.
.	25	35	.	.
.	.	.	.	.
.	29	.	.	.
.	.	.	.	.
.	.	.	.	.
.	.	21	.	.
.	.	85	.	.
.	.	50	.	.
.	.	.	.	.
.	.	.	.	.
.	.	.	.	.
.	.	.	.	.
.	.	85	.	.
.	.	92	.	.
.	.	.	.	.
.	.	.	.	.
.	.	.	.	.
.	.	.	85	.
.	.	.	.	.
.	.	.	.	.
.	.	.	.	.
.	.	.	33	29
.	.	.	.	.
.	.	.	.	.
.	.	.	11	.
.	.	.	.	.
.	.	.	.	.
.	.	.	.	.
.	.	.	.	.
.	.	.	.	.
.	.	.	0	.
.	.	.	.	.
.	.	.	.	.
.	.	.	.	.
.	.	.	.	.
.	.	.	.	.
.	.	.	100	.
.	.	.	.	10
.	.	.	.	.
.	.	.	71	46
.	.	.	.	.
.	.	.	.	.
.	.	.	.	.
.	.	.	.	.
.	.	.	.	.
.	.	.	50	43
.	.	.	.	.

0

80
72

50

60

happiness_2019_10	happiness_2019_11	happiness_2019_12	happiness_2019_13	happiness_2019_14
.	.	.	.	.
.	.	.	.	.
.	.	.	.	.
.	.	.	.	.
.	.	.	.	.
.	.	.	.	.
.	.	.	.	.
.	.	.	.	.
.	.	.	.	.
.	.	.	.	.
.	.	.	.	.
.	.	.	.	.
.	.	.	.	.
.	.	.	.	.
.	.	.	.	.
.	.	.	.	.
.	.	.	.	.
.	.	.	.	.
.	.	.	.	.
.	.	.	.	.
.	.	.	.	.
.	.	.	.	.
.	.	.	.	.
.	.	.	.	.
.	.	.	.	.
.	.	.	.	.
.	.	.	.	.
.	.	.	.	.
.	.	.	.	.
.	.	.	.	.
.	.	.	.	.
.	.	.	.	.
.	.	.	.	.
.	.	.	.	.
.	.	.	.	.
.	.	.	.	.
.	.	.	.	.
.	.	.	.	.
.	.	.	.	.
.	.	.	.	.
49	52	66	45	68
.	.	.	.	.
.	.	.	.	.
.	.	.	.	.
.	.	.	.	30
.	.	.	.	.
.	.	.	.	.
.	.	.	.	.
.	.	.	.	.
.	.	.	.	.
.	.	.	.	.
.	.	.	.	.
.	.	.	.	.
.	.	.	.	.
.	.	.	.	.
.	.	.	.	.
.	.	.	.	.
.	.	.	.	.

37	30	45	40	20
100	100	99		100
			80	
36	41	69		

0 63

43 61 48 57

67

0	0	0	0	0
21	21	17	14	
		17		
54		77	24	
47	50	54	48	53

45	45	38	
10	0	0	0
25			
56	28		
0			
31			
38			
	91		
	66	73	83
	70		
		73	
		72	
		50	

.	.	46	.	.
.	.	.	.	.
.	.	.	.	.
.	.	.	.	.
.	.	60	.	.
.	.	74	.	.
.	.	.	.	.
.	.	.	0	.
.	.	.	.	.
.	.	.	.	.
.	.	.	.	.
.	.	.	.	.
.	.	.	.	.
.	.	.	.	.
.	.	.	.	.
.	.	.	.	.
.	.	.	.	.
.	.	.	.	.
.	.	70	.	.
.	.	.	.	.
.	.	.	29	26
.	.	.	.	.
.	.	.	.	.
.	.	.	33	67
.	.	.	0	.
.	.	.	55	.
.	.	.	.	.
.	.	.	100	.
.	.	.	.	.
.	.	.	75	51
.	.	.	70	85
.	.	.	100	100
.	.	.	.	100
.	.	.	.	0
.	.	.	.	.
.	.	.	.	.
.	.	.	.	.
.	.	.	.	.
.	.	.	20	.
.	.	.	.	.
.	.	.	.	.
.	.	.	.	.
.	.	.	.	.
.	.	.	.	100
.	.	.	.	0
.	.	.	.	.
.	.	.	.	.
.	.	.	.	.
.	.	.	.	.
.	.	.	.	.
.	.	.	.	65
.	.	.	.	.
.	.	.	.	.
.	.	.	.	0
.	.	.	.	.
.	.	.	.	.
.	.	.	.	.
.	.	.	.	.
.	.	.	.	.

happiness_2019_15	happiness_2019_16	happiness_2019_17	happiness_2020_2	happiness_2020_3
.	.	.	.	.
.	.	.	.	.
.	.	.	.	.
.	.	.	.	.
.	.	.	.	.
.	.	.	.	.
.	.	.	.	.
.	.	.	.	.
.	.	.	.	.
.	.	.	.	.
.	.	.	.	.
.	.	.	.	.
.	.	.	.	.
.	.	.	.	.
.	.	.	.	.
.	.	.	.	.
.	.	.	.	.
.	.	.	.	.
.	.	.	.	.
.	.	.	.	.
.	.	.	.	.
.	.	.	.	.
.	.	.	.	.
.	.	.	.	.
.	.	.	.	.
.	.	.	.	.
.	.	.	.	.
.	.	.	.	.
.	.	.	.	.
.	.	.	.	.
.	.	.	.	.
.	.	.	.	.
.	.	.	.	.
.	.	.	.	.
.	.	.	.	.
.	.	.	.	.
.	.	.	.	.
.	.	.	.	.
.	.	.	.	.
.	.	.	.	.
74	.	.	.	.
.	.	.	.	.
.	.	.	.	.
.	.	.	.	.
43	30	28	.	.
.	.	.	.	.
.	.	.	.	.
.	.	.	.	.
.	.	.	.	.
.	.	.	.	.
.	.	.	.	.
.	.	.	.	.
.	.	.	.	.
.	.	.	.	.
.	.	.	.	.
.	.	.	.	.
.	.	.	.	.
.	.	.	.	.

36	56	18	
			87
83	100	77	

100

38

67

0	0	0	
	35		
57	52	51	

72	66	57
61	57	52

.	.	.	.	.
.	.	.	.	.
49	.	.	.	.
58	.	.	.	.
0	.	.	.	.
.	.	.	.	.
.	.	.	.	.
91	.	.	.	.
.	.	.	.	.
.	.	.	.	.
30	.	.	.	.
.	.	.	.	.
39	.	.	.	.
.	.	.	.	.
.	.	.	.	.
19	.	.	.	.
.	.	.	.	.
.	.	.	.	.
.	.	.	.	.
.	36	38	.	.
.	.	.	.	.
50	33	.	.	.
.	.	.	.	.
.	.	.	.	.
.	.	.	.	.
.	50	.	.	.
.	53	.	.	.
.	.	.	.	.
.	.	.	.	.
.	.	.	.	.
.	.	.	.	.
.	.	.	.	.
.	100	.	.	.
.	.	.	.	.
.	61	.	.	.
.	.	.	.	.
.	.	.	.	.
.	.	.	.	.
.	.	.	.	.
.	.	.	.	.
.	.	.	.	.
.	.	.	.	.
.	.	.	.	.
.	.	.	.	.
.	.	.	.	.
.	.	.	.	.
.	.	.	.	.
.	.	.	.	.
.	.	.	.	.
.	.	.	.	.
.	.	.	.	.
.	.	.	.	.
.	.	.	.	.
.	.	.	.	.
.	.	.	.	.
.	.	.	.	.
.	.	.	.	.
.	.	.	.	.
.	.	.	.	.

69	69
0	0
19	
25	41
74	
64	
15	
50	

.	.	.		0	.	
.	.	.	.		.	
.	.	.	.		.	
.	.	.	.		.	
.	.	.	.		.	
.	.	.	.		.	
.	.	.	.		.	
.	.	.	.		.	
.	.	.		82	.	
.	.	.	.		.	
.	.	.	.		.	
.	.	.	.		.	
.	.	.		80	.	
.	.	.	.		.	
.	.	.	.		.	
.	.	.	.		.	
.	.	.		0	.	
.	.	.		78	.	
.	.	.		20		29
.	.	.		80		84
.	.	.	.		.	
.	.	.	.		.	
.	.	.		42	.	
.	.	.	.		.	
.	.	.	.		.	
.	.	.	.		.	
.	.	.	.		.	
.	.	.	.		.	
.	.	.		30	.	
.	.	.	.		.	
.	.	.		50	.	
.	.	.	.		.	
.	.	.	.		.	
.	.	.	.		.	
.	.	.	.		.	
.	.	.		55	.	
.	.	.	.		.	
.	.	.	.		.	
.	.	.	.		.	
.	.	.	.		.	
.	.	.	.		.	
.	.	.	.		.	
.	.	.	.		.	
.	.	.	.			75
.	.	.	.			75
.	.	.	.		.	
.	.	.	.			87
.	.	.	.		.	
.	.	.	.		.	
.	.	.	.		.	
.	.	.	.		.	
.	.	.		89		53
.	.	.	.			50
.	.	.	.		.	
.	.	.	.		.	
.	.	.	.			70
.	.	.	.		.	
.	.	.	.		.	
.	.	.	.		.	

0

0

55

happiness_2020_4	happiness_2020_5	happiness_2020_6	happiness_2020_7	happiness_2020_8
.	.	.	.	.
.	.	.	.	.
.	.	.	.	.
.	.	.	.	.
.	.	.	.	.
.	.	.	.	.
.	.	.	.	.
.	.	.	.	.
.	.	.	.	.
.	.	.	.	.
.	.	.	.	.
.	.	.	.	.
.	.	.	.	.
.	.	.	.	.
.	.	.	.	.
.	.	.	.	.
.	.	.	.	.
.	.	.	.	.
.	.	.	.	.
.	.	.	.	.
.	.	.	.	.
.	.	.	.	.
.	.	.	.	.
.	.	.	.	.
.	.	.	.	.
.	.	.	.	.
.	.	.	.	.
.	.	.	.	.
.	.	.	.	.
.	.	.	.	.
.	.	.	.	.
.	.	.	.	.
.	.	.	.	.
.	.	.	.	.
.	.	.	.	.
.	.	.	.	.
.	.	.	.	.
.	.	.	.	.
.	.	.	.	.
.	.	.	.	.
.	.	.	.	.
.	.	.	.	.
.	.	.	.	.
.	.	.	.	.
.	.	.	.	.
.	.	.	.	.
.	.	.	.	.
.	.	.	.	.
.	.	.	.	.
.	.	.	.	.
.	.	.	.	.
.	.	.	.	.
.	.	.	.	.
.	.	.	.	.
.	.	.	.	.
.	.	.	.	.
.	.	.	.	.

71

	48			
74	71	70	69	63
0				
31	16	69		
100				

65

53			
59	12		
70	40		
20			
28			
42			
50			
70			
80			
50			
100			
60	62	73	100
	50		
	50		

61				
	51			
	0			
	100			
	50			
	70			
	90	88	74	78
	68			
	20			
	0			
		95		
		76		
		41		
		64		
		80		

.	.	.	.	.
.	.	64	.	.
.	.	48	42	59
.	.	.	.	.
.	.	.	.	.
.	.	83	.	.
.	.	89	.	.
.	.	.	.	.
.	.	.	.	.
.	.	.	.	.
.	.	0	.	.
.	.	.	.	.
.	.	.	.	.
.	.	.	.	.
.	.	.	.	.
.	.	.	50	.
.	.	.	.	.
.	.	.	.	.
.	.	.	.	.
.	.	.	41	100
.	.	.	.	.
.	.	.	.	.
.	.	.	.	.
.	.	.	.	.
.	.	.	.	.
.	.	.	50	.
.	.	.	.	.
.	.	.	26	.
.	.	.	.	.
.	.	.	70	.
.	.	.	.	.
.	.	.	.	.
.	.	.	.	.
.	.	.	.	.
.	.	.	.	.
.	.	.	.	.
.	.	.	.	.
.	.	.	.	.
.	.	.	.	0
.	.	.	100	85
.	.	.	.	.
.	.	.	.	.
.	.	.	.	.
.	.	.	.	.
.	.	.	.	50
.	.	.	.	30
.	.	.	.	.
.	.	.	.	.
.	.	.	.	33
.	.	.	.	.
.	.	.	.	62
.	.	.	.	.
.	.	.	.	71
.	.	.	.	.
.	.	.	.	.
.	.	.	.	.
.	.	.	.	.
.	.	.	.	.
.	.	.	.	.

happiness_2020_9	happiness_2020_10	happiness_2020_11	happiness_2020_12	happiness_2020_13
.	.	.	.	.
.	.	.	.	.
.	.	.	.	.
.	.	.	.	.
.	.	.	.	.
.	.	.	.	.
.	.	.	.	.
.	.	.	.	.
.	.	.	.	.
.	.	.	.	.
.	.	.	.	.
.	.	.	.	.
.	.	.	.	.
.	.	.	.	.
.	.	.	.	.
.	.	.	.	.
.	.	.	.	.
.	.	.	.	.
.	.	.	.	.
.	.	.	.	.
.	.	.	.	.
.	.	.	.	.
.	.	.	.	.
.	.	.	.	.
.	.	.	.	.
.	.	.	.	.
.	.	.	.	.
.	.	.	.	.
.	.	.	.	.
.	.	21	.	.
.	.	.	.	.
.	.	.	.	.
.	.	.	.	.
.	.	.	.	.
.	.	.	.	.
.	.	.	.	.
.	.	.	.	.
.	.	.	.	.
.	.	.	.	.
.	.	.	.	.
.	.	.	.	.
.	.	.	.	.
.	.	.	.	.
.	.	.	.	.
.	.	.	.	.
.	.	.	.	.
.	.	85	.	.
.	.	.	.	.
.	.	.	.	.
.	.	.	.	.
.	.	.	.	.
.	.	.	.	.
.	.	.	.	.
.	.	.	.	.
.	.	.	.	.
.	.	.	.	.
.	.	.	.	.
.	.	.	.	.

69	80	68	77

81 79 63 77 58

72

5

66 60

70

77	68	79	72	66
72	57	69		
100		100		
0				
0				
		60		
50				
	78			
	50			
100				
	30			
	50			
	80			
	55			
	60	60	47	46
	0			
	70	100		
	85			
		90		
	0			
	42			

.	.	.	.	.	
.	.	.	.	.	
.	.	.	.	.	
.	.	.	.	.	
.	.		50	50	50
.	.		28 .	.	
.	.	.	.	.	
.	.		28 .	.	
.	.		0	0 .	
.	.	.	.		60
.	.	.	.	.	
.	.		51 .	.	
.	.	.	.	.	
.	.	.	.	.	
.	.	.	.	.	
.	.	.	.	.	
.	.		58	73	60
.	.	.	.	.	
.	.		76	73 .	
.	.	.	.		80
.	.	.	.	.	
.	.	.	.	.	
.	.	.	.	.	
.	.	.	.	.	
.	.	.	.	.	
.	.	.	.	.	
.	.	.	.	.	
.	.	.	.	.	
.	.	.	.	.	
.	.	.	.	.	
.	.	.	.	.	
.	.	.	.	.	
.	.	.	.	.	
.	.	.	.	.	
.	.	.	.	.	
.	.	.	.	.	
.	.	.	.	.	
.	.	.		34 .	
.	.	.	.	.	
.	.	.	.	.	
.	.	.		55 .	
.	.	.	.	.	
.	.	.	.	.	
.	.	.	.	.	
.	.	.	.	.	
.	.	.	.	.	
.	.	.		87 .	
.	.	.	.	.	
.	.	.	.		81
.	.	.		55 .	
.	.	.		51 .	
.	.	.	.	.	
.	.	.	.		76
.	.	.	.	.	
.	.	.	.	.	
.	.	.	.	.	
.	.	.	.	.	
.	.	.	.	.	
.	.	.	.	.	

34

100

happiness_2020_14	happiness_2020_15	happiness_2020_16	happiness_2020_17	breakfast_2019_2
.	.	.	.	.
.	.	.	.	.
.	.	.	.	.
.	.	.	.	.
.	.	.	.	.
.	.	.	.	.
.	.	.	.	.
.	.	.	.	.
.	.	.	.	.
.	.	.	.	.
.	.	.	.	446
.	.	.	.	.
.	.	.	.	.
.	.	.	.	.
.	.	.	.	.
.	.	.	.	.
.	.	.	.	.
.	.	.	.	.
.	.	.	.	.
.	.	.	.	.
.	.	.	.	.
.	.	.	.	.
.	.	.	.	.
.	.	.	.	451
.	.	.	.	.
.	.	.	.	.
.	.	.	.	490
.	.	.	.	517
.	.	.	.	.
.	.	.	.	.
.	.	.	.	.
.	.	.	.	.
.	.	.	.	.
.	.	.	.	.
.	.	.	.	.
.	.	.	.	.
.	.	.	.	.
.	.	.	.	.
.	.	.	.	360
.	.	.	.	600
.	.	.	.	493
.	.	.	.	.
.	.	.	.	.
.	.	.	.	.
.	.	.	.	301
.	.	.	.	.
.	.	.	.	.
.	.	.	.	.
.	.	.	.	.
.	.	.	.	.
.	.	.	.	.
.	.	.	.	.
.	.	.	.	.
.	.	.	.	.
.	.	.	.	.
.	.	.	.	592
.	.	.	.	.
.	.	.	.	.

410
499
583
697
466
563
477

423
491
625
539
71
398
562
400
372
542
617
530
835
551
262
505
489
405
590
521
407
528
399
537

.	.	.	.		1260
.	.	.	.		403
.	.	.	.		600
.	.	.	.		436
.	.	.	.		472
.	.	.	.	.	
.	.	.	.	.	
.	.	.	.		687

100

66 70

63	74	68	64
1	13		

51

50

44

45	46	57	58
30			
70			
25			
56	68		
73			
74			
0	0	0	0
0	0		
			100
78	50		
57			
0			
0	0		
	18		
	55		

.	27	35	57	.
.	.	.	.	.
.	.	.	.	.
.	.	.	.	.
.	.	.	.	.
.	.	.	.	.
.	.	.	.	.
.	.	.	.	.
.	0	.	.	.
.	.	.	.	.
.	.	.	.	.
.	.	.	.	.
.	40	.	.	.
.	.	.	.	.
.	.	.	.	.
.	.	.	.	.
.	78	.	.	.
.	.	.	.	.
.	.	.	.	.
.	.	.	.	.
.	100	.	.	.
.	.	.	.	.
.	.	.	.	.
.	.	.	.	.
.	.	.	.	.
.	.	.	.	.
.	.	.	.	.
.	.	.	.	.
.	.	.	.	.
.	.	.	.	.
.	.	.	.	.
.	.	.	.	.
.	.	.	.	.
.	.	.	.	.
.	.	.	.	.
.	.	.	.	.
.	.	.	.	.
.	.	72	.	.
.	.	.	.	.
.	50	50	.	.
.	41	76	.	.
.	.	.	.	.
.	87	.	.	.
.	.	0	.	.
.	.	.	.	.
.	.	.	.	.
.	.	.	.	.
.	.	20	.	.
.	.	.	.	.
.	.	.	.	.
.	.	.	.	.
.	.	0	0	.
.	.	0	.	.
.	.	0	.	.
.	.	.	.	.
.	.	.	.	.
.	.	24	.	.
.	.	47	.	.
.	.	18	.	.

	55
59	70
90	60
	72
100	
100	
	51
	92
	77
	73
	78
	50
	60

0

70

breakfast_2019_3	breakfast_2019_4	breakfast_2019_5	breakfast_2019_6	breakfast_2019_7
.	.	.	.	.
.	.	.	.	544
.	.	.	.	.
.	.	.	.	.
.	.	.	.	.
.	.	.	.	.
.	.	.	.	.
.	.	.	.	.
.	.	.	.	.
.	.	.	.	.
493	431	335	331	421
.	.	.	.	.
.	.	.	.	.
.	.	.	.	.
.	.	.	.	.
.	.	.	.	.
.	.	.	.	.
.	.	.	.	.
.	.	.	.	.
.	.	.	.	.
.	.	.	.	.
.	.	.	.	.
.	.	.	.	.
.	.	.	.	.
.	.	.	.	.
.	.	.	.	.
503	.	.	.	.
.	.	.	.	.
.	.	.	.	.
.	.	.	.	.
.	.	.	.	.
.	.	.	.	.
.	.	.	.	.
.	.	.	.	.
.	.	.	.	.
.	540	.	.	.
.	510	.	.	.
.	.	.	.	.
391	397	422	387	.
616	640	.	.	.
594	477	480	487	490
.	.	.	.	.
.	.	.	.	.
.	.	.	.	.
.	570	.	.	.
.	.	.	.	.
.	.	.	.	.
.	.	.	.	.
.	.	.	.	.
567	612	.	.	.
.	.	.	.	.
.	.	.	.	.
.	.	.	.	.
.	.	.	.	.
.	.	.	.	.
632	.	.	.	.
.	.	.	.	.
.	.	.	.	.

				529
527	502	500	592	413
495	534			
499	529	483	516	481
				482
593	665	661	690	
474	471	470	471	561
494	533	491	496	490
				474
446				

	412		409	602
489			503	
	280	475	456	
435	445	488	595	456
586	505	512	531	535
450	381	427	430	554
613	552	593	420	591
	533	463	585	577
543	514	502	531	508
529	626			593
553	534			
569				
531	461	441	451	536

.		.		.		.		.	
	407	.		.		.		.	
.		.		.		.		.	
	585	.		.		.		.	
.		.		.		.		.	
	536		514		487		527		471
.		.		.		.		.	
	534		472	.		.			437
	450	.		.		.		.	
	523	.		.		.		.	
	330	.		.		.		.	
	455		444		466		442		466
.		.		.		.		.	
	495	.		.		.		.	
.		.		.		.		.	
	800	.		.		.		.	
	438	.		.		.		.	
.			497		474		502		483
.		.		.		.		.	
.		.		.		.		.	
	600		555	.		.		.	
	570	.		.		.		.	
	480		380	.		.		.	
.			428	.		.		.	
.			653	.		.		.	
.			368	.		.		.	
.			427	.		.		.	
.		.		.		.		.	
.			570	.		.		.	
.			397	.		.		.	
.		.			423	.		.	
.		.		.		.		.	
.			450	.		.		.	
.			478		508	.		.	
.			450	.		.		.	
.		.		.		.		.	
.			375	.		.		.	
.			558	.		.		.	
.			725	.		.		.	
.		.		.		.		.	
.			595	.		.		.	
.			490	.		.		.	
.			295	.		.		.	
.			913	.		.		.	
.			600	.		.		.	
.			768		707	.		.	
.		.		.		.		.	
.			1205	.		.		.	
.		.			340	.		.	
.		.		.		.		.	
.		.			484		457	.	
.		.			482	.		.	
.		.			406		321		367
.		.		.		.		.	
.		.			430	.		.	
.		.			570		540		430
.		.			500	.		.	
.		.			450		492		532
.		.			497	.		.	

.	.	.	.	.
.	.	.	495	.
.	.	.	578	660
.	.	.	600	.
.	.	.	540	.
.	.	.	.	.
.	.	.	435	.
.	.	.	.	.
.	.	.	.	.
.	.	.	497	.
.	.	.	548	.
.	.	.	455	.
.	.	.	360	408
.	.	.	.	615
.	.	.	.	.
.	.	.	.	494
.	.	.	.	.
.	.	.	.	.
.	.	.	.	451
.	.	.	.	606
.	.	.	.	410
.	.	.	.	.
.	.	.	.	.
.	.	.	.	.
.	.	.	.	.
.	.	.	.	587
.	.	.	.	1257
.	.	.	.	511
.	.	.	.	.
.	.	.	.	.
.	.	.	.	.
.	.	.	.	.
.	.	.	.	.
.	.	.	.	.
.	.	.	.	.
.	.	.	.	.
.	.	.	.	.
.	.	.	.	.
.	.	.	.	.
.	.	.	.	.
.	.	.	.	.
.	.	.	.	.
.	.	.	.	.
.	.	.	.	.
.	.	.	.	.
.	.	.	.	.
.	.	.	.	.
.	.	.	.	.
.	.	.	.	.
.	.	.	.	.
.	.	.	.	.
.	.	.	.	.
.	.	.	.	.
.	.	.	.	.
.	.	.	.	.
.	.	.	.	.
.	.	.	.	.

.	.	.	.	.
.	.	.	.	.
.	.	.	.	.
.	.	.	.	.
.	.	.	.	.
.	.	.	.	.
.	.	.	.	.
.	.	.	.	.
.	.	.	.	.
.	.	.	.	.
.	.	.	.	.
.	.	.	.	.
.	.	.	.	.
.	.	.	.	.
.	.	.	.	.
.	.	.	.	.
.	.	.	.	.
.	.	.	.	.
.	.	.	.	.
.	.	.	.	.
.	.	.	.	.
.	.	.	.	.
.	.	.	.	.
.	.	.	.	.
.	.	.	.	.

breakfast_2019_8	breakfast_2019_9	breakfast_2019_10	breakfast_2019_11	breakfast_2019_12
.	.	.	.	.
.	480	485	.	.
.	.	.	.	.
.	.	.	.	944
.	.	.	.	.
.	.	.	.	.
.	.	.	.	.
.	.	.	.	.
.	.	.	.	.
.	.	.	.	.
.	.	.	.	.
.	.	.	.	.
.	.	.	.	.
.	.	.	.	.
.	.	.	.	.
.	.	.	.	.
.	.	.	.	.
.	.	.	.	.
.	.	.	.	.
.	.	.	.	.
.	.	.	.	.
.	.	.	.	.
.	.	.	.	.
.	.	.	.	.
.	.	.	.	.
.	.	.	.	.
.	.	.	.	.
.	.	.	.	.
.	.	.	.	.
.	.	.	.	.
.	.	.	.	.
.	.	.	.	.
.	.	.	.	.
.	.	.	.	.
.	.	.	.	.
.	.	.	.	.
.	.	.	.	.
.	.	.	.	.
398	372	360	385	360
619	608	610	589	629
483	509	383	439	451
.	.	.	.	.
.	.	.	.	.
.	.	.	.	.
.	.	.	.	484
.	.	.	.	.
.	.	.	.	.
.	.	.	410	.
.	.	.	.	.
.	539	591	518	.
.	.	.	.	.
.	.	.	.	.
.	.	.	.	.
.	556	.	.	.
.	.	.	.	.
.	.	.	.	.
.	.	.	.	.
.	.	.	.	.

455	484			
506	484	472	479	509
	474			
		450		
476	481	489	479	477
519	538	503	748	713
	475	482		
	531			
			415	

352		355	352	324
561	470	471	450	565
435			474	485
527	504	531	495	476
	524			
	611			
503	391	402	477	396
	538		583	
497	632	581		
	524	537	587	493
		28		
511	498	495	509	502
600	428	437	404	
564	614			
583				
467	444	457	462	524

486	448	444	440	467
465	473	500	490	462
			480	
900	491			
479	522			

546	538	516	516	550
550	446	432	502	542
567	564	537		551
420				
455				
570				
510	585	580	679	548
490				
482				
510				
549				
448				
377				
640				
509				
381				
	512		505	493
	471	505	474	497
	486			
	512			
	411			
	495			
	457	444	441	459
	555			

.	.	.	.	.
.	.	.	.	.
.	458	513	.	.
.	.	.	.	.
.	465	.	.	.
.	540	.	.	.
.	404	.	.	.
.	447	388	391	357
.	600	.	.	.
.	495	420	.	.
.	445	406	368	385
.	540	550	.	.
.	450	.	.	.
.	510	.	.	.
.	960	.	.	.
.	.	986	.	.
.	.	440	.	.
.	.	599	.	448
.	.	465	.	.
.	.	.	.	.
.	.	530	.	.
.	.	435	345	.
.	.	.	.	.
.	.	.	516	.
.	.	.	.	.
.	.	530	.	.
.	.	595	.	.
.	.	.	.	.
.	.	470	426	.
.	.	420	.	.
.	.	.	.	.
.	.	.	.	.
.	.	.	473	.
.	.	.	.	.
.	.	.	473	.
.	.	.	730	.
.	.	.	456	409
.	.	.	555	.
.	.	.	.	.
.	.	.	351	366
.	.	.	.	.
.	.	.	420	.
.	.	.	510	.
.	.	.	639	.
.	.	.	.	.
.	.	.	630	646
.	.	.	0	.
.	.	.	495	390
.	.	.	1337	.
.	.	.	585	.
.	.	.	.	504
.	.	.	.	690
.	.	.	.	.
.	.	.	.	367
.	.	.	.	356
.	.	.	.	.
.	.	.	.	.
.	.	.	.	360
.	.	.	.	485

.	.	.	.	.	
.	.	.	.	.	
.	.	.	.		450
.	.	.	.		630
.	.	.	.		504
.	.	.	.		450
.	.	.	.	.	
.	.	.	.		510
.	.	.	.		510
.	.	.	.	.	
.	.	.	.		552
.	.	.	.	.	
.	.	.	.	.	
.	.	.	.		542
.	.	.	.	.	
.	.	.	.	.	
.	.	.	.	.	
.	.	.	.	.	
.	.	.	.	.	
.	.	.	.	.	
.	.	.	.	.	
.	.	.	.	.	
.	.	.	.	.	
.	.	.	.	.	
.	.	.	.	.	
.	.	.	.	.	
.	.	.	.	.	
.	.	.	.	.	
.	.	.	.	.	
.	.	.	.	.	
.	.	.	.	.	
.	.	.	.	.	
.	.	.	.	.	
.	.	.	.	.	
.	.	.	.	.	
.	.	.	.	.	
.	.	.	.	.	
.	.	.	.	.	
.	.	.	.	.	
.	.	.	.	.	
.	.	.	.	.	
.	.	.	.	.	
.	.	.	.	.	
.	.	.	.	.	
.	.	.	.	.	
.	.	.	.	.	
.	.	.	.	.	
.	.	.	.	.	
.	.	.	.	.	
.	.	.	.	.	
.	.	.	.	.	
.	.	.	.	.	
.	.	.	.	.	
.	.	.	.	.	
.	.	.	.	.	
.	.	.	.	.	
.	.	.	.	.	
.	.	.	.	.	
.	.	.	.	.	

breakfast_2019_13	breakfast_2019_14	breakfast_2019_15	breakfast_2019_16	breakfast_2019_17
.	475	.	.	.
.	.	485	.	.
.	.	.	.	.
.	.	.	.	.
.	.	.	.	.
.	.	.	.	.
.	.	.	.	.
.	.	.	.	.
.	.	.	.	.
.	.	.	.	.
.	.	.	.	.
.	.	.	.	.
.	.	.	.	.
.	.	.	.	.
.	.	.	.	.
.	.	.	.	.
.	.	.	.	.
.	.	.	.	.
.	.	.	.	.
.	.	.	.	.
.	.	.	.	.
.	.	.	.	.
.	.	.	.	.
.	.	.	.	.
.	.	.	.	.
.	.	.	.	.
.	.	.	.	.
.	.	.	.	.
.	.	.	.	.
.	.	.	.	.
.	.	.	.	.
.	.	.	.	.
.	.	.	.	.
.	.	.	.	.
.	.	.	.	.
.	.	.	.	.
.	.	.	.	.
.	.	.	.	.
397	.	431	380	379
.	589	.	596	.
424	472	464	439	432
.	.	.	.	.
.	.	.	.	.
.	.	.	.	.
477	464	419	433	426
.	.	.	.	.
.	.	.	.	.
.	.	.	.	.
.	.	.	.	.
.	.	.	.	.
.	.	.	.	.
.	.	.	.	.
.	.	.	.	.
.	.	.	.	.
.	.	.	.	.
.	.	.	.	.
.	.	.	.	.
.	.	.	.	.

501	472	487	524	466
457	465	479	467	475
685				
			0	

			645	
436	377		393	
388				
551	518	506		
391	490			350
606	521	522	613	566
518				
493	342	433	372	502
		402		
507	513	511	508	500
			627	521
489	475	442	458	494

456

441	468	496	448	469

526	639	525	511	541
511				
1025				735
597	650	650		
468	511	480	522	450
431	476	469	463	457

395	360			
419	384			
499				
426	452	484	477	462
380	360			
470	580	527	495	522
			715	627
		396		

.		.		.		.		.	
.		.		.		.		.	
.		.		.		.		.	
.		.		.		.		.	
	448	.		.		.		.	
	429	.		.		.		.	
.		.		.		.		.	
	630		575	.			570		645
.		.		.		.		.	
.		.		.		.		.	
.		.		.		.		.	
.		.		.		.		.	
	1080	.		.		.		.	
.		.		.		.		.	
.		.		.		.		.	
.		.		.		.		.	
.		.		.		.		.	
.		.		.		.		.	
.		.		.		.		.	
.		.		.		.		.	
	448		425		493		472		415
	480	.		.		.		.	
	501	.		.		.		.	
	481		228	.		.		.	
	447	.		.		.		.	
.		.		.		.		.	
	510	.		.		.		.	
.			440	.		.		.	
	1348	.		.		.		.	
.			457		418		433		401
	573		447	.		.		.	
	608		360	.		.		.	
.			436	.		.		.	
	1115	.		.		.		.	
	470	.		.		.		.	
.		.		.		.		.	
.			451	.		.		.	
	510		473	.		.		.	
.		.		.		.		.	
.			316	.		.		.	
.			300	.		.		.	
.		.		.		.		.	
.		.		.		.		.	
.			432		427	.		.	
.			434	.		.		.	
.		.		.		.		.	
.		.		.		.		.	
.		.		.		.		.	
.			375	.		.		.	
.			636	.		.		.	
.			561	.		.		.	
.		.		.		.		.	
.		.		.		.		.	
.		.		.		.		.	
.		.		.		.		.	
.		.		.		.		.	
.		.		.		.		.	
.			498		456		476		459
.		.			404	.		.	

.	.	.	.	.
.	.	400	.	.
.	.	480	.	.
.	.	553	558	.
.	.	1398	.	.
.	.	601	.	.
.	.	.	.	.
.	.	405	.	.
.	.	540	.	.
.	.	.	.	.
.	.	768	752	774
.	.	444	.	.
.	.	.	515	.
.	.	450	.	.
.	.	432	.	.
.	.	659	.	.
.	.	.	.	.
.	.	.	.	.
.	.	.	343	.
.	.	.	477	448
.	.	.	343	.
.	.	.	481	.
.	.	.	512	.
.	.	.	.	.
.	.	.	1242	.
.	.	.	.	.
.	.	.	399	.
.	.	.	.	.
.	.	1409	450	.
.	.	.	.	0
.	.	.	883	.
.	.	.	.	.
.	.	.	.	.
.	.	.	441	.
.	.	.	620	.
.	.	.	465	.
.	.	.	.	.
.	.	.	.	462
.	.	.	431	.
.	.	.	.	.
.	.	.	.	.
.	.	.	.	0
.	.	.	.	563
.	.	.	.	510
.	.	.	.	540
.	.	.	.	570
.	.	.	.	.
.	.	.	.	.
.	.	.	.	.
.	.	.	.	.
.	.	.	.	.
.	.	.	.	691
.	.	.	.	.
.	.	.	.	.
.	.	.	.	.
.	.	.	.	.
.	.	.	.	.
.	.	.	.	.
.	.	.	.	.

.	.	.	.	.
.	.	.	.	.
.	.	.	.	.
.	.	.	.	.
.	.	.	.	.
.	.	.	.	.
.	.	.	.	.
.	.	.	.	.
.	.	.	.	.
.	.	.	.	.
.	.	.	.	.
.	.	.	.	.
.	.	.	.	.
.	.	.	.	.
.	.	.	.	.
.	.	.	.	.
.	.	.	.	.
.	.	.	.	.
.	.	.	.	.
.	.	.	.	.
.	.	.	.	.
.	.	.	.	.
.	.	.	.	.
.	.	.	.	.
.	.	.	.	.
.	.	.	.	.
.	.	.	.	.
.	.	.	.	.
.	.	.	.	.
.	.	.	.	.
.	.	.	.	.
.	.	.	.	.
.	.	.	.	.
.	.	.	.	.
.	.	.	.	.
.	.	.	.	.
.	.	.	.	.
.	.	.	.	.
.	.	.	.	.
.	.	.	.	.
.	.	.	.	.
.	.	.	.	.
.	.	.	.	.
.	.	.	.	.
.	.	.	.	.
.	.	.	.	.
.	.	.	.	.
.	.	.	.	.
.	.	.	.	.
.	.	.	.	.
.	.	.	.	.
.	.	.	.	.
.	.	.	.	.
.	.	.	.	.
.	.	.	.	.
.	.	.	.	.
.	.	.	.	.
.	.	.	.	.
.	.	.	.	.

breakfast_2020_2	breakfast_2020_3	breakfast_2020_4	breakfast_2020_5	breakfast_2020_6
.	.	.	.	.
.	.	.	.	.
.	.	.	.	.
.	.	.	.	.
.	.	.	.	.
.	.	.	.	.
.	.	.	.	.
.	.	.	.	.
.	.	.	.	.
.	.	.	.	.
.	.	.	.	.
.	.	.	.	.
.	.	.	.	.
.	.	.	.	.
.	.	.	.	.
.	.	.	.	.
.	.	.	.	.
.	.	.	.	.
.	.	.	.	.
.	.	.	.	.
.	.	.	.	.
.	.	.	.	.
.	.	.	.	.
.	.	.	.	.
.	.	.	.	.
.	.	.	.	.
.	.	.	.	.
.	.	.	.	.
.	.	.	.	.
.	.	.	.	.
.	.	.	.	.
.	.	.	.	.
.	.	.	.	.
.	.	.	.	.
.	.	.	.	.
.	.	.	.	.
.	.	.	.	.
.	.	.	.	.
.	.	400	.	.
.	.	.	.	.
.	468	472	455	.
.	.	.	.	.
.	.	.	.	.
.	.	.	.	.
.	.	.	.	.
.	.	.	.	.
.	.	.	.	.
.	.	.	.	.
.	.	.	.	.
.	.	.	.	.
.	.	.	.	.
.	.	.	.	.
.	.	.	.	.
.	.	.	.	.
.	.	.	.	.
.	.	.	.	.
.	.	.	.	.
.	.	.	.	.

478 451 481 456 491

600

447	458			
468	747<br>447	440	466	464
525	513	523	533	477

0

492	598	495	539	629

450	414	419	401	427

.		.		.		.		.	
.		.		.		.		.	
	626		466		590	.			627
.		.		.			540	.	
.		.		.		.		.	
.		.		.		.		.	
	457	.		.		.		.	
	611		612		616		551		693
.		.		.		.		.	
.		.		.		.		.	
.		.		.		.		.	
.		.		.		.		.	
	464	.		.		.		.	
.		.		.		.		.	
.		.		.		.		.	
.		.		.		.		.	
.		.		.		.		.	
.		.		.		.		.	
.		.		.		.		.	
.		.		.		.		.	
.		.		.		.		.	
.		.		.		.		.	
.		.		.		.		.	
.		.		.		.		.	
	164	.		.		.		.	
.		.		.		.		.	
.		.		.		.		.	
.		.		.		.		.	
.		.		.		.		.	
	449		452		438		444		450
.		.		.		.		.	
.		.		.		.		.	
.		.		.		.		.	
	434		432		439		432		428
.		.		.		.		.	
	415		415		394	.			426
	540		572		504	.		.	
	413	.		.		.		.	
.		.		.		.		.	
.		.			454	.		.	
	446		456		431		396		391
	739		647	.		.			
	454		461		454		457		458
	462		481		468		570		466
	473		459		468	.		.	
.		.		.		.		.	
.		.			0	.		.	
.		.		.		.		.	
	388	.		.		.		.	
	774	.		.		.		.	
	629	.		.		.		.	
	458	.		.		.		.	
.		.		.			629	.	
.		.		.		.		.	
	714		490	.		.		.	
.		.		.		.		.	
.		.		.			505	.	
	536	.		.		.		.	
.		.		.		.		.	

405	.	.	.	.
620	.	.	.	.
517	.	.	.	.
.	430	.	.	.
440	.	.	.	.
625	594	611	599	650
477	.	.	.	.
630	.	.	.	.
520	.	.	.	.
.	.	.	.	.
453	.	.	.	.
483	518	.	.	.
675	.	.	.	.
.	.	.	.	.
.	.	.	.	.
.	.	.	.	.
.	.	.	.	.
510	447	.	.	.
.	.	.	.	.
612	422	.	.	.
0	.	.	.	.
480	532	.	.	.
502	.	.	.	.
.	.	.	.	.
.	.	.	.	.
481	.	.	.	.
.	.	.	.	.
.	.	.	.	.
388	.	.	.	.
618	.	.	.	.
.	.	.	.	.
455	499	.	.	.
404	540	530	624	542
.	.	.	.	.
.	.	.	.	.
685	.	.	.	.
.	.	.	.	.
591	.	.	.	.
.	690	.	.	.
.	.	.	.	.
.	.	.	.	.
.	737	.	.	.
540	581	.	.	.
.	570	.	.	.
.	390	.	.	.
.	.	.	.	.
.	480	.	.	.
.	.	.	.	.
.	.	.	.	.
.	379	.	.	.
.	.	.	.	.
.	.	.	.	.
.	711	.	.	.
.	291	.	.	.
.	390	.	.	.
.	467	412	.	.
.	466	.	.	.
.	.	.	.	.
.	.	.	.	.

.	1174	.	.	.
.	209	303	342	.
.	.	.	756	.
.	.	.	.	.
.	.	.	.	.
.	450	.	.	.
.	537	.	.	.
.	591	.	.	.
.	508	.	.	.
.	.	.	.	.
.	.	.	.	.
.	.	.	.	.
.	417	.	.	.
.	.	.	.	.
.	.	.	.	.
.	.	.	.	.
.	.	.	.	.
.	767	607	.	.
.	859	971	.	840
.	449	297	0	0
.	345	389	400	.
.	.	478	421	390
.	570	.	.	.
.	.	646	.	.
.	.	.	.	.
.	.	523	553	.
.	.	.	.	.
.	.	.	.	.
.	.	.	.	.
.	.	450	.	.
.	.	450	.	.
.	.	.	.	.
.	.	500	535	.
.	.	420	.	.
.	.	.	.	.
.	.	.	.	.
.	.	.	635	.
.	.	506	.	.
.	.	476	512	.
.	.	.	.	.
.	.	.	.	.
.	.	.	.	.
.	.	602	626	.
.	.	571	.	.
.	.	443	297	349
.	.	.	.	.
.	.	.	.	.
.	.	361	.	.
.	.	511	495	.
.	.	.	.	.
.	.	.	517	.
.	.	.	.	.
.	.	780	.	.
.	.	405	424	443
.	.	.	.	.
.	.	661	.	.
.	.	.	630	.
.	.	.	.	.
.	.	.	354	.

.	.	.	122	.
.	.	.	.	.
.	.	.	474	465
.	.	.	491	.
.	.	.	.	.
.	.	.	455	.
.	.	.	455	.
.	.	.	455	.
.	.	.	361	.
.	.	.	.	.
.	.	.	.	540
.	.	.	.	.
.	.	.	.	.
.	.	.	451	.
.	.	.	200	.
.	.	502	0	.
.	.	.	492	.
.	.	.	485	.
.	.	.	391	.
.	.	.	461	483
.	.	.	518	437
.	.	.	.	.
.	.	.	742	.
.	.	.	495	.
.	.	.	570	.
.	.	.	597	587
.	.	.	615	.
.	.	.	.	.
.	.	.	.	.
.	.	.	.	.
.	.	.	.	.
.	.	.	650	.
.	.	.	.	.
.	.	.	.	460
.	.	.	.	.
.	.	.	.	.
.	.	.	.	.
.	.	.	.	.
.	.	.	.	.
.	.	.	660	.
.	.	.	.	527
.	.	.	.	510
.	.	.	.	.
.	.	.	.	350
.	.	.	.	.
.	.	.	.	1203
.	.	.	.	720
.	.	.	.	623
.	.	.	.	346
.	.	.	.	.
.	.	.	.	539
.	.	.	.	.
.	.	.	.	.
.	.	.	.	.
.	.	.	.	.
.	.	.	.	.
.	.	.	.	500
.	.	.	.	.
.	.	.	.	.

.	.	.	.		390
.	.	.	.		479
.	.	.	.		671
.	.	.	.	.	
.	.	.	.	.	
.	.	.	.		510
.	.	.	.		510
.	.	.	.		540
.	.	.	.	.	
.	.	.	.	.	
.	.	.	.		822
.	.	.	.		501
.	.	.	.	.	
.	.	.	.		510
.	.	.	.	.	
.	.	.	.	.	
.	.	.	.	.	
.	.	.	.	.	
.	.	.	.	.	
.	.	467	.	.	
.	.	.	.	.	
.	.	.	.	.	
.	.	.	.	.	
.	.	.	.	.	
.	.	.	.	.	
.	.	.	.	.	
.	.	.	.	.	
.	.	.	.	.	
.	.	.	.	.	
.	.	.	.	.	
.	.	.	.	.	
.	.	.	.	.	
.	.	.	.	.	
.	.	.	.	.	
.	.	.	.	.	
.	.	.	.	.	
.	.	.	.	.	
.	.	.	.	.	
.	.	.	.	.	
.	.	.	.	.	
.	.	.	.	.	
.	.	.	.	.	
.	.	.	.	.	
.	.	.	.	.	
.	.	.	.	.	
.	.	.	.	.	
.	.	.	.	.	
.	.	.	.	.	
.	.	.	.	.	
.	.	.	.	.	
.	.	.	.	.	
.	.	.	.	.	
.	.	.	.	.	
.	.	.	.	.	
.	.	.	.	.	
.	.	.	.	.	
.	.	.	.	.	
.	.	.	.	.	
.	.	.	.	.	

.	.	.	.	.
.	.	.	.	.
.	.	.	.	.
.	.	.	.	.
.	.	.	.	.
.	.	.	.	.
.	.	.	.	.
.	.	.	.	.
.	.	.	.	.
.	.	.	.	.
.	.	.	.	.
.	.	.	.	.
.	.	.	.	.
.	.	.	.	.
.	.	.	.	.
.	.	.	.	.
.	.	.	.	.
.	.	.	.	.
.	.	.	.	.
.	.	.	.	.
.	.	.	.	.
.	.	.	.	.
.	.	.	.	.
.	.	.	.	.
.	.	.	.	.
.	.	.	.	.
.	.	.	.	.
.	.	.	.	.
.	.	.	.	.
.	.	.	.	.
.	.	.	.	.
.	.	.	.	.
.	.	.	.	.
.	.	.	.	.
.	.	.	.	.
.	.	.	.	.
.	.	.	.	.
.	.	.	.	.
.	.	.	.	.
.	.	.	.	.
.	.	.	.	.
.	.	.	.	.
.	.	.	.	.
.	.	.	.	.
.	.	.	.	.
.	.	.	.	.
.	.	.	.	.
.	.	.	.	.
.	.	.	.	.
.	.	.	.	.
.	.	.	.	.
.	.	.	.	.
.	.	.	.	.
.	.	.	.	.
.	.	.	.	.
.	.	.	.	.
.	.	.	.	.
.	.	.	.	.
.	.	.	.	.

breakfast_2020_7	breakfast_2020_8	breakfast_2020_9	breakfast_2020_10	breakfast_2020_11
.	.	.	.	.
.	.	.	.	.
.	691	.	.	.
.	.	.	.	.
.	.	.	.	.
.	.	.	.	.
.	.	.	.	.
.	.	.	.	.
.	.	.	.	.
.	.	.	.	.
.	.	.	.	.
.	.	.	.	.
.	.	.	.	.
.	.	.	.	.
.	.	.	.	.
.	.	.	.	.
.	.	.	.	.
.	.	.	.	.
.	.	.	.	.
.	.	.	.	.
.	.	.	.	.
.	.	.	.	.
.	.	.	.	.
.	.	.	.	.
.	.	.	.	.
.	.	.	.	.
.	.	.	.	.
.	.	.	.	.
.	.	.	.	.
.	.	.	.	.
.	.	.	.	.
.	.	.	.	.
.	.	.	.	.
.	.	.	.	.
.	.	.	.	.
.	.	.	.	.
.	.	.	.	.
.	.	.	.	.
.	465	423	.	.
.	.	.	.	.
463	459	455	.	.
.	.	.	.	.
.	.	.	.	.
.	.	.	.	.
.	.	.	.	.
.	.	.	.	.
.	.	.	.	465
.	.	.	.	.
.	.	.	.	.
.	.	.	.	.
.	.	.	.	.
.	.	.	.	.
.	.	.	.	.
.	.	.	.	.
.	.	.	.	.
.	.	.	.	.
.	.	.	.	.
.	.	.	.	.

456 504

462 478 447 454 448

		327		303
402	497	391	378	426
478	534	529	500	485

607	566	581	550	561

388	406	401	385	353

545	582			
665	569	619	659	604
		454		
		415		
				461
452	448	444	435	423
422				
400	430	420	411	399
510				
466	458	456		
463			528	475
			477	

679	609	656	

807	778	805		
0	0	76	0	0
475				

	490	481		
484	514	492	479	477
		560		
365				

.	.	.	.	.
.	.	.	.	.
620	619	.	.	.
.	.	.	.	.
.	.	.	.	.
.	.	.	.	.
.	.	.	.	.
.	.	.	.	.
.	.	.	.	.
.	.	.	.	.
454	.	.	.	.
.	.	.	.	.
.	.	.	.	.
510	.	.	.	.
.	.	.	.	.
795	.	.	.	.
.	.	.	.	.
468	.	.	.	.
0	.	.	.	.
450	468	439	.	.
465	.	.	.	.
558	553	.	.	.
.	.	.	.	.
820	.	.	.	.
.	.	.	.	.
.	.	.	.	.
557	.	.	.	.
.	.	.	.	.
.	.	.	.	.
.	.	.	.	.
511	.	.	.	.
.	.	.	.	.
450	.	.	.	.
.	.	.	.	.
.	543	.	.	.
510	.	.	.	.
676	557	593	659	628
420	.	.	.	.
.	.	.	.	.
.	483	.	.	.
.	.	.	.	.
.	630	.	.	.
.	.	.	.	.
.	483	.	.	.
.	600	.	.	.
.	548	.	.	.
.	352	484	.	.
.	.	.	.	.
.	517	473	.	.
.	635	.	.	.
.	457	460	465	.
.	.	.	.	.
.	582	569	.	.
.	420	.	.	.
.	480	.	.	.
.	310	.	.	.
.	600	.	.	.
.	.	444	.	.
.	450	.	.	.

.	.	.	.	.
.	.	421	500	332
.	.	.	.	.
.	556	517	656	619
.	.	.	.	.
.	.	421	.	.
.	.	514	.	578
.	.	.	.	.
.	.	630	.	.
.	.	505	.	.
.	.	.	.	.
.	.	481	.	.
.	.	.	540	.
.	.	.	.	.
.	.	.	488	.
.	.	610	.	.
.	.	612	707	639
.	.	354	365	.
.	.	.	.	.
.	.	557	.	.
.	.	.	.	.
.	.	467	467	462
.	.	461	.	.
.	.	.	.	.
.	.	966	.	.
.	.	.	.	.
.	.	432	521	.
.	.	629	.	.
.	.	540	525	.
.	.	.	426	.
.	.	.	478	458
.	.	.	.	.
.	.	.	666	613
.	.	.	366	.
.	.	.	530	.
.	.	.	.	.
.	.	.	451	.
.	.	.	420	.
.	.	.	541	497
.	.	.	.	.
.	.	.	632	.
.	.	.	.	.
.	.	.	828	.
.	.	.	413	570
.	.	.	1314	.
.	.	.	501	.
.	.	.	.	.
.	.	.	620	570
.	.	.	.	.
.	.	.	481	568
.	.	.	631	.
.	.	.	.	.
.	.	.	548	497
.	.	.	.	.
.	.	.	.	508
.	.	.	.	.
.	.	.	1362	340
.	.	.	.	.
.	.	.	.	473

.	.	.	.	.	
.	.	.	.	.	
.	.	.	.	.	
.	.	.	.	.	
.	.	.	.		473
.	.	.	.		587
.	.	.	.	.	
.	.	.	.		558
.	.	.	.		480
.	.	.	.	.	
.	.	.	.		600
.	.	.	.	.	
.	.	.	.	.	
.	.	.	.	.	
.	.	.	.		360
.	.	.	.		580
.	.	.	.		532
.	.	.	.		540
.	.	.	.		319
.	.	.	.		520
.	.	.	.	.	
.	.	.	.	.	
.	.	.	.		508
.	.	.	.	.	
.	.	.	.		1365
.	.	.	.	.	
.	.	.	.		616
.	.	.	.		618
.	.	.	.	.	
.	.	.	.	.	
.	.	.	.	.	
.	.	.	.	.	
.	.	.	.	.	
.	.	.	.	.	
.	.	.	.	.	
.	.	.	.	.	
.	.	.	.	.	
.	.	.	.	.	
.	.	.	.	.	
.	.	.	.	.	
.	.	.	.	.	
.	.	.	.	.	
.	.	.	.	.	
.	.	.	.	.	
.	.	.	.	.	
.	.	.	.	.	
.	.	.	.	.	
.	.	.	.	.	
.	.	.	.	.	
.	.	.	.	.	
.	.	.	.	.	
.	.	.	.	.	
.	.	.	.	.	
.	.	.	.	.	
.	.	.	.	.	
.	.	.	.	.	
.	.	.	.	.	

.	.	.	.	.
.	.	.	.	.
.	.	.	.	.
.	.	.	.	.
.	.	.	.	.
.	.	.	.	.
.	.	.	.	.
.	.	.	.	.
.	.	.	.	.
.	.	.	.	.
.	.	.	.	.
.	.	.	.	.
.	.	.	.	.
.	.	.	.	.
.	.	.	.	.
.	.	.	.	.
.	.	.	.	.
.	.	.	.	.
.	.	.	.	.
.	.	.	.	.
.	.	.	.	.
.	.	.	.	.
.	.	.	.	.
.	.	.	.	.
.	.	.	.	.

breakfast_2020_12	breakfast_2020_13	breakfast_2020_14	breakfast_2020_15	breakfast_2020_16
.	.	.	.	.
.	.	.	.	.
.	.	.	.	.
.	.	.	.	.
.	.	.	.	.
.	.	.	.	.
.	.	.	.	.
.	.	.	.	.
.	.	.	.	.
.	.	.	.	.
.	.	.	.	.
.	.	.	.	.
.	.	.	.	.
.	.	.	.	.
.	.	.	.	.
.	.	.	.	.
.	.	.	.	.
.	.	.	.	.
.	.	.	.	.
.	.	.	.	.
.	.	.	.	.
.	.	.	.	.
.	.	.	.	.
.	.	.	.	.
.	.	.	.	.
.	.	.	.	.
.	.	.	.	.
.	.	.	.	.
.	.	.	.	.
.	.	.	.	.
.	.	.	.	.
.	.	.	.	.
.	.	.	.	.
.	.	.	.	.
.	.	.	.	.
.	.	.	.	.
.	.	.	.	.
.	.	.	.	.
.	.	.	.	.
.	.	.	.	.
458	408	.	477	.
.	.	.	.	.
.	.	.	.	.
.	.	.	.	.
.	.	.	.	.
.	.	.	.	.
.	.	.	.	.
.	.	.	.	.
.	.	.	.	.
.	.	.	.	.
.	.	.	.	.
.	.	.	.	.
.	.	.	.	.
.	.	.	.	.
.	.	.	.	.
.	.	.	.	.
.	.	.	.	.
.	.	.	.	.

448
451
455
443
447
603

		457	460	469
415	404	458	397	400
488	502	519	482	493

553	542	613	569	575

418
411
398
366
366
379

650	553	515	582	656
				370
430				
393				
498	466	464		
				500

806

473 481 475 477

	627	629		
604	603	513	534	676

405	352	369	398	448
460	548			
423	270	276		
				647
459	496	502	497	496
596		532	695	658
571	590	554	555	558
503				

.		.		.		.		.	
.		.		.		.		.	
.		.		.		.		.	
.		.		.		.		.	
	493		452		440		473	.	
.		.		.		.		.	
.		.		.		.		.	
	543		556		545	.		.	
	460	.		.		.		.	
.			390	.		.		.	
.		.		.		.		.	
.		.		.		.		.	
.		.		.		.		.	
.		.		.		.		.	
.		.		.		.		.	
.		.		.		.		.	
	453		470		514	.		.	
.		.		.		.		.	
	365		360		446		335		507
	432		698	.		.		.	
	615	.		.		.		.	
.		.		.		.		.	
.		.		.		.		.	
.		.		.		.		.	
.		.		.		.		.	
.		.		.		.		.	
.		.		.		.		.	
.		.		.		.		.	
.		.		.		.		.	
.		.		.		.		.	
	614	.		.		.		.	
.		.		.		.		.	
	515	.		.		.		.	
.		.		.		.		.	
	684	.		.		.		.	
.		.		.		.		.	
.		.		.		.		.	
.		.		.		.		.	
	600	.		.		.		.	
	340		356	.		.		.	
	286	.		.		.		.	
.		.		.		.		.	
.		.		.		.		.	
.		.		.		.		.	
	447		431	.		.		.	
	674		550	.		.		.	
	430	.		.		.		.	
	428	.		.		.		.	
	898		276	.		.		.	
.		.		.		.		.	
.			592	.		.		.	
.		.		.		.		.	
.			600	.		.		.	
.			509	.		.		.	
.			271	.		.		.	
.			395	.		.		.	
.		.		.		.		.	
.			0	.		.		.	
.			503	.		.		.	

.	810	.	.	.
.	507	443	781	752
.	497	.	.	
.	472	508	.	
.	490	.	.	
.	.	.	.	
.	660	.	.	
.	523	.	.	
.	500	.	.	
.	510	.	.	
.	501	.	.	
.	457	596	.	
.	.	.	.	
.	360	.	.	
.	530	361	.	
.	.	.	.	
.	.	.	.	
.	.	419	.	
.	.	534	.	
.	.	348	.	
.	.	574	.	
.	.	435	.	
.	.	.	.	
.	.	629	.	
.	.	639	670	662
.	.	390	.	
.	.	.	.	
.	.	570	.	
.	.	585	560	.
.	.	507	.	
.	.	.	.	
.	.	.	.	
.	.	810	.	
.	.	.	754	.
.	.	.	.	
.	.	306	.	
.	.	534	.	
.	.	.	.	
.	.	483	498	529
.	.	379	543	605
.	.	.	.	
.	.	.	643	.
.	.	420	480	.
.	.	.	.	
.	.	.	.	
.	.	.	1080	.
.	.	.	460	.
.	.	.	570	.
.	.	657	.	517
.	.	.	0	.
.	.	.	570	.
.	.	.	435	.
.	.	.	402	.
.	.	.	.	
.	.	.	.	
.	.	.	734	.
.	.	.	510	.
.	.	.	420	.
.	.	.	566	.

.	.	.	559	584
.	.	.	574	.
.	.	.	.	.
.	.	.	430	.
.	.	.	.	.
.	.	.	.	.
.	.	.	.	.
.	.	.	390	.
.	.	.	510	.
.	.	.	0	.
.	.	.	267	.
.	.	.	509	482
.	.	.	484	.
.	.	.	.	.
.	.	.	.	.
.	.	.	.	.
.	.	.	.	.
.	.	.	575	.
.	.	.	561	.
.	.	.	.	.
.	.	.	788	.
.	.	.	.	.
.	.	.	.	.
.	.	.	1048	.
.	.	.	.	.
.	.	.	.	.
.	.	.	733	623
.	.	.	486	600
.	.	.	555	.
.	.	.	614	516
.	.	.	510	.
.	.	.	.	.
.	.	.	.	.
.	.	.	390	.
.	.	.	450	.
.	.	618	510	.
.	.	.	346	.
.	.	.	.	484
.	.	.	598	533
.	.	.	.	497
.	.	.	.	430
.	.	.	.	515
.	.	.	0	462
.	.	.	420	497
.	.	.	.	580
.	.	.	.	710
.	.	.	.	675
.	.	.	.	511
.	.	.	.	.
.	.	.	.	.
.	.	.	.	1040
.	.	.	.	580
.	.	.	480	481
.	.	.	.	460
.	.	.	.	467
.	.	.	.	.
.	.	.	.	582
.	.	.	.	460
.	.	.	444	545

.	.	.	.		446
.	.	.	.		526
.	.	.	.	.	
.	.	.	.		527
.	.	.	.	.	
.	.	.	.	.	
.	.	.	.	.	
.	.	.	.		601
.	.	.	.		562
.	.	.	.	.	
.	.	.	.		620
.	.	.	.	.	
.	.	.	.		615
.	.	.	.		480
.	.	.	.	.	
.	.	.	.		584
.	.	.	.		480
.	.	.	.		602
.	.	.	.		561
.	.	.	.		540
.	.	.	.	.	
.	.	.	.		540
.	.	.	.	.	
.	.	.	.	.	
.	.	.	.	.	
.	.	.	.		424
.	.	.	.	.	
.	.	.	.	.	
.	.	.	.	.	
.	.	.	.	.	
.	.	.	.	.	
.	.	.	.	.	
.	.	.	.	.	
.	.	.	.	.	
.	.	.	.	.	
.	.	.	.	.	
.	.	.	.	.	
.	.	.	.	.	
.	.	.	.	.	
.	.	.	.	.	
.	.	.	.	.	
.	.	.	.	.	
.	.	.	.	.	
.	.	.	.	.	
.	.	.	.	.	
.	.	.	.	.	
.	.	.	.	.	
.	.	.	.	.	
.	.	.	.	.	
.	.	.	.	.	
.	.	.	.	.	
.	.	.	.	.	
.	.	.	.	.	
.	.	.	.	.	
.	.	.	.	.	
.	.	.	.	.	
.	.	.	.	.	
.	.	.	.	.	
.	.	.	.	.	

breakfast_2020_17	dinner_2019_2	dinner_2019_3	dinner_2019_4	dinner_2019_5	dinner_2019_6
.	.	.	.	.	.
.	.	.	.	.	.
.	.	.	.	.	.
.	.	.	.	.	.
.	.	.	.	.	.
.	.	.	.	.	.
.	.	.	.	.	.
.	.	.	.	.	.
.	.	.	.	.	.
.	.	.	.	.	.
.	1220	1197	1274	1198	1270
.	.	.	.	.	.
.	.	.	.	.	.
.	.	.	.	.	.
.	.	.	.	.	.
.	.	.	.	.	.
.	.	.	.	.	.
.	.	.	.	.	.
.	.	.	.	.	.
.	.	.	.	.	.
.	.	.	.	.	.
.	.	.	.	.	.
.	.	.	.	.	.
.	1146	1167	.	.	.
.	.	.	.	.	.
.	.	.	.	.	.
.	1256	1086	.	.	.
.	.	.	.	.	.
.	.	.	.	.	.
.	.	.	.	.	.
.	.	.	.	.	.
.	.	.	.	.	.
.	.	.	.	.	.
.	.	.	.	.	.
.	.	.	.	.	.
.	.	.	.	.	.
.	.	.	.	.	.
.	.	.	.	.	.
.	1193	1180	.	.	1200
.	1265	1258	1318	1331	1346
407	1230	1091	1205	1167	1191
.	.	.	.	.	.
.	.	.	.	.	.
.	.	.	.	.	.
.	.	.	.	.	.
.	.	.	.	.	.
.	.	.	.	.	.
.	.	.	.	.	.
.	.	.	.	.	.
.	.	1108	.	.	.
.	.	.	.	.	.
.	.	.	.	.	.
.	.	.	.	.	.
.	.	.	.	.	.
.	.	.	.	.	.
.	1182	1153	.	.	.
.	.	.	.	.	.
.	.	.	.	.	.

	1113	1091	1131	1133	1228
	1177	1157			
	1200	1231	1212	1240	1155
	1194	1155	1226	1187	1183
446	1168	1152	1164	1209	1177
	1116	1107	1129	1210	1131
	1099	1124			
	1186				

.	.	.	.	.	.	
.	.	.	.	.	.	
.	.	.	.	.	.	
.	.	.	.	.	.	
.	.	.	.	.	.	
.	.	.	.	.	.	
	435	1296	1136	1342	1253	1380
.	.	.	.	.	.	
.	.	.	.	.	.	
.	.	.	.	.	.	
.	.	.	.	.	.	
.		1083	1078 .	.	.	
.	.	.	.	.	.	
.	.	.	.	.	.	
.	.	.	.	.	.	
.	.		1004	1046	1051	1050
.	.	.	.	.	.	
.	.	.	.	.	.	
.		1490	1231 .	.		1366
.		1199	1186	1212	1241	1166
.	.	.	.	.	.	
.	.	.	.	.	.	
.	.	.	.	.	.	
.	.	.	.	.	.	
.	.	.	.	.	.	
.		1150 .	.	.	.	
.	.	.	.	.	.	
.	.	.	.	.	.	
	408	1248	1276	1145	1147	1187
.	.	.	.		1180 .	
.		1232	1211	1194	1203	1233
.	.	.	.	.	.	
.	.	.		1107	1066	1082
.	.	.	.	.	.	
.		1191 .	.	.	.	
.	.	.	.	.	.	
.	.	.	.	.	.	
.	.	.	.	.	.	
.	.	.	.	.	.	
.		1203 .	.	.	.	
	484	1231	1212	1226	1221	1222
.	.	.	.	.	.	
.	.	.	.	.	.	
.		1134	1217	1183	1146	1178
.		1202 .		1056 .	.	
.		1419 .	.	.	.	
.		1206	1183	1212 .	.	
.	.	.	.	.	.	
.	.	.	.	.	.	
.		1055 .	.	.	.	
.		1281	1211 .	.	.	
.		1298 .	.	.	.	
.		1209 .	.	.	.	
.		1201 .	.	.	.	
.		1231 .	.	.	.	
.		1074 .	.	.	.	
.	.	.	.	.	.	
.		1088 .	.	.	.	
.		1119	1087	1080	1098	1126

.	.	.	.	.	.
.	1254	1178	1140	.	.
.	.	.	.	.	.
.	1190	.	.	.	.
.	.	.	.	.	.
.	.	.	756	.	.
.	.	.	.	.	.
.	1170	1195	1248	.	.
.	.	.	.	.	.
.	.	1150	.	.	.
.	.	1230	.	.	.
.	.	1165	1177	1161	1178
.	.	1296	.	.	.
.	.	.	.	.	.
.	.	.	.	.	.
.	.	.	.	.	.
.	.	.	.	.	.
.	.	1080	1186	1270	1252
.	.	1200	.	.	.
.	.	.	.	.	.
.	.	.	1207	.	.
.	.	.	.	.	.
.	.	.	1275	.	.
.	.	.	.	.	.
.	.	.	.	.	.
.	.	.	1130	.	.
.	.	.	1096	.	.
.	.	.	1329	.	.
.	.	.	1261	.	.
.	.	.	.	.	.
.	.	.	.	.	.
.	.	.	1291	.	.
.	.	.	.	.	.
.	.	.	1221	1179	.
.	.	.	1176	.	.
.	.	.	.	.	.
.	.	.	1089	.	.
.	.	.	.	.	.
.	.	.	.	.	.
.	.	.	.	.	.
.	.	.	1053	.	.
.	.	.	1080	.	.
.	.	.	.	.	.
.	.	.	.	.	.
.	.	.	.	.	.
.	.	.	1090	.	.
.	.	.	.	.	.
.	.	.	.	.	.
.	.	.	.	1246	.
.	.	.	.	.	.
.	.	.	.	1252	1181
.	.	.	.	959	.
.	.	.	.	1029	1033
.	.	.	.	.	.
.	.	.	.	1105	.
.	.	.	.	1240	1193
.	.	.	.	.	.
.	.	.	.	1180	1221
.	.	.	.	1340	.

.	.	.	.	.	.	
.	.	.	.	.		1142
.	.	.	.	.		1305
.	.	.	.	.	.	
.	.	.	.	.	.	
.	.	.	.	.	.	
.	.	.	.	.		1085
.	.	.	.	.		1140
.	.	.	.	.		1180
.	.	.	.	.		1197
.	.	.	.	.		1260
.	.	.	.	.		1199
.	.	.	.	.		1271
.	.	.	.	.	.	
.	.	.	.	.	.	
.	.	.	.	.	.	
.	.	.	.	.	.	
.	.	.	.	.	.	
.	.	.	.	.	.	
.	.	.	.	.	.	
.	.	.	.	.	.	
.	.	.	.	.	.	
.	.	.	.	.	.	
.	.	.	.	.	.	
.	.	.	.	.	.	
.	.	.	.	.	.	
.	.	.	.	.	.	
.	.	.	.	.	.	
.	.	.	.	.	.	
.	.	.	.	.	.	
.	.	.	.	.	.	
.	.	.	.	.	.	
.	.	.	.	.	.	
.	.	.	.	.	.	
.	.	.	.	.	.	
.	.	.	.	.	.	
.	.	.	.	.	.	
.	.	.	.	.	.	
.	.	.	.	.	.	
.	.	.	.	.	.	
.	.	.	.	.	.	
.	.	.	.	.	.	
.	.	.	.	.	.	
.	.	.	.	.	.	
.	.	.	.	.	.	
.	.	.	.	.	.	
.	.	.	.	.	.	
.	.	.	.	.	.	
.	.	.	.	.	.	
.	.	.	.	.	.	
.	.	.	.	.	.	
.	.	.	.	.	.	
.	.	.	.	.	.	
.	.	.	.	.	.	
.	.	.	.	.	.	
.	.	.	.	.	.	
.	.	.	.	.	.	

507

710

409

431

557

600

.		.	.	.	.	.
	707	.	.	.	.	.
.		.	.	.	.	.
.		.	.	.	.	.
.		.	.	.	.	.
.		.	.	.	.	.
.		.	.	.	.	.
.		.	.	.	.	.
.		.	.	.	.	.
.		.	.	.	.	.
.		.	.	.	.	.
.		.	.	.	.	.
.		.	.	.	.	.
.		.	.	.	.	.
.		.	.	.	.	.
.		.	.	.	.	.
.		.	.	.	.	.
.		.	.	.	.	.
.		.	.	.	.	.
.		.	.	.	.	.
.		.	.	.	.	.
.		.	.	.	.	.
.		.	.	.	.	.
.		.	.	.	.	.
	720	.	.	.	.	.
.		.	.	.	.	.
.		.	.	.	.	.
.		.	.	.	.	.
.		.	.	.	.	.
.		.	.	.	.	.
.		.	.	.	.	.
.		.	.	.	.	.
.		.	.	.	.	.
.		.	.	.	.	.
.		.	.	.	.	.
.		.	.	.	.	.
.		.	.	.	.	.
.		.	.	.	.	.
	498	.	.	.	.	.
	619	.	.	.	.	.
.		.	.	.	.	.
	692	.	.	.	.	.
.		.	.	.	.	.
.		.	.	.	.	.
.		.	.	.	.	.
.		.	.	.	.	.
.		.	.	.	.	.
.		.	.	.	.	.
.		.	.	.	.	.
.		.	.	.	.	.
.		.	.	.	.	.
.		.	.	.	.	.
.		.	.	.	.	.
.		.	.	.	.	.
.		.	.	.	.	.
.		.	.	.	.	.
.		.	.	.	.	.
.		.	.	.	.	.
.		.	.	.	.	.

630

600

522

579

423

468

551

	471	.	.	.	.	.
.		.	.	.	.	.
	686	.	.	.	.	.
.		.	.	.	.	.
.		.	.	.	.	.
.		.	.	.	.	.
.		.	.	.	.	.
	593	.	.	.	.	.
.		.	.	.	.	.
.		.	.	.	.	.
	569	.	.	.	.	.
.		.	.	.	.	.
.		.	.	.	.	.
.		.	.	.	.	.
.		.	.	.	.	.
.		.	.	.	.	.
.		.	.	.	.	.
	624	.	.	.	.	.
.		.	.	.	.	.
.		.	.	.	.	.
.		.	.	.	.	.
	469	.	.	.	.	.
.		.	.	.	.	.
	593	.	.	.	.	.
.		.	.	.	.	.
	471	.	.	.	.	.
	557	.	.	.	.	.
.		.	.	.	.	.
.		.	.	.	.	.
	540	.	.	.	.	.
	540	.	.	.	.	.
.		.	.	.	.	.
.		.	.	.	.	.
	450	.	.	.	.	.
	420	.	.	.	.	.
.		.	.	.	.	.
	538	.	.	.	.	.
	463	.	.	.	.	.
.		.	.	.	.	.
	524	.	.	.	.	.
.		.	.	.	.	.
	1051	.	.	.	.	.
.		.	.	.	.	.
	420	.	.	.	.	.
	572	.	.	.	.	.
.		.	.	.	.	.
	412	.	.	.	.	.
	538	.	.	.	.	.
	440	.	.	.	.	.
	663	.	.	.	.	.
.		.	.	.	.	.
	339	.	.	.	.	.
	438	.	.	.	.	.
.		.	.	.	.	.
	523	.	.	.	.	.
.		.	.	.	.	.
.		.	.	.	.	.
	576	.	.	.	.	.
	480	.	.	.	.	.

.		.	.	.	.	.
	480	.	.	.	.	.
	506	.	.	.	.	.
.		.	.	.	.	.
	388	.	.	.	.	.
	390	.	.	.	.	.
.		.	.	.	.	.
.		.	.	.	.	.
	600	.	.	.	.	.
	496	.	.	.	.	.
	630	.	.	.	.	.
	875	.	.	.	.	.
.		.	.	.	.	.
	515	.	.	.	.	.
	455	.	.	.	.	.
	477	.	.	.	.	.
	483	.	.	.	.	.
	526	.	.	.	.	.
.		.	.	.	.	.
.		.	.	.	.	.
	540	.	.	.	.	.
.		.	.	.	.	.
.		.	.	.	.	.
.		.	.	.	.	.
.		.	.	.	.	.

dinner_2019_7	dinner_2019_8	dinner_2019_9	dinner_2019_10	dinner_2019_11	dinner_2019_12
.	.	.	.	.	.
1206	.	1170	1200	.	.
.	.	.	.	.	.
.	.	.	.	.	.
.	.	.	.	.	.
.	.	.	.	.	.
.	.	.	.	.	.
.	.	.	.	.	.
.	.	.	.	.	.
.	.	.	.	.	.
1173	.	.	.	.	.
.	.	.	.	.	.
.	.	.	.	.	.
.	.	.	.	.	.
.	.	.	.	.	.
.	.	.	.	.	.
.	.	.	.	.	.
.	.	.	.	.	.
.	.	.	.	.	.
.	.	.	.	.	.
.	.	.	.	.	.
.	.	.	.	.	.
.	.	.	.	.	.
.	1234	.	.	.	.
.	.	.	.	.	.
.	.	.	.	.	.
.	.	.	.	.	.
.	.	.	.	.	.
.	.	.	.	.	.
.	.	.	.	.	.
.	.	.	.	.	.
.	.	.	.	.	.
.	.	.	.	.	.
.	.	.	.	.	.
.	.	.	.	.	.
.	.	.	.	.	.
.	.	.	.	.	.
.	.	.	.	.	.
1225	1181	1210	1320	1289	1216
1310	1263	1251	1272	1306	1314
1236	1203	1260	1219	1202	1208
.	.	.	.	.	.
.	.	.	.	.	.
.	.	.	.	.	.
.	.	.	.	.	1127
.	.	.	.	.	.
.	.	.	.	.	.
.	.	.	.	.	.
.	.	.	.	.	.
.	.	1117	1131	.	.
.	.	.	.	.	.
.	.	.	.	.	.
.	.	.	.	.	.
.	.	.	.	.	.
.	.	.	.	.	.
.	.	.	.	.	.
.	.	.	.	.	.
.	.	.	.	.	.

1130	1132	1118			
1154	1228		1184	1213	1214
1164	1179	1221	1232	1226	1218
1178	1111	1169	1122	1185	1126
		1165			

1249	1464	1159	1408	1202	1430
				1089	
			1231	1341	1230
1235	1208	1132	1211	1189	1102
1181	1142	1160	1214	1171	1143
		1284			
1178		1200	1243		
1058		1039	1157		1107
1208	1222	1197	1226	1218	1228
	1260	1286	1129	999	
1106	1181	1124	1111		1159
716					
1159	1053	1197	1153	1159	1040

1192					
1145	1173	1170	1111	1186	1166
1220	1233	1222	1271	1228	1257
1349	1196	1068			
1174					
1264	1266	1280			

.	.	.	.	.	.
.	.	.	.	.	.
1339	1326	1313	1367	1327	1314
.	.	.	.	.	.
.	.	.	.	.	.
.	.	.	.	.	.
.	.	.	.	.	.
.	.	.	.	.	.
.	.	.	.	.	.
.	.	.	.	.	.
.	.	.	.	.	.
.	.	.	.	.	.
1224	.	.	.	.	.
.	.	.	.	.	.
.	.	.	.	.	.
1023	970	.	1004	1089	1115
.	.	.	.	.	.
.	.	.	.	.	.
.	.	.	.	.	.
1102	1118	1155	.	.	1088
1173	.	.	.	.	.
.	.	.	.	.	.
.	.	.	.	.	.
1297	.	.	.	.	.
.	.	.	.	.	.
1028	1147	.	.	.	.
.	.	.	.	.	.
.	.	.	.	.	.
.	.	.	.	.	.
.	1142	.	.	.	.
.	1113	.	.	.	.
.	.	.	.	.	.
.	1127	1111	1168	1199	1160
.	1004	994	989	1026	1066
.	.	.	.	.	.
.	.	.	.	.	.
.	1140	.	.	.	.
.	1090	.	.	.	.
.	1200	.	.	.	.
.	.	.	.	.	.
.	1003	.	.	.	.
.	.	.	.	.	.
.	.	.	.	.	.
.	.	.	.	.	.
.	1090	.	.	.	.
.	.	.	.	.	.
.	1374	1597	.	.	.
.	.	.	.	.	.
.	1111	.	.	.	.
.	.	1216	1140	.	.
.	.	.	.	.	.
.	1212	1288	1278	1331	1278
.	.	.	.	.	.
.	.	1140	.	.	.
.	.	1038	.	.	.
.	.	1020	.	.	.
.	.	.	.	.	.
.	.	1264	1252	1285	1250
.	.	1164	.	.	.

.	.	.	.	.	.
.	.	.	.	.	.
.	.	1188	1139	.	.
.	.	.	.	.	.
.	.	1200	.	.	.
.	.	.	.	.	.
.	.	.	.	.	.
.	.	1043	1184	1206	1307
.	.	.	.	.	.
.	.	1214	1211	.	.
.	.	1150	1066	.	1294
.	.	1140	.	.	.
.	.	.	.	.	.
.	.	.	.	.	.
.	.	.	.	.	.
.	.	1380	1400	.	.
.	.	.	.	.	.
.	.	.	1266	1252	1204
.	.	.	1310	.	.
.	.	.	1211	.	.
.	.	.	1291	.	.
.	.	.	1281	.	.
.	.	.	1170	.	.
.	.	.	.	1110	.
.	.	.	.	.	.
.	.	.	.	.	.
.	.	.	1320	.	.
.	.	.	.	.	.
.	.	.	1130	.	.
.	.	.	421	.	.
.	.	.	.	.	.
.	.	.	.	.	.
.	.	.	.	.	.
.	.	.	.	.	.
.	.	.	.	.	.
.	.	.	.	.	.
.	.	.	.	1130	1168
.	.	.	.	1110	.
.	.	.	.	.	.
.	.	.	.	1073	1068
.	.	.	.	.	.
.	.	.	.	.	.
.	.	.	.	.	.
.	.	.	.	.	.
.	.	.	.	.	.
.	.	.	.	.	1189
.	.	.	.	.	.
.	.	.	.	1215	1080
.	.	.	.	.	.
.	.	.	.	.	.
.	.	.	.	.	1179
.	.	.	.	.	1235
.	.	.	.	.	.
.	.	.	.	.	1185
.	.	.	.	.	1082
.	.	.	.	.	.
.	.	.	.	.	.
.	.	.	.	.	.
.	.	.	.	.	1206

1230
1148
1372

1230
1331

.	.	.	.	.	.
.	.	.	.	.	.
.	.	.	.	.	.
.	.	.	.	.	.
.	.	.	.	.	.
.	.	.	.	.	.
.	.	.	.	.	.
.	.	.	.	.	.
.	.	.	.	.	.
.	.	.	.	.	.
.	.	.	.	.	.
.	.	.	.	.	.
.	.	.	.	.	.
.	.	.	.	.	.
.	.	.	.	.	.
.	.	.	.	.	.
.	.	.	.	.	.
.	.	.	.	.	.
.	.	.	.	.	.
.	.	.	.	.	.
.	.	.	.	.	.
.	.	.	.	.	.
.	.	.	.	.	.
.	.	.	.	.	.
.	.	.	.	.	.

dinner_2019_13	dinner_2019_14	dinner_2019_15	dinner_2019_16	dinner_2019_17	dinner_2020_2
.	.	.	.	.	.
.	.	.	.	.	.
.	.	.	.	.	.
.	.	.	.	.	.
.	.	.	.	.	.
.	.	.	.	.	.
.	.	.	.	.	.
.	.	.	.	.	.
.	.	.	.	.	.
.	.	.	.	.	.
.	.	.	.	.	.
.	.	.	.	.	.
.	.	.	.	.	.
.	.	.	.	.	.
.	.	.	.	.	.
.	.	.	.	.	.
.	.	.	.	.	.
.	.	.	.	.	.
.	.	.	.	.	.
.	.	.	.	.	.
.	.	.	.	.	.
.	.	.	.	.	.
.	.	.	.	.	.
.	.	.	.	.	.
.	.	.	.	.	.
.	.	.	.	.	.
.	.	.	.	.	.
.	.	.	.	.	.
.	.	.	.	.	.
.	.	.	.	.	.
.	.	.	.	.	.
.	.	.	.	.	.
.	.	.	.	.	.
.	.	.	.	.	.
.	.	.	.	.	.
.	.	.	.	.	.
.	.	.	.	.	.
.	.	.	.	.	.
1223	.	.	1230	1200	.
1318	1280	1293	1287	1290	.
1205	1177	1184	1211	1168	.
.	.	.	.	.	.
.	.	.	.	.	.
.	.	.	.	.	.
1294	1171	1201	1194	1215	.
.	.	.	.	.	.
.	.	.	.	.	.
.	.	.	.	.	.
.	.	.	.	.	.
.	.	.	.	.	.
.	.	.	.	.	.
.	.	.	.	.	.
.	.	.	.	.	.
.	.	.	.	.	.
.	.	.	.	.	.
.	.	.	.	.	.
.	.	.	.	.	.
.	.	.	.	.	.

1263	1239	1251	943		
1285	1282	1219	1218	1244	1201
1200					

1431	1419	1357	1443	1251	1141
		1150			
	1307				
1260	1251	1229	1242	1230	
1178	1201	1173	1220	1146	1183
		1110			
1233	1265	1240	1199	1244	1186
	1248	1205	1257	1149	
1201	1285	1201	1265	1189	

1260	1227	1263	1230	1260

1341	1267	1309	1394	1320
1164<br>1035	1181	1309		
1211	1244		1173	1294
1287	1278	1248	1330	1285

1195	1117				
1110	1140				
1163		1240			
1186	1219	1171	1157	1152	
1087					
1237	1200	1143	1245	1223	1192
				1246	

.	.	.	.	.	.
.	.	.	.	.	.
.	.	.	.	.	.
.	.	.	.	.	.
1200	.	.	.	.	.
1371	.	.	.	.	.
.	.	.	.	.	.
1226	1140	.	1350	1149	.
.	.	.	.	.	.
.	.	.	.	.	.
.	.	.	.	.	.
.	.	.	.	.	.
.	.	.	.	.	.
.	.	.	.	.	.
.	.	.	.	.	.
.	.	.	.	.	.
.	.	.	.	.	.
.	.	.	.	.	.
.	.	.	.	.	.
.	.	.	.	.	.
1159	1139	1132	1089	1093	.
.	.	.	.	.	.
.	.	.	.	.	.
1180	.	.	.	.	.
1100	.	.	.	.	.
1205	.	.	.	.	.
900	.	.	.	.	.
1188	1153	.	.	.	.
.	.	.	.	.	.
1158	1147	1152	1153	1206	.
1260	1233	.	.	.	.
1080	1155	.	.	.	.
.	.	.	.	.	.
538	.	.	.	.	.
1131	.	.	.	.	.
.	.	.	.	.	.
.	720	.	.	.	.
1020	1080	.	.	.	.
.	.	.	.	.	.
.	.	.	.	.	.
.	1169	.	.	.	.
.	.	.	.	.	.
.	.	.	.	.	.
.	1103	1226	.	.	.
.	1247	.	.	.	.
.	.	.	.	.	.
.	.	.	.	.	.
.	.	.	.	.	.
.	.	.	.	.	.
.	.	.	.	.	.
.	.	.	.	.	.
.	.	.	.	.	.
.	.	.	.	.	.
.	.	.	.	.	.
.	.	.	.	.	.
.	.	.	.	.	.
.	.	.	.	.	.
.	1110	1061	1060	1101	.
.	.	.	.	.	.

.	.	.	.	.	.
.	.	1125	.	.	.
.	.	1111	.	.	.
.	.	1141	1149	.	.
.	.	.	.	.	.
.	.	.	.	.	.
.	.	.	.	.	.
.	.	.	.	.	.
.	.	.	.	.	.
.	.	.	.	.	.
.	.	1249	1231	1229	.
.	.	.	.	.	.
.	.	1235	.	.	.
.	.	1245	.	.	.
.	.	.	.	.	.
.	.	.	.	.	.
.	.	.	.	.	.
.	.	.	.	.	.
.	.	.	.	.	.
.	.	.	1245	1257	.
.	.	.	.	.	.
.	.	.	1222	.	.
.	.	.	1165	.	.
.	.	.	.	.	.
.	.	.	.	.	.
.	.	.	.	.	.
.	.	.	1118	.	.
.	.	.	.	.	.
.	.	.	.	.	.
.	.	.	.	.	.
.	.	.	.	.	.
.	.	.	.	.	.
.	.	.	.	.	.
.	.	.	1257	.	.
.	.	.	1330	.	.
.	.	.	.	.	.
.	.	.	.	.	.
.	.	.	.	.	.
.	.	.	1230	.	.
.	.	.	.	.	.
.	.	.	.	.	.
.	.	.	.	.	.
.	.	.	.	1284	.
.	.	.	.	1033	.
.	.	.	.	.	.
.	.	.	.	1155	.
.	.	.	.	1305	.
.	.	.	.	1170	.
.	.	.	.	.	.
.	.	.	.	.	.
.	.	.	.	.	.
.	.	.	.	.	.
.	.	.	.	.	.
.	.	.	.	.	.
.	.	.	.	.	.
.	.	.	.	.	.
.	.	.	.	.	.
.	.	.	.	.	1148
.	.	.	.	.	1241

.	.	.	.	.	.
.	.	.	.	.	.
.	1265	.	.	1275	1189
.	.	.	.	.	.
.	.	.	.	.	.
.	.	.	.	.	.
.	.	.	.	.	.
.	.	.	.	.	1260
.	.	.	.	.	.
.	.	.	.	.	.
.	.	.	.	.	.
.	.	.	.	.	.
.	.	.	.	.	.
.	.	.	.	.	.
.	.	.	.	.	.
.	.	.	.	.	.
.	.	.	.	.	.
.	.	.	.	.	.
.	.	.	.	.	.
.	.	.	.	.	.
.	.	.	.	.	.
.	.	.	.	.	.
.	.	.	.	.	.
.	.	.	.	.	.
.	.	.	.	.	.
.	.	.	.	.	.
.	.	.	.	.	.
.	.	.	.	.	.
.	.	.	.	.	.
.	.	.	.	.	1285
.	.	.	.	.	.
.	.	.	.	.	.
.	.	.	.	.	.
.	.	.	.	.	1095
.	.	.	.	.	.
.	.	.	.	.	.
.	.	.	.	.	1367
.	.	.	.	.	1063
.	.	.	.	.	.
.	.	.	.	.	.
.	.	.	.	.	1145
.	.	.	.	.	1170
.	.	.	.	.	1087
.	.	.	.	.	1162
.	.	.	.	.	.
.	.	.	.	.	.
.	.	.	.	.	.
.	.	.	.	.	.
.	.	.	.	.	1109
.	.	.	.	.	1215
.	.	.	.	.	.
.	.	.	.	.	.
.	.	.	.	.	.
.	.	.	.	.	.
.	.	.	.	.	1284
.	.	.	.	.	.
.	.	.	.	.	1237
.	.	.	.	.	1149
.	.	.	.	.	1178

.	.	.	.	.	.	
.	.	.	.	.		1179
.	.	.	.	.		1036
.	.	.	.	.		1030
.	.	.	.	.	.	
.	.	.	.	.		1258
.	.	.	.	.	.	
.	.	.	.	.		1140
.	.	.	.	.		1175
.	.	.	.	.	.	
.	.	.	.	.	.	
.	.	.	.	.		1146
.	.	.	.	.		1166
.	.	.	.	.		1200
.	.	.	.	.	.	
.	.	.	.	.	.	
.	.	.	.	.	.	
.	.	.	.	.		1178
.	.	.	.	.		1248
.	.	.	.	.		1468
.	.	.	.	.	.	
.	.	.	.	.		1019
.	.	.	.	.		1112
.	.	.	.	.	.	
.	.	.	.	.	.	
.	.	.	.	.	.	
.	.	.	.	.	.	
.	.	.	.	.	.	
.	.	.	.	.		1139
.	.	.	.	.	.	
.	.	.	.	.	.	
.	.	.	.	.	.	
.	.	.	.	.		1194
.	.	.	.	.	.	
.	.	.	.	.	.	
.	.	.	.	.	.	
.	.	.	.	.	.	
.	.	.	.	.		1130
.	.	.	.	.		1035
.	.	.	.	.		1301
.	.	.	.	.	.	
.	.	.	.	.	.	
.	.	.	.	.		1080
.	.	.	.	.	.	
.	.	.	.	.	.	
.	.	.	.	.	.	
.	.	.	.	.	.	
.	.	.	.	.	.	
.	.	.	.	.	.	
.	.	.	.	.	.	
.	.	.	.	.	.	
.	.	.	.	.	.	
.	.	.	.	.	.	
.	.	.	.	.	.	
.	.	.	.	.	.	
.	.	.	.	.	.	
.	.	.	.	.	.	
.	.	.	.	.	.	
.	.	.	.	.	.	

.	.	.	.	.	.
.	.	.	.	.	.
.	.	.	.	.	.
.	.	.	.	.	.
.	.	.	.	.	.
.	.	.	.	.	.
.	.	.	.	.	.
.	.	.	.	.	.
.	.	.	.	.	.
.	.	.	.	.	.
.	.	.	.	.	.
.	.	.	.	.	.
.	.	.	.	.	.
.	.	.	.	.	.
.	.	.	.	.	.
.	.	.	.	.	.
.	.	.	.	.	.
.	.	.	.	.	.
.	.	.	.	.	.
.	.	.	.	.	.
.	.	.	.	.	.
.	.	.	.	.	.
.	.	.	.	.	.
.	.	.	.	.	.
.	.	.	.	.	.

dinner_2020_3	dinner_2020_4	dinner_2020_5	dinner_2020_6	dinner_2020_7	dinner_2020_8
.	.	.	.	.	.
.	.	.	.	.	.
.	.	.	.	.	.
.	.	.	.	.	.
.	.	.	.	.	.
.	.	.	.	.	.
.	.	.	.	.	.
.	.	.	.	.	.
.	.	.	.	.	.
.	.	.	.	.	.
.	.	.	.	.	.
.	.	.	.	.	.
.	.	.	.	.	.
.	.	.	.	.	.
.	.	.	.	.	.
.	.	.	.	.	.
.	.	.	.	.	.
.	.	.	.	.	.
.	.	.	.	.	.
.	.	.	.	.	.
.	.	.	.	.	.
.	.	.	.	.	.
.	.	.	.	.	.
.	.	.	.	.	.
.	.	.	.	.	.
.	.	.	.	.	.
.	.	.	.	.	.
.	.	.	.	.	.
.	.	.	.	.	.
.	.	.	.	.	.
.	.	.	.	.	.
.	.	.	.	.	.
.	.	.	.	.	.
.	.	.	.	.	.
.	.	.	.	.	.
.	.	.	.	.	.
.	.	.	.	.	.
.	.	.	.	.	.
.	.	.	.	.	1230
.	.	.	.	.	.
1203	1138	1299	.	1215	1190
.	.	.	.	.	.
.	.	.	.	.	.
.	.	.	.	.	.
.	.	.	.	.	.
.	.	.	.	.	.
.	.	.	.	.	.
.	.	.	.	.	.
.	.	.	.	.	.
.	.	.	.	.	.
.	.	.	.	.	.
.	.	.	.	.	.
.	.	.	.	.	.
.	.	.	.	.	.
.	.	.	.	.	.
.	.	.	.	.	.
.	.	.	.	.	.
.	.	.	.	.	.

1132	1148	1173	1189	1142	1138

1256	1189	1184	1175	1171	1156
1227	1188	1125	1220	1159	1120
1091	1175	984	1136	1196	1156

1192	1170	1222	1188	1231	1191

1105	1129	1135	1145	1143	
1203	1245	1245	1230	1212	1255

.	.	.	1265	.	.
.	.	.	.	.	.
1229	1242	1249	1209	1181	1183
.	.	.	.	.	.
.	.	.	.	.	.
.	.	.	.	.	.
.	.	.	.	.	.
1200	1225	1199	1203	1228	1161
.	.	.	.	.	.
.	.	.	.	.	.
.	.	.	.	.	.
.	.	.	.	.	.
.	.	.	.	.	.
.	.	.	.	.	.
.	.	.	.	.	.
.	.	.	.	.	.
.	.	.	.	.	.
.	.	.	.	.	.
.	.	.	.	.	.
.	.	.	.	.	.
.	.	.	.	.	.
.	.	.	.	.	.
.	.	.	.	.	.
.	.	.	.	.	.
.	.	.	.	.	.
1096	.	.	.	.	.
.	.	.	.	.	.
.	.	.	.	.	.
.	.	.	.	.	.
1252	1225	1220	1259	1240	1248
.	.	.	.	.	.
.	.	.	.	.	.
.	.	.	.	.	.
1109	1102	1105	1090	1143	.
.	.	.	.	.	.
1125	1138	.	.	.	.
1394	1356	.	.	.	.
.	.	.	.	.	.
.	1200	.	.	.	.
.	.	.	.	.	.
1106	1128	.	.	.	.
1147	.	.	.	.	.
1087	1086	1086	1087	1099	1101
1129	1105	.	1173	844	.
1158	.	.	.	.	.
.	.	.	.	.	.
.	.	.	.	.	.
.	.	.	.	.	.
.	.	.	.	.	.
.	.	.	.	.	.
.	.	.	.	.	.
.	.	.	.	.	.
.	.	.	.	.	.
.	.	.	.	.	.
1270	.	.	.	.	.
.	.	.	.	.	.
.	.	.	.	.	.
.	.	.	.	.	.
.	.	.	.	.	.

.	.	.	.	.	.
.	.	.	.	.	.
.	.	.	.	.	.
1063	.	.	.	.	.
.	.	.	.	.	.
1154	1174	.	1157	1218	.
.	.	.	.	.	.
.	.	.	.	.	.
.	.	.	.	.	.
.	.	.	.	.	.
.	.	.	.	.	.
.	.	.	.	.	.
.	.	.	.	.	.
.	.	.	.	.	.
.	.	.	.	.	.
.	.	.	.	.	.
.	.	.	.	.	.
1156	.	.	.	.	.
1294	.	.	.	.	.
1287	.	.	.	.	.
.	.	.	.	.	.
.	.	.	.	.	.
.	.	.	.	.	.
.	.	.	.	.	.
.	.	.	.	.	.
.	.	.	.	.	.
.	.	.	.	.	.
.	.	.	.	.	.
.	.	.	.	.	.
.	.	.	.	.	.
.	.	.	.	.	.
.	.	.	.	.	.
.	1091	1155	1116	.	.
.	.	.	.	.	.
.	.	.	.	.	.
.	.	.	.	.	.
.	.	.	.	.	.
.	.	.	.	.	.
.	.	.	.	.	.
.	.	.	.	.	.
.	.	.	.	.	.
.	.	.	.	.	.
.	.	.	.	.	.
1160	.	.	.	.	.
.	.	.	.	.	.
.	.	.	.	.	.
1058	.	.	.	.	.
.	.	.	.	.	.
.	.	.	.	.	.
.	.	.	.	.	.
.	.	.	.	.	.
.	.	.	.	.	.
1170	.	.	.	.	.
.	.	.	.	.	.
.	.	.	.	.	.
1153	1191	.	.	.	.
1245	.	.	.	.	.
.	.	.	.	.	.
.	.	.	.	.	.

.	.	.	.	.	.
1247	1501	1057	.	.	.
.	.	1035	.	.	.
.	.	.	.	.	.
.	.	.	.	.	.
1050	.	.	.	.	.
801	.	.	.	.	.
.	.	.	.	.	.
.	.	.	.	.	.
.	.	.	.	.	.
1200	.	.	.	.	.
.	.	.	.	.	.
1163	.	.	.	.	.
.	.	.	.	.	.
.	.	.	.	.	.
.	.	.	.	.	.
.	.	.	.	.	.
1080	1387	.	.	.	.
1186	1230	.	1206	1190	1186
1260	1410	1440	1379	1440	1420
1020	1075	1062	.	.	.
1062	1179	1178	1211	.	.
.	.	.	.	.	.
.	.	.	.	.	.
.	.	.	.	.	.
.	1230	1240	.	.	.
.	.	.	.	.	.
.	.	.	.	.	.
.	.	.	.	.	.
.	1110	.	.	.	.
.	.	.	.	.	.
.	1046	.	.	.	.
.	1187	1196	.	.	.
.	1200	.	.	.	.
.	.	.	.	.	.
.	1160	.	.	.	.
.	1191	1263	.	.	.
.	1309	.	.	.	.
.	1114	1105	.	.	.
.	1293	.	.	.	.
.	.	.	.	.	.
.	.	.	.	.	.
.	1125	.	.	.	.
.	.	.	.	.	.
.	1112	1261	1166	.	.
.	.	.	.	.	.
.	.	.	.	.	.
.	.	.	.	.	.
.	.	.	.	.	.
.	1125	1210	.	.	.
.	.	1163	.	.	.
.	.	.	.	.	.
.	.	.	.	.	.
.	1265	1105	1277	1172	.
.	.	.	.	.	.
.	.	.	.	.	.
.	.	.	.	.	.
.	.	.	.	.	.
.	.	1142	.	.	.

.	.	.	.	.	.
.	.	.	.	.	.
.	.	1092	1080	.	1080
.	.	1145	.	.	.
.	.	.	.	.	.
.	.	.	.	.	.
.	.	.	.	.	.
.	.	1040	.	.	.
.	.	.	.	.	.
.	.	1155	.	.	.
.	.	1280	.	.	.
.	.	.	.	.	.
.	.	.	.	.	.
.	.	1260	.	.	.
.	.	.	.	.	.
.	.	.	.	.	.
.	.	480	.	.	.
.	.	1140	.	.	.
.	.	1140	.	.	.
.	.	1270	1205	1214	1252
.	.	1199	.	.	.
.	.	.	.	.	.
.	.	.	.	.	.
.	.	1370	.	.	.
.	.	1170	.	.	.
.	.	1180	.	.	.
.	.	.	.	.	.
.	.	.	.	.	.
.	.	.	.	.	.
.	.	.	.	.	.
.	.	.	.	.	.
.	.	1100	.	.	.
.	.	.	.	.	.
.	.	.	.	.	.
.	.	.	.	.	.
.	.	.	.	.	.
.	.	.	.	.	.
.	.	.	.	.	.
.	.	.	.	.	.
.	.	1200	.	.	.
.	.	.	1030	.	.
.	.	.	.	.	.
.	.	.	.	.	.
.	.	.	1320	.	.
.	.	.	.	.	.
.	.	.	.	.	.
.	.	.	1440	.	.
.	.	.	1148	.	.
.	.	.	954	.	.
.	.	.	1200	.	.
.	.	.	.	.	.
.	.	.	.	.	.
.	.	.	.	.	.
.	.	.	.	.	.
.	.	.	.	.	.
.	.	.	1255	.	.
.	.	.	1194	.	.
.	.	.	.	.	.
.	.	.	.	.	.

.	.	.	1156	.	.
.	.	.	1135	.	.
.	.	.	1187	1194	1194
.	.	.	.	.	.
.	.	.	.	.	.
.	.	.	1530	.	.
.	.	.	1155	.	.
.	.	.	1080	.	.
.	.	.	1056	.	.
.	.	.	.	.	.
.	.	.	1215	.	.
.	.	.	.	.	.
.	.	.	.	.	.
.	.	.	1095	.	.
.	.	.	.	.	.
.	.	.	.	1230	.
.	.	.	.	.	.
.	.	.	.	1188	.
.	.	.	.	.	.
.	.	.	.	.	1049
.	.	.	.	.	.
.	.	.	.	1170	.
.	.	.	.	.	.
.	.	.	.	.	.
.	.	.	.	.	.
.	.	.	.	1215	.
.	.	.	.	1202	.
.	.	.	.	.	.
.	.	.	.	.	.
.	.	.	.	1140	.
.	.	.	.	.	.
.	.	.	.	.	.
.	.	.	.	.	.
.	.	.	.	.	.
.	.	.	.	403	1058
.	.	.	.	.	.
.	.	.	.	1155	1119
.	.	.	.	.	.
.	.	.	.	.	.
.	.	.	.	.	1132
.	.	.	.	.	.
.	.	.	.	.	1176
.	.	.	.	.	.
.	.	.	.	.	1048
.	.	.	.	.	1101
.	.	.	.	.	1210
.	.	.	.	.	.
.	.	.	.	.	.
.	.	.	.	.	1164
.	.	.	.	.	1039
.	.	.	.	.	1169
.	.	.	.	.	.
.	.	.	.	.	1253
.	.	.	.	.	.
.	.	.	.	.	.
.	.	.	.	.	1200
.	.	.	.	.	1410
.	.	.	.	.	.
.	.	.	.	.	.

dinner_2020_9	dinner_2020_10	dinner_2020_11	dinner_2020_12	dinner_2020_13	dinner_2020_14
.	.	.	.	.	.
.	.	.	.	.	.
.	.	.	.	.	.
.	.	.	.	.	.
.	.	.	.	.	.
.	.	.	.	.	.
.	.	.	.	.	.
.	.	.	.	.	.
.	.	.	.	.	.
.	.	.	.	.	.
.	.	.	.	.	.
.	.	.	.	.	.
.	.	.	.	.	.
.	.	.	.	.	.
.	.	.	.	.	.
.	.	.	.	.	.
.	.	.	.	.	.
.	.	.	.	.	.
.	.	.	.	.	.
.	.	.	.	.	.
.	.	.	.	.	.
.	.	.	.	.	.
.	.	.	.	.	.
.	.	.	.	.	.
.	.	.	.	.	.
.	.	.	.	.	.
.	.	.	.	.	.
.	.	.	.	.	.
.	.	.	.	.	.
.	.	.	.	.	.
.	.	.	.	.	.
.	.	.	.	.	.
.	.	.	.	.	.
.	.	.	.	.	.
.	.	.	.	.	.
.	.	.	.	.	.
.	.	.	.	.	.
.	.	.	.	.	.
1200	.	.	.	.	.
.	.	.	.	.	.
1170	.	.	1193	1174	.
.	.	.	.	.	.
.	.	.	.	.	.
.	.	.	.	.	.
.	.	.	.	.	.
.	.	.	.	.	.
.	.	1238	.	.	.
.	.	.	.	.	.
.	.	.	.	.	.
.	.	.	.	.	.
.	.	.	.	.	.
.	.	.	.	.	.
.	.	.	.	.	.
.	.	.	.	.	.
.	.	.	.	.	.
.	.	.	.	.	.
.	.	.	.	.	.
.	.	.	.	.	.

1088	1140				
1108	1151	1143	1112	1158	1174

1218	1232	1192	1246	1161	1244
1255	1110	1201	1157	1263	1091
1073	1184	1177	1196	1178	1203

1157	1163	1159	1174	1222	1231

1170 1243 1248 1236 1236 1210

1223	1250	1213	1284	1193	1174
1244	1226	1190	1245		
1230		1188	1129		

1206				1088	
1440	1440				

1139	1250	1119	1162	1070	1197

1116 1073

1155 1140 1089 1126 1053

1108

1154 1215

1185

1480

1168	1074	1166	1147	1116	1097
1204	1201	1172	1005		
1308		1188	1175	1226	
1140					
1167					
	1248				
1220	1235	1298			
1009	956				
1156					
1125	1225	1218	1185	1073	1087
1300	1026				
	1100	1132			
	1160	1251	1268	1119	1152
	1245				
	1055	1083	1107	1042	1048
	1335	1239			
	1151				
		660			
	1140	1167			
	1091	1208	1140		
	1157	1116			
	1180				
		1170			
	1372				
		1140			

.	.	.		.		.		.	
.	.	.		.		.		.	
.	.	.		.		.		.	
.	.	.		.		.		.	
.	.		1226		1165		1207	.	
.	.		1281	.		.		.	
.	.	.		.		.		.	
.	.		1151		1160		1157	.	
.	.		1168		1149	.		.	
.	.		1320	.		.		.	
.	.	.		.		.		.	
.	.	.		.		.		.	
.	.	.		.		.		.	
.	.	.		.		.		.	
.	.	.		.		.		.	
.	.	.		.		.		.	
.	.		1170		1174		1147		1148
.	.	.		.		.		.	
.	.		1203		1192		1063		1270
.	.		1239		1125	.		.	
.	.		1222	.		.		.	
.	.	.		.		.		.	
.	.		1018	.		.		.	
.	.		1174		1176	.		.	
.	.	.		.		.		.	
.	.	.		.		.		.	
.	.	.		.		.		.	
.	.	.		.		.		.	
.	.	.		.		.		.	
.	.	.		.		.		.	
.	.	.		.		.		.	
.	.	.		.		.		.	
.	.	.			996	.		.	
.	.	.		.		.		.	
.	.	.		.		.		.	
.	.	.		.		.		.	
.	.	.		.		.		.	
.	.	.		.		.		.	
.	.	.			1095	.		.	
.	.	.		.			1155	.	
.	.	.			975	.		.	
.	.	.		.		.		.	
.	.	.		.		.		.	
.	.	.		.		.		.	
.	.	.			1199	.		.	
.	.	.			1219		1200	.	
.	.	.			1138	.		.	
.	.	.			1223	.		.	
.	.	.			1663		959	.	
.	.	.		.		.		.	
.	.	.		.		.		.	
.	.	.		.		.		.	
.	.	.		.			1440	.	
.	.	.		.		.		.	
.	.	.		.		.		.	
.	.	.		.		.		.	
.	.	.		.		.		.	
.	.	.		.		.		.	
.	.	.		.		.		.	

.	.	.	.	.	.
.	.	.	.	1389	1165
.	.	.	.	.	.
.	.	.	.	.	.
.	.	.	.	.	.
.	.	.	.	1289	.
.	.	.	.	.	.
.	.	.	.	1170	.
.	.	.	.	.	.
.	.	.	.	.	.
.	.	.	.	1198	.
.	.	.	.	1176	1182
.	.	.	.	.	.
.	.	.	.	1020	.
.	.	.	.	1180	1150
.	.	.	.	.	.
.	.	.	.	.	.
.	.	.	.	.	1154
.	.	.	.	.	1146
.	.	.	.	.	.
.	.	.	.	.	.
.	.	.	.	.	1230
.	.	.	.	.	.
.	.	.	.	.	.
.	.	.	.	.	1170
.	.	.	.	.	1250
.	.	.	.	.	.
.	.	.	.	.	.
.	.	.	.	.	.
.	.	.	.	.	.
.	.	.	.	.	.
.	.	.	.	.	.
.	.	.	.	.	.
.	.	.	.	.	.
.	.	.	.	.	.
.	.	.	.	.	995
.	.	.	.	.	1157
.	.	.	.	.	.
.	.	.	.	.	1084
.	.	.	.	.	1269
.	.	.	.	.	.
.	.	.	.	.	1193
.	.	.	.	.	1152
.	.	.	.	.	.
.	.	.	.	.	.
.	.	.	.	.	.
.	.	.	.	.	.
.	.	.	.	.	.
.	.	.	.	.	.
.	.	.	.	.	.
.	.	.	.	.	.
.	.	.	.	.	.
.	.	.	.	.	.
.	.	.	.	.	.
.	.	.	.	.	.
.	.	.	.	.	.
.	.	.	.	.	.
.	.	.	.	.	.
.	.	.	.	.	.

1108

1207 1169

dinner_2020_15	dinner_2020_16	dinner_2020_17	lunch_2019_2	lunch_2019_3	lunch_2019_4
.	.	.	.	.	.
.	.	.	.	.	.
.	.	.	.	.	.
.	.	.	.	.	.
.	.	.	.	.	.
.	.	.	.	.	.
.	.	.	.	.	.
.	.	.	.	.	.
.	.	.	.	.	.
.	.	.	.	.	.
.	.	.	730	726	728
.	.	.	.	.	.
.	.	.	.	.	.
.	.	.	.	738	.
.	.	.	.	.	.
.	.	.	.	.	.
.	.	.	.	.	.
.	.	.	.	.	.
.	.	.	.	.	.
.	.	.	.	.	.
.	.	.	.	.	.
.	.	.	.	.	.
.	.	.	.	.	.
.	.	.	825	823	.
.	.	.	.	.	.
.	.	.	.	.	.
.	.	.	746	715	.
.	.	.	.	.	.
.	.	.	.	.	.
.	.	.	.	.	.
.	.	.	.	.	.
.	.	.	.	.	.
.	.	.	.	.	.
.	.	.	.	.	.
.	.	.	.	.	.
.	.	.	.	.	.
.	.	.	.	.	.
.	.	.	.	.	.
.	.	.	721	755	.
.	.	.	810	883	.
1195	.	1169	793	779	861
.	.	.	.	.	.
.	.	.	.	.	.
.	.	.	.	.	.
.	.	.	.	.	.
.	.	.	.	.	.
.	.	.	.	.	.
.	.	.	.	.	.
.	.	.	.	.	.
.	.	.	.	.	.
.	.	.	.	.	.
.	.	.	.	.	.
.	.	.	.	.	.
.	.	.	.	.	.
.	.	.	.	.	.
.	.	.	810	820	.
.	.	.	.	.	.
.	.	.	.	.	.

			739	755	715
			754	750	715
			795	766	760
			815	833	879
1166	1135	1190	792	773	823
			823	719	783
			729	744	
			805		
			803	713	874

.	.	.	.	.	.
.	.	.	.	.	.
.	.	.	.	.	.
.	.	.	.	.	.
.	.	.	.	.	.
.	.	.	.	.	.
1142	1241	1140	.	765	.
.	.	.	.	.	.
.	.	.	.	.	.
.	.	.	.	.	.
.	.	.	.	.	.
.	.	.	704	707	.
.	.	.	.	.	.
.	.	.	.	.	.
.	.	.	.	.	.
.	.	.	.	744	732
.	.	.	.	.	.
.	.	.	.	.	.
.	.	.	758	733	.
.	.	.	676	678	743
.	.	.	.	.	.
.	.	.	.	.	.
.	.	.	.	.	.
.	.	.	.	.	828
.	.	.	.	.	.
.	.	.	726	.	.
.	.	.	.	.	.
.	.	.	.	.	.
1185	1167	1080	774	763	771
.	.	.	.	.	.
.	.	.	769	771	751
.	.	.	.	.	.
.	.	.	.	.	742
.	.	.	.	.	.
.	.	.	731	.	.
.	.	.	.	.	.
.	.	.	.	.	.
.	.	.	.	.	.
.	.	.	.	.	.
.	.	.	773	.	.
1198	1173	1191	799	799	760
.	.	.	.	.	.
.	.	.	.	.	.
.	.	.	829	829	841
.	.	.	764	.	720
.	.	.	1031	.	.
.	.	.	802	774	734
.	.	.	916	.	.
.	.	.	.	.	0
.	.	.	664	.	.
.	.	.	854	827	.
.	.	.	759	.	.
.	.	.	774	.	.
.	.	.	856	.	.
.	.	.	780	.	.
.	.	.	745	.	.
.	.	.	.	.	.
.	.	.	735	.	.
.	.	.	783	738	770

.	.	.	.		.		.	
.	.	.		765		745		721
.	.	.		790	.		.	
.	.	.		726		820	.	
.	.	.	.		.		.	
.	.	.		756		795		725
.	.	.	.		.		.	
.	.	.	.			795		720
.	.	.	.		.		.	
.	.	.	.			727	.	
.	.	.	.			680	.	
.	.	.	.			730		755
.	.	.	.			664	.	
.	.	.	.		.		.	
.	.	.	.		.		.	
.	.	.	.		.		.	
.	.	.	.		.		.	
.	.	.	.			744		712
.	.	.	.			960	.	
.	.	.	.		.		.	
.	.	.	.		.		.	
.	.	.	.			720	.	
.	.	.	.			675		800
.	.	.	.		.			754
.	.	.	.		.		.	
.	.	.	.		.			750
.	.	.	.		.			656
.	.	.	.			1021		911
.	.	.	.		.			900
.	.	.	.		.		.	
.	.	.	.		.		.	
.	.	.	.		.			690
.	.	.	.		.		.	
.	.	.	.		.			846
.	.	.	.		.			690
.	.	.	.		.			744
.	.	.	.		.			704
.	.	.	.		.		.	
.	.	.	.		.		.	
.	.	.	.		.		.	
.	.	.	.		.			1052
.	.	.	.		.		.	
.	.	.	.		.			734
.	.	.	.		.		.	
.	.	.	.		.		.	
.	.	.	.		.		.	
.	.	.	.		.		.	
.	.	.	.		.		.	
.	.	.	.		.		.	
.	.	.	.		.		.	
.	.	.	.		.		.	
.	.	.	.		.		.	
.	.	.	.		.		.	
.	.	.	.		.		.	
.	.	.	.		.		.	
.	.	.	.		.		.	
.	.	.	.		.		.	
.	.	.	.		.		.	
.	.	.	.		.		.	

1215	1183	1203			

1243	1224	1238

788

1210 1165 1179

1138

1220

1135 1149 1167

1057	1063	1068
1144		
	1306	1229
1183	1064	1112
1153	1111	1204
1104	1049	1039

1260

.	.	.	.	.	.
	1522	1400	1342 .	.	.
.	.	.	.	.	.
.	.	.	.	.	.
.	.	.	.	.	.
.	.	.	.	.	.
.	.	.	.	.	.
.	.	.	.	.	.
.	.	.	.	.	.
.	.	.	.	.	.
.	.	.	.	.	.
.	.	.	.	.	.
.	.	.	.	.	.
.	.	.	.	.	.
.	.	.	.	.	.
.	.	.	.	.	.
.	.	.	.	.	.
.	.	.	.	.	.
.	.	.	.	.	.
.	.	.	.	.	.
.	.	.	.	.	.
.	.	.	.	.	.
.	.	.	.	.	.
.	.	.	.	.	.
	1130	1145	1145 .	.	.
.	.	.	.	.	.
.	.	.	.	.	.
.	.	.	.	.	.
.	.	.	.	.	.
.	.	.	.	.	.
.	.	.	.	.	.
.	.	.	.	.	.
.	.	.	.	.	.
.	.	.	.	.	.
.	.	.	.	.	.
.	.	.	.	.	.
.	.	.	.	.	.
.	.	.	.	.	.
	1092	1079 .	.	.	.
	1159	1082	1178 .	.	.
.	.	.	.	.	.
	1206 .		1188 .	.	.
	1174	1187 .	.	.	.
.	.	.	.	.	.
.	.	.	.	.	.
	1305 .	.	.	.	.
.	.	.	.	.	.
.	.	.	.	.	.
.		1130 .	.	.	.
.	.	.	.	.	.
.	.	.	.	.	.
.	.	.	.	.	.
.	.	.	.	.	.
.	.	.	.	.	.
.	.	.	.	.	.
.	.	.	.	.	.
	1140 .	.	.	.	.
.	.	.	.	.	.
	1107 .	.	.	.	.

	1080		1084		1095	.	.	.
	1096	.		.		.	.	.
.		.		.		.	.	.
	1328	.		.		.	.	.
.		.		.		.	.	.
.		.		.		.	.	.
	1167	.		.		.	.	.
.		.		.		.	.	.
	1290	.		.		.	.	.
.		.		.		.	.	.
.		.		.		.	.	.
	1142	.		.		.	.	.
	1299	.		.		.	.	.
.		.		.		.	.	.
.		.		.		.	.	.
.		.		.		.	.	.
.		.		.		.	.	.
.		.		.		.	.	.
.		.		.		.	.	.
.		.		.		.	.	.
.		.		.		.	.	.
	1000	.		.		.	.	.
.		.		.		.	.	.
.		.		.		.	.	.
.		.		.		.	.	.
	1179	.		.		.	.	.
	1130		1189	.		.	.	.
	1149		1200	.		.	.	.
.		.		.		.	.	.
	1327		1282		1293	.	.	.
.		.		.		.	.	.
.		.		.		.	.	.
.		.		.		.	.	.
	1140	.		.		.	.	.
.		.		.		.	.	.
	1095		1130	.		.	.	.
.		.		.		.	.	.
.			1063	.		.	.	.
	1193		1235		1171	.	.	.
.			1100	.		.	.	.
.		.		.		.	.	.
.		.		.		.	.	.
	1440		1242	.		.	.	.
	1110		1024	.		.	.	.
.		.		.		.	.	.
.		.		.		.	.	.
.			1193	.		.	.	.
.		.		.		.	.	.
.		.		.		.	.	.
.		.		.		.	.	.
.		.		.		.	.	.
.			1129		1121	.	.	.
	1020		1020	.		.	.	.
.			1034	.		.	.	.
.		.		.		.	.	.
.		.		.		.	.	.
.			996	.		.	.	.
.			1057	.		.	.	.
	1170		1160		1191	.	.	.

.	1178	1243	.	.	.
.	.	.	.	.	.
.	1114	1119	.	.	.
.	1203	.	.	.	.
.	.	.	.	.	.
.	.	.	.	.	.
.	.	.	.	.	.
.	1139	1074	.	.	.
.	.	.	.	.	.
.	1357	.	.	.	.
.	1259	1271	.	.	.
.	.	.	.	.	.
.	1165	.	.	.	.
.	1131	.	.	.	.
.	.	.	.	.	.
.	.	.	.	.	.
.	.	.	.	.	.
.	1086	1104	.	.	.
.	1101	.	.	.	.
.	.	.	.	.	.
.	.	.	.	.	.
.	1110	1117	.	.	.
.	.	.	.	.	.
.	.	1186	.	.	.
.	.	.	.	.	.
.	1051	1039	.	.	.
.	.	.	.	.	.
.	.	.	.	.	.
.	.	.	.	.	.
.	.	.	.	.	.
.	.	.	.	.	.
.	.	.	.	.	.
.	.	1422	.	.	.
.	.	1146	.	.	.
.	.	1120	.	.	.
.	.	.	.	.	.
.	.	1200	.	.	.
.	.	1156	.	.	.
.	.	1120	.	.	.
.	.	1107	.	.	.
.	.	.	.	.	.
.	.	.	.	.	.
.	.	.	.	.	.
.	.	1200	.	.	.
.	.	1163	.	.	.
.	.	.	.	.	.
.	.	1140	.	.	.
.	.	1117	.	.	.
.	.	1118	.	.	.
.	.	1195	.	.	.
.	.	.	.	.	.
.	.	.	.	.	.
.	.	1163	.	.	.
.	.	.	.	.	.
.	.	.	.	.	.
.	.	.	.	.	.
.	.	.	.	.	.
.	.	1304	.	.	.
.	.	.	.	.	.

.	.	.	.	.	.
.	.	1126	.	.	.
.	.	1108	.	.	.
.	.	1206	.	.	.
.	.	.	.	.	.
.	.	1115	.	.	.
.	.	960	.	.	.
.	.	.	.	.	.
.	.	.	.	.	.
.	.	.	.	.	.
.	.	.	.	.	.
.	.	.	.	.	.
.	.	.	.	.	.
.	.	1278	.	.	.
.	.	1185	.	.	.
.	1317	1316	.	.	.
.	.	.	.	.	.
.	.	.	.	.	.
.	.	.	.	.	.
.	.	.	.	.	.
.	.	1110	.	.	.
.	.	.	.	.	.
.	.	.	.	.	.
.	.	.	.	.	.
.	.	.	.	.	.

lunch_2019_5	lunch_2019_6	lunch_2019_7	lunch_2019_8	lunch_2019_9	lunch_2019_10	lunch_2019_11
.	.	.	.	.	.	.
.	.	745	690	750	730	.
.	.	.	.	.	.	.
.	.	.	842	.	.	.
.	.	.	.	.	.	.
.	.	.	.	.	.	.
.	.	.	.	.	.	.
.	.	.	.	.	.	.
.	.	.	.	.	.	.
.	.	.	.	.	.	.
747	719	731	.	.	.	.
.	.	.	.	.	.	.
.	.	.	.	.	.	.
.	.	.	.	.	.	.
.	.	.	.	.	.	.
.	.	.	.	.	.	.
.	.	.	.	.	.	.
.	.	.	.	.	.	.
.	.	.	.	.	.	.
.	.	.	.	.	.	.
.	.	.	.	.	.	.
.	.	.	.	.	.	.
.	.	.	.	.	.	.
.	806	.	785	.	.	.
.	.	.	.	.	.	.
.	.	.	.	.	.	.
.	.	.	.	.	.	.
.	.	.	.	.	.	.
.	.	.	.	.	.	.
.	.	.	.	.	.	.
.	.	.	.	.	.	.
.	.	.	.	.	.	.
.	.	.	.	.	.	.
.	.	.	.	.	.	.
.	.	.	.	.	.	.
.	.	.	.	.	.	.
.	.	.	.	.	.	.
.	.	.	.	.	.	.
.	729	730	.	.	496	.
848	763	871	807	910	917	870
772	751	801	803	814	766	755
.	.	.	.	.	.	.
.	.	.	.	.	.	.
.	.	.	.	.	.	.
.	.	.	.	.	.	.
.	.	.	.	.	.	.
.	.	.	.	.	.	.
.	.	.	.	.	.	.
.	.	.	.	.	.	.
.	.	.	.	.	820	.
.	.	.	.	.	.	.
.	.	.	.	.	.	.
.	.	.	.	.	.	.
.	.	.	.	800	.	.
.	.	.	.	.	.	.
.	.	.	.	.	.	.
.	.	.	.	.	.	.
.	.	.	.	.	.	.

		728				
754	812	709	823	726		
763	788	784	766	764	741	752
		702		714		
782	820					
825	809	801	775	784	804	793
808	768	768	782	771	813	810
				636		
749	820					

775	813	755	797	765	927	748
	739			722		674
742	671					
					814	
753	363		728		766	
727	709	735	696	707	708	729
		640				
759	777	788	775	765	766	772
875				854		819
783	791	776		753	726	
767	809	858		792	889	698
					28	
669	825	792	818	755	776	772
			720	718	777	730
834	861	845	811	791	810	800
		767	841			
		0				
758	767	768	786	757	728	719

740	747	779	841	741	764	764
694	798	774	772	738	802	708
794						
780						
26						
764	728					
714						
1219	798	1143	1164	897		
		810				
753						
787	863	819		651		
812	740	780	765	764		
797						

.	.	.	.	.	.	.
.	797	.	.	.	.	.
.	742	739	812	751	772	748
.	.	.	.	.	.	.
.	.	.	.	.	.	.
.	720	.	.	.	.	.
.	758	.	.	.	.	.
.	750	.	.	.	.	.
.	766	.	.	.	.	.
.	643	.	.	.	.	.
.	.	.	.	.	.	.
.	756	.	.	.	.	.
.	.	720	.	.	.	.
.	.	.	.	.	.	.
.	.	.	.	.	.	.
.	.	693	700	644	654	758
.	.	.	.	.	.	.
.	.	.	.	.	.	.
.	.	738	.	.	.	.
.	.	808	759	854	749	.
.	.	731	.	.	.	.
.	.	.	.	.	.	.
.	.	732	.	.	.	.
.	.	.	.	.	.	.
.	.	870	.	.	.	.
.	.	.	891	.	.	.
.	.	.	.	.	.	.
.	.	.	.	.	.	.
.	.	.	.	.	.	.
.	.	.	750	.	.	.
.	.	.	720	.	.	.
.	.	.	851	.	.	.
.	.	.	738	795	743	816
.	.	.	735	704	732	752
.	.	.	490	.	.	.
.	.	.	.	.	.	.
.	.	.	810	.	.	.
.	.	.	790	.	.	.
.	.	.	735	.	.	.
.	.	.	.	.	.	.
.	.	.	701	.	.	.
.	.	.	.	.	.	.
.	.	.	.	.	.	.
.	.	.	.	.	.	.
.	.	.	670	.	.	.
.	.	.	.	.	.	.
.	.	.	801	728	.	.
.	.	.	.	.	.	.
.	.	.	647	.	.	.
.	.	.	.	782	813	.
.	.	.	.	.	.	.
.	.	.	855	751	749	729
.	.	.	.	.	.	.
.	.	.	.	760	.	.
.	.	.	.	676	.	.
.	.	.	.	0	.	.
.	.	.	.	.	.	.
.	.	.	.	788	758	811
.	.	.	.	735	.	.

.	.	.	.	.	.	.
.	.	.	.	.	.	.
.	.	.	.	763	810	.
.	.	.	.	.	.	.
.	.	.	.	735	.	.
.	.	.	.	.	.	.
.	.	.	.	.	.	.
.	.	.	.	758	723	763
.	.	.	.	.	.	.
.	.	.	.	808	.	.
.	.	.	.	793	756	720
.	.	.	.	720	960	.
.	.	.	.	.	.	.
.	.	.	.	.	.	.
.	.	.	.	.	.	.
.	.	.	.	1005	855	.
.	.	.	.	.	766	.
.	.	.	.	.	909	750
.	.	.	.	.	750	.
.	.	.	.	.	901	.
.	.	.	.	.	780	.
.	.	.	.	.	739	787
.	.	.	.	.	750	.
.	.	.	.	.	.	750
.	.	.	.	.	.	.
.	.	.	.	.	.	.
.	.	.	.	.	796	.
.	.	.	.	.	.	.
.	.	.	.	.	780	.
.	.	.	.	.	748	.
.	.	.	.	.	1074	.
.	.	.	.	.	.	.
.	.	.	.	.	.	.
.	.	.	.	.	.	.
.	.	.	.	.	.	.
.	.	.	.	.	.	1010
.	.	.	.	.	.	797
.	.	.	.	.	.	824
.	.	.	.	.	.	.
.	.	.	.	.	.	733
.	.	.	.	.	.	.
.	.	.	.	.	.	.
.	.	.	.	.	.	740
.	.	.	.	.	.	.
.	.	.	.	.	.	.
.	.	.	.	.	.	.
.	.	.	.	.	.	.
.	.	.	.	.	.	780
.	.	.	.	.	.	.
.	.	.	.	.	.	.
.	.	.	.	.	.	.
.	.	.	.	.	.	.
.	.	.	.	.	.	.
.	.	.	.	1301	.	.
.	.	.	.	.	.	.
.	.	.	.	.	.	.
.	.	.	.	.	.	.
.	.	.	.	.	.	.
.	.	.	.	.	.	.

lunch_2019_12	lunch_2019_13	lunch_2019_14	lunch_2019_15	lunch_2019_16	lunch_2019_17
.	589	616	.	.	.
.	.	751	780	.	.
.	.	.	.	.	.
945	.	.	.	.	.
.	.	.	.	.	.
.	.	.	.	.	.
.	.	.	.	.	.
.	.	.	.	.	.
.	.	.	.	.	.
.	.	.	.	.	.
.	.	.	.	.	.
.	.	.	814	.	.
.	.	.	.	.	.
.	.	.	.	.	.
.	.	.	.	.	.
.	.	.	.	.	.
.	.	.	.	.	.
.	.	.	.	.	.
.	.	.	.	.	.
.	.	.	.	.	.
.	.	.	.	.	.
.	.	.	.	.	.
.	.	.	.	.	.
.	.	.	.	.	.
.	.	.	.	.	.
.	.	.	.	.	.
.	.	.	.	.	.
.	.	.	.	.	.
.	.	.	.	.	.
.	.	.	.	.	.
.	.	.	.	.	.
.	.	.	.	.	.
.	.	.	.	.	.
.	.	.	.	.	.
.	.	.	.	.	.
.	.	.	.	.	.
.	.	.	.	.	.
.	.	.	.	.	.
720	761	.	0	761	736
873	817	851	842	923	729
787	690	696	691	795	745
.	.	.	.	.	.
.	.	.	.	.	.
.	.	.	.	.	.
749	742	739	739	732	722
.	.	.	.	.	.
.	.	.	.	.	.
.	.	.	.	.	.
.	.	.	.	.	.
.	.	.	.	.	.
.	.	.	.	.	.
.	.	.	.	.	.
.	.	.	.	.	.
.	.	.	.	.	.
.	.	.	.	.	.
.	.	.	.	.	.
.	.	.	.	.	.
.	.	.	.	.	.

762	762	771	761	722	767
804	850	772	820	800	835
730					

772	780	545	769	893	744
		765			
606		724	695		
854	682	769	784	819	773
797	789	747	768	767	783
632			674		
774	606	795	787	781	767
747	881	849	845	871	814
737	746	724	715	622	738

756

728 752 711 708 715 748

789 771

741	762	780	752	745	750
751					
683	666				
781	827	845	863		
796	766				
733	716	766	723	723	722
796	713	839	869	709	795

724	751	725			
743	734	745			
894	829				
761	779	763	800	834	769
784	762	790			
750					
784	661	752	774	765	739
769					
721					
736					
					730
866					
60					
840					

.	.	.	.	.	.
.	.	.	.	.	.
.	.	.	.	.	.
840	.	.	.	.	.
843	.	.	.	.	.
779	780	.	.	.	.
.	.	.	.	.	.
.	926	730	.	885	930
824	.	.	.	.	.
.	.	.	.	.	.
.	.	.	.	.	.
.	.	.	.	.	.
.	1380	.	.	.	.
.	.	.	.	.	.
.	.	.	.	.	.
.	.	.	.	.	.
.	.	.	.	.	.
858	.	.	.	.	.
.	.	.	.	.	.
.	.	.	.	.	.
.	757	833	818	748	761
.	.	.	.	.	.
.	.	.	.	.	.
.	795	.	.	.	.
.	720	.	.	.	.
.	901	.	.	.	.
.	.	.	.	.	.
.	825	720	.	.	.
.	.	.	.	.	.
.	722	741	752	725	722
.	795	730	.	.	.
.	750	735	.	.	.
.	.	.	.	.	.
.	.	.	.	.	.
.	780	.	.	.	.
.	.	.	.	.	.
.	.	1154	.	.	.
.	840	748	.	.	.
.	.	.	.	.	.
.	.	.	.	.	.
.	.	1169	.	.	.
.	.	773	.	.	.
.	.	.	.	.	.
.	.	788	.	.	.
.	.	743	.	.	.
.	.	781	.	.	.
.	.	.	.	.	.
.	.	.	.	.	.
.	.	.	.	.	.
.	.	.	.	.	.
.	.	824	.	.	.
.	.	660	.	.	.
.	.	900	.	.	.
.	.	.	.	.	.
.	.	.	.	.	.
.	.	20	.	.	.
.	.	.	.	.	.
.	.	820	726	734	743
.	.	.	.	.	.

.	.	.	.	.	.
.	.	.	770	.	.
.	.	.	730	.	.
.	.	.	832	848	880
.	.	.	.	.	.
.	.	.	.	.	.
.	.	.	.	.	.
.	.	.	.	.	.
.	.	.	811	.	.
.	.	.	945	.	.
.	.	.	909	935	881
.	.	.	.	.	.
.	.	.	.	.	.
.	.	.	800	.	.
.	.	.	770	.	.
.	.	.	.	.	.
.	.	.	.	.	.
.	.	.	.	.	.
.	.	.	.	.	.
.	.	.	.	749	741
.	.	.	.	723	.
.	.	.	.	798	.
.	.	.	.	826	.
.	.	.	.	720	.
.	.	.	.	1242	.
.	.	.	.	.	.
.	.	.	.	730	.
.	.	.	.	.	.
.	.	.	.	841	.
.	.	.	.	.	.
.	.	.	.	.	.
.	.	.	.	.	.
.	.	.	.	.	.
.	.	.	.	784	.
.	.	.	.	732	.
.	.	.	.	.	.
.	.	.	.	.	.
.	.	.	.	.	.
.	.	.	.	790	.
.	.	.	.	.	.
.	.	.	.	.	.
.	.	.	.	.	.
.	.	.	.	.	794
.	.	.	.	.	686
.	.	.	.	.	.
.	.	.	.	.	730
.	.	.	.	.	725
.	.	.	.	.	726
.	.	.	.	.	.
.	.	.	.	.	.
.	.	.	.	.	680
.	.	.	.	.	.
.	.	.	.	.	.
.	.	.	.	.	.
.	.	.	.	.	.
.	.	.	.	.	.
.	.	.	.	.	.
.	.	.	.	.	.
.	.	.	.	.	.

803	758	725

lunch_2020_2	lunch_2020_3	lunch_2020_4	lunch_2020_5	lunch_2020_6	lunch_2020_7	lunch_2020_8
.	.	.	.	.	.	.
.	.	.	.	.	.	.
.	.	.	.	.	.	.
.	.	.	.	.	.	.
.	.	.	.	.	.	.
.	.	.	.	.	.	.
.	.	.	.	.	.	.
.	.	.	.	.	.	.
.	.	.	.	.	.	.
.	.	.	.	.	.	.
.	.	.	.	.	.	.
.	.	.	.	.	.	.
.	.	.	.	.	.	.
.	.	.	.	.	.	.
.	.	.	.	.	.	.
.	.	.	.	.	.	.
.	.	.	.	.	.	.
.	.	.	.	.	.	.
.	.	.	.	.	.	.
.	.	.	.	.	.	.
.	.	.	.	.	.	.
.	.	.	.	.	.	.
.	.	.	.	.	.	.
.	.	.	.	.	.	.
.	.	.	.	.	.	.
.	.	.	.	.	.	.
.	.	.	.	.	.	.
.	.	.	.	.	.	.
.	.	.	.	.	.	.
.	.	.	.	.	.	.
.	.	.	.	.	.	.
.	.	.	.	.	.	.
.	.	.	.	.	.	.
.	.	.	.	.	.	.
.	.	.	.	.	.	.
.	.	.	.	.	.	.
.	.	.	.	.	.	.
.	.	.	.	.	.	.
.	.	0	.	.	.	720
.	.	.	.	.	.	.
.	.	.	836	.	.	.
.	.	.	.	.	.	.
.	.	.	.	.	.	.
.	.	.	.	.	.	.
.	.	.	.	.	.	.
.	.	.	.	.	.	.
.	.	.	.	.	.	.
.	.	.	.	.	.	.
.	.	.	.	.	.	.
.	.	.	.	.	.	.
.	.	.	.	.	.	.
.	.	.	.	.	.	.
.	.	.	.	.	.	.
.	.	.	.	.	.	.
.	.	.	.	.	.	.
.	.	.	.	.	.	.
.	.	.	.	.	.	.
.	.	.	.	.	.	.

831 758 774 824 765 792 752

30

940

788	776	740	726	769	790	740
789	738	740	779	713	735	752
793	796	807	772	777	768	759

787 766 713 761 759 783 732

737	704	742	744	718	722	
750	719	762	697	753	697	728

.		.		.		.		.			875	.	
.		.		.		.		.		.		.	
	777		773		829		796		740		768		745
.		.		.		.		.		.		.	
.		.		.		.		.		.		.	
.			779	.		.		.		.		.	
.		.		.		.		.		.		.	
	774		762		823		780		799		806		784
.		.		.		.		.		.		.	
.		.		.		.		.		.		.	
.		.		.		.		.		.		.	
.		.		.		.		.		.		.	
	728	.		.		.		.		.		.	
.		.		.		.		.		.		.	
.		.		.		.		.		.		.	
.		.		.		.		.		.		.	
.		.		.		.		.		.		.	
.		.		.		.		.		.		.	
.		.		.		.		.		.		.	
.		.		.		.		.		.		.	
.		.		.		.		.		.		.	
.		.		.		.		.		.		.	
.		.		.		.		.		.		.	
.		.		.		.		.		.		.	
.		.		.		.		.		.		.	
.			665	.		.		.		.		.	
.		.		.		.		.		.		.	
.		.		.		.		.		.		.	
.		.		.		.		.		.		.	
	789		748		736		743		780		739		728
.		.				.		.		.		.	
.		.		.		.		.		.		.	
.		.		.		.		.		.		.	
	743		737		749		738		670		751	.	
.		.		.		.		.		.		.	
	523		511		773		708		753	.			726
	806		867		777	.		.		.		.	
	713	.		.		.		.		.		.	
	712		729		814	.		.			789	.	
.		.		.		.		.		.		.	
	764		800		750		751	.			751	.	
	826		746	.		.		.		.		.	
	725		735		724		723		730		735		728
	772		805		737		725		741		754	.	
	785	.		.		.		.		.		.	
	804	.		.		.		.		.		.	
.		.		.		.		.		.		.	
.		.		.		.		.		.		.	
	750	.		.		.		.		.		.	
.		.		.		.		.		.		.	
.		.		.		.		.		.		.	
	810	.		.		.		.		.		.	
	649	.		.		.		.		.		.	
	690	.		.		.		.		.		.	
	940		847	.		.		.		.		.	
.		.		.		.		.		.		.	
	741	.		.		.		.		.		.	
	718	.		.		.		.		.		.	
	782	.		.		.		.		.		.	

670	.	.	.	.	.	.
840	.	.	.	.	.	.
765	.	.	.	.	.	.
761	768	.	.	.	.	.
.	.	.	.	.	.	.
885	1012	932	974	1008	904	.
896	.	.	.	.	.	.
.	.	.	.	.	.	.
693	.	.	.	.	.	.
765	.	.	.	.	.	.
752	.	.	.	.	.	.
766	816	.	.	.	.	.
856	.	.	.	.	.	.
749	.	.	.	.	.	.
.	.	.	.	.	.	.
.	.	.	.	.	.	.
.	.	.	.	.	.	.
776	728	.	.	.	.	.
759	810	.	.	.	.	.
873	798	.	.	.	.	.
780	.	.	.	.	.	.
.	.	.	.	.	.	.
739	.	.	.	.	.	.
.	.	.	.	.	.	.
.	761	.	.	.	.	.
.	.	.	.	.	.	.
.	.	.	.	.	.	.
.	.	.	.	.	.	.
753	.	.	.	.	.	.
.	.	.	.	.	.	.
.	.	.	.	.	.	.
864	.	.	.	.	.	.
818	771	833	757	794	.	.
815	759	.	.	755	.	.
.	.	.	.	.	.	.
.	.	.	.	.	.	.
.	.	.	.	.	.	.
.	.	.	.	.	.	.
690	757	.	.	.	.	.
780	.	.	.	.	.	.
.	.	.	.	.	.	.
.	.	.	.	.	.	.
780	781	.	.	.	.	.
.	810	.	.	.	.	.
.	781	.	.	.	.	.
.	.	.	.	.	.	.
.	747	.	.	.	.	.
.	726	.	.	.	.	.
.	.	.	.	.	.	.
.	720	.	.	.	.	.
.	.	.	.	.	.	.
.	.	.	.	.	.	.
.	855	.	.	.	.	.
.	.	.	.	.	.	.
.	740	.	.	.	.	.
.	775	726	.	.	.	.
.	820	.	.	.	.	.
.	.	.	.	.	.	.
.	1005	.	.	.	.	.

.	.	.	.	.	.	.
.	.	905	706	.	.	.
.	.	.	.	.	.	.
.	750	.	.	.	.	.
.	.	.	.	.	.	.
.	660	.	.	.	.	.
.	.	.	.	.	.	.
.	.	.	.	.	.	.
.	.	.	.	.	.	.
.	750	.	.	.	.	.
.	740	.	.	.	.	.
.	.	.	.	.	.	.
.	.	.	.	.	.	.
.	.	.	.	.	.	.
.	.	.	.	.	.	.
.	767	.	.	.	.	.
.	1396	.	.	.	.	.
.	.	788	.	.	.	.
.	.	.	.	.	1007	1063
.	500	213	0	193	0	0
.	683	685	679	.	.	.
.	740	773	750	759	750	.
.	826	.	.	.	.	.
.	.	740	.	.	.	.
.	.	.	.	.	.	.
.	.	759	795	.	.	.
.	.	.	.	.	.	.
.	.	.	.	.	.	.
.	.	.	.	.	.	.
.	.	721	684	.	.	.
.	.	.	.	.	.	.
.	.	746	.	.	.	.
.	.	812	916	.	.	.
.	.	730	.	.	.	.
.	.	.	.	.	.	.
.	.	757	.	.	.	.
.	.	794	767	765	.	.
.	.	743	.	.	.	.
.	.	730	762	.	.	.
.	.	777	.	.	.	.
.	.	.	.	.	.	.
.	.	.	.	.	.	.
.	.	802	735	.	.	.
.	.	.	.	.	.	.
.	.	682	729	742	.	.
.	.	.	.	.	.	.
.	.	.	.	.	.	.
.	.	.	.	.	.	.
.	.	.	.	.	.	.
.	.	660	670	.	.	.
.	.	.	880	.	.	.
.	.	.	.	.	.	.
.	.	.	.	.	.	.
.	.	717	772	806	678	.
.	.	.	.	.	.	.
.	.	.	.	.	.	.
.	.	.	.	.	.	.
.	.	.	.	.	.	.
.	.	.	703	.	.	.

.	.	.	.	.	.	.
.	.	.	.	.	.	.
.	.	.	723	790	.	730
.	.	.	735	.	.	.
.	.	.	.	.	.	.
.	.	.	.	.	.	.
.	.	.	.	.	.	.
.	.	.	760	.	.	.
.	.	.	760	.	.	.
.	.	.	796	.	.	.
.	.	.	860	.	.	.
.	.	.	.	.	.	.
.	.	.	756	.	.	.
.	.	.	750	.	.	.
.	.	.	.	.	.	.
.	.	.	.	.	.	.
.	.	.	983	.	.	.
.	.	.	767	.	.	.
.	.	.	.	.	.	.
.	.	.	811	779	780	813
.	.	.	503	645	.	.
.	.	.	.	.	.	.
.	.	.	.	.	.	.
.	.	.	861	.	.	.
.	.	.	825	.	.	.
.	.	.	844	738	781	742
.	.	.	.	.	.	.
.	.	.	.	.	.	.
.	.	.	.	.	.	.
.	.	.	.	.	.	.
.	.	.	769	.	.	.
.	.	.	.	.	.	.
.	.	.	.	.	.	.
.	.	.	.	750	.	.
.	.	.	.	.	.	.
.	.	.	810	.	.	.
.	.	.	.	.	.	.
.	.	.	.	711	.	.
.	.	.	.	.	.	.
.	.	.	960	.	.	.
.	.	.	.	786	.	.
.	.	.	.	840	.	.
.	.	.	.	.	.	.
.	.	.	.	660	.	.
.	.	.	.	.	.	.
.	.	.	.	.	.	.
.	.	.	.	.	.	.
.	.	.	.	762	.	.
.	.	.	.	821	.	.
.	.	.	.	900	.	.
.	.	.	.	.	.	.
.	.	.	.	690	.	.
.	.	.	.	.	.	.
.	.	.	.	.	.	.
.	.	.	.	.	.	.
.	.	.	.	826	.	.
.	.	.	.	803	.	.
.	.	.	.	781	.	.
.	.	.	.	.	.	.

.	.	.	.	795	.	.
.	.	.	.	812	.	.
.	.	.	.	.	770	793
.	.	.	720	.	.	.
.	.	.	.	870	.	.
.	.	.	.	908	.	.
.	.	.	.	870	.	.
.	.	.	.	660	.	.
.	.	.	.	683	.	.
.	.	.	.	840	.	.
.	.	.	.	.	1050	.
.	.	.	.	.	.	.
.	.	.	.	840	.	.
.	.	.	.	692	720	.
.	.	.	.	.	.	.
.	.	.	.	.	960	.
.	.	.	.	.	748	.
.	.	.	.	.	780	.
.	.	.	.	.	.	.
.	.	.	.	.	729	698
.	.	.	.	.	.	.
.	.	.	.	.	789	.
.	.	.	.	.	.	.
.	.	.	.	.	.	.
.	.	.	.	.	.	.
.	.	.	.	.	720	.
.	.	.	.	.	.	.
.	.	.	.	.	817	.
.	.	.	.	.	.	.
.	.	.	.	.	750	.
.	.	.	.	.	720	.
.	.	.	.	.	.	.
.	.	.	.	.	871	.
.	.	.	.	.	.	.
.	.	.	.	.	944	.
.	.	.	.	.	.	.
.	.	.	.	.	881	824
.	.	.	.	.	.	.
.	.	.	.	.	.	.
.	.	.	.	.	.	808
.	.	.	.	.	.	.
.	.	.	.	.	.	780
.	.	.	.	.	.	.
.	.	.	.	.	.	818
.	.	.	.	.	.	756
.	.	.	.	.	.	756
.	.	.	.	.	.	610
.	.	.	.	.	.	.
.	.	.	.	.	.	757
.	.	.	.	.	.	861
.	.	.	.	.	.	711
.	.	.	.	.	.	.
.	.	.	.	.	.	879
.	.	.	.	.	.	738
.	.	.	.	.	.	.
.	.	.	.	.	.	807
.	.	.	.	.	.	795
.	.	.	.	.	.	.
.	.	.	.	.	.	.

743

735

711

lunch_2020_9	lunch_2020_10	lunch_2020_11	lunch_2020_12	lunch_2020_13	lunch_2020_14
.	.	.	.	.	.
.	.	.	.	.	.
.	.	.	.	.	.
.	.	.	.	.	.
.	.	.	.	.	.
.	.	.	.	.	.
.	.	.	.	.	.
.	.	.	.	.	.
.	.	.	.	.	.
.	.	.	.	.	.
.	.	.	.	.	.
.	.	.	.	.	.
.	.	.	.	.	.
.	.	.	.	.	.
.	.	.	.	.	.
.	.	.	.	.	.
.	.	.	.	.	.
.	.	.	.	.	.
.	.	.	.	.	.
.	.	.	.	.	.
.	.	.	.	.	.
.	.	.	.	.	.
.	.	.	.	.	.
.	.	.	.	.	.
.	.	.	.	.	.
.	.	.	.	.	.
.	.	.	.	.	.
.	.	.	.	.	.
.	.	.	.	.	.
.	.	.	.	.	.
.	.	.	.	.	.
.	.	.	.	.	.
.	.	.	.	.	.
.	.	.	.	.	.
.	.	.	.	.	.
.	.	.	.	.	.
.	.	.	.	.	.
720	.	.	.	.	.
.	.	.	.	.	.
.	.	.	.	.	.
.	.	.	.	.	.
.	.	.	.	.	.
.	.	.	.	.	.
.	.	.	.	.	.
.	.	.	.	.	.
.	.	791	.	.	.
.	.	.	.	.	.
.	.	.	.	.	.
.	.	.	.	.	.
.	.	.	.	.	.
.	.	.	.	.	.
.	.	.	.	.	.
.	.	.	.	.	.
.	.	.	.	.	.
.	.	.	.	.	.
.	.	.	.	.	.
.	.	.	.	.	.

768 784

750 869 753 789 715 789

758

791	768	809	747	728	
745	745	777	793	765	740
785	779	786	771	764	697

716	744	772	755	746	737

734 729 710 737 732 722

810	828	792	848	817	808
	709	732		797	
759	746	740	715		
716	727	656			
782					
	821	825	773	761	761
695			683		

918

1057
210 0

782	799	797	788	779	774

746					
				821	856
899		859	916		694
823					
790					
770	597				
934	769				
826					

673	736	731	809	745	747
783	794	768	875	690	
819		805	715	880	
1116					
1050					
767					
	854				
774	815	778			
816	780				
	729				
1017					
734	808	770	793	800	765
967					
830					
670	821				
	741				
	743	753			
	796	831	605	819	710
	830				
	720				
	730	745	752	747	792
	805	795			
	756				
		762			
	780	833			
	710	833	819		
	781				
	779	806			
	30	720			
	720	767			
	720				
		759			
		720			

.	.	.	.	.	.
.	.	786	.	.	.
.	.	.	.	.	.
.	.	.	.	.	.
.	.	762	770	784	.
.	.	812	.	.	.
.	.	.	.	.	.
.	.	692	666	657	758
.	.	741	743	.	.
.	.	900	.	780	.
.	.	.	.	.	.
.	.	730	.	.	.
.	.	.	.	.	.
.	.	.	.	.	.
.	.	780	.	.	.
.	.	900	.	.	.
.	.	759	744	734	807
.	.	735	.	.	.
.	.	790	794	799	766
.	.	826	790	.	.
.	.	922	.	.	.
.	.	.	.	.	.
.	.	931	.	.	.
.	.	755	710	.	.
.	.	.	.	.	.
.	.	.	.	.	.
.	.	.	.	.	.
.	.	.	.	.	.
.	.	740	.	.	.
.	.	.	.	.	691
.	.	.	793	.	.
.	.	.	.	.	.
.	.	.	706	.	.
.	.	.	.	.	.
.	.	.	752	.	.
.	.	.	.	.	.
.	.	.	.	.	.
.	.	.	752	.	.
.	.	.	.	.	.
.	.	.	738	726	.
.	.	.	.	.	.
.	.	.	.	.	.
.	.	.	.	.	.
.	.	.	.	.	.
.	.	.	832	.	.
.	.	.	831	746	.
.	.	.	735	.	.
.	.	.	740	.	.
.	.	.	1244	457	.
.	.	.	.	790	.
.	.	.	.	.	.
.	.	.	.	.	.
.	.	.	.	817	.
.	.	.	.	.	.
.	.	.	.	.	.
.	.	.	.	750	.
.	.	.	.	.	.
.	.	.	.	.	.
.	.	.	.	.	.

.	.	.	.	.	.
.	.	.	.	1101	977
.	.	.	.	.	.
.	.	.	.	841	728
.	.	.	.	690	.
.	.	.	.	900	.
.	.	.	.	750	.
.	.	.	.	750	.
.	.	.	.	.	.
.	.	.	.	.	.
.	.	.	.	748	.
.	.	.	.	826	761
.	.	.	.	769	.
.	.	.	.	720	.
.	.	.	.	817	766
.	.	.	.	.	.
.	.	.	.	.	810
.	.	.	.	.	909
.	.	.	.	.	765
.	.	.	.	.	.
.	.	.	.	.	.
.	.	.	.	.	627
.	.	.	.	.	.
.	.	.	.	.	.
.	.	.	.	.	772
.	.	.	.	.	720
.	.	.	.	.	791
.	.	.	.	.	.
.	.	.	.	.	836
.	.	.	.	.	.
.	.	.	.	.	813
.	.	.	.	.	.
.	.	.	.	.	972
.	.	.	.	.	.
.	.	.	.	.	.
.	.	.	.	.	896
.	.	.	.	.	795
.	.	.	.	.	.
.	.	.	.	.	725
.	.	.	775	.	758
.	.	.	.	.	690
.	.	.	.	756	779
.	.	.	.	.	720
.	.	.	.	.	.
.	.	.	.	.	.
.	.	.	.	.	.
.	.	.	.	.	.
.	.	.	.	.	.
.	.	.	.	.	.
.	.	.	.	.	.
.	.	.	.	.	.
.	.	.	.	.	.
.	.	.	.	.	.
.	.	.	.	.	.
.	.	.	.	.	.
.	.	.	.	.	.
.	.	.	.	.	.
.	.	.	.	.	.
.	.	.	.	.	.

743	756
808	774

lunch_2020_15	lunch_2020_16	lunch_2020_17	steps_2019_2	steps_2019_3	steps_2019_4
.	.	.	.	.	.
.	.	.	.	.	.
.	.	.	10986	10293	8513
.	.	.	4513	4506	.
.	.	.	.	.	.
.	.	.	5152	.	3888
.	.	.	.	.	5251
.	.	.	.	.	.
.	.	.	.	.	.
.	.	.	.	.	.
.	.	.	5292	4435	5028
.	.	.	.	.	.
.	.	.	.	.	.
.	.	.	.	5145	.
.	.	.	.	.	.
.	.	.	.	.	.
.	.	.	4973	.	.
.	.	.	.	1379	.
.	.	.	.	.	.
.	.	.	.	.	.
.	.	.	.	.	.
.	.	.	.	4704	.
.	.	.	.	.	.
.	.	.	4612	5558	6287
.	.	.	.	.	.
.	.	.	.	.	.
.	.	.	8112	4602	1557
.	.	.	.	.	.
.	.	.	.	.	.
.	.	.	.	.	.
.	.	.	.	.	.
.	.	.	1741	.	2419
.	.	.	.	.	.
.	.	.	.	.	.
.	.	.	6493	.	6322
.	.	.	.	.	.
.	.	.	.	5679	8000
.	.	.	.	.	.
.	.	.	.	.	.
.	.	.	.	.	.
.	.	.	1563	5191	1406
.	.	.	.	.	.
.	.	.	.	3227	.
.	.	.	.	.	.
.	.	.	2585	2707	4133
.	.	.	.	.	.
.	.	.	.	.	.
.	.	.	.	.	.
.	.	.	6044	.	.
.	.	.	.	.	.
.	.	.	.	.	.
.	.	.	.	.	.
.	.	.	.	.	.
.	.	.	.	.	.
.	.	.	.	.	.
.	.	.	5154	4591	.
.	.	.	.	.	5905
.	.	.	.	.	.

.	.	.	4090	17668	.
.	.	.	.	.	.
.	.	.	3092	1681	.
.	.	.	.	.	.
.	.	.	.	.	.
.	.	.	2337	1353	1110
.	.	.	.	.	.
.	.	.	5106	2599	8159
.	.	.	.	.	.
.	.	.	6027	7899	11460
.	.	.	2160	.	.
.	.	.	.	.	7883
.	.	.	.	9055	.
.	.	.	14370	.	9282
.	.	.	1792	2192	12223
.	.	.	.	1185	.
.	.	.	.	.	.
.	.	.	.	4893	.
.	.	.	.	.	.
.	.	.	.	.	.
.	.	.	6973	6193	5939
.	.	.	.	.	.
.	.	.	.	2306	.
.	.	.	.	.	.
.	.	.	.	.	.
.	.	.	.	5188	.
.	.	.	.	.	.
.	.	.	.	.	.
735	816	841	13592	11118	11988
.	.	.	7131	1474	.
.	.	.	.	.	.
.	.	.	12405	.	.
.	.	.	.	.	.
.	.	.	.	.	1033
.	.	.	533	.	.
.	.	.	.	.	.
.	.	.	.	.	.
.	.	.	.	.	.
.	.	.	12996	.	10857
.	.	.	.	7162	.
.	.	.	.	.	.
.	.	.	4492	.	.
.	.	.	.	.	.
.	.	.	.	.	.
.	.	.	.	.	.
.	.	.	.	.	.
.	.	.	3847	.	354
.	.	.	.	.	2128
.	.	.	2943	2327	1953
.	.	.	.	.	.
.	.	.	3028	2318	.
.	.	.	265	.	.
.	.	.	.	1912	.
.	.	.	.	.	.
.	.	.	.	.	5802
.	.	.	.	.	.
.	.	.	.	.	.
.	.	.	12320	1492	11003
.	.	.	.	.	.

.	.	.	8580	.	6160
.	.	.	.	.	.
.	.	.	.	.	.
.	.	.	.	.	.
.	.	.	1066	.	.
.	.	.	10873	.	.
744	818	754	14504	12545	12426
.	.	.	.	.	.
.	.	.	6736	.	.
.	.	.	.	5451	.
.	.	.	.	.	.
.	.	.	7979	5382	.
.	.	.	.	.	.
.	.	.	.	.	.
.	.	.	.	5099	41
.	.	.	.	5944	4852
.	.	.	.	.	.
.	.	.	1152	.	.
.	.	.	3390	2052	5057
.	.	.	1837	1208	.
.	.	.	1493	36	.
.	.	.	.	4148	.
.	.	.	.	.	.
.	.	.	.	4940	3517
751	.	.	.	.	.
.	.	.	.	.	.
.	.	.	.	.	.
.	.	.	6828	.	8750
767	773	838	2135	1667	2851
.	.	.	.	.	.
.	.	.	7524	8675	7985
.	.	.	.	.	6892
.	.	.	14746	.	12751
.	.	.	.	.	.
.	.	.	4884	.	.
.	.	.	.	.	.
.	.	.	.	.	6975
.	.	.	.	.	.
.	.	.	.	6910	.
.	.	.	.	.	.
800	796	792	2337	4934	7876
.	.	.	.	.	.
.	.	.	.	.	.
.	.	.	.	.	.
.	.	.	5830	.	3023
.	.	.	343	.	.
.	.	.	8143	4735	10183
.	.	.	3188	.	.
.	.	.	6671	.	.
.	.	.	.	.	.
.	.	.	6577	8867	.
.	.	.	.	.	.
.	.	.	1261	.	.
.	.	.	9166	.	.
.	.	.	4199	.	.
.	.	.	.	.	.
.	.	.	52	.	.
.	.	.	.	.	.
.	.	.	3938	5651	5325

.	.	.	12441	.	.
.	.	.	4365	.	11041
.	.	.	2938	.	.
.	.	.	8061	8334	.
.	.	.	9446	.	.
.	.	.	3991	2589	2322
.	.	.	.	.	.
.	.	.	2175	2105	3532
.	.	.	.	53	.
.	.	.	.	.	.
.	.	.	.	9781	.
.	.	.	.	9596	7391
.	.	.	.	10172	.
.	.	.	.	1215	.
.	.	.	.	836	.
.	.	.	.	69	.
.	.	.	.	.	.
.	.	.	.	5826	2293
.	.	.	.	.	.
.	.	.	.	1323	.
.	.	.	.	.	.
.	.	.	.	3321	.
.	.	.	.	7514	3903
.	.	.	.	765	757
.	.	.	.	.	.
.	.	.	.	.	1436
.	.	.	.	.	2571
.	.	.	.	.	3650
.	.	.	.	.	3774
.	.	.	.	.	.
.	.	.	.	.	8886
.	.	.	.	.	8273
.	.	.	.	.	2644
.	.	.	.	.	6041
.	.	.	.	.	5108
.	.	.	.	.	4537
.	.	.	.	.	13807
.	.	.	.	.	57
.	.	.	.	.	10
.	.	.	.	.	.
.	.	.	.	.	.
.	.	.	.	.	6048
.	.	.	.	.	.
.	.	.	.	.	2306
.	.	.	.	.	.
.	.	.	.	.	.
.	.	.	.	.	429
.	.	.	.	.	.
.	.	.	.	.	7331
.	.	.	.	.	.
.	.	.	.	.	.
.	.	.	.	.	.
.	.	.	.	.	.
.	.	.	.	.	.
.	.	.	.	.	.
.	.	.	.	.	.
.	.	.	.	.	.
.	.	.	.	.	.
.	.	.	.	.	.

767	720	747			

752	734	745			

832	801	774
	823	
	714	
	733	

791

804 839

743	758	800
762		
935		818
817	817	791
686	785	813
724	728	609

844 787

.	.	.	.	.	.
1179	1112	1087	.	.	.
.	.	.	.	.	.
.	.	.	.	.	.
.	.	.	.	.	.
.	.	.	.	.	.
.	.	.	.	.	.
.	.	.	.	.	.
.	.	.	.	.	.
.	.	.	.	.	.
.	.	.	.	.	.
.	.	.	.	.	.
.	.	.	.	.	.
.	.	.	.	.	.
.	.	.	.	.	.
.	.	.	.	.	.
.	.	.	.	.	.
.	.	.	.	.	.
.	.	.	.	.	.
.	.	.	.	.	.
.	.	.	.	.	.
.	.	.	.	.	.
.	.	.	.	.	.
.	.	.	.	.	.
716	769	736	.	.	.
.	.	.	.	.	.
.	.	.	.	.	.
.	.	.	.	.	.
849	.	.	.	.	.
.	.	.	.	.	.
.	.	.	.	.	.
.	.	.	.	.	.
.	.	.	.	.	.
755	762	717	.	.	.
.	.	.	.	.	.
.	.	.	.	.	.
691	.	.	.	.	.
.	.	.	.	.	.
732	726	726	.	.	.
802	844	831	.	.	.
.	.	.	.	.	.
779	.	879	.	.	.
765	758	.	.	.	.
.	.	.	.	.	.
.	.	.	.	.	.
.	.	.	.	.	.
926	.	.	.	.	.
645	.	.	.	.	.
.	.	.	.	.	.
.	.	.	.	.	.
841	.	.	.	.	.
949	.	.	.	.	.
.	.	.	.	.	.
.	.	.	.	.	.
753	.	.	.	.	.
.	.	.	.	.	.
855	.	.	.	.	.
.	.	.	.	.	.
877	.	.	.	.	.

771	752	769	.	.	.
759	.	.	.	.	.
.	.	.	.	.	.
.	.	.	.	.	.
.	.	.	.	.	.
.	.	.	.	.	.
780	.	.	.	.	.
.	.	.	.	.	.
811	.	.	.	.	.
487	.	.	.	.	.
.	.	.	.	.	.
806	876	.	.	.	.
810	.	.	.	.	.
.	.	.	.	.	.
.	.	.	.	.	.
.	.	.	.	.	.
.	.	.	.	.	.
.	.	.	.	.	.
.	.	.	.	.	.
921	.	.	.	.	.
.	.	.	.	.	.
785	.	.	.	.	.
707	.	.	.	.	.
.	.	.	.	.	.
.	.	.	.	.	.
760	.	.	.	.	.
720	834	.	.	.	.
716	.	.	.	.	.
.	.	.	.	.	.
.	779	720	.	.	.
720	.	.	.	.	.
.	.	.	.	.	.
1122	.	.	.	.	.
730	.	.	.	.	.
.	.	.	.	.	.
778	871	.	.	.	.
786	.	.	.	.	.
.	767	.	.	.	.
729	760	778	.	.	.
.	793	.	.	.	.
.	750	.	.	.	.
.	.	.	.	.	.
0	721	.	.	.	.
720	708	.	.	.	.
.	.	.	.	.	.
.	.	.	.	.	.
.	803	.	.	.	.
.	720	.	.	.	.
.	.	.	.	.	.
.	.	.	.	.	.
.	.	.	.	.	.
.	786	997	.	.	.
720	720	.	.	.	.
.	705	.	.	.	.
.	737	.	.	.	.
.	797	.	.	.	.
.	805	.	.	.	.
.	686	.	.	.	.
754	793	797	.	.	.

.	766	785	.	.	.
.	752	.	.	.	.
.	770	821	.	.	.
.	785	.	.	.	.
.	.	.	.	.	.
.	.	.	.	.	.
.	.	.	.	.	.
.	724	810	.	.	.
.	801	.	.	.	.
.	864	.	.	.	.
.	798	847	.	.	.
.	.	.	.	.	.
.	900	.	.	.	.
.	772	.	.	.	.
.	.	.	.	.	.
.	.	.	.	.	.
.	.	.	.	.	.
.	718	774	.	.	.
.	741	.	.	.	.
.	.	.	.	.	.
.	1340	.	.	.	.
.	854	750	.	.	.
.	.	.	.	.	.
.	.	775	.	.	.
.	.	.	.	.	.
.	690	773	.	.	.
.	.	.	.	.	.
.	.	.	.	.	.
.	.	720	.	.	.
.	.	.	.	.	.
.	.	.	.	.	.
.	.	.	.	.	.
.	.	1082	.	.	.
.	.	703	.	.	.
.	.	.	.	.	.
.	.	781	.	.	.
.	.	826	.	.	.
.	.	783	.	.	.
.	.	721	.	.	.
.	.	723	.	.	.
.	.	.	.	.	.
.	.	1051	.	.	.
.	.	.	.	.	.
.	.	720	.	.	.
.	.	793	.	.	.
.	.	.	.	.	.
.	.	805	.	.	.
.	.	797	.	.	.
.	.	725	.	.	.
.	.	783	.	.	.
.	.	.	.	.	.
.	.	1070	.	.	.
.	.	750	.	.	.
.	.	.	.	.	.
.	.	.	.	.	.
.	.	.	.	.	.
.	.	721	.	.	.
.	.	842	.	.	.
.	.	780	.	.	.

.	.	120	.	.	.
.	.	731	.	.	.
.	.	723	.	.	.
.	.	815	.	.	.
.	.	.	.	.	.
.	.	.	.	.	.
.	.	787	.	.	.
.	.	.	.	.	.
.	.	.	.	.	.
.	.	749	.	.	.
.	.	799	.	.	.
.	.	875	.	.	.
.	.	.	.	.	.
.	.	784	.	.	.
.	.	741	.	.	.
.	790	772	.	.	.
.	.	906	.	.	.
.	.	.	.	.	.
.	.	.	.	.	.
.	.	.	.	.	.
.	.	750	.	.	.
.	.	.	.	.	.
.	.	.	.	.	.
.	.	.	.	.	.
.	.	.	.	.	.

steps_2019_5	steps_2019_6	steps_2019_7	steps_2019_8	steps_2019_9	steps_2019_10	steps_2019_11
.	.	.	.	.	.	.
.	.	1905	3382	1077	3116	4225
6832	.	.	9157	8564	11183	.
7030	4837	.	6530	9564	13627	.
.	.	3253	.	5518	.	.
.	.	.	.	.	5423	.
.	.	.	.	.	6482	.
.	.	.	.	.	28035	.
.	.	.	.	.	.	.
.	4188	.	.	14082	.	.
7030	8432	4232	.	.	.	.
.	.	.	9321	.	.	.
6928	.	.	.	.	.	.
.	.	.	.	.	.	.
.	.	.	.	.	10327	.
.	3880	5648	6952	6277	7432	5675
.	.	.	.	.	.	.
.	.	.	.	.	.	.
.	.	.	.	.	.	.
10337	.	.	.	.	.	.
.	.	.	.	.	.	.
.	.	.	.	.	.	15880
6872	7029	.	.	5706	7663	.
44	5671	8169	4420	.	.	.
.	.	.	.	.	9439	959
.	.	.	.	.	.	.
9329	.	.	.	.	.	.
.	.	.	.	.	.	.
.	57	.	.	.	.	.
.	.	.	.	.	.	.
11996	.	.	.	1865	9350	4093
4676	.	.	.	.	.	.
.	.	.	.	.	.	.
.	.	.	.	.	.	.
.	.	.	5938	.	6277	.
.	.	.	.	.	.	.
8557	8807	.	8402	.	.	10703
.	8326	.	8273	.	.	.
.	.	.	.	.	.	.
.	.	.	.	.	.	.
1370	1661	1306	1145	1594	2012	2440
.	.	.	.	.	.	.
.	.	11336	.	5523	.	.
.	.	.	.	.	.	.
.	.	.	.	.	.	.
7234	.	.	.	.	.	.
.	.	.	.	6545	.	.
.	.	.	.	.	.	483
.	.	.	.	.	.	.
.	.	.	.	.	.	.
.	.	.	3595	.	.	.
.	.	8166	.	.	.	.
1676	.	.	.	.	.	.
.	.	.	.	2137	.	.
.	6723	.	.	.	.	.
.	.	.	.	.	.	.
.	.	.	.	.	.	.
.	3800	.	.	.	.	.

.		.		.		.			10100		11535	.	
.		.		.		.		.		.		.	
.		.		.		.				.		.	
.		.			5917	.		.		.		.	
.		.		.		.			12335	.		.	
	1763		2701		1954		1538		798	.		.	
.		.		.		.		.		.		.	
.		.		.		.		.		.		.	
.		.			3956	.		.		.		.	
	9268		8524		9245		7699		8597		9840		9447
.		.		.		.		.		.		.	
.		.		.		.		.		.		.	
.		.		.		.		.		.		.	
	5548	.			11496	.			11434	.		.	
.		.		.		.		.		.		.	
.			2546	.		.		.		.		.	
.		.		.			12174		8599	.		.	
	4357	.		.		.		.		.		.	
.		.		.			920	.		.		.	
.			6721	.			4899		12288		7503		4945
	4773		4890		88		71		506	.			9801
.		.			9253	.		.		.		.	
.		.		.		.		.		.		.	
.		.		.		.		.		.		.	
.			4762	.			1532	.			5831		14977
	5403	.		.			5220		4543	.		.	
	10766	.		.		.		.		.		.	
.		.		.		.		.		.		.	
	17859		10848		15655		10669		11632		11996		13217
	2804	.		.		.			1828	.		.	
.		.		.		.		.			7089	.	
.		.		.		.		.		.		.	
.		.		.		.		.		.		.	
.		.		.		.		.		.		.	
.		.			144	.		.		.		.	
.		.		.		.		.		.		.	
.		.		.		.		.		.		.	
.		.			1250	.		.		.		.	
	17207	.			19138		2594	.		.			9540
.		.		.		.		.		.		.	
.		.		.		.		.		.		.	
	4097		2053		3459	.		.		.		.	
	4506	.		.		.		.		.		.	
.		.		.		.		.		.			2509
.		.		.		.		.		.		.	
	1175	.		.		.		.		.		.	
	1749	.			6600		6655	.			11843	.	
.		.		.		.			3328	.		.	
.		.		.		.		.		.		.	
	2707	.		.		.		.		.			6964
.		.		.		.		.		.		.	
.		.		.		.		.		.		.	
.		.		.		.		.		.		.	
.		.		.		.			1838	.		.	
	6119	.		.			6091		3174		5351	.	
.		.		.		.		.		.			13413
.			6034	.		.		.		.		.	
	9734		5347	.		.		.		.		.	
	2996	.		.		.		.		.		.	

.	.	.	.	.	4925	5705
.	.	2531	.	.	.	.
.	.	.	.	916	.	.
.	.	.	.	.	.	.
.	.	.	.	.	.	.
.	.	.	.	.	10946	.
11637	11448	11078	13873	12355	11580	12833
.	.	.	5673	.	3455	.
.	.	.	.	.	.	.
.	.	.	6374	.	4877	.
.	.	.	.	.	.	.
.	7197	.	3563	8116	11424	6005
2952	.	.	.	.	.	.
.	3313	.	.	.	.	.
.	.	.	.	.	.	.
270	191	.	.	.	.	.
.	.	.	.	.	.	.
3341	679	.	.	.	2722	.
1073	1904	2234	1939	1415	3549	1671
1779	3018	.	.	1535	3823	4656
.	.	.	.	.	.	.
.	1002	.	.	6566	.	.
.	.	.	.	.	.	.
4929	.	6610	.	.	.	9257
.	.	.	.	.	.	.
.	.	.	.	.	.	.
.	.	.	.	.	.	.
7394	.	13872	12476	11348	15357	.
1743	2039	2461	1965	2382	2400	2058
.	.	.	.	.	.	.
6249	7525	6781	6024	9206	7910	.
.	.	.	4670	8102	4860	4174
8831	7322	8989	.	8228	4948	14192
.	5667	.	2899	.	3912	.
.	.	.	.	.	.	.
.	.	.	.	.	.	.
.	.	.	.	.	.	.
.	.	.	.	.	.	.
.	.	.	22	.	.	.
.	.	.	.	.	.	.
5506	5481	3736	4008	4923	5833	4182
.	.	.	.	6551	9022	7220
.	.	.	.	.	.	.
.	.	.	.	.	.	.
.	.	.	2526	.	.	.
.	.	.	.	.	.	.
.	.	.	.	.	.	.
.	.	9806	7458	.	.	.
579	.	5539	5476	.	.	.
.	.	.	.	.	.	.
.	.	.	.	.	.	.
.	.	.	.	.	.	.
.	.	.	.	.	.	.
.	.	.	.	.	.	.
.	.	.	.	.	.	.
.	.	.	.	.	.	.
.	.	.	.	.	.	.
.	.	.	.	.	.	.
4333	3797	5927	4549	5790	3638	4038

9083	2947	20				
		1113				
3206	8145	5512	6133	8058	8204	8154
6537	5972	2430	2789	3000	4581	2647
		1472				
7089						
4164				4154		6776
9803						
2702						
532						
6615	8463	293				
314						
2484	4178	2640	1230	1661		
2406		821				
1365						
3678	4673	5500		8406		
8351						
10592	6806	5945	8909	5356		

.	7250	.	.	.	.	.
.	13284	.	.	.	.	.
.	11889	14426	15860	11570	11846	15298
.	111	.	.	.	.	.
.	.	.	.	.	.	.
.	4474	.	.	.	.	.
.	2523	3799	.	.	.	.
.	.	.	.	.	.	.
.	.	.	.	.	.	.
.	3565	.	.	.	.	.
.	11224	.	.	.	.	.
.	7644	3800	.	.	.	.
.	7184	3827	20	.	.	.
.	.	.	.	.	.	.
.	.	.	.	.	.	.
.	.	667	3397	2279	3810	2391
.	.	1939	2993	.	.	.
.	.	4994	.	.	.	.
.	.	8959	.	.	.	.
.	.	.	.	.	.	.
.	.	3146	.	.	.	.
.	.	.	.	.	.	.
.	.	456	.	.	.	.
.	.	.	.	.	.	.
.	.	3794	.	.	.	.
.	.	6060	4753	.	.	.
.	.	514	.	.	.	.
.	.	29	.	.	.	.
.	.	.	.	.	.	.
.	.	.	208	281	.	.
.	.	.	6678	14236	.	.
.	.	.	3356	.	.	.
.	.	.	.	.	.	.
.	.	.	.	.	.	.
.	.	.	.	.	.	.
.	.	.	4070	.	.	.
.	.	.	.	.	.	.
.	.	.	5119	.	.	.
.	.	.	780	.	.	.
.	.	.	4503	.	.	.
.	.	.	.	.	.	.
.	.	.	.	.	.	.
.	.	.	.	.	.	.
.	.	.	2937	.	.	.
.	.	.	4510	.	.	.
.	.	.	54	.	.	.
.	.	.	.	.	.	.
.	.	.	.	.	.	.
.	.	.	.	.	.	.
.	.	.	2488	940	2882	295
.	.	.	.	.	.	.
.	.	.	3437	10379	9319	8385
.	.	.	.	4367	3409	.
.	.	.	.	.	.	.
.	.	.	.	.	.	.
.	.	.	.	2877	.	.
.	.	.	.	.	.	.
.	.	.	.	3927	5595	5154
.	.	.	.	888	.	.

.	.	.	.	1085	.	.
.	.	.	.	10258	.	.
.	.	.	.	5022	5341	4987
.	.	.	.	7968	.	9113
.	.	.	.	4182	.	.
.	.	.	.	.	.	.
.	.	.	.	.	.	.
.	.	.	.	8798	12851	13530
.	.	.	.	.	.	.
.	.	.	.	2384	1145	.
.	.	.	.	8037	6337	1974
.	.	.	.	9897	6576	.
.	.	.	.	.	.	.
.	.	.	.	55	.	.
.	.	.	.	1640	.	.
.	.	.	.	.	.	.
.	.	.	.	.	10549	.
.	.	.	.	.	4165	2405
.	.	.	.	.	645	.
.	.	.	.	.	5592	.
.	.	.	.	.	.	.
.	.	.	.	.	155	93
.	.	.	.	.	1170	.
.	.	.	.	.	11578	1889
.	.	.	.	.	6052	.
.	.	.	.	.	945	.
.	.	.	.	.	4931	.
.	.	.	.	.	272	.
.	.	.	.	.	4109	2945
.	.	.	.	.	.	.
.	.	.	.	.	3744	.
.	.	.	.	.	10306	.
.	.	.	.	.	3770	1639
.	.	.	.	.	.	.
.	.	.	.	.	.	1639
.	.	.	.	.	.	243
.	.	.	.	.	.	.
.	.	.	.	.	.	6530
.	.	.	.	.	.	7404
.	.	.	.	.	.	.
.	.	.	.	.	.	2793
.	.	.	.	.	.	4582
.	.	.	.	.	.	3
.	.	.	.	.	.	.
.	.	.	.	.	.	11
.	.	.	.	.	.	4518
.	.	.	.	.	.	.
.	.	.	.	.	.	3431
.	.	.	.	.	.	1454
.	.	.	.	.	.	.
.	.	.	.	.	.	.
.	.	.	.	.	.	.
.	.	.	.	.	.	.
.	.	.	.	.	.	.
.	.	.	.	.	.	.
.	.	.	.	.	.	.
.	.	.	.	.	.	.
.	.	.	.	.	.	.
.	.	.	.	.	.	.

steps_2019_12	steps_2019_13	steps_2019_14	steps_2019_15	steps_2019_16	steps_2019_17
.	3674	4008	.	.	.
.	.	888	7708	10	7274
10169	10055	.	997	11237	10114
5858	3129	6319	8715	6109	4504
.	.	.	.	.	.
.	.	.	.	.	5951
7716	.	.	.	.	.
.	.	.	.	.	.
.	6976	.	.	.	.
.	.	.	.	.	.
.	.	.	.	.	.
.	.	.	6418	.	.
.	.	.	.	.	324
.	.	.	.	.	.
.	.	.	.	.	.
.	7193	2026	5611	10461	8298
.	.	.	.	.	.
.	.	.	.	.	.
.	4460	.	.	.	.
7389	.	.	.	.	5096
.	.	.	.	.	.
.	.	.	.	.	.
.	.	.	6816	.	.
.	.	.	.	.	.
.	13625	.	13889	.	.
.	.	.	.	.	.
.	.	.	11084	.	.
.	.	.	.	.	.
.	.	.	.	.	.
.	.	.	.	.	.
.	.	5315	6230	.	.
.	.	7892	.	.	.
.	.	3104	.	.	.
16	.	.	.	.	8529
.	.	86	.	.	.
.	.	.	.	.	.
.	.	10310	.	.	.
.	.	.	.	.	.
.	.	.	.	.	.
.	.	.	.	.	.
1871	2026	1701	1878	1803	1412
.	.	.	.	5515	.
.	.	.	.	.	.
.	.	.	.	.	.
4334	3543	5353	5589	6656	6508
.	.	.	.	.	.
.	.	.	.	.	.
.	.	.	.	.	.
.	.	.	.	.	.
.	.	.	.	.	.
.	.	.	.	.	.
.	.	.	.	.	.
.	.	.	.	.	.
.	.	.	1603	2357	.
.	.	.	.	.	.
.	.	.	.	.	.
.	.	.	.	.	.
.	.	.	.	.	.

	10019				
		2344			
9097	12497	8526	12310	10075	9518
	10201				
				5184	
2951	16259	10309		3276	6650
				6806	
					10917
12500	6617	15046	9676	10039	11355
	17607				
	4393				
	2076	681		729	3714
		439	381	452	
	844				
		2422			
4427			126		
	4986				
		870			
				3929	
5618				9835	
				12701	

.	.	.	.	.	.
.	.	.	.	3618	2657
16986	.	.	.	.	.
.	.	.	.	4197	.
.	.	.	.	.	.
9566	.	.	.	.	.
10598	12053	11302	11200	16388	11957
.	10202	.	.	.	.
.	.	.	.	.	.
.	15967	42	.	67	.
.	11	.	.	.	.
1883	6997	5302	1531	.	.
.	.	.	.	.	9
.	.	.	.	.	.
.	.	.	.	.	.
.	.	.	.	.	.
9553	.	.	.	.	.
.	.	.	.	.	.
2395	2895	3521	1843	385	2318
3219	.	.	5611	3155	3025
.	.	.	.	.	.
.	.	.	.	.	.
8679	.	5530	4140	.	.
.	.	.	.	.	.
.	.	.	.	.	.
.	.	.	.	.	.
.	.	.	4952	.	.
39	8058	.	10273	.	.
3528	1478	1515	2715	3947	3345
.	.	.	.	.	.
.	.	.	.	.	.
.	.	.	.	.	.
8071	.	.	13405	.	.
.	.	.	.	.	.
.	.	.	.	.	.
.	12672	.	.	.	.
.	.	.	.	.	.
.	.	.	488	.	.
.	.	.	44	.	.
.	.	.	.	.	.
3595	5683	4489	5734	4622	4036
.	.	.	.	.	.
.	.	.	.	.	.
.	.	.	.	.	.
.	.	.	.	.	.
.	.	.	.	.	.
.	.	.	.	.	.
.	.	.	.	.	.
.	.	.	.	.	.
.	.	.	.	.	.
.	.	.	.	.	.
.	.	.	.	.	.
.	.	.	.	.	.
.	.	.	.	.	.
.	.	.	.	.	.
.	.	.	.	.	.
.	.	.	.	.	.
.	.	.	.	.	.
5162	3189	5827	3864	3279	3168

8824					
	200				
2025	1990	3013	5783	2558	5714
4050		5191		5069	
	7754				

15643	12671	11765	11269	12007	11472
2032					
				207	
654		318			
8783	9553	9210	6600	7851	8894
					5884
5149	6559	6092	5461	6555	5354

.	.	.	.	.	.
.	.	.	.	.	.
2584	.	.	.	.	.
.	12311	.	8734	.	.
.	.	.	.	.	.
.	.	.	.	.	.
.	.	.	.	.	.
7370	13648	10581	.	.	.
.	.	.	.	.	.
.	.	.	.	.	.
.	.	.	.	.	.
.	.	.	.	.	.
.	.	.	.	.	.
.	.	.	.	.	.
.	.	.	.	.	.
.	.	.	.	.	.
.	.	.	.	.	.
7112	3684	12133	7394	.	.
.	.	.	.	.	.
.	.	.	.	.	.
.	.	.	.	.	.
.	.	.	.	.	.
.	.	.	.	.	.
.	.	.	.	.	.
.	.	.	.	.	.
.	.	.	.	.	.
.	.	.	.	.	.
.	.	.	.	.	.
.	.	.	.	.	.
.	.	.	.	.	.
.	.	.	.	.	.
.	.	.	.	.	.
.	.	.	.	.	.
.	.	.	.	.	.
.	.	.	.	.	.
.	.	.	.	.	.
.	.	.	.	.	.
.	.	.	.	.	.
.	.	.	.	.	.
.	.	.	.	.	.
.	.	.	.	.	.
.	.	.	.	.	.
.	.	.	.	.	.
.	.	.	.	.	.
.	.	.	.	.	.
2527	.	.	.	.	.
.	.	.	.	.	.
2207	.	.	.	.	.
.	.	.	.	.	.
.	.	.	.	.	.
11607	6862	8793	9671	8326	8376
3681	.	.	.	.	.
.	.	.	.	.	.
10939	.	.	.	.	.
5151	698	599	.	298	4870
495	.	.	.	.	.
.	.	.	.	.	.
153	.	.	.	.	.
1639	.	.	99	.	.

.	.	.	.	.	.
9573	.	.	.	.	.
1045	.	.	.	.	.
8543	.	.	.	.	.
2185	3374	.	.	.	.
.	.	.	.	.	.
174	.	.	.	.	.
.	.	.	.	.	.
5200	.	.	.	.	.
143	.	.	.	.	.
.	.	.	.	.	.
4293	.	.	.	.	.
.	.	.	.	.	.
.	.	.	.	.	.
1405	.	.	.	.	.
4959	.	.	.	.	.
4507	.	.	.	.	.
1196	.	.	.	.	.
1683	.	.	.	.	.
.	42	.	.	.	.
.	9842	8159	6236	3499	5663
.	.	.	.	.	.
.	2721	.	.	.	.
.	3292	7897	.	.	.
.	2147	.	.	.	.
.	4721	.	.	.	.
.	4879	.	.	.	.
.	8197	5767	.	.	.
.	.	.	.	.	.
.	10750	13162	11018	13317	13905
.	3964	2192	1257	.	5903
.	2044	2038	.	.	.
.	3462	961	.	.	.
.	.	.	.	.	.
.	4701	.	.	.	.
.	384	6006	32	2237	.
.	1707	864	.	.	.
.	.	.	.	.	.
.	.	.	.	.	.
.	.	15	.	.	.
.	.	.	.	.	.
.	.	.	.	.	.
.	.	.	.	.	.
.	.	9025	5866	.	.
.	.	5129	.	.	.
.	.	.	.	.	.
.	.	4054	.	.	.
.	.	8494	.	.	3108
.	.	5717	159	.	8986
.	.	.	.	.	.
.	.	.	.	.	.
.	.	5283	.	.	.
.	.	3393	.	.	.
.	.	.	.	.	.
.	.	9	.	.	.
.	.	.	.	.	.
.	.	3597	.	.	.
.	.	2084	2858	1889	.
.	.	.	.	.	.

.	.	5579	.	.	.
.	.	.	17	.	.
.	.	.	.	.	.
.	.	.	1459	1971	1391
.	.	.	.	.	.
.	.	.	3338	.	.
.	.	.	1414	.	.
.	.	.	.	.	.
.	.	.	720	.	.
.	.	.	1077	.	.
.	.	.	.	.	.
.	.	.	1172	.	.
.	.	.	.	.	.
.	.	.	5161	.	.
.	.	.	6534	.	.
.	.	.	.	.	.
.	.	.	2724	.	.
.	.	.	16	.	.
.	.	.	.	.	.
.	.	.	6018	5485	8859
.	.	.	.	.	.
.	.	.	.	7095	.
.	.	.	.	2111	.
.	.	.	.	4839	.
.	.	.	.	.	.
.	.	.	.	506	.
.	.	.	.	469	.
.	.	.	.	3567	.
.	.	.	.	319	.
.	.	.	.	3615	2626
.	.	.	.	4929	.
.	.	.	.	2031	.
.	.	.	.	2957	.
.	.	.	.	.	.
.	.	.	.	1626	.
.	.	.	.	632	.
.	.	.	.	10783	.
.	.	.	.	10	2871
.	.	.	.	.	.
.	.	.	.	.	2
.	.	.	.	.	.
.	.	.	.	.	1002
.	.	.	.	.	6492
.	.	.	.	.	11374
.	.	.	.	.	58
.	.	.	.	.	4968
.	.	.	.	.	1328
.	.	.	.	.	3777
.	.	.	.	.	2050
.	.	.	.	.	9612
.	.	.	.	.	.
.	.	.	.	.	4259
.	.	.	.	.	.
.	.	.	.	.	.
.	.	.	.	.	.
.	.	.	.	.	.
.	.	.	.	.	.
.	.	.	.	.	.
.	.	.	.	.	.

steps_2020_2	steps_2020_3	steps_2020_4	steps_2020_5	steps_2020_6	steps_2020_7	steps_2020_8
.	.	.	.	.	.	.
.	.	.	.	.	.	.
.	.	.	.	.	.	.
.	.	.	.	.	.	.
.	.	.	.	.	.	.
.	.	.	831	.	.	.
.	.	.	.	.	.	.
.	.	.	.	.	.	.
.	.	.	.	.	.	.
.	.	.	.	.	.	.
.	.	.	.	.	.	.
.	.	.	.	.	.	.
.	.	.	.	.	.	.
.	.	.	.	.	.	.
.	.	.	.	.	.	.
.	5898	.	.	8500	317	.
.	.	.	.	.	.	.
.	.	.	.	.	.	.
.	.	.	.	.	.	.
.	.	.	.	.	.	.
.	.	.	6653	.	.	.
.	.	.	.	.	.	.
.	.	.	.	.	.	.
.	.	.	.	.	.	.
.	.	.	.	.	.	.
.	.	.	.	.	.	.
.	.	.	.	.	.	.
.	.	.	.	.	.	.
.	.	.	.	.	.	.
.	.	.	.	.	.	.
.	.	.	.	.	.	.
.	.	.	.	.	.	.
.	.	.	.	.	.	.
.	.	.	.	.	.	.
.	.	.	.	.	.	.
.	.	.	.	.	.	.
.	.	.	.	.	.	.
.	.	.	8303	.	.	.
.	.	.	.	.	.	.
.	.	.	.	.	.	.
2336	1888	1740	2252	1118	1579	1606
.	.	.	.	.	.	.
.	.	.	.	.	.	.
.	.	2067	.	.	.	.
.	.	.	.	.	.	.
.	.	.	.	.	.	.
.	.	.	.	.	.	.
.	.	.	.	.	.	.
.	.	.	.	.	.	.
.	.	.	.	.	.	.
.	.	.	.	.	.	.
.	.	.	.	.	.	.
.	.	.	.	.	.	.
.	.	.	.	.	.	1371
.	.	.	.	.	.	.
.	.	.	.	.	.	.
.	.	.	.	.	.	.
.	.	.	.	.	.	.

							3393
			30				
7519	5997				4841		4904
18398	18745	17153	18677	12578	22168	14987	
25							

				3434		
				11363	7168	
6757	6554	5886	5652	6113	5541	7194
6827	5800		5065			6438
3202	2983	3159	2737	3196	2544	1203
						874
3983	4689	5242	3602	5316	5081	4153

14078	8098	8994	11764	6300	8240	12367

		131	2539			
4201					4883	
		4365		2484		
6229	7515	7762	10874	9346	12100	9292

.		.		.		.		.		.		.	
.		.		.		.		.		.		.	
	3799		4283		3588		3095		5917		2089		1499
.		.		.		.		.		.		.	
.		.		.		.			9299	.		.	
.			33	.		.			928	.		.	
	4403	.		.		.		.		.		.	
	10945		6653		7577		9072		4687		7085		641
.		.		.			21438	.		.		.	
.		.			7006	.		.		.		.	
.		.			4407		6403		5602		2499		5469
.		.			8960	.			7807	.		.	
	4401	.		.		.		.			6762	.	
.		.		.		.		.		.		.	
.		.		.			4597	.		.		.	
	816	.		.		.		.		.		.	
.			3148	.		.		.			7691	.	
	6695	.		.		.		.		.		.	
.		.		.		.		.		.		.	
.		.		.			121	.			6575	.	
	3456		10814	.		.		.		.		.	
	3981	.		.		.		.		.		.	
.		.		.			843	.		.		.	
.		.			8476	.		.		.		.	
	106	.		.		.		.		.		.	
	4732		5987		3154	.			5026		1889	.	
.		.		.		.		.		.		.	
	1577	.		.		.		.		.		.	
	2302	.		.		.		.		.		.	
	3910		3876		5505		3761		6884		8130		4594
.		.		.		.		.			4501	.	
.		.		.		.		.		.		.	
.		.		.			9640	.		.		.	
	994		1126		1209		1137		2850		923	.	
.		.			841	.		.		.		.	
.		.		.		.		.		.		.	
.		.		.		.		.		.		.	
	10279	.		.		.		.		.		.	
	8418		4293		4820		3772	.			4751	.	
	10031	.			11120	.		.		.		.	
	3200		3196		7051		3127		5025		7022		5517
.		.		.		.		.		.		.	
	1576		2012		3013		1749		308		114		244
.		.		.		.		.		.		.	
	5611		1295		849	.		.			1630	.	
	34	.		.		.		.		.		.	
.		.		.		.		.		.		.	
	3169	.			1959	.		.		.		.	
	3390	.		.		.		.		.		.	
	5000	.		.		.		.		.		.	
.		.		.		.		.		.		.	
.		.		.		.		.		.		.	
.		.		.		.		.		.		.	
	4677	.		.		.		.		.		.	
.		.		.		.		.		.		.	
	3530	.		.		.		.		.		.	
	1506		2218		2203		1726	.		.		.	
.		.		.		.		.		.		.	
	730	.		.		.		.		.		.	

.	.	.	.	.	.	.
1611	.	.	.	.	.	.
.	.	.	.	.	.	.
7240	5400	.	.	.	.	.
6102	.	.	.	.	.	.
1907	3339	1832	2667	1409	3310	2922
9433	.	.	.	.	.	.
4825	.	.	.	.	.	.
9088	.	.	.	.	.	.
.	.	.	.	.	.	.
4553	.	.	.	.	.	.
8309	5408	.	.	.	.	.
.	.	.	.	.	.	.
2840	.	.	.	.	.	.
3226	.	.	.	.	.	.
810	.	.	.	.	.	.
12324	.	.	.	.	.	.
.	.	.	.	.	.	.
6382	8458	9837	.	.	.	.
7851	6106	.	.	.	.	.
361	.	.	.	.	.	.
6696	3150	.	.	.	.	.
4602	5715	.	.	.	.	.
2939	.	.	.	.	.	.
5966	7336	.	.	.	.	.
2851	.	.	.	.	.	.
919	.	.	.	.	.	.
3199	.	.	.	.	.	.
1101	.	.	.	.	.	.
144	.	.	.	.	.	.
.	.	.	.	.	.	.
2695	4892	.	.	.	.	.
.	.	.	.	.	.	.
1617	11487	.	7120	15083	.	.
.	.	.	.	.	.	.
843	.	.	.	.	.	.
70	.	.	.	.	.	.
2583	113	.	.	.	.	.
1605	2035	.	.	.	.	.
5258	.	.	.	.	.	.
.	7421	.	.	.	.	.
.	.	.	.	.	.	.
.	2078	.	.	.	.	.
.	2975	.	.	.	.	.
.	12432	.	.	.	.	.
.	.	.	.	.	.	.
.	1529	.	.	.	.	.
.	.	.	.	.	.	.
.	3646	.	.	.	.	.
.	1397	.	2114	126	.	.
.	3733	.	.	.	.	.
.	.	.	.	.	.	.
.	1872	.	.	.	.	.
.	.	.	.	.	.	.
.	24	.	.	.	.	.
.	.	.	.	.	.	.
.	2992	.	.	.	.	.
.	2946	.	.	.	.	.
.	.	.	.	.	.	.

.	1392	.	.	.	.	.
.	4057	7328	7090	.	.	.
.	7068	6360	3525	9312	.	5830
.	787	.	.	.	.	.
.	11417	.	.	.	.	.
.	1262	.	.	.	.	.
.	10150	.	.	.	.	.
.	2167	.	.	.	.	.
.	.	.	.	.	.	.
.	2357	.	.	.	.	.
.	.	.	.	.	.	.
.	3519	.	.	.	.	.
.	.	.	.	.	.	.
.	816	.	.	.	.	.
.	171	.	.	.	.	.
.	2871	.	.	.	.	.
.	116	.	.	.	.	.
.	7161	9833	.	.	.	.
.	3744	6210	3698	2290	5314	9376
.	2057	7734	6739	8218	10416	7395
.	2631	1186	1101	.	.	.
.	694	3675	5194	4290	22	.
.	161	.	.	.	.	.
.	.	2435	.	.	.	.
.	.	1602	2834	.	.	.
.	.	.	.	.	.	.
.	.	5097	.	.	.	.
.	.	5533	.	.	.	.
.	.	.	.	.	.	.
.	.	.	.	.	.	.
.	.	.	.	.	.	.
.	.	2465	.	.	.	.
.	.	7862	8460	.	.	.
.	.	1488	.	.	.	.
.	.	672	.	.	.	.
.	.	3810	.	.	.	.
.	.	7346	7445	6389	12433	.
.	.	.	.	.	.	.
.	.	2945	2265	.	.	.
.	.	.	.	.	.	.
.	.	.	.	.	.	.
.	.	6426	.	.	.	.
.	.	518	1885	.	.	.
.	.	273	.	.	.	.
.	.	6422	4847	5976	.	.
.	.	1649	.	.	.	.
.	.	.	.	.	.	.
.	.	2882	.	.	.	.
.	.	4453	146	.	.	.
.	.	.	.	.	.	.
.	.	5933	4371	.	.	.
.	.	135	.	.	.	.
.	.	.	.	.	.	.
.	.	5541	5545	3657	5130	.
.	.	898	.	.	.	.
.	.	.	.	.	.	.
.	.	.	2375	.	.	.
.	.	.	4292	.	.	.
.	.	.	10830	.	.	.

.	.	.	33	.	.	.
.	.	.	11331	.	.	6657
.	.	.	4867	12051	3663	12703
.	.	.	4355	.	.	.
.	.	.	1828	.	.	.
.	.	.	59	.	.	.
.	.	.	3	.	.	.
.	.	.	3	.	.	.
.	.	.	1826	.	.	.
.	.	.	.	.	.	.
.	.	.	3012	5325	.	.
.	.	.	9582	.	.	.
.	.	.	10219	.	.	.
.	.	.	8692	.	.	.
.	.	.	2046	.	.	.
.	.	.	.	.	.	.
.	.	.	7889	.	.	.
.	.	.	8668	51	.	.
.	.	.	8491	.	.	.
.	.	.	.	.	.	.
.	.	.	6128	7836	.	.
.	.	.	7895	.	.	.
.	.	.	3295	.	.	.
.	.	.	630	.	.	.
.	.	.	10882	.	.	.
.	.	.	.	.	.	.
.	.	.	.	.	.	.
.	.	.	5460	.	.	.
.	.	.	1533	.	.	.
.	.	.	.	.	.	.
.	.	.	.	.	.	.
.	.	.	11700	.	.	.
.	.	.	.	.	.	.
.	.	.	4039	1642	.	.
.	.	.	7145	.	.	.
.	.	.	7996	.	.	.
.	.	.	16205	.	.	.
.	.	.	.	49	.	.
.	.	.	.	.	.	.
.	.	.	.	.	.	.
.	.	.	.	.	.	.
.	.	.	.	1496	4443	.
.	.	.	.	5554	.	.
.	.	.	.	116	.	.
.	.	.	.	4071	.	.
.	.	.	.	2965	.	.
.	.	.	.	4428	.	.
.	.	.	.	7186	.	.
.	.	.	.	2180	3365	119
.	.	.	.	820	.	.
.	.	.	.	.	.	.
.	.	.	.	.	.	.
.	.	.	.	1181	55	.
.	.	.	.	1611	.	.
.	.	.	.	3076	.	.
.	.	.	.	635	.	.
.	.	.	.	7929	.	.
.	.	.	.	2788	.	.
.	.	.	.	6350	.	.

.	.	.	.	.	.	.
.	.	.	.	9451	.	.
.	.	.	.	1538	1028	876
.	.	.	.	7743	.	.
.	.	.	.	.	.	.
.	.	.	.	7140	9271	4052
.	.	.	.	4815	5634	.
.	.	.	.	1622	.	.
.	.	.	.	7350	.	7710
.	.	.	.	1286	.	.
.	.	.	.	7602	11782	.
.	.	.	.	97	.	.
.	.	.	.	.	.	.
.	.	.	.	8335	12707	.
.	.	.	.	.	.	.
.	.	.	.	.	1998	.
.	.	.	.	.	.	.
.	.	.	.	.	2901	.
.	.	.	.	.	51	.
.	.	.	.	.	7296	5454
.	.	.	.	.	363	.
.	.	.	.	.	.	.
.	.	.	.	.	605	.
.	.	.	.	.	.	.
.	.	.	.	.	6660	.
.	.	.	.	.	5161	.
.	.	.	.	.	14582	.
.	.	.	.	.	29	.
.	.	.	.	.	2500	.
.	.	.	.	.	9476	.
.	.	.	.	.	118	.
.	.	.	.	.	7889	.
.	.	.	.	.	.	.
.	.	.	.	.	.	.
.	.	.	.	.	94	38
.	.	.	.	.	33	.
.	.	.	.	.	5360	6491
.	.	.	.	.	9	.
.	.	.	.	.	.	.
.	.	.	.	.	.	.
.	.	.	.	.	.	29
.	.	.	.	.	.	11356
.	.	.	.	.	.	54
.	.	.	.	.	.	.
.	.	.	.	.	.	8308
.	.	.	.	.	.	416
.	.	.	.	.	.	.
.	.	.	.	.	.	.
.	.	.	.	.	.	1468
.	.	.	.	.	.	4504
.	.	.	.	.	.	.
.	.	.	.	.	.	.
.	.	.	.	.	.	7209
.	.	.	.	.	.	548
.	.	.	.	.	.	6227
.	.	.	.	.	.	1792
.	.	.	.	.	.	9810
.	.	.	.	.	.	6494
.	.	.	.	.	.	2498

94

80

steps_2020_9	steps_2020_10	steps_2020_11	steps_2020_12	steps_2020_13	steps_2020_14
.	.	.	.	.	.
.	.	.	.	.	.
.	.	.	.	.	.
.	.	.	.	.	.
.	.	.	.	.	.
.	.	.	.	.	.
.	.	.	.	.	.
.	.	.	.	.	.
.	.	.	.	.	.
.	.	.	.	.	.
.	.	.	.	.	.
.	.	.	.	.	.
.	.	.	.	.	.
.	.	.	.	.	.
.	.	.	.	.	.
.	2354	6094	1776	.	.
.	.	.	.	.	.
.	.	.	.	.	.
.	.	.	.	.	.
.	.	.	.	.	.
.	.	.	.	.	.
.	.	.	.	.	.
.	.	.	.	.	.
.	.	.	.	.	.
.	.	.	.	.	.
.	.	.	.	.	.
.	.	.	.	.	.
.	.	.	.	.	.
.	.	.	.	.	.
.	.	.	.	.	.
.	.	.	.	.	.
.	.	.	.	.	.
.	.	.	.	.	.
.	.	.	.	.	.
.	.	.	.	.	.
.	.	.	.	.	.
.	.	.	.	.	.
.	.	.	.	.	.
.	.	.	.	.	.
.	.	.	.	.	.
1573	1314	2119	1471	2112	901
.	.	.	.	.	.
.	.	.	.	.	.
.	.	.	.	.	.
.	.	.	.	.	.
.	.	.	.	.	.
.	4057	2086	.	.	.
.	.	.	.	.	.
.	.	.	.	.	.
.	.	.	.	.	.
.	.	.	.	.	.
.	.	.	.	.	.
.	.	.	.	.	.
.	.	.	.	.	.
.	.	.	.	.	.
.	.	.	.	.	.
.	.	.	.	.	.
.	.	.	.	.	.

		4060		4611	
922	1604	1895	1942	4202	5958
	16540				9805

1291<br>7028	6365	6235	6340	5523	4756
1911	1626	7151<br>3039	4345<br>1627	2524	2372
4948	3437	2455	4421	4370	3416

1041

11856	10636	13676	12224	7685	8204

				6911	327
			8409		
			1389		
	8280	18			
9971	11319	8309	8560	8053	10417

.	.	.	.	.	.	
.	.	.	.	.	.	
	3248 .	.	.	.	.	
.	.	.	.	.	.	
.	.	.	.	.	.	
.	.	.	.	.	.	
.	.	.	.	.	.	
	10040	9153	8426	18047	6540	9492
.	.	.	.	.	.	
.	.	.	.	.	.	
	4790	5330 .	.	3890 .	.	
.		4467 .	.	.	.	
	3543	3965 .		4317 .		1600
.	.	.	.	.	.	
.	.	.		1600 .	.	
.	.	.	.	.	.	
.	.	.	.	.	.	
	10292 .		25 .	.	.	
	795 .	.	.	.	.	
.	.	.		5543 .	.	
.	.	.	.	.	.	
.	.	.	.	.		4532
.	.	.	.	.	.	
	22272 .	.		381 .	.	
.	.	.	.	.	.	
.	.	.	.	.	.	
.		4408	4526 .		2234 .	
.	.	.		.	.	
.	.	.	.	.	.	
	3837	5311	3705	10485 .	.	
.	.		.	.	.	
.	.		636 .	.		24
.	.	.	.	.	.	
.	.	.	.	.	.	
.	.	.	.	.	.	
.	.	.	.	.	.	
.	.	.	.	.	.	
.	.	.	.	.	.	
	3950 .	.	.	.	.	
.	.	.	.	.	.	
	5373	3454	8976	6633	6336 .	
.	.	.	.	.	.	
.	.	.	.	.	.	
.	.	.	.	.	.	
	801 .		1682	1155 .		889
.	.	.	.	.	.	
.	.	.	.	.	.	
.	.	.	.	.	.	
.	.	.	.	.	.	
.	.	.	.	.	.	
.	.	.	.	.	.	
.	.	.	.	.	.	
.	.	.	.	.	.	
.	.	.	.	.	.	
.	.	.	.	.	.	
.	.	.	.	.	.	
.	.	.	.	.	.	
.	.	.	.	.	.	
.	.	.	.	.	.	

2638	3530

4114	4773		4441
7361	6637	1407	

3626					
9462					61
		4776			
		632			
	19282		5739		
1828					
579		219		84	

					1218
5051					
6165	662	5412	9608	839	5029
841	151	6369			126
1667		3716			
6806	6103				1090
746					
4792					

.	.	.	.	.	.
.	.	.	.	.	.
.	.	.	.	.	.
6338	796	654	3600	2917	.
.	.	.	.	.	.
.	.	.	.	.	.
7108	5578	3106	2719	1439	6754
4782	.	.	.	.	.
12051	.	.	.	.	.
4084	.	.	.	.	.
3010	.	.	.	.	.
112	.	.	.	.	.
5519	1184	.	.	.	.
70	.	.	.	.	.
.	.	.	.	.	.
.	.	.	.	.	.
2363	2549	3585	2194	.	.
.	.	.	.	.	.
883	.	.	.	.	.
18018	2566	.	.	.	.
1164	.	.	.	.	.
.	.	.	.	.	.
14	.	.	.	.	.
10	.	.	.	.	.
.	.	.	.	.	.
3053	3794	.	.	.	.
.	.	.	.	.	.
10481	.	.	.	.	.
.	.	.	.	.	.
.	3342	.	.	.	.
.	1353	3251	.	.	.
.	.	.	.	.	.
.	5467	7538	8571	8752	7095
.	.	.	.	.	.
.	3861	.	.	.	.
.	9215	.	.	.	.
.	98	.	.	.	.
.	2725	.	.	.	.
.	10	1137	1619	2594	2429
.	13239	.	.	.	.
.	.	.	.	.	.
.	1063	.	.	.	.
.	1496	.	.	.	.
.	1717	5388	.	.	.
.	.	.	.	.	.
.	2803	.	.	.	.
.	11243	4752	.	.	.
.	5189	2358	.	.	.
.	1825	.	.	.	.
.	2553	4405	6372	.	.
.	3457	.	.	.	.
.	16	.	.	.	.
.	8684	5054	.	.	.
.	23	227	.	.	.
.	1823	6083	4042	.	.
.	664	.	.	.	.
.	3068	2818	.	.	.
.	.	2588	.	.	.
.	.	3302	5262	.	.

.	.	4076	.	.	.
.	.	.	.	.	.
.	.	5454	.	.	.
.	.	1954	.	.	.
.	.	6396	5056	7823	2971
.	.	8951	.	.	.
.	.	.	.	.	.
.	.	3806	3612	2389	1300
.	.	4827	7737	3295	.
.	.	2427	45	4065	.
.	.	3231	.	.	.
.	.	6759	.	.	.
.	.	2416	.	.	.
.	.	376	.	.	.
.	.	9301	.	.	.
.	.	10911	.	.	.
.	.	.	.	.	.
.	.	234	.	.	.
.	.	3073	3302	3025	2273
.	.	.	.	.	.
.	.	7648	22264	.	.
.	.	.	.	.	.
.	.	3776	.	.	.
.	.	4476	971	.	.
.	.	.	.	.	.
.	.	226	.	.	.
.	.	69	.	.	.
.	.	69	.	.	.
.	.	5018	.	.	.
.	.	4020	.	.	4
.	.	7130	8439	.	.
.	.	.	74	.	.
.	.	.	7804	.	.
.	.	.	1569	.	.
.	.	.	1936	.	.
.	.	.	3932	.	.
.	.	.	.	.	.
.	.	.	1746	.	.
.	.	.	.	.	.
.	.	.	18913	17608	.
.	.	.	.	.	.
.	.	.	9974	.	.
.	.	.	6221	.	.
.	.	.	5346	4733	.
.	.	.	10226	610	.
.	.	.	.	.	.
.	.	.	.	.	.
.	.	.	11	.	.
.	.	.	4778	5492	.
.	.	.	.	.	.
.	.	.	12403	.	.
.	.	.	.	36	.
.	.	.	.	1976	.
.	.	.	.	2953	.
.	.	.	.	4675	.
.	.	.	.	8	.
.	.	.	.	1643	.
.	.	.	.	.	.
.	.	.	.	70	.

.	.	.	.	.	.
.	.	.	.	2151	3519
.	.	.	.	19	.
.	.	.	.	.	.
.	.	.	.	.	.
.	.	.	.	1737	.
.	.	.	.	697	.
.	.	.	.	16	.
.	.	.	.	4843	.
.	.	.	.	.	.
.	.	.	.	17	.
.	.	.	.	4738	1908
.	.	.	.	127	.
.	.	.	.	1160	.
.	.	.	.	.	11261
.	.	.	.	.	3381
.	.	.	.	.	716
.	.	.	.	.	4745
.	.	.	.	.	.
.	.	.	.	.	.
.	.	.	.	.	.
.	.	.	.	.	5271
.	.	.	.	.	.
.	.	.	.	.	2607
.	.	.	.	.	.
.	.	.	.	.	.
.	.	.	.	.	8956
.	.	.	.	.	.
.	.	.	.	.	7865
.	.	.	.	.	33
.	.	.	.	.	.
.	.	.	.	.	683
.	.	.	.	.	2159
.	.	.	.	.	.
.	.	.	.	.	8
.	.	.	.	.	7355
.	.	.	.	.	4283
.	.	.	.	.	919
.	.	.	.	.	1952
.	.	.	.	.	4990
.	.	.	.	.	13875
.	.	.	.	.	8125
.	.	.	.	.	1029
.	.	.	.	.	990
.	.	.	.	.	.
.	.	.	.	.	.
.	.	.	.	.	.
.	.	.	.	.	.
.	.	.	.	.	.
.	.	.	.	.	.
.	.	.	.	.	.
.	.	.	.	.	.
.	.	.	.	.	.
.	.	.	.	.	.
.	.	.	.	.	.
.	.	.	.	.	.
.	.	.	.	.	.
.	.	.	.	.	.
.	.	.	.	.	.

steps_2020_15	steps_2020_16	steps_2020_17
.	.	.
.	.	.
.	.	.
.	.	.
.	.	.
2583	.	.
.	.	.
.	.	.
.	.	.
.	.	.
.	.	.
.	.	.
.	.	.
.	.	.
.	.	.
11822	.	.
.	.	.
.	.	.
.	.	.
.	.	.
.	.	.
.	.	.
.	.	.
.	.	.
.	.	.
.	.	.
.	.	.
.	.	.
.	.	.
.	.	.
.	.	.
.	.	.
.	.	.
.	.	.
.	.	.
.	.	.
.	.	.
.	.	.
.	.	.
.	.	.
1003	2294	1697
.	.	.
.	.	.
.	.	.
.	.	.
.	.	.
.	.	.
.	.	.
.	.	.
.	.	.
.	.	.
.	.	.
.	.	.
.	.	41
.	.	.
.	.	.
.	.	.
.	.	.

2627

8158 12822 14576

6278	3544	5825
1964		
2064	1826	1974
5341	2106	2313

477

9702	5407	5353

	113	58
5551	7254	6659

.	.	.
.	3062	.
.	2181	.
.	.	.
.	.	.
.	.	.
.	.	.
5013	4004	6684
.	.	.
.	.	.
726	2899	4733
.	.	.
.	2241	.
.	.	53
.	.	.
.	.	.
.	.	.
.	.	.
.	.	.
.	.	.
.	.	.
.	.	.
.	.	.
969	246	193
.	.	.
2774	.	.
.	3298	.
.	.	.
.	.	.
.	.	.
.	.	.
.	.	.
.	.	.
.	.	.
.	.	.
.	.	.
.	.	.
.	.	.
.	.	.
.	.	.
.	.	.
.	.	.
.	.	.
.	.	.
.	97	.
.	.	.
.	.	.
.	.	.
.	.	.
.	.	.
.	.	.
.	.	.
.	.	.
.	.	.
.	.	.
.	.	.
.	.	.
.	.	.
.	.	.

5536	1688	4966

4526	7272	5925

3259		
654	1695	2465
4628	7000	8610
2294	26	3839

4203

1034	1566	2108

12501

.	.	.
910	2101	1564
.	.	.
.	.	.
.	.	.
.	.	.
.	.	.
.	.	.
.	.	.
.	.	.
.	.	.
199	.	.
.	.	.
.	.	.
.	.	.
1954	.	.
.	.	.
.	.	.
.	.	.
.	.	.
.	.	.
.	.	.
.	.	.
.	.	.
.	.	.
.	.	.
.	.	.
.	.	.
4870	.	.
.	.	.
.	.	.
.	.	.
.	.	.
.	.	.
.	.	.
.	.	.
1507	.	.
.	.	.
2282	542	194
5514	3745	4931
.	.	.
6564	.	7824
1228	1609	.
.	.	.
2	.	.
141	.	.
.	.	.
977	.	.
4883	.	1708
.	.	.
5524	.	.
3014	.	.
5304	.	.
1169	.	.
2997	.	.
7433	.	.
2768	.	.
761	.	.
.	.	.

516	723	1144
2490	.	.
3285	.	.
5620	.	.
.	.	.
.	.	.
72	.	.
8477	.	.
3601	.	.
.	.	.
4003	.	.
664	2005	.
1901	.	.
.	.	.
219	.	.
3247	.	.
.	.	.
.	.	.
.	.	.
4501	.	.
.	.	.
3525	.	.
.	.	.
.	.	.
9951	.	.
.	.	.
182	508	97
.	.	.
.	.	.
3979	5357	7122
3670	.	.
.	.	.
294	.	.
4143	.	.
.	.	.
191	2531	.
.	.	.
.	11924	.
3893	4717	5130
.	5952	.
.	.	.
.	8493	.
.	.	.
.	.	.
.	20	.
.	98	.
.	2532	.
.	344	.
.	23	.
.	6900	.
.	.	.
.	3231	5475
.	.	.
.	.	.
.	1713	.
.	.	.
.	5157	.
.	3381	3603
.	2626	3227

.		5809	7130
.		179	.
.		1009	2406
.		920	.
.		661	333
.		2333	.
.		3615	.
.	.		.
.		8	.
.		88	.
.		11542	11493
.		7734	.
.	.		.
.		11412	.
.		351	.
.	.		.
.		381	.
.		2757	2943
.	.		.
.		3085	.
.		658	.
.		210	1342
.		1221	.
.		25	1987
.		3456	.
.	.		.
.	.		90
.	.		14
.	.		.
.	.		42
.	.		139
.	.		2700
.	.		3400
.	.		.
.	.		1016
.	.		7572
.	.		8706
.	.		.
.	.		1410
.	.		1699
.	.		.
.	.		4053
.	.		5015
.	.		3981
.	.		4254
.	.		100
.	.		.
.	.		2597
.	.		.
.	.		.
.	.		4504
.	.		4342
.	.		52
.	.		8
.	.		160
.	.		2
.	.		112
.	.		6536
.	.		.

.	.		350
.	.		3933
.	.	.	
.	.		793
.	.	.	
.	.		4561
.	.		71
.	.		51
.	.		1130
.	.		188
.	.	.	
.	.		6394
.	.		4915
.	.		658
.	.		409
.	.		1600
.	.		16
.	.		453
.	.	.	
.	.	.	
.	.		16190
.	.		10553
.	.		117
.	.		1736
.	.		16304
